# Supplementary material for: Facilitating high quality acute care in resource-constrained environments: Perspectives of patients recovering from sepsis, their caregivers and healthcare workers in Uganda and Malawi
Source: PLOS Glob Public Health. 2022 Aug 15;2(8):e0000272. doi: 10.1371/journal.pgph.0000272 (PMC10021962; doi:10.1371/journal.pgph.0000272)
Supplement: S1 Text — (DOCX) [file pgph.0000272.s001.docx]

**S1 Text**

**The African Research Collaboration on Sepsis, Patient Experience Study Group**

Barbara Njamwaha^1^, Hilda Muwando^2^, Jacob Phulusa^1^, Lucy Keyala^1^, Madalitso Chiutsi^1^, Priscilla Haguma^2^, Sharon Nyesiga^2^, Sylvester H. Kaimba^1^ and Solomon Kyakuha^3^.

^1^ Malawi-Liverpool Wellcome Trust Clinical Research Programme, Chichiri, Blantyre 3, Malawi

^2^ Walimu, Coral Crescent, Kololo, Kampala, Uganda

^3^ Hoima Regional Referral Hospital, Uganda
