# Supplementary material for: Facilitating high quality acute care in resource-constrained environments: Perspectives of patients recovering from sepsis, their caregivers and healthcare workers in Uganda and Malawi
Source: PLOS Glob Public Health. 2022 Aug 15;2(8):e0000272. doi: 10.1371/journal.pgph.0000272 (PMC10021962; doi:10.1371/journal.pgph.0000272)
Supplement: S1 File — (DOCX) [file pgph.0000272.s002.docx]

|  | | | |
| --- | --- | --- | --- |
| **ARCS patient, caregiver, and healthcare workers’ experience of sepsis care report (Malawi)** | | | |
| **Thematic areas** | **Summary** | | **Quotes** |
| 1. **AMENITIES OF CARE** | This defines sepsis care delivery **context** including equipment and materials, availability of medication, the hospital environment and, adequacy of HCWs. | |  |
| 1. **Accommodation in the hospital is limited and lack basic necessities to make it conducive** | Participants (including patients, guardians and HCWs raised the problem of accommodation in the hospital. Among others this included lack of specific space for guardians to sleep (usually on the floor in between the patient beds). This turns to be hazardous and uncomfortable for the guardians. Others mentioned the arrangement of mixing patients in the ward and staying close to each other. They felt patients with similar illnesses to be put in one section (bay – which is supposed to be the case). Participants expressed that in some instances, patients that seemed to be having mental problems and were shouting throughout, were put close to patients with heart problems and needed some rest. In terms of congestion, HCWs indicated that they normally advise clinicians to quickly discharge patients that are better off to create space in the wards. | | *The only thing that I have observed is the place to sleep guardians. When the patient is not feeling well we tend to sleep in the ward with them, and the place is bad, and maybe if the government would provide a place for guardians somewhere else so that when the patient is feeling much better we should be going there.* |
|  |  |  | *No we cannot be sending patients away when the ward is full. What we do is to ask the clinicians to at least find the patients that are better and recovering well so that they are discharged and be given drugs to continue treatment at home just too create space for the new one, but we do not send the new patients back home.* |
| 1. **Non provision of and/or availability of worn out bedding materials** | Most participants complained about lack of beddings in the hospitals. Patients are expected bring their own bedding when coming to the hospital. Participants expressed that it becomes more challenging because they do not plan to get admitted in the hospital. When this happens, they either have to go back home and get beddings or sleep on mattresses that have plastic covers. Much as this is uncomfortable, it is can also a recipe for other illnesses. Because of lack of beddings, participants felt the beds are not suitable for patients, mostly from poor households. Patients are often either afraid or shy to ask for beddings from HCWs. Other patients justified the approach by the hospital saying that government cannot afford to buy certain items for the hospital. Very few patients mentioned of having been given beddings which were not changed till the day they were discharged. Others mentioned of old and torn mattresses and broken beds unfit for patients | | *This hospital used to have bed covers but now it’s only mattress covered with plastic covers, sometimes you don’t get ready to come to XXXX and when you are admitted you can’t just go home and get necessary things, so the patient will sleep on uncovered mattress which is not comfortable, there’s need to bring them back.* |
|  |  |  | *Another issue is that the beds are not covered and there are no blankets or bed sheets for patients to use so you have to bring them from home, which may not be possible for everyone. This is the same issue at XXXX hospital as well, I had to go back home to bring them (beddings).* |
|  |  |  | *All I know is that the beds are not covered, I don’t know if it is the problem of nurses or the hospital has no bed sheets, if you don’t bring bed sheets from home then you will sleep on the bed just like that. People who came Friday evening were given bed sheets and Saturday as well, we don’t know why but they stopped again. I think it’s easy to clean those bed sheets than to clean the mattress after a patient has gone.* |
|  |  |  | *On that indeed, the beddings needs to be changed, we have been here for a week and we haven’t seen them being changed, and if it’s changing, we do it alone, using our own piece of cloth, washing the, and dry them. The beddings that belong to the hospital we just remove them and use our piece of chitenje cloth.* |
|  |  |  | *No, when you ask them they say they don’t have, but I once saw them giving bedsheet, they looked new. Mmmm, giving them new bedsheet, which means maybe they have bedsheet, but they didn’t give them, I don’t know whether it was just those but at the moment everyone at the hospital uses their own beddings, they just give you bed and mattress.* |
|  |  |  | *I thought when they are giving a bed they should also give you a blanket or something to cover the patient at night but they don’t, you have to bring your own. There are other patients are just sleeping even without bed sheet which is not ideal for a patient*. |
|  |  |  | *The beddings were ours, they brought the hospital beddings the day inspectors were coming to visit the hospital* |
|  |  |  | *You are supposed to bring the beddings from home. On the first admission there was a patient who looked like came from the villages, she was covering herself with a piece of chitenje and the mattress was not covered, so I asked my mother if the hospital does not have beddings for people who come unprepared. I called the nurse to tell her that the patient was feeling cold and they brought one bed sheet for her, it wasn’t enough but at least she was covered.* |
| 1. **Ward and emergency areas were highly congested** | The hospital is highly congested especially in the wards. When it’s full, some patients end up sleeping on the floor and along the corridor, making the process of giving treatment awkward i.e. difficult to put IV fluids and do other examinations. It is worth noting that this only happens when the ward is full. There are instances when there are no patients on the floor or along the corridor. When patients are sleeping on the floor, it exerts further challenges to guardians as they now have nowhere to sleep. Other rooms with limited space include the resuscitation rooms which has a bed capacity of 4 patients but it sometimes goes up to 9 patients. Because of the congestion in the rooms, patients and guardians feared that there might be quick spreading of other diseases | | *Another thing is the number of patients in the ward is increasing every day, there’s congestion, and other patients are sleeping on the flow which makes their treatment a bit awkward. That should be considered, they may just extend the building to make it big and bring in more beds* |
|  |  |  | *XXXX ward where I was staying is like a waiting ward, most patients are admitted here if the nurses are not sure where to put them so it full all the time, you will find patients in corridors and on the floor which means the hospital is becoming small, there’s need to expand it.* |
|  |  |  | *We had challenges sleeping in the corridor because they would want to pass now and then with patients that were going to the theater, and we would also move now and then to give them space to move. We would fold the mattress for them to pass and that’s the challenge we faced unlike someone sleeping on a bed.* |
|  |  |  | *The challenges are, one, it’s a path and the patient is always bumped with people and must move now and then, and the patient doesn’t sleep properly because when people are receiving food and the corridor is always full of people. And sometimes the doctors come in late on their day, and people are always on queue in the corridor waiting.* |
|  |  |  | *The day we got here, we had no space because the ward was full so he had to sleep on the floor, and because he was feeling better he told me to go home just because it wasn’t hygiene wise it wasn’t ok, and I would also get infected. And I listened to him and I was coming in the morning, afternoon and leaving around 7 and when I get home around 9 I would call to check on him.* |
|  |  |  | *The resuscitation room is supposed to accommodate four patients, but sometimes you may find even nine patients in there, which means the extra patients are treated while still on trollies which makes it difficult to change position for doctors to work properly. This is the only room with oxygen so it is a forced move to have more patients than required.* |
|  |  |  | *No we cannot do that sending patients away when the ward is full), what we do is to ask the clinicians to at least find the patients that are better and recovering well so that they are discharged and be given drugs to continue treatment at home just too create space for the new one, but we do not send the new patients back home.* |
|  |  |  | *It was XXXX, they gave us a bed but it was full, more than full in fact, the beds were close to one another, if you tried to put your legs on the floor, you would be touching another bed. That ward receives different patients with different issues, you wouldn’t know what is wrong with your neighbor so it is much of a risk, and you can get new infections. The first time it was full yes but this time around was too much.* |
|  |  |  | *The second thing is space, of course we are a poor nation but the way people are packed there, you may get weaker and worse because patients are too close to one another, exchanging air from different patients. You may have one problem today, after two days you have another problem and so on. More space is needed.* |
| 1. **The hospital infrastructure is not supportive of providing effective care to sepsis patients and increased number of patients generally** | We explored how the hospital infrastructure is supportive or not supportive for care of patients with sepsis. Patients and guardians mentioned of specific ward (XXXX) that had nonfunctional electricity bulbs, broken windows (permitting mosquitoes to easily come in) lack of some fans in certain wards. Guardians also raised lack of conducive guardian shelters. Participants complained of inability of the HCWs to contain constant noise in ward XXXX (unlike ward XXXX). Participants also wished the infrastructure was designed in a way that patients with the different medical conditions were not mixed in the same ward to avoid further spread of infection among patients. HWCs wished the XXXX was expanded to accommodate more patients. | | *In ward XXXX, the whole room has one fluorescent (electricity bulb), the other one is broke for some time, windows are broken and mosquitos just come in freely.* |
|  |  |  | *Ward XXXX is better, XXXX is always full, I don’t know how the hospital decided that but XXXX is quiet and peaceful. People in XXXX are living like students at school, showing a level of discipline, not disturbing doctors when they are working, you won’t hear loud noise like in XXXX.* |
|  |  |  | *At the XXXX if could have more beds because we only 6 beds and it happens that we a patient that condition is worse but they can’t go to the XXXX because there is no space and we end up leaving them at the ward.* |
|  |  |  | *The infrastructure maybe if we had different wards for different medical conditions so that those with different condition like maybe Sepsis patient should not end up being mixed with other conditions to avoid getting other infections.* |
|  |  |  | *Guardians in XXXX don’t have a shelter for them to sleep when they are chased from the wards, so where they sleep to me I don’t feel comfortable, I wish they had a shelter. Of course they have it but it is not covered so during rainy season and this winter they could at least feel comfortable, they come to look after patients and they don’t have to end up patients as well.* |
| - 1. **Food diet provided to patients is viewed as sub standard** | We sought views of patients, guardians and HCWs over the food and diet that patients with sepsis receive when they are in the hospital. We realized that food is an important component of the care and treatment process in patients. We explored the common type of food patients receive, its preparation process and their general views towards the food. We noted that generally, that some patients depend of food from the home while others receive from the hospital. For most participants (including HCWs) they expressed poor preparation of the food in the hospital and provision of the same type of food over long periods of time. In some instances, patients resolve to buy some food recommended for specific patients, which is always challenging for patients from poor households. However, few respondents did not see any problem with the diet in the hospital | | *I remember in the past they used to provide chiponde and every Friday they were preparing rice. And they could occasionally give every patient necessities like sobo, soap, salt and matemba.* |
|  |  |  | *I saw the nsima that was prepared and I told her not to be receiving and we will be bringing for her. It was with beans, but not well cooked, and the nsima if you want to get better one you have to receive at the beginning, most times the nsima at the bottom is not nice.* |
|  |  |  | *Some patients that are in XXXX require 02 concentration so that they can be supplied with 02 therapy, and those patients with NGDs require feeding every 2 hours, and the food needs to be soft e.g. porridge, maheu, and milk. So for milk and maheu, most poor patients can’t or to buy eggs, and even porridge is not made of white maize flour that is not nutritious, so basically the nutrition status is very big challenge to the patients.* |
|  |  |  | *Maybe it is the planned diet because it was nsima and one egg, nsima and beans, one day it was nsima and chicken but the food was ok and porridge was better.* |
| 1. **Patients are provided with the same type of meals over a long period** | We learnt that the hospital commonly provide cabbage and beans to the patients in the afternoon and evening. One in a while they would provide eggs and chicken (which sometimes come as donation from well-wishers). Some patients expressed having stayed in the hospital for 2 weeks but were only served with an egg once. Most patients commended the porridge that is given in the morning as being nutritious and well soft. For patients that had been transferred from district hospitals, they indicated that the type of food was similar to that in district hospitals. However, fewer participants felt that the way certain things happen in the hospital is the norm because it has always been like that or it’s the norm in the village. For instance, some participants were ok with the food/ diet in the hospital. | | *The food that is provided at the hospital is not that good, nsima most of the times feels like it is not properly cooked and most days it is served with cabbage only and the way cabbage is prepared it’s like it was just boiled with water and salt. There are days they serve nsima with eggs, chicken and beans but most days its cabbage. This is hard especially for people coming from far, they depend on this food all the time unlike some people who are from around town their relatives can bring them different kind of food. Another issue is the amount of food that is given, it’s quite little that most of the times the guardian will not have any of it.* |
|  |  |  | *Nsima and beans today, nsima and cabbage tomorrow, those were the meals, in three weeks only once they gave us nsima and eggs. I was not impressed to say the truth; health workers are always preaching about health food but this was not an example of it.* |
|  |  |  | *Cabbage and beans and sometimes soya pieces that is donated by some Indians and it was once.* |
|  |  |  | *The time I started working things were much better than now but they changed and now it’s like they coming back to normal, sometimes if there is nsima and beans people do receive but nsima and cabbage some don’t, and other people receive beef, eggs and milk* |
|  |  |  | *Even the porridge that they get is too watery, yah, in the morning they are given porridge, too watery, not notorious that we can say that we say a patient needs something that can provide nutrition to his condition.* |
|  |  |  | *I’m talking about food that is beneficial to the body, porridge prepared in a way that a patient will be able to take it easily, likuni phala is not just any porridge, it is healthy, and it is good for patients and guardians too. Nsima and cabbage may some sound may sound harsh as people are used to eating meat but cabbage with nsinjiro is better for health than some of the fancy food, you might get fat with the other foods but that’s all about it.* |
| 1. **There is lack of nutritious food for patients in the hospital** | Both patients/ guardians and HCWs felt that the food was not nutritious enough to support the patient’s recovery process. This was based on repeated eating of the same kind of food with regard for balancing the recommended 6 groups of food. Participants expressed that poor nutrition would affect the ability of medication to work properly and quickly in the body. | | *If it’s beans that means it will be without cabbage, same thing if it’s cabbage that means no beans and that’s the biggest challenge because that means we are not eating 6 group of foods, and it could have been better if the food was cooked properly.* |
|  |  |  | *I remember one of the guardians said in the past they used to eat beef, eggs, and fish and well-nourished porridge, and when someone is hospitalized at XXXX nobody would bother to bring food but now we call people from home to bring food for us, and he talked a lot about nutrition part.* |
|  |  |  | *We all know nutrition plays a vital role in human being, so sometimes the food that the person is given is not appetizing. The medication cannot work, because the patient will eat a little and the medication will start working on the body, and because the patient is only relying on the sugars that being stored in the body, the condition of the patient will start deteriorating, because he’ll be weak with no food in the body to give him energy, for medication to work well in the body it requires energy, if nutrition of the patient is poor, recovering takes longer and also the stay in the hospital is prolonged.* |
|  |  |  | *Food that is given to patients here is not good at all, imagine giving a patient nsima with cabbage without any spice If a patient can refuse well prepared food imagine this meal.* |
|  |  |  | *The food that the hospital prepares for staff and patients, I can give it 30%. I have been to the kitchen and seen with my eyes the way they prepare cabbage. They just remove from the cold room, remove the outer leaves, put on a chopping board, then into the pot without cleaning. I have seen flies in beans and meat that is being served to patients. I don’t know how they keep/ store the food. The way the food is, there is not patient who can like it. Those that eat are extremely desperate and have no plan B.* |
| 1. **There is need for improvements in the processes of preparing food** | In addition to lack of nutritious food and repeated provision of the same type of food, participants were worried with the way the food is prepared. Among others, the mentioned Nsima that is not well cooked and cabbage that is not properly prepared. However, for patients that have no choice, they end up eating this type of food. Some participants also felt that the food is usually served cold (as result of time taken to move around the hospital to serve everyone) and the amount of food that is served is too little especially to carter for guardians as well. One participant said that they previously had a meeting with HCWs and requested that they should be given flour to prepare the nsima on their own. Since then, preparation of nsima improved. | | *At this hospital it’s mostly cabbage and beans, the flour is fine but sometimes they bring nsima that is no well-cooked so to take with cabbage or beans is not easy. Yesterday they prepared chicken and today they gave us eggs.* |
|  |  |  | *Aaa,I am sure healthy workers encourage people to be eating food that has been very cooked, but it’s better when the food is cooked properly and you put other spices but cooking without spices and give them to people, that’s why people don’t get the food when its cabbage.* |
|  |  |  | *And another it’s about food, we usually have nsima but the relish is a problem, its either beans or cabbage, if its beans people do eat but cabbage only few people receive the food because it’s properly prepared.* |
|  |  |  | *The food that is provided at the hospital is not that good, nsima most of the times feels like it is not properly cooked and most days it is served with cabbage only and the way cabbage is prepared it’s like it was just boiled with water and salt. There are days they serve nsima with eggs, chicken and beans but most days its cabbage. This is hard especially for people coming from far, they depend on this food all the time unlike some people who are from around town their relatives can bring them different kind of food. Another issue is the amount of food that is given, it’s quite little that most of the times the guardian will not have any of it.* |
|  |  |  | *It wasn’t cooked properly, and it happened that they had a meeting and those people apologized after they heard that maybe we should be given flour as guardians and we should be cooking alone and since then the nsima is cooked properly but relish is a problem, cabbage at times it seems like they cut using a panga knife then cook it.* |
|  |  |  | *The food that they cook sometimes it’s not prepared as if it will be eaten by people. If its cabbage day I think they don’t use cooking oil when cooking because when chewing you would see that it’s hard to chew and looks like its low and people nicknamed it java* |
| 1. **Hygiene and sanitation** | We explored participant’s views towards hygiene and sanitation in the hospital. Participants’ responses were mainly aligned towards approaches to cleaning, disposal of wastes and sharps, hygiene practices by patients, limited and non-functional toilets and bathrooms and materials and equipment for cleaning. Most participants understood the importance and need for maintaining high standards of sanitation in the hospital environment as a way of preventing further spread of infection which (which is one of the key prevention measures for sepsis infection). There were mixed views among participants as some felt that the hospital tries to maintain high sanitation standards by ensuring the premises is always cleaned (and that the blame is on patients whose practices compromise good hygiene in the hospital). Others felt the hospital could have improved in provision of functional sanitary equipment e.g. water and toilets. Participants also alluded to high number of people in the wards (congestion) that results in challenges to maintain good hygiene practices especially in the toilets | | *When you are going to the toilet from the ward, there is a water passage from the washing sink, this drain is so dirty, and a lot of garbage is there, it’s never cleaned*. |
|  |  |  | *Hygiene at the hospital is alright, it has people who take care of the place, who sees that when a patient is going to bathroom they call their guardians to help them and sometimes I would walk barefooted, because the bathrooms are clean.* |
|  |  |  | *The first problem at XXXX district hospital is sanitation and hygiene because there is no running water, especially the toilets, women use buckets to get water from a borehole to flash their stools in the toilets.* |
|  |  |  | *XXXX, Bay 2, they had a sewage blockage, and patients were complaining of bad smell coming from there, and my relative also complained.* |
|  |  |  | *How can you ensure sanitation in that environment, it’s not possible even if you mop every hour and the mopping thoroughly because there is floor base, even for you to put up a drip it becomes a nightmare, or you may put a drip but you need something to hang it, isn’t it? The base don’t have, it’s not there, so how do you ensure adequate care, drip and the like, people try as much as possible to make the ward clean but there are certain structural limitations that will not make that sanitation 100% ok, so the bed number, there is always so many patients, aaaaa, so that too our hospital, our hospital we allow guardians, isn’t it? to be in the ward.* |
|  |  |  | *Sanitation? as I said I have been here since 1999, I have seen changes happening, in the past we used to, when you entered XXXX you’ll be greeted by very fine smell, that is no longer happening, there are many reasons but I should think that the hospital has tried it’s best on infection prevention, that includes the sanitation in the wards, aaaa, but there are also still challenges, our hospital was meant for 1000 or 1200 patients, and it was built long time ago, now the disease burden has increased, but we are still using the same number of beds, so if you go to our medical wards you’ll find that, we have floor beds, even other patients in the corridor.* |
|  |  |  | *The bottom, so it’s really difficult to ensure sanitation, three if you go to the sink, they are continuously running and the flow is wet, that also difficulty to ensure good sanitation in the ward, aaaa, so I can mention all sorts of things, that much as we want our sanitation to be up there, but we are failing because of a number things.* |
|  |  |  | *Hygiene was good, sometimes it is those people in ward who do not cooperate with the cleaners, if they say move your belongings we want to clean some people do not cooperate so they just clean space which is available to clean. My biggest concern was the toilets but no they are well maintained even though there is water problem sometimes, there was this one day a patient relieved himself in the bathroom because there was no water in the toilet, we all condemned that behavior. So they are trying their best using cleaning chemicals, cleaning windows and the ward is mopped three times a day.* |
|  |  |  | *Yes they do clean but the issue is water, when water is not running in the taps it becomes a big challenge, even the first time I came, there was no water for a long period than this time. There are no alternate toilets to use when there’s no water, patients are forced to keep using the toilets.* |
|  |  |  | *In terms of hygiene I think they are trying, in each ward there is a chairman who makes sure the ward is always clean, when you go to the bathrooms you will find them clean and the toilets too, the only problem is that both men and women use the same bathrooms and toilets, I would like them to specify one for women and the other for men.* |
| 1. **Lack of detailed approaches to cleaning of the hospital environment** | Participants (both patients/ guardians and HCWs) agreed that the hospital wards are cleaned but most of them were not impressed with the way cleaning was done. They raised that cleaning is done in a hurry, there is no proper and detailed mopping. Some cleaners would not clean if guardians hesitate to go out of the wards. HCWs felt that ever since the hospital subcontracted cleaning services to external companies, levels of cleaning in the hospital had gone down. | | *Cleaners also are moping the floor in haste, they only mop in spaces between beds, and they don’t clean under them.* |
|  |  |  | *They do clean in the ward, though they tend give up quite often when the guardians delay to clear the room. It’s not an easy thing for the guardians to be leaving their patients alone but that’s what is required, so sometimes the cleaners will just leave if the guardians are reluctant to go out. This normally happens in the evening and sometimes there’s only one cleaner available..* |
|  |  |  | *For the two weeks I have been there, the mopping is not done properly. They don’t mop under the bed, they don’t move the things we do it alone, but there is someone who is doing his job perfectly, but some they don’t mop well. Sometimes they say all guardians should go outside, and if they see that we are hesitating, they don’t mop. But in the hospital we have people from different places and you wouldn’t say you won’t mop because the guardians have not gone outside. They have to force the people out, we see some wards with clean sanitation and we don’t know if it’s the same people doing that.* |
|  |  |  | *The guardian who has stayed longer than others would be the chairman, responsible for the toilets, if they are not clean he would ask all the guardians to go and clean but they just use water no chemicals.* |
|  |  |  | *if I compare from when I was a student and now, I think the wards are dirtier, this is my opinion, from the time they switched from having permanent staff to the contracted ones, I think they became dirtier, I have a lot of time when I am doing a ward round and the cleaner comes to clean, the way they clean if it was my house I wouldn’t clean like that. They put the mop in the pail and mop a long distance before they go back to the pail, so basically what they are doing is just putting water there not cleaning. So in the emergency department a patient can vomit, and the vomit can stay there for 2 hours without someone cleaning until I go tell them to go and clean it.* |
|  |  |  | *The mattresses are supposed to have a plastic cover but quite a number of them are torn so the flex foam absorbs all that. Just on my last call I sat on a mattress that looked clean I saw fluid coming from the flex foam and I felt wet and I was sure it was urine, I had to go home change. Another thing about infection prevention for example cannulas are supposed to be in three days’ maximum but people don’t change them unless the patient is messed up then the nurses will follow that through. And I have seen a patient die, they remove all katundu and they put another patient and the bed is not even cleaned.* |
|  |  |  | *There is a lot of luggage that patients and their guardians would bring in, making even the cleaning process difficult, so every day we have to say please take out your stuff, leave it on the shelter, let the people clean alongside the beds, head side, but when there is a lot of luggage they cannot move.* |
| 1. **Poor disposal of wastes and sharps** | Participants raised several examples over concerns of how patients and their guardians and HCWs disposes wastes and sharps. Such examples included littering of left overs of food in sinks and surrounding areas. Cleaners complained how nurses would dispose used needles in waste bins than sharp boxes. Participants realized that such practices could lead to infections | | *Another area which is of concern in the place where guardians rest just outside the wards. They litter anywhere including throwing left over from nsima right at the sink where they get drinking water. The place is always smelling with water jamming and becoming stationery. There is a person who cleans the area and is always complaining to the guardians.* |
|  |  |  | *The gloves that we use when cleaning are not heavy duty but are the surgical ones. They are easily torn when pressing the mop. So even when mopping a place where there was blood or vomits, it could get torn and even have the water sprinkle into your eyes. I have also once seen a nurse putting an already used needle at the top of a waste bin. I didn’t say anything. I just removed it but I asked myself what she was thinking. A needle could easily get trapped in a mop. I have previously been pricked by a used needle.* |
|  |  |  | *It’s very dangerous because we are not sure what the patient they used a needle on is suffering from. Even to us the healthy workers the way we have our waste it’s a challenge, they would see a bin is full but they would still throw and let it overflow, the management is poor.* |
| 1. **Soiling of toilets and bathrooms by patients and guardians** | This is a concern that was raised by many respondents. Despite efforts by the hospital to clean the hospital premises and ensure sanitation facilities are in place, most people blamed patients and guardians for practices that compromised sanitation and hygiene in the hospital. They pointed out that although cleaners are always cleaning the wards now and again, patients and guardians frequently makes the toilets messy. They often find waste material or urine anywhere even right at the door to the toilet  This on the other hand raises what strategies are in place for HCW engagement with patients and guardians on hygiene practices in the hospital and monitoring of how such strategies are working if they exist. | | *Sanitation is very hard to practice it because we are people from different districts, and we leave in different houses but some do take of their homes, some don’t and it’s hard to give each other directions on it, for example in bathrooms it might happen that it was cleaned and we are the same people who go there and makes it dirty again.* |
|  |  |  | *The hospital cleaners are trying their best to clean the environment but we guardians are the ones making this place dirt, when we take a patient to the toilet we don’t clean after, washing dishes where we are supposed to do our laundry, so it’s the guardians who don’t show seriousness about sanitation.* |
|  |  |  | *As a guardian even the nurses needs help and we are supposed to show them that we can also do better, there are people who clean the bathrooms and toilets, but you would see someone going there and spoil the place because we live at different households and our behaviors are different but if we could be monitoring each other that would be good because if we don’t we could easily get infections.* |
|  |  |  | *And throwing them in the toilet, and also how to use bins, the time that someone has finished using the grooves (guardians), they just throw them anywhere, because they don’t know how to use those things, and that’s why the duties of a chairman comes in just that the chairman is not active* |
|  |  |  | *Yes, the chairman are there but I also think they have problems, but some can get fixed, what is missing is that the chairman should talk to people like for example, someone has come from the village and they don’t know how to use a toilet, and they don’t know how to use the grooves, in such a case in the evening when people have settled the chairman comes and talk to people and train them not just sitting in the hospital and watching.* |
|  |  |  | *Despite hearing people saying medical wards are now looking clean, but still they are not, the main contributing factor is the congestion of the wards and the way people uses either their waste products and whatsoever, like they have eaten something, they would throw actually in the wards, disregarding that they have a bin outside, and they can go out and throw, they don’t , they throw actually in the wards,* |
|  |  |  | *Despite that sometimes is the use of toilet and bathrooms, some they soil that deliberately ,I remember last week I had to call all the guardians and we went there and we had a meeting, because there is no way you can leave the toilet and defecate in the bathroom, what do you expect people to do? Just because they have seen that there are cleaners, they would say iiiiiiih a ntchito a boma akolopa (government workers will clean). On the sanitation part it really a challenge* |
|  |  |  | *Chambers that patients use to relieve themselves are mostly not cleaned by the previous user, so the guardians are forced to clean them before they bring them to their patient, everyone is supposed to clean after using it.* |
|  |  |  | *Both bathrooms and toilets are lacking hygiene. Yes, because some people might have running stomach but end up using the using the toile without cleaning with water, and it might happen someone steps on that.* |
|  |  |  | *Yes, people would just defect anywhere near the toilet, and some even that area used for washing, and it was sad because we are already sick and it might happen that we are coming back to the hospital* |
|  |  |  | *Yes, they cleaned the ward. The problem was because of the occupants of the wards. For example, people brought in a plastic that clogged the toilets and that was not a problem created by the cleaner but the people that were using the toilets.* |
| 1. **Limited and non-functional sanitary facilities i.e. toilets, bathrooms and sinks** | Participants complained that one of the factors that affected the hospitals ability to maintained high standards of sanitation and hygiene was the existence of non-functional sanitary equipment and the hospitals failure to maintain them. Such problems included: broken/blocked water basins/sinks and toilets, and inconsistent supply of water. Participants recommended quick maintenance of broken toilets and provisional of additions toilet and bathrooms to match the increasing number of people in the hospital. They also recommended that men and women to use different bathrooms and toilets. | | *The sinks in the ward are broken, like the ward I was in there was only one sink which was working and the whole ward will be lining up there to get some water* |
|  |  |  | *Toilets and bathrooms are few, if you consider the number of beds in XXXX, they are sixty something beds. You go there you have to wait for someone to come out, the same thing happens with bathrooms.* |
|  |  |  | *Let me talk about the bathrooms, the showers, the flashers are broken, you need to bring your own bucket for bathing and there are small containers that we use to carry water into the toilet to clean once you use it, these containers are not cleaned, looks very dirty. And the toilets are few, people have to wait on one another to use them and there are no different rooms for women and men, they all use the same rooms.* |
|  |  |  | *The hospital has water problems as well, the whole ward has only one sink which is working, everyone is waiting on the same sink, the bathrooms and toilets have same problem, and water is not consistent so with that crowd for everyone to take a bath is not easy.* |
|  |  |  | *We had an issue here last week, two of the four toilets we use blocked so people were using two only, this was not ideal as there are so many people in this ward, we had to be waiting in turn to use the toilet, Saturday I saw a woman who could not hold on to her urine go behind the ward helped herself there, after seeing this I didn’t like it so I went the office to report about it, they asked if I was the chairman and I said no, I’m just a concerned member, they promised to get it fixed and they did.* |
|  |  |  | *Right now only one bathroom is being used, the other two have blockage for three weeks now according to the people I found there, they are not fixed until today, it comes to a point you just go to the toilet room use it as a bathroom.* |
|  |  |  | *There’s no hygiene, taps in the toilets are broken, you have to get water from a pipe that leaks water to wash your hands the use the same water to flash the toilet. The bathroom door is broken, you have to move it to close when you want to take a shower* |
|  |  |  | *The mop inside nicely, but we had a broken toilet somewhere behind, and it was smelling badly the whole time we were there.* |
|  |  |  | *It was good to see people being treated but there was a problem, a sink broke and it was leaking water, there was water everywhere. I could not sleep because I slept on the floor so it was no possible to sleep with water running all over.* |
| 1. **Lack of cleaning materials i.e. heavy duty gloves** | This was an issue mainly raised by HCWs. Hospital attendants complained of non-heavy duty gloves which gets torn when pressing the mop. Initially they had cleaning equipment that could press the mop without touching it. There is no consistency in the supply of chemicals used for cleaning. | | *We have challenges like unavailability of glove and toiletries, we are buying these things ourselves.* |
|  |  |  | *They do get teared off, back in the day we used to remove water from the mop with our hands, we could use a bucket that had its own place to remove water from the mop, right now we even touch the mop when it has blood, vomits, and the bins they are just thrown anywhere and the needles too which makes us hard to work and sometimes people throw trash while you are mopping and we do talk to them at times but sometimes not to avoiding noise and we just end u collecting the trash.* |
|  |  |  | *Chemicals for cleaning are not readily available. Department of medicine we get 1 litre which we share with ward XXXX, XXXX, 2A and 2B and due so many places that needs to be cleaned this isn’t enough.* |
|  |  |  | *Bathrooms hygiene is alright only that we are lacking chemicals that use for cleaning, and these we don’t usually have them and stains form* |
| 1. **Limited staff** | We considered adequacy of HCWs to effectively manage the number of patients in the hospital as an important factor to the care of patients with sepsis. We explored from participants their views over the number of HCWs in the hospital. The general perception (especially among HCWs) was of limited number of HCW. Their responses reflected; limited clinicians’ time with patients and patients being missed in the care process, as resulting from limited staff in the hospital. Burn out was one of the effects and HCWs recommended changes in the work shifts. They also felt that staff are overwhelmed because of lack of community understanding that the hospital is a referral centre. In several instances, patients come straight to the hospital with conditions that could be addressed in community clinics. | | *The EMERGENCY is one of the busiest departments. I would wish that nurses from XXXX work maybe from 7:30am and knock off at 12noon, and others should come at 12:30 and knock off at 4pm. In so doing, they could provide the best care ever, because it’s the busiest department, one. Otherwise they work for 8 hours just the same as those in the ward which is a bit tiresome and they can’t provide the best care* |
|  |  |  | *Challenge that I see here is that its referral hospital from health centers or other district hospitals and that their condition they cannot manage their due to lack of resources including doctors. But people don’t understand. They could come to the triage straight from home complaining about stomachache. That’s an issue we experience every day. And yet we are already few in numbers.* |
|  |  |  | *Yes, but it depends on what complaints have they presented, some may rightly come straight here without referral note we manage them, some patients you can even just see how they are, you don’t need to ask them you just manage them but those with minor complaints are told to go to a nearest health center in their area.* |
| 1. **Clinicians have limited time with patients** | Both doctors and nurses alluded to the fact that due to high numbers of patients in the hospital and associated shortage of HCWs, patients are not monitoring as often as they should. This is particularly challenging for sepsis patients that have high fever as well as timely requires monitoring of fluids. | | *It’s because of understaffing, like maybe 6 patients against 1 doctor despite the hard work of the doctor maybe monitoring the patients 3 times on a day but still it’s not well practiced.* |
|  |  |  | *Like most times Sepsis patients have fever and if we don’t treat them in good time it leads to other problems. Mmmhu, because you are not monitoring the patient now and then, like maybe urine output is low, which means fluid output and fluid input it’s not balancing, and if you are not following the urine output, it’s hard to know the know other problems but if you are monitoring the patient you are able to track other problems.* |
|  |  |  | *Currently like I said we six nurses on duty during ward round, five nurses during normal days against sixty patients and tough to listen to every patient and as a result patients are left un attended. During the night, there are three nurses against sixty patients. I would wish the government could recruit more nurses so that the ratio is reduced.* |
|  |  |  | *I have received complaints especially from guardians, lodged a complaint that his patient was supposed to be checked blood sugar, it was a two hourly random check but due to the issue of understaff the nurses could not manage, so he came to me and said this nurse did not properly manage my patient. I explained to him that the nurses are few and it will be difficult for them to manage two hourly checks* |
|  |  |  | *I think the lack of nurses, if it is in the day and there’s shortage of nurses’, patients don’t get seen as frequently, so patients who are getting worse are seen frequently but identifying them in the first place is the problem, but if they are identified, they are usually followed up by both clinicians and nurses*. |
| 1. **Shortage of nurses** | All participants felt that HCWs were in short supply in the hospital. Patient/nurse ratio was extremely high that the recommended resulting in limited monitoring of patients. The situation is the same both in the emergency department and in the wards. In turn, HCWs pay much attention to patients who are very sick, losing out on other patients. HCWs recognized that this has been a long standing issue and they don’t expect to be addressed soon. Despite the challenge, HCWs expressed that they try their best to provide care to all patients as needed. Doctors also expressed that they are too few in numbers. | | *In terms of treatment I think there are few health workers who work at night, we arrived there after 5pm at the emergency department but we stayed there until late in the night, it takes time to help all patients* |
|  |  |  | *Normally it’s supposed to be 1:1 ratio, 1 patient, 1 nurse, but because we are very few we were told 1 nurse to 5/6 patients, and you can give holistic care, but because we are very few its 1 nurse to 15/20 patients per day, of which you cannot carry out the orders or maybe you might carry out all the orders but at the end you can’t have enough time to rest and refresh to go back and start helping the patients again.* |
|  |  |  | *Sometimes maybe someone might be alone on duty and when knocking off he/she might have left some work and you have to go through the file to check what was done and what was not done so that you should complete unfinished work.* |
|  |  |  | *In the EMERGENCY at the priority section, you would find that it’s full and yet the nurses are very few. Sometimes you could find that all 14 beds are full yet there are only 2 nurses. When we do the allocations, 2 nurses would be in the cubicles, 2 would be at the triage, 1 in the trauma and another in gynae. When it’s very full, you wouldn’t find anyone just sitting down.* |
|  |  |  | *there are few of us on the ground, too few, for example a day like today I have to do a ward round in the morning and I have to go to the clinic in the afternoon and at lunch time I have to pick up my kids, so what happens is that I come early maybe 7 am see a few patients, go to the meeting, do the ward round and go to the clinic but a lot of times like for me on Wednesdays after clinic I still need to go back to the ward and I leave at around 6:30 to do everything, to make sure everything is done but if you have a very tight schedule that day its difficult but everyone needs to be seen. That the difference between us and nurses, so a nurse, if its 12noon they will certainly go for lunch, if its 4:30 they will make hand overs. I can’t do that, it’s 4:30 and I have 4 patients that I have not seen, I still have to see them even if it means knocking off at 7pm, so to make sure you should knock off at 5pm instead of 7pm, which is rare for me, you have to be fast and that limits the engagement.* |
|  |  |  | *The XXXX the only issue I have with the XXXX is the workload, because when you are on XXXX call you also do consultations so the entire hospital all medical patients will be looking for you, your day start at 6:30 in the morning and ends at 7:30 in the evening and the cycle starts again in the morning for straight seven days, and then these patients needs to be seen frequently so marrying these is a bit of a big deal, sometimes you have to go to the clinic as well but I have to say that because there is more staff in XXXX so there is you who is supposed to see these patients, there are also nurses who are stationed there so things are a bit quickly and usually they put someone who is more experienced. And there is a lot of senior support in XXXX because the consultant is readily available for you to discuss what you should do with the patient. But the funny is sepsis patients rarely get admitted to XXXX and they are rarely admitted to ICU.* |
| 1. **Shortage of equipment** | Equipment for medical procedures was one factor that participants raised as being essential in the care of patients with sepsis. Generally HCWs raised shortage of equipment in the hospital. We explored effects of shortage of equipment on medical procedures and examples of equipment that are in short supply. We learnt that shortage of equipment was as a result of theft, non-maintenance of broken equipment and equipment simply not being available in the hospital. However, HCWs indicated that in the midst of the fewer resources, the improvise to ensure that patients get the required care | | *When we arrived two doctors came with three thermometers, they tried to read his temperature with the first one, it wasn’t working, the second one the same and the third. The patient looked very sick so according to them the temperature should be high not low. They asked if we had given him any medication from Sochi, we said yes but the other doctor said it could not be the drugs. They brought another thermometer which worked properly.* |
|  |  |  | *We also have few material resources but we do improvise to make sure that our patients should get the best treatment ever according to what we learnt so we do improvise and apply the knowledge to our patients* |
|  |  |  | *Most of the equipment’s here are not durable, maybe they have limits to how many patients can use it, for example may unknowingly use a faulty BP machine and give wrong readings to a patient.* |
|  |  |  | *Our XXXX is not to the level of XXXX which is supposed to be, because it’s like a bed and oxygen concentrator, there is no monitor, we use the very same blood pressure machines like the Digital ones that use, we don’t have something that we would say this is the way the patient is looking just looking the monitor continuously checking on the vital signs on that particular patient* |
|  |  |  | *You leave it there and you come back its not there, so these things go missing all the time. The only thing that doesn’t go missing is stethoscope because it is always around my neck* |
| 1. **Shortage of equipment affect clinical procedures** | We explored the effects of shortage of equipment on clinical procedures and the care of patients with sepsis. Respondents raised several challenges including: giving of oxygen to patients using dry concentrators because there are no humidified concentrators; provision of oxygen to patients using concentrators instead of cylinders because there are no gauges; watching a patient die since guardians cannot manage to buy NGDs; sepsis patients failing to go for x-ray or tested for blood samples because machines are not functioning; unable to carry some procedures i.e. BP measurements due to lack of forms for recording; unable to get timely medication and death. | | *Other materials like catheters are also a problem here. We come to an extent of telling a patient to buy. Some patients do buy but others are unable to do so.* |
|  |  |  | *We do not have concentrators that give humidified oxygen, they are not working and we choose to give patients oxygen using dry concentrators and it becomes a challenge and a burden, and you tend to think what if it was me. If they could repair the concentrators and also buy other gauges it could really help.* |
|  |  |  | *Another challenge is about cylinders, we only have two gauges which we share to patients, and you’ll find out that we have we have a patient that needs oxygen from the cylinder but you would find out that we don’t have gauges and we are forced to use concentrators* |
|  |  |  | *NGDs sometimes we don’t have and Sepsis patients at some point they reach a level that they can’t eat and we need gastro tubes and we tell the guardians to go buy but if they can’t manage we end watching a patient die and we are also out of stock* |
|  |  |  | *It is big problem, for instance, there was a patient with signs of BP, and we could not confirm this because stationery was not available to help him so I asked the patient to see me again in the morning but he never showed. It’s quite difficult to assist a patient without these forms, every test is supposed to have a form, in case a clerk forgot to indicate the type of test for patient on his or her bottle, the one in the lab will be able to know through the forms.* |
|  |  |  | *We also have a problem with papers used to print or copy various forms, it’s a big challenge too because each test a patient is going through is supposed to have a form, be it BP, urine test, FBC and so on, sometimes we have to photocopy them in room 5.* |
|  |  |  | *I think maybe just to add on the lab part if there machines are not working well, we want to process samples maybe because sometimes we are told that we are not taking ABC because the machine is not functioning, we are not doing this sample because the machine is not functioning, or sometimes the x ray is not functioning maybe we needed this patient with the sepsis to go for x-ray maybe for a particular diagnosis.* |
|  |  |  | *On the part of management, yes, they will say I think we failed them, it’s like maybe the care that we gave, it wasn’t enough, the patient was not supposed to die maybe because we did not do abcd, sometimes it will happen that we wanted to commence on oxygen but we don’t have because all the oxygen concentrators are with patients so we would say there no way I can we remove from this particular patient and connect on this patient, then on ethics part am doing harm, I would just explain that we were supposed to do abcd but we don’t have, had it been that we had we could have done abcd, they will come in and say but sometimes we say maybe because of the grieving part they can’t understand why this has happened, and sometimes we tell them that if they feel that they haven’t been treated well they can take the issue further maybe, as long as the moment I was explaining I documented in there file, the patient need oxygen but we did not have.* |
|  |  |  | *We need the blood gas machine in the emergency department, we need blood gas machines in the XXXX and in the wards, like for me one of the things that I hate doing but I have to do it and I end up doing it is being on call the night and there is a patient who is very sick, who you think has acidosis, you know is very sick yeah and he needs blood gases immediately and I have to walk in the night all the way to XXXX and convince someone there that they should share me either the syringe or the bottle of the blood gas then I have to call their clinician on call there and convince them that their nurses should let me use there machine, it’s quite a process that takes close to an hour but you have to get it done. So the commute from the emergency department to XXXX back to the emergency department, to the point that at one time when I went there I said just give me a few syringes to keep in my back. So in I deal emergency department you are supposed to have that machine so that you can access it every time. And then I think if we can have vital machines that are fixed to the wall so that they don’t grow legs (stolen).* |
|  |  |  | *On the drip issue, the hangers at XXXX it seems they have few, the day that I went, I had done two drip at the reception, while I was going to the ward I had drip which was about to get done and it took them sometime to hang it again because of lacking those hangers, they searched and found one that someone was using and they hanged it there, and it happened that the next day I didn’t have drip because of lacking the hanger. Around 4 that’s when they said I needed a drip of water, and they searched for a hanger they couldn’t find it, then and the day passed, and in the evening that’s when they had found one.* |
| 1. **Thermometers, BP machines, Pulse oximeters, blood gas machines, sanctioning machines, oxygen concentrators, Y connectors, Central venous pressure, and catheters. In short supply.** | This sections lists some of the common equipment that are either in short supply of unavailable in the hospital and compromise the care that is given to patients with sepsis: drip hangers, O2 concentrators, gauges for oxygen cylinders, Catheter bags, NGDs, BP cuff, thermometers, tunicates, breaking down of computers for hospital clerks affecting recording and retrieval of triage data, pulse oximeter, PPE for hospital cleaners i.e. glasses and heavy duty gloves and sanctioning machines  HCWs noted that for certain equipment i.e. thermometers, they usually stop functioning so quickly once they are bough. They are not sure if they type of the thermometers bought is not durable (it might also be as a result of being over used on many patients). Equipment like BP machine is often shared on three station in the emergency department. HCWs normally report these problems to the Matron who takes them up with Management. Unfortunately little or no change is observed. | | *There are few drip hangers, if your patient is on drip and he happens to go to the toilet they will take it and give it to another patient, it will be appropriate if each bed will have its own hanger.* |
|  |  |  | *The other challenge is 02 concentrator, it doesn’t provide 100% oxygen, and some give oxygen but not humidified one which the uptake is easier than the dry one and provide the better and well needed oxygen to the body, the dry one gives challenges because you to be feeding the patient with fluids* |
|  |  |  | *They are there, but maybe they are not functioning, and the best cylinders because you know the right concentrated oxygen but the other challenge is the connector itself called gauge, you might have a gauge that is not working, yet the cylinder one is the one that provides better the oxygen needed in the body.* |
|  |  |  | *We can’t check for vital signs without equipment. In the ward you’ll find that we only have one BP cuff and maybe 2 working thermometers to cover 80 patients, and we find that we are only checking vital signs in the morning due to lack of equipment* |
|  |  |  | *Instead of having tunicates we do use gloves, instead of screens we do use stingy raps. In XXXX the gauges we have are supposed to have some rubbers so that air should not leak so we improvise by having rubbers from syringes. Transportation of patients, instead of having proper trailers we sometimes use beds which do not have proper wheels.* |
|  |  |  | *We don’t have enough concentrators, there’s only one working at the moment. So you can imagine if ten out of 60 patients are having respiratory problems it’s a big problem, we don’t want them to die but in this situation death will probably occur because those three would be in use already and another patient is desaturating. A normal person is supposed to saturate at 90 to 100%, if it reads 89 and below it means that patient needs oxygen therapy, we would try to get extra from other wards but to no avail so the patient dies.* |
|  |  |  | *Like for the past six months until now we don’t have Orgiastic tubes that are used to feed patients, we don’t have catheters, NGD,* |
|  |  |  | *At first when dusting we used to put on glass to protect our eyes from dust and we had very strong gloves but now we no longer have those. We just wear the small plastic gloves* |
|  |  |  | *During rainy season our computers have network problems, we are forced to write patient information on a paper, if you lose that paper it means patient information is lost as well. If we were provided with electronic tablets to use when the computer network is down that will be helpful to at least send the data to HIMS.* |
|  |  |  | *There’s x-ray department, in the process of diagnosing a patient, x-ray machines are needed. But we have only one which is working and it has a limited number of patients it can handle in a day, this delays treatment too because when it reaches its maximum number other patients will wait till next day.* |
|  |  |  | *I think on the Sepsis, unless that patient with Sepsis requires oxygen, or requires sanctioning then I think the equipment that I would say we don’t have or we are not able to supply ABC because the machine is not working, then its either the oxygen concentrator that maybe we are run short of because the patients that will require that maybe more. And maybe the sanctioning machine if this patient let’s say has meningitis, with levels of conscience is very low, needs to be sanctioned, I think I would say these.* |
|  |  |  | *they would receive a call that we are sending a patient in the XXXX sometimes there is not space in the XXXX, we are supposed to tell them there is no space, or maybe if the patient comes will be on bay one because XXXX is full or we will have space but we will not have oxygen so if the patient needs oxygen likely will suffer, there is no oxygen, sometimes we do the sharing with the Y connector part, say one concentrator can give two patients, but we don’t have that Y connector so we would say , we have the concentrator it’s one and it’s in use, unless if we had Y connector, but if you have bring that patient with the patient, when we are done with patient we will send it back but sometimes it doesn’t work that way* |
|  |  |  | *thermometers, BP machines, so one of the irritating things that going around looking for a BP machine, sometimes it’s just one for the whole place, there were times when there was only one pulse oximeter, as doctors, that is not an excuse we are supposed to have our own personal items, I have been here for four years and I have lost the entire bag three times so right now I don’t have.* |
|  |  |  | *The XXXX does very well, the only complaint is that they don’t have the blood gases, they don’t have central Venus pressure, so they don’t have some equipment that is supposed to be there. They do have ulterior blood pressure monitoring but not always, so patients with sepsis need those things as well. Even if there is shortage critical drug in the pharmacy, if the patient is in critical condition we try to get it, so patients in the ICU I think are taken care of very well and the nurses are good and as a doctor stationed in ICU you stay there, you are not allowed to go to the clinic, you don’t go on call, you do teaching but it’s rare.* |
|  |  |  | *On the drip issue, the hangers at 3 B it seems they have few, the day that I went, I had done two drip at the reception, while I was going to the ward I had drip which was about to get done and it took them sometime to hang it again because of lacking those hangers, they searched and found one that someone was using and they hanged it there, and it happened that the next day I didn’t have drip because of lacking the hanger. Around 4 that’s when they said I needed a drip of water, and they searched for a hanger they couldn’t find it, then and the day passed, and in the evening that when they had found one and those are the problems we are facing because we wouldn’t just carry it in our hands.* |
|  |  |  | *Though I was not aware of anything the time I was moved to XXXX, I woke up with oxygen tubes connected to me but I realized that the cylinders do not work properly, sometimes they don’t bring oxygen and the nurses rely on patients to tell them if oxygen is coming or not and I find that unpractical. They have to look into that and get them fixed.* |
| 1. **Shortage of medicine** | Availability of medicine in the hospital had mixed views and experience among patients and guardians. For most participants, shortage of medicine was not experienced during their stay in the hospital. There were fewer respondents who were told to buy medicine or seek for further paying care from private hospitals. However, in certain instances, some drugs are not available in the pharmacy and patients are told to buy. HCWs pointed out types of drug that were is constant shortage in the hospital. In this section we explore factors affecting supply of the medicine, effects on non-availability of medicine and situations where patients where referred to private care. | | *Normally in XXXX sometimes you see the patient was supposed to get transfusion maybe there is results is 2 gram the patient needs urgent blood but they haven’t done that they haven’t followed up. So sometimes is the attitude of that particular healthy worker who was attending to this patient but sometimes it’s the lack of the resources that were supposed to be used for the example the blood say the blood bank doesn’t have the blood so they haven’t been given or on the drugs they will say the EMERGENCY has run out of the drug and the pharmacy during the night doesn’t open so they would wait until in the morning.* |
|  |  |  | *Assuming this patient require Iv drug, there is no fluids if it will be a drug that needs to be dissolved and go through the system, and the other thing like fragile IV, we normally get the drug upon the file or using the prescription so we will say we have 6 patients who are on fragile IV, each patient has to receive 8 hourly that’s 3 times a day,, so in 24 hourly its likely to be 6 doses, there supposed to have it continuously, if you do not have likely the other dose the patient will miss.* |
|  |  |  | *The hospital should improve on how they are treating patients, I am saying this because I believe we receive proper care this time because we were selected to take part in this study and we are very thankful for that, there was no single day they told us they have no medicine but another patient on a bed next to ours with meningitis as well was told there was no medicine so they needed to buy, the same medicine my patient was given. They had to go right away to buy the drugs that cost 14000 something, this was supposed to happen for 7 days, just imagine.* |
|  |  |  | *Sometimes you would find that we don’t have shortage of drugs, and at times you would find that we don’t have drugs, you would find that a Sepsis patient who’s taking Amoxil on a day, in the evening you would find we are out of stock because we also order drugs per patient file which means we get drugs on order of file and patient share depending on stock available.* |
|  |  |  | *They scanned my stomach and they found that I had some swelling in the kidneys, by the time I was being discharged, they tested my blood and they found out that I had low blood but they did not gave me any blood transfusion just the pills. The day they discharged me, they told me to get the pills at the pharmacy, and at the pharmacy they gave me the pills for blood only, and they did not have the other medicine in stores.* |
|  |  |  | *From the day that I arrived, I never came across such problem, I even went to get Bactrim at the pharmacy.* |
|  |  |  | *Not always short in supply, but we might have IUV drugs like Tresue are in store, and when the patient has been switched to oral drugs like tetraxon to efromaxin, you’ll find that in our pharmacy we don’t have.* |
|  |  |  | *Regardless of ordering for materials from our pharmacies, sometimes they tell us that they don’t have these resources so for us to care for our patients it is a challenge, so we ask the patients to buy which is not normal, some patients are poor they cannot buy them, they suffer or even die because they could not manage to buy them.* |
| 1. **There is shortage of antibiotics, meropenem and dry blood banks** | In this section, we present examples of drugs/ medicine that HCWs reported that they are in constant supply in the hospital and patients/guardians were not able to access at a particular time in the hospital. These medicines included: Amoxil and Paracetamol. Sometimes the delay is from central medical stores. When HCWs order from the pharmacy, they are normally told that they have ordered from CMS. We learnt that medicine are ordered as replacements, and that certain medicines like Meropenem are expensive | | *Drugs like amoxlyne which is a very important drug we don’t have, paracetamol is now available but we didn’t have it for the past four months* |
|  |  |  | *They try their best to help but sometimes the issues go beyond their control so they are forced to wait as well, last month we didn’t have Panadol up to the point that we could ask patients to buy.* |
|  |  |  | *The big problem is shortage of drugs for example Sepsis patient is supposed to receive antibiotics and most of time we are of stock like as of now we don’t Amoxil, and most patient they buy alone.* |
|  |  |  | *Let me start with what makes that care sometimes not to be as expected - sometimes the patient with sepsis will be on antibiotics, but sometimes the flow of giving that antibiotic or the duration is not met, because maybe the drugs are not available. I remember I think there was a patients that after blood culture was supposed to be given chlorofenico but we did not have the chlorofenico even the pharmacy did not have so on such things it’s more likely that will require the patient or the guardian to provide thereby it will also depend on the finances if they are able to buy that drug* |
|  |  |  | *the last thing that I have also noted is antibiotics, for organism that are easy like cop pneumonia, typhoid, we readily have the antibiotics but there instances that you get blood culture results, which says this patient has got ESBL, it’s a multi drug resistance organism, the only antibiotics that you can give is possibly cabapenilamu, like melepenimo, ulepenimo or amicasin, we don’t have those drugs so it becomes a challenge to the attending physician to say what am I going to do, it’s a poor patient who cannot buy ….even you attending clinician you are even poor you cannot buy for the patient.* |
|  |  |  | *No, they did not. They just gave me dosaicleen, and after given the medicine I went to the healthy center and they gave me buffen, they told me that they did not have the other medication they were supposed to give me, I do not know their type.* |
|  |  |  | *Like in our case as in charge, I do go to the senior pharmacist and he says we have ordered them from central medical stores so if they are available we will let you know.* |
|  |  |  | *Sometimes it’s the mismanagement of the us because our pharmacy we order drugs, it’s like through replacement, they will use the empties, and send them to pharmacy for them to supply the other drugs, so if we do not have enough empties so they will likely give less amount of the drug that we require, so it’s like that the other doses the patient will delay, or will look from other wards if they have fragile let us use yours, when we order we will replace, so these are some of the challenges that we meet.* |
| 1. **Referral to private hospitals and pharmacies affect patients from poor households** | Participants expressed that most often, in case of non-availability of medicine in the hospital, they were informed to buy from pharmacies. In certain instances some respondents were advised to go for further examinations at private hospitals in situations where such equipment did not exist in the hospital. These decisions had different responses from patients – depending on availability of money by the patients, some would go while other would not. However, participants wished medication was readily available in the hospital without being referred to buy | | *Then they explained to us that the problem they have is with their machine. The pictures that are getting are not clear and there is need that we should go to XXXX for a better picture. They said we should discuss among our relations in order to raise the money. I asked if there is no where we can get the same services apart from XXXX. They said no. Later on Tuesday a professor came and was explained about or problem. He said we should receive medication and go home.* |
|  |  |  | *In such a case we ask the guardians to buy of which maybe they come from far and they don’t have anyone to help them and it takes time for them to find the drugs of which at times we might find that the patient condition is getting worse and we find it difficult to take care of patients, and another alternative might be that for example a patient is supposed to get Amoxil but if we don’t have we always have alternative drug but we wait for the guardian to tell us that they can’t afford finding the drug that was prescribed at first.* |
|  |  |  | *Another thing is availability of drugs, they should be able to get the drugs from our pharmacy, not being told to buy from private pharmacies, and some of them are poor that they can’t afford them, they just go home then they get worse only to come back again.* |
|  |  |  | *No, the same that am taking because they gave me 30 for morning hours, afternoon and evening but I wasn’t given Panadol so I buy.* |
|  |  |  | *I remember one day they said we need to buy from private pharmacy, I don’t remember the name of the drug. My wife went to buy.* |
| 1. **Transport challenges** | Patients and Guardians raised the issue of transport challenges as one that affected timely access to care for patients with sepsis. Patients are normally delayed mainly as a result lack of transport to PHC facilities and from PHC facilities to XXXX. In this section we highlight the experience of patients and guardians in getting transport to access care. | | *The time I was being discharged they had not removed the drip tube, and I went to see a nurse after I got back from getting drugs, I showed the nurse the form and the medicines, and I told the nurse to come remove the drip tube, and I was told after I have finished packing my stuff, and I should pass by they should remove it and I did that, they removed it and he was also given some advice for second time and then we said goodbyes. We waited for ambulance that was going the same direction which we were despite that it was supposed to carry funeral we still joined them and they first dropped the body at XXXX and proceed to drop us at home.* |
|  |  |  | *The major problem is transport, even when the patient is seriously ill you still need to provide your own transport to the clinic or even when you are referred to a hospital.* |
| 1. **Limited number of ambulances affects and delays referral to hospital** | The normal pathway for patients with sepsis is that they are firstly required to present at their local clinic which in turn they are referred to either XXXX or their District hospital. The district hospital can then further refer them to XXXX. In both instances, we learnt that patients are expected to find their own transport when referred to XXXX. Most often patients would find money to carter for transport costs (increasing the out of pocket expenses on the illness). In few cases, patients had to stay longer before going to XXXX while they search for money, thereby delaying patients’ access to timely care. In one incident, a patients was sent alone to XXXX in order to save on money. It is a common occurrence to see people carrying patients on their back to XXXX. In a special experience, one patients was provided ambulance to and from XXXX (after discharge) for being a member of staff at the local clinic. | | *No, form the clinic we went home first because it was on a Sunday, and the next day that’s when we went to XXXX and we were welcomed and after she was tested and the result showed negative but she was given injection and we were told to take her to XXXX if we had transport because the ambulance would take long, and we left for XXXX.* |
|  |  |  | *We used our means of transport and because we did not have enough money for transport me and my mother did not go with the patient as the expectation was that he was going to come back, we called our brother in XXXX to take him here and do the scans, when they are done he should board a bus back to XXXX.* |
|  |  |  | *At the clinic level they helped us really well as expected, the only problem we have there is that no matter how sick your patient is you have to find your own means to the district hospital, they only help pregnant women in this regard.* |
|  |  |  | *We can say it was not easy to get here because there are no ambulances to get patients from the clinic, we don’t know if the transport department is still working here but we had to borrow money from people so that we could hire a car to drop us here.* |
|  |  |  | *The challenge was that his heart was beating fast, which made us walk slowly and we got on a bicycle, and we even knew that we won’t be there in good time despite that we are supposed to go to hospital in good time. We got on a bicycle to XXXX stage, and then boarded a minibus to main stage at the trading, we walked slowly to here and that’s when we waited there.* |
|  |  |  | *It around 7, we got helped, and tested but we were told that X-ray machine is not working from the start. They tried to give us drugs and the ones that they thought could help, and then they gave us a bed, and everyday Jackson, three time, morning hours, afternoon and evening hours. It was close to weekend and we had to wait for Monday for doctors to come and we were told to that he needs to be tested but we weren’t guided where, the patient who was close to us had means of transport so they left for Mulanje mission, got tested and came back, and we had to make a decision because there it was costing to 13000, of which we didn’t have, and that’s when we decided to go to XXXX, and the doctor gave us a letter and because of transport we decided that patient should go alone.* |
| 1. **Lack of transport/high transport costs, delays access to PHC facilities** | There were instances that participants struggled to get money to cover transport costs to local clinics. These were situations where clinics were very far from participants’ homes and required bicycles for transportation. | | *I failed to walk properly because of dizziness, and then I decided to go back, then my mother came asking what happened, and I told her I wasn’t feeling well, she bought buffen and then things weren’t improving, but I couldn’t go to the hospital because I had no transport and that month I really struggled.* |
|  |  |  | *I told my relatives that I needed to go to a hospital but there was no transport, the next day they managed to get transport and we went to a clinic where they told us to go to XXXX.* |
| 1. **RELATIONAL ASPECTS OF CARE** | Literature and different theories on patient experience of care emphasize on relationships between and among patients, guardians and HCWs as an important aspect. Positive relationships is often associated with positive experience of care. In this study, we explored the nature of relationship along the sepsis patient care pathway and how it influences on patient experience of care. Key thematic areas that came up included: attitudes of both patients and HCWs, existence of conflicts, favoritism, and HCW engagement with patients on their health condition, communication and working relations among HCWs.  There were mixed responses among patients, guardians and HCWs on the nature of relationships in the hospital. Much as most participants that indicated the existence of conflicts, poor communication and coordination in the hospital, we learnt that there are specific individuals and encounters that are normally associated with such practices and not necessarily a reflection of the whole hospital. Participants were also able to highlight experience of positive relationships.  In this section we reflect of key example or occurrences where both positive and negative relationships are likely to occur and their effect on the care of patients with sepsis.  HCWs indicated that difference experience of care and how they relate to patients may be as a result of health system factors. Patients that are admitted at a time when the hospital has fewer nurses or certain types of drugs are not available, may have a negative preselection of the HCWs and feeling of not being assisted.  Some of the common things that patients always complain is the long periods of time that HCWs take to attend to them. Patients also complain of lack of proper assessment/ taking of detailed history by clinicians but just rushing to provide medication. One patient complained of not being listened to by the doctors on medication he stopped taking due to side effects | | *The relationship was good generally, the cleaners would come in and ask politely to clean the room, the doctors were always available and the communication was good too. The nurses were there all the time, it could happen that the nurses would act somehow questionable, like they could leave the medicine on the bed and come back later but that’s not a big concern they are human after all, they get tired as well. Overall it was a good relationship.* |
|  |  |  | *Yes, it happened in the XXXX, some women were complaining that a nurse was called a number of times but the response was always rude and they gave up.* |
|  |  |  | *I think they need to change the way they handle findings after assessing patients, I understand that some information is sensitive but a guardian is supposed to know what he or she is doing in order to give the best care to the patient. A guardian is supposed to know how to help a TB patient or HIV patient, so the doctors should be able to tell the guardian what the patient is suffering from and how to protect themselves if the disease is contagious. If the guardian is not sure about what the patient is suffering from, it would be difficult to properly take care of the patient* |
|  |  |  | *And sometimes a patient may come while we have 80 patients and one nurse, and also someone comes while we have drugs someone while we don’t have, they could have a different experience.* |
|  |  |  | *No, we have a problem of team work, we as clerks, the nurses and doctors are our bosses so if you try to talk to them about patients you will be accused of undermining your boss.* |
|  |  |  | *Sometimes you would you meet a patient or relative outside when they have been discharged they would say thank you very much you assisted me and you tend to wonder when, but some I would say yes it will depend on the particular healthy worker who is attending to the patient.* |
|  |  |  | *Of cause the other point will be what the guardians normally say that maybe my patient hasn’t been seen, to them they think that they have stayed 2 or 3 days no senior coming to see their patients, and maybe they expect a quick improvement within maybe a day or 2, they would there patient recovered and see discharged* |
|  |  |  | *The first thing is I have also seen guardians that when you see their relative in good time they also see that you have tried your best to do all the possible investigations by giving them the treatment, the patient has gotten better, they come to you and say doctor thank you for your effort, even when the patient dies but if they have appreciated the care you provide to their patient* |
|  |  |  | *The first that people always complain is the time it takes doctors to see their relative, they wait only to be seen 2-3 down the line, yet the patient is very sick. Guardians also complain if a doctor do not asses them, you know there are some clinicians instead of taking detail history, they will just say, what’s your problem, aa no am feeling fever or what, they check the temperature is high, ok ,malaria, without properly examining the patient, yah, so lack of proper assessment* |
|  |  |  | *I explained to them that I stopped taking Bactrim but I was still told to go and get Bactrim so that we observe what will happen after. After I take Bactrim I feel itchy and then if I start sweating things got worse.* |
| 1. **Perceived poor attitude towards work among some HCWs** | Poor attitude towards work, and feeling and acting of not wanting to work, was an issue that was raised with both patients/guardians and HCWs. There was poor attitude and relationship that was reports between HCWs working in different departments affecting handovers and continuation of care from one department to another. Patients also felt that HCWs non commitment to work delay the treatment process. However, some health workers felt that their perceived slow approach to work is normally associated with poor attitude towards work when in actual sense is as a result of high workload. Other HCWs associated poor attitude among HCWs towards work due to lack of incentives including additional allowances (which normally come in other wards through research studies) and lack of training opportunities | | *I will talk of attitude in EMERGENCY, a good number of clinicians in EMERGENCY do not have good attitude towards fellow nurses who are working in other departments, yeah even attitudes towards patients, and they complain a lot.* |
|  |  |  | *They said I should wait for a senior doctor who was not available and yet they had their phone number, they could have called the doctor and explained that we have a patient whom we think has this problem, and it started because of something, they could have explained. For example like the lighthouse, they are open and friendly to patients, they call patients for counselling, like maybe they would say we have found out that you are HIV positive and they show you how to take care of yourself and if you have questions you ask.* |
|  |  |  | *Mostly it is the attitude of the staff, the way they commit to work is what delays the whole process of treatment, I have been here since 2014 and I know all these things. You can come one of these days to see for you what is happens.* |
|  |  |  | *In XXXX, XXXX we have task allocation whereby we locate nurses according to bay, and every nurse has to be on a specific bay, looking for those patients, normally they either 15-20 per bay, so what we do is every morning after we do the hand over you go on your bay, in case there are students together you make the environment to look conducive, the bed making, the dusting and what so ever and then you come to the vital signs checking then following up on what was supposed to be done on this particular patient, did we miss something in case the patients’ needs blood, we need to get samples and send to the lab, and I think the patient is very sick cannot eat, we need to connect an NGD so it will depend on that team on that particular bay, if they are willing to work as team, they will work effectively so I would like to come in that ward to meet that nurse and see maybe guide him and maybe this nurse is not willing to do that. So that means I can’t manage if I have 3 to 4 wards that I need to go around, I can’t stay on a particular ward the whole day I need to also see some other wards so sometimes I would come in and say let’s work together, if she is interested we will work together and maybe leave part of the work and say continue do this, but if this nurse is not willing then it’s a challenge so they will seem like you are policing them when they see you now they will be like heeee, she is coming. So to me I would say that’s the challenge but if the nurse is willing to work together then I feel like my job is much easier when I meet such nurses.* |
|  |  |  | *The other part the drug might be there but sometimes the frequency of giving drugs is 6 hourly, there are other nurses they don’t give according to the prescription time so it’s the attitude part now because the 6 hourly is 6-12 6-12, or 10-2, 0r 10-6 . Roughly it’s 6 hourly. The gap of 4,10,2,4 like that, some would think that aaaa, 12 midnight maybe they were resting , waking up go give the drug, so on that part it also affect the management because likely the patient will not get the medications required, because when we are giving the drug we also consider that on the half-life that when the drug is in the system when it ends another drug has to come in so regarding that it’s a challenge so the attitude part to me it’s something very difficult to say that you can end it, if we cannot end it despite that we have been talking, I would give you an example last week there was a patient who needed mobiliser for hourly, some of the nurses were doing but some others were not, so despite talking every day you would hear the doctor complain the patient did not get the drug ,why aaaa, we forgot and yet the handle over was Cleary written and they did the other things except this patient with 4 hourly mobilize, asking you mean you didn’t see, they will say aaaah no, so on that part I think it’s a challenge* |
|  |  |  | *I would give an example or even transfusion maybe there is results is 2 gram the patient needs urgent blood but they haven’t done that they haven’t followed up. So sometimes is the attitude of the, that particular healthy worker who was attending to this patient.* |
|  |  |  | *Sometimes it’s also a few individuals, they would receive a call that we are sending a patient in the XXXX sometimes there is not space in the XXXX, we are supposed to tell them there is no space, or maybe if the patient comes will be on bay one because XXXX is full or we will have space but we will not have oxygen so if the patients’ needs oxygen likely will suffer, there is no oxygen. Maybe the other part I would say it’s like the attitude part am saying that they are not ready maybe to them it’s like disturbance instead of them resting, now a patient will come and make them busy, what so ever.* |
|  |  |  | *Medical wards especially XXXX, XXXX and XXXX these are the departments which are very busy but they don’t have any motivation at all but when it goes to other wards which are a bit stable, there are some motivations, maybe they are given top up allowances so with that people may have that passion of working hard towards the patients. A good number of staff when asked where do you work? And you say XXXX, they will tell you that you are working at XXXX. So for me this can be improved by giving them motivation through money or any kind of gifts.*  *Other wards receive such kind of allowances because of studies, maybe there’s a certain study in their ward so all the nurses benefit from it.* |
|  |  |  | *From what they say, some would say maybe because they are not being considered on training issues, allowances are not much, I would say on welcome part, they would come on free time to work like a part time work, so some they would mention of that but sitting down with a particular nurse and finding out, I have never done that.* |
| 1. **Communication between HCWs and patients varies. Good communication is prioritized.** | Patients, guardians and HCWs generally had mixed experience of their communication with each other. Others had a positive experience while some had a negative experience. As earlier said, such negative experiences might pertain to certain individuals or scenarios. However, such experiences are worth highlighting and drawing lessons from. In this section we present a few example of where participants experienced both positive and negative communication from either HCWs or patients/guardians.  Some HCWs felt that generally patients have negative attitudes towards them thinking maybe we will shout at. This was confirmed by some patient who said that they are afraid of asking HCWs anything for fear of being shout and that they look at them as their bosses who know everything.  Sometimes there are misunderstandings between patients and HCWs over medical procedures, which leads to conflicts. For instance, a patient may feel that he/she is not getting oxygen and yet HCWs know that the oxygen is working well. Doctors felt that sometime the rush to see many patients end up having no adequate time to properly engage to communicate to patients | | *I am not fearing anything, I am telling the truth without hiding anything. The relationship between us and the healthy workers is great, ever since I came I have never heard them shouting at us or speaking something bad about us.* |
|  |  |  | *Because we meet people from different homes with different attitudes, there are some who are friendly, they will help you if you approach them regarding an issue with your patient, and there are some who will not be happy if you approach them, they will be shouting instructions; just go and do this and that! They need to improve how they communicate with guardians and patients.* |
|  |  |  | *For me there was this other day my patient was trying to remove a catheter, I went to inform them (nurses) about it, they said that it was installed by the study nurses so it is their responsibility to remove it but considering how dangerous it is if he removes it by himself they agreed to help. But there was this doctor who got very angry because a patient removed cannula, the patient did not have sense of anything at all because of his sickness but the doctor was angry anyway so he gave him an injection without the cannula which I thought was out of order.*  *There is one particular nurse who is known for being rude to guardians and most guardians dread facing him to report any issues regarding their patient, one day I got a terrible headache and I went to ask for some Panadol but he shouted at me to go to the clinic.* |
|  |  |  | *I believe our relationship with those people is great because we work together as a family, and we see them as our guardians because whenever we have a problem we call them to assist us, even at night when death has occurred they help us.* |
|  |  |  | *The workers clean, and if you don’t want to give them space to clean it then who is tough? Yet you’ll sleep alone on the place, and sometimes people would eat biscuits and throw the trash on the same place instead on the bin, and sometimes even the remains of a maize cob with a mind of saying the cleaners will remove the trash.* |
|  |  |  | *I was close to the nurses’ room, and when people would come to knock, the nurse would help them without resistance, sometimes people would come looking for space to sleep the nurse would assist and also look for mattresses for them and advise them that if someone is out they will give them good place.* |
|  |  |  | *After going to the ward I did not see any problems until I noticed that some nurses were not friendly, for example there was an incident when a patient whose bed was near to my patient’s bed complained that a catheter was loose he should help him to make it stable but the nurse didn’t do anything about it, that night the catheter fell off when the patient was sleeping, the nurse came and was like; why did you remove the catheter (Cannulas?)? I will inject the drug on your butt or if you don’t like it you will follow me to the office to fix it for you there. Imagine an elderly man should be pushed on a wheelchair just to get a catheter fixed.* |
|  |  |  | *This is another problem, the doctors come here as if they just had an argument with their spouse, no smile on the face, and they talk to patients as if they are arguing with them.* |
|  |  |  | *An incident happened someone died yet the patient looked health, but the guardian didn’t know what the patient was suffering till the time the patient died, it happened that they inserted a tube inside the patient but when bringing it out it had blood, and it didn’t take long for the patient to die, and the guardians complained that they killed there patient yet they didn’t know what the patient was suffering from. They were called and the doctor explained to them to find out that the patient had very serious conditions and the guardians were even surprised to hear. And I believe it’s good to communicate at the beginning so that they should know.* |
|  |  |  | *Patients who have gone to local clinics several times without improving may decide to come here without reference, they may have sepsis but they may be denied treatment if they come without reference. I would like to see nurses checking health passports for patients without reference before sending them back, this will help to identify septic patients.* |
|  |  |  | *Most patients who are not referred from their local centers are turned away in a disgraceful manner without even checking them if they have a serious issue. There was a patient who was very sick but was turned away by the lead nurse, so it happened that I knew the patient and helped them to talk to another staff and they got admitted, unfortunately the patient died the following day which means the patient could have died at home because he was turned away from hospital.* |
|  |  |  | *I think it’s our attitude, they’re more open to you if you show them respect. I don’t really if its culture in Malawi but people don’t respect patients in my opinion, we tend to dismiss them, we don’t talk nicely to them, and we scold them far too often. But if you show that you respect them and you care and you understand their circumstances, find out why they have made the decisions they have made instead of telling them off: you made the wrong decision, why did you do this bla bla bla, but if you ask them how come you did this then they start telling you and they become more open they even tell you things that were not on the file then you start to understand what’s going on. With me, I have realized that I do this when I have few patients but if you have a lot of them I don’t have the time to sit down and talk, I just do what is necessary at that point.* |
|  |  |  | *Basically I think our (HCW) attitude is what they (patients)don’t like, a cleaner comes to clean and will shout, a guard comes to tell the guardians out but the way he talks to them is not respectful, the nurse’s attitude has improved tremendously since when I was in school third year, I have seen a great improvement, for doctors I don’t think they are disrespectful to patients, at least for the ones I have worked with, doctors we are not disrespectful we are just in a hurry and a bit aloof to their complaints* |
|  |  |  | *Some doctors don’t like to be asked questions. When patients ask - doctor why do you think it’s not ABC, doctors ask - are you trying to treat yourself? I have cases whereby a patient will be in the ward, for maybe more than two weeks, you ask them what is the problem they say they don’t know, “the doctor didn’t tell me, aaaa, doctor didn’t tell you? Yet a patient has got a very bad disease or condition, aaaa, why did the doctor not tell you? I don’t know, I asked but they were not giving me any information, aaaa, he was even mad at me because I was asking too many questions”. So yes we do get those type of cases whereby guardians or patient complaining that they weren’t informed of what is really happening with their patient.* |
|  |  |  | *In the past a doctor or healthy worker would be seen as a semi God. Doctor can talk to the patient but the patient will not have the courage to ask what do you think is the reason why am sick, or what have you done, or what treatment have you given me but these days with the change of education system, during the training there is also emphasis on communication, the importance of communicating to the patient and the guardian informing them of what’s likely of the diagnosis of the illness, the investigation that we have done, the treatment you are giving and why, what are the possible side effects from the treatment that you are giving, aaaa, and things are improving, we do encourage that, even when we are doing ward round, that after you have seen the patient, you also discuss with the guardian and the patient this is what we think you have, we have done these investigations and these are the results and we are going to give you this treatment because of ABCD, it does happen but not always, so it’s still work in progress, but communication to patient is very important, very important, when I was doing my undergraduate training, I was not assessed on my communication skills, but these days it’s in the curriculum, we also do have a communication station, just explaining the diagnosis management to the patient ,or even breaking bad news or even aaaa, telling them that this disease there is nothing we can do but there is these ways of palliative care. This communication is very important and I have seen it, it makes a huge impact on the well-being of the patient but also the guardian in the ward* |
|  |  |  | *It happened to me as well, with my condition I have to choose what to eat. One day a doctor found me eating porridge which is provided at the hospital, he told me to stop eating this porridge because there’s salt which is not good for me. I have to eat frequently or else I loose conscience so one morning I asked my daughter to get something for me to eat as I was very hungry, she went to buy cooked cassava, she mixed it with avocado and salad and gave it to me to eat then this female doctor walks in and she saw what I was eating, she rubbished me like never before, I was not happy about, she could just tell me that I wasn’t supposed to eat that like the other doctor did. She was like this is why your disease is getting worse you just eating anything. I said am sorry doctor but she kept shouting; don’t say sorry to me apologize to your body, then I just kept quiet.* |
|  |  |  | *It was a good relationship, the cleaners would tell all the guardians to go out for them to clean, they will be called if needed. When nurses come they asked if you have received your medicines, if not they would take your file and bring your medicines and water, so I think it was a good relationship.* |
| 1. **Roles and conduct of care givers** | We explored the roles of care givers/ guardians in the provision of care to their patients and how that affects patients’ experience of care. We also aimed to understand factors affecting role of providing effective care for their patients in the hospital. We understood that guardians play an important part in the care process and their relationship to their patients and HCW may contribute to either negative of positive experience of care among patients.  Participants raised that guardians are normally affected by unavailability of glove and toiletries in the hospital and they are required to buy. They also complained of the place the sleep which are normally very small, under the bed and they fear getting infections while in the hospital. Some guardians complain of income loss during their stay in the hospital especially those that stay for longer periods of time. | | *Yes, the gloves are there but not enough, sometimes when you go to get some you find that there is no one to attend or they are finished you have to wait while your patient wants to be attended to. It would be better if they could just put a carton in the room for easy access.* |
|  |  |  | *There are many, one, you are used to busy life at home, and then you go to the hospital and stay 2 months without doing anything, it affects your daily income, money, soap, and many more because you are staying at one place and the other thing is the place you sleep, under the bed, just shows that things are not ok, some sleep outside and at least now they have improved, because at first the guardians were not sleeping there, they were sleeping in the kitchen I think or somewhere else. If patients are sleeping on the floor, it’s hard for guardians to sleep with them.* |
|  |  |  | *They (guardians) do complain about time taken to get treatment for their patient, sometimes a patient may have complications in the ward and they struggle to get the nurse to pay attention, other complaints include food, beds to sleep on and blankets and so on.* |
|  |  |  | *Most guardians do not know how to take care of a patient, for example sepsis is affected by hygiene and many homes are not that clean* |
|  |  |  | *The other point will be what the guardians normally say that maybe my patient hasn’t been seen, to them they think that they have stayed 2 or 3 days no senior coming to see their patients, and maybe they expect a quick improvement within maybe a day or 2, they would there patient recovered and see discharged* |
| 1. **Care givers were often in conflict with guards, patient attendants, nurses** | An important element that frequently was raised by most respondents was the constant conflicts that exist between guardians and security guards in the hospital wards. We believe it is an important element to raise as it would influence patients’ experience of care despite guard not being frontline care providers.  We learnt that when cleaners are cleaning, guardians are required to go outside, and when doctors are doing ward rounds, there should only be one guardian. Security guard normally chase guardians from the wards and this does not go well with the guardians. This becomes more of a concern for guardians whose patients are very sick. On the contrary, nurses said that guardians do not understand the need of going outside and giving space to the patient to be alone (i.e. for privacy purposes) and let HCWs do their work. They always want to take the role of nurses to show that they really care. Otherwise HCWs would only need guardians whose patients are very sick. | | *Guardians we also ignore hospital guidance for example if they are giving out drugs we are supposed to go outside, most guardians don’t go.* |
|  |  |  | *There’s another issue that I forgot earlier, when a guardian is refusing to leave a patient because of the condition of the patient the guards will report you to the doctors and you get marked, they can even discharge you early before your patient gets better.* |
|  |  |  | *Sometimes the security guards do not understand the guardians when they want them to go out, some patients require a guardian on them all the time. Patients have been falling off their beds. Last week a patient escaped in the evening when all guardians were forced out, he was mentally unstable, so a patient like that can never tell the doctor he is mad, a guardian should be present to report such things so that he can get treatment.* |
|  |  |  | *We are like their clients and they are supposed to show us discipline. If something has gone wrong, talk to us nicely and sometimes if something has happened, they even say they will report us to matron yet if the matron came would say something different with what they are saying. A matron is like a parent who when something has gone wrong, they can’t say you have been discharged because a guard has reported something to them. But some day we might tell her that we follow our taxes here at the hospital, we work and they tax us and that’s why we come here to see any type of doctor at a government hospital.* |
|  |  |  | *Security and the people that mop, there is a big problem, being a government owned hospital, security guards, they don’t talk nicely to guardians, they act like they are nurses, and as I am talking this something happened to me when I was there. The second time, I was told to go look for a wheelchair by a doctor so that we go to x-ray to check on the patient, when I was looking for the wheelchair, then another guard was like someone died and I escort them to the mortuary, and I was accused of not attending.* |
|  |  |  | *Another day, we were told to go out of the ward for them to mop, then my patient was in pain, her legs, then she told them to call me so that I should go get drugs for her, the one who was mopping refused to open a door for me while my patient was in pain, even crying and then I saw the one who was mopping pouring water outside and I said I want to go see my patient.* |
|  |  |  | *The other challenge is to the part of guardians. I think they don’t understand that this is restricted area (XXXX) whereby we want to have one guardian pa patient, though sometimes it’s for the privacy part and sometimes is also for the management part that we don’t want the ward to be congested.* |
|  |  |  | *Somehow guardians would want to take the role of the nurse or they will really want to show that they caring for their patients, some of the things they do its not required for them to be doing, or either because the nurse would say aaa, do this, like a patient with catheter, there is urine draining, it’s not the responsibility of the guardian to do that, it’s the nurse, so some of the nurses they would instruct the guardian aaa do this, so whenever you meet them they will say but we were told, so we say no, that’s not part of your job, it’s for the nurse, so some maybe they feel if I don’t do my patient will say am not caring, or if I don’t bath the patient, they will say am not caring but maybe what they will expect them is just provision of water because we don’t have hot water, so sometimes they are asked to provide hot water from home using a big flask, or the guardian cook they can bring so that maybe we can clean your patient so that your patient should at least look smart, be able to sit on bed side, be able to talk to him or her, being near because when the patient is clean it’s likely that there will be odors or what so ever that will prevent this guardian to sit comfortably, so I would say on the much of the complaints, I think many it’s that but the other it’s like they would want to be on bed side through out, so whenever we tell them that please go out, be on the guardian shelter, allow this patient, if this patient is able to ambulate, is able to talk, would love on bed side to be the patient alone unless we require your attention would call you so the moment we do that, most of the guardians they don’t feel it and they don’t accept.* |
|  |  |  | *I think guardians are concerned about if their patient is receiving the correct care. Patients with sepsis you start giving them antibiotics as soon as possible, maybe one of the things they will be worried about is their patient is not improving so they will call you aside and say we are not seeing any change.* |
|  |  |  | *The only communication there is with the guards, they don’t talk nice. They don’t understand that there are some patients who need a guardian by their side all the time. Sometimes they argue with patients, if a patient asks them to call their guardian, they will take longer than required or they don’t call them at all. They will even stop you from going to the bathroom, when you ask them why they say it’s the doctor’s orders. A patient may need something inside they won’t let a guardian in to help the patient.* |
|  |  |  | *There was a cleaner, people were eating nsima and it happened that he told them that they should move, he wants to mop, and they said they want to eat where they are and when they are done they’ll tell him, and he started shouting.* |
| 1. **Care givers want to always be in close contact with their patients especially with incapacitated patients** | Generally, many guardians were unhappy with the system of sending them outside while the cleaners clean or doctors see patients. They were more concerned of patients who were incapacitated and could not properly communicate with the doctor or support themselves when go to the toilet. | | *Second thing is this system of forcing out all guardians when doctors come to see patients, some patients have unstable minds and can easily hurt themselves or others if they are left alone but they insist so one day my patient caused problems while we were out and the nurses could not help him so I was furious about, he was trying to get to the toilet to pass urine but he doesn’t know where it is because of his memory problem until he peed in his pants. It’s a system every guardian is not happy about.* |
|  |  |  | *And this system of getting rid of guardians when doctors come to see patients, they have to see which patient cannot be left alone not all patient can talk to the doctor, others have mental issues that they may act like they know what they are doing but they don’t, a guardian is supposed to be around all the time, I once refused to go back in when my patient was troubling them because they would not listen when I told them he was not stable to be left alone..* |
|  |  |  | *I think the only problem I saw was demanding guardians to leave the ward when doctors come to check on patients, there are some people who are very sick that they need someone by their side all time. Problems happen sometimes when guardians are out.* |
|  |  |  | *The issue with guardians was a serious concern to me, when the doctors are coming to see patients all guardians are sent out, some patients are not able to talk, some can’t even open their mouth to take the medicine and the doctors struggle to give them treatment. Other than that I think things are ok, especially now we are seeing more doctors on duty.* |
|  |  |  | *The problem is the patients who are using chambers, because they would chase us out but the patients wouldn’t care after themselves, sometimes the patients didn’t have strength to go the toilet, and sometimes even the porridge would come while we are outside and our plates and other materials are outside and if you want to go run inside they would tell us not to enter, therefore this policy has to change because we can’t be outside for hours and what if the patient wants to use toilet and they fall there.* |
| 1. **Deplorable relationship existed between some care givers and their patients** | Respondent to the study expressed dismay to how some guardians care for their patients and recommendation that the hospital should be planning to orient guardians on the same. One guardian was worried that there are some guardians who don’t treat or take care of their patients with kindness. She mentioned of one patient who had not eaten for some time and was crying begging the guardian to give her food. The guardian, who was the patient’s sister, paid no attention and refused to do so. Other people offered to give their food but the guardian did not receive the food. The patient died the following day. However, some guardians show some companion and love to their patients. | | *Sometimes us guardians we do things that delay our patient to be helped, I have said this because of my in-law. The patient even said that the brother shouldn’t come, he was doing things differently from instructions of how to take care of the patient like instead of dissolving the drugs before giving them to patient, and he wasn’t.* |
|  |  |  | *In our ward, most patients will show signs of mental disorder, they will be doing abnormal things sometimes, so you find some guardians are outside leaving such patients alone, I have seen a few times a patient falling down from the bed because there’s nobody to help them, that’s not what guardians do, you stay with the patient at all times, if you want to go out someone else should be there.* |
|  |  |  | *Some guardians abuse patients. They would shout at them, and sometimes we have to ask them things with love, like what are they thinking, do they have any pain or why are they sad but don’t shout at them.* |
|  |  |  | *Guardians should also love their patients, sometimes patients are not easy to deal with, especially when they are in great pain, they talk unnecessary things that can sometimes offend the guardian but we must remain calm and be patient with them, don’t abandon them.* |
|  |  |  | *Most of the times it is about food, some patients refuse food or eat just too little. So most guardians become frustrated and they shout at the patient in attempt to force them to eat a bit more, forgetting that when you are sick, your digestion system becomes slow as a result a sick person feels full in the stomach for longer periods than a healthy person.* |
|  |  |  | *I have seen it twice a guardian shouting at his patient, but this is how human beings are, it can happen even to me, but it’s not good, it causes unnecessary stress to the patient and some may wish to die so that others may live in peace.* |
|  |  |  | *Thank you, the patient is my young brother, he was having unending fever/flue, we tried to take him to different hospitals but nothing was changing because they all prescribed the same drug, when we came here, they did some tests and scans on him then they found that he has TB, so the doctors were there to help him with drugs. Because he was having difficulties in breathing they decided to give him oxygen but he was refusing because he saw another patient die because they gave him oxygen, so he was scared, they called me in and I reasoned with him that doctors do not kill patients, they help patients to get better, the one who died it was just time, nothing to do with doctors, and he agreed.* |
|  |  |  | *Some patients come with no guardian. On the nutritional part then it’s a challenge, because as we have said the hospital does not provide nutritious food, so we rely on the food coming from home so if there is no guardian then it’s a challenge* |
| 1. **Acts of favoritism exist, leading to unequitable delivery of care** | We explored equality in the distribution and access of care by patients with sepsis. We aimed at understanding whether elements of favoritism existed in the hospital there by denying of prioritizing individuals in the process of delivering care. This was based on the need the patients with sepsis require urgent identification, assessment of severity and timely treatment. We explored whether patients were required to pay for certain services in expectation for better care, whether patients would access better care because of the social status and relationship they have with HCWs and generally what are other factors that influence favoritism if the existed? Most respondents (including HCW) agreed that there are elements of favoritism that happen in the hospital. However, some participants expressed that participants might be mistaken to be receiving more attention as a result of favoritism and yet it is the nature of their health condition that is necessitating that. | | *They provide the care to all patients equally. There is no segregation or favoritism of any sort. They move from one bed to another until all patients are seen* |
|  |  |  | *Patients I believe all have different diseases, and if there many healthy workers helping the patients that means it is part of the job since they give each other shifts, and patients may have different status of illness some you would find them eating some not and those are two different things and I wouldn’t say there is favoritism.* |
|  |  |  | *Sometimes it just happens because the patient looks like he knows many things and if we make a mistake, they know the rules and instructions and they would sue us, but some would treat someone in with negative thoughts that they have nowhere to sue them and which is bad to have those thoughts.* |
|  |  |  | *According to my experience guardians or even patients themselves do influence based on the way they are behaving, mmmmm we may have these two septic patients, one is quiet when is being treated and the other one is like telling you what to do, shouting at you why are you not doing ABCD I was told that you should do this today, so the nurse or the doctor will just say she knows what to do.* |
|  |  |  | *yeah it happens, like I said, nurses or doctors do have attitudes in them so sometimes according to this attitude, they may attend to some patients and say I am tired and go. Someone is calling them to check them they don’t go, when they come back tomorrow they go back to the same patient they treated yesterday and then he gets discharged, and the other one is still in the ward.* |
|  |  |  | *Sometimes people take care of the patient according to how they look, and some not being taken care of, and sometimes patients asking me that they want a private doctor because they say they have seen other people being helped quickly but being junior staff I always say I don’t know. Sometimes patients ask us to get them a doctor. I once met a patient that was helped in a corruption way by giving them drugs and in return they gave out 10000.* |
| 1. **Favoritism is influenced by a myriad of factors including relationship between patient/ guardian and HCW** | For participants that reported experience of favoritism in the hospital, we further explored factors that influence favoritism and these included: being a relative to or they are just know a nurse or a clinician, being a better social background i.e. being educated (HCWs become more careful hence getting more attention, attending to a rude patient or guardian, being seen either at the beginning or at the end of the ward round (towards the end, doctors usually rush to finish seeing patients hence spending little time with them). Although with no concrete evidence, some HCWs felt some patients pay for private services in the hospital. | | *A nurse or clinician can bring a relative for treatment and normally they skip all stages and treat the patient privately without recording in the system, give them prescription then take them to the pharmacy, we miss out on this data.* |
|  |  |  | *Firstly, it depends on who you know at the hospital, for example a doctor’s mother comes in for treatment, everyone will do their best to help and give her better treatment all protocols will definitely be followed. Another cause is the doctor to patients’ ratio, if the doctor has so many patients, they may skip some stages just to give everyone a chance.* |
|  |  |  | *I would say yes, that can happen though I haven’t seen it someone doing it but from the look of things or how things are managed some they would come in as private patient just because they know a certain clinician or they are related to a certain nurse, and they are attended somehow differently.* |
|  |  |  | *When you are at XXXX, everyone is equal. But sometimes you have people that have connections with healthy workers, the system, so they may, even before coming to the hospital, they may call, you know Doc, I have got a relationship or I am sick, I want to come to XXXX, how do I go about it, ok, come to emergency ward, the doctor goes there, they’ll just come in, they are assessed quickly, they go to ideology, quickly they come here, they find a bed, even in staff room.* |
|  |  |  | *Or if you are a politician you just call the hospital director I have got this patient can you arrange a specialist to see the patient, what would the hospital do? You just go call a physician, a specialist where are you? Can you see this patient, now?* |
|  |  |  | *I think I might be biased in my answer, people with better social background are educated so they tend to pick up abnormalities quicker and they tend to alert nurses quicker, they tend to question things more than others so once they do that health workers realize that these people are knowledgeable so they get more careful with them as a result they get more attention. For me, honestly I give attention to the one who is sick regardless of social status actually those with high status irritate me because every little thing they want to be attended to, they get offended quickly, they might invite another doctor from another place to come and see them. Of course when they are knowledgeable, you work very well together but there are some who just question everything, start pestering, getting opinions from several people, so it’s like you make a decision, they question it. It’s not a good experience for me as a doctor. For people that are coming from a poor social background they don’t have all these things at their disposal so the decision is up to me, so for me the first thing is who needs more attention in terms of clinical condition regardless.* |
|  |  |  | *So most of the times, we are trained to treat all patients equally. But for some reason maybe the patient is rude or maybe the guardian is rude, I assume that can also affect the way you handle that particular patient. There are some patients that every time that you come and review them and you had a chat with them, giving them progress and them giving you progress, they will tell you, doctor thank you so much for seeing me, that word alone, thank you doctor, you develop some attachment, and next time that you come for the ward, you would want to see this patient how he is doing, is he getting better or not, and you would want to take time to, ok, and those that are rude obviously no need to have an impact on their relationship with the doctor,* |
|  |  |  | *but what also I have noted in our wards since they are always full, and we have ward around maybe the time maybe between 9 and 12,the patients that you may start with you may have adequate time to talk to and when you have got student you also need to teach them, but the time you are approaching 12, it becomes difficult to see the patient adequately so we may rush things, so those patients may sometimes feel that maybe this doctor didn’t care much about us, so for a number of reasons patients, may feel very satisfied with the care, and others may think this doctor doesn’t care, about my illness* |
|  |  |  | *That may influence treatment, like you have patient A and B both need blood and patient A’s guardian says if you find blood for my patient I will see what to do, so you try your best to find blood for patient A then you get something from them. Because B is poor will remain the way she is.* |
|  |  |  | *I have heard that it happens here, this is corruption and only way for us to have evidence is for one party to admit it otherwise it remains a secret.* |
|  |  |  | *I would say yes, that can happen though I haven’t seen it someone doing it but from the look of things or how things are managed some they would come in as private patient just because they know a certain clinician or they are related to a certain nurse, and they are attended somehow differently.* |
| 1. **Social status and connections influences favoritism** | We also learnt that social status and personal connections with HCWs also plays an important part on how quickly one can get attended to in the hospital. The following examples depict such scenario: | | *We go there whole heartedly that we are going to meet doctors, but it happens that we don’t meet the doctors so I would say it’s something that works better if you have connections in the hospital and some are assisted in quickly.* |
|  |  |  | *It happens, there are nurses or doctors who joined the field because they didn’t have a choice as a result their commitment to the job is questionable, they will be giving attitudes to patients looking less privileged. They don’t want to help people who do not understand what they are saying. They will ignore a patient because he is not smelling good.* |
|  |  |  | *Yes, people get helped because of how they are looking. Sometimes a nurse would just come and talk to a patient on the queue, and people would think the person works in the hospital while in actual sense he doesn’t but he just looks presentable and knowledgeable.* |
|  |  |  | *I would say yes, it can exist though I don’t have actual proof but because of the way we handle our patients, some would prefer going to patients regarding the social status, they would be attached to that while the other patient maybe he is poor, they would ignore that one, or in case there is complaint they would attend that one not attending to the other patients,* |
|  |  |  | *They could see someone but then ignoring the person and they would be walking around randomly, and some people would not get on the line but go straight in front that’s why it took us long time to be assisted despite that we went there in good time.* |
| 1. **HCW engagement with patients and guardians on information about their diagnosis and prognosis.** | Another area of concern that most participants raised was about HCWs ability to engage and communicate clinical information about the patient’s diagnosis and prognosis of their medical condition. The majority of the respondents expressed that doctors do not inform them findings of their assessments. We learnt that some patients had stayed for close to 2 weeks without knowing what their problem was. Others patients make efforts to ask doctors of their result and yet they don’t get any feedback. Others fear to ask as they would not want to be seen to be interfering with the work of the health workers. Some doctors do not like to be asked questions. We categorized responses we got from respondents into: information related to handling of death, health workers’ attitude toward questions and patients’ knowledge of their diagnosis. | |  |
| 1. **Approaches to communication and handling death require some reviews** | Participants raised a number of concerns on how death is handled when it occurs in a ward and they made recommendation on how to improve on the same. Participants complained that time take to prepare the dead body before they take it out is very long. The wished it was shorter or there was a separate room to prepare the body as guardians are kept waiting outside and there is emotional stress among patients that remain in the ward. Some patients wished death could not be announced in the ward to avoid creating panic among patients. Nurses indicated that once they realize that a patient’s condition is worsening, they engage the guardians to inform them of the condition of the patient as a way of preparing them for either positive or negative outcomes. | | *I think they should plan for time that they take the funeral out, for example if someone has passed on around 2am, before 7am, they should make sure they have taken the body out, but if it has reached 7 am without them doing their process, they should wait for people drop food and go, then they do the whole process around 8 so that they shouldn’t be arguments and I think that’s another thing they have to improve.* |
|  |  |  | *This was happening while the guardians we have gone outside, and we would wait close to 50mins or 40mins outside waiting for doctors to finish since after the funeral we are supposed to go outside. We escort them to mortuary, and found ourselves waiting outside for an hour, and if remove those minutes they prepare the body and that’s why it takes so long.* |
|  |  |  | *That time I was a student, we reported the case to the senior, and I was advised to try other possible ways, but the patient died, and the guardians complained why he sent me as a student while he was just on the phone, while they did not notice the input, but all what they wanted was the presence of the senior.* |
|  |  |  | *In my situation normally if I have seen that the patient is very either he is on oxygen or we have just noticed that the levels of breathing pattern is not good, the oxygen saturation is very low, I would normally call the guardian ,explain, that I have noted that the patient is very sick, the breathing pattern has a problem it’s not the expected one in a normal patient, so maybe what we will do is a abcd, what we want is to support on oxygen, let’s try so that maybe the patient can improve but your patient is very sick, normally I do tell the guardians that the patient is very sick the reason of telling this the guardian is to prepare him or her that the condition of the patient of his/her they should expect anything from there either the patient is dying or the patient will improve but we normally encourage if this patient is a Christian to say if you are free to call a pastor, if you feel like you can pray on your own you are free to do that, but am just telling you that you just have to be aware of the patient’s condition, so in case the patient dies so I would still say as earlier said the patient is very sick, yes, our plan was to make this patient recover but unfortunately we are not God, nature has taken charge.* |
|  |  |  | *It was my first time to be admitted in a hospital and that day seven people died, this is the day I will never forget in my life, I couldn’t even sleep, fortunately I was discharged the next day. I lost my mind for a bit, it was like everyone was dying.* |
|  |  |  | *When they realize that a patient is dead they should not announce right there that she or he is dead, that announcement gives pressure to other patients. I think they can have a room like the serious ward, they will be taking them like they are going to serious ward but they be going in that room to prepare the body, when other patients know that one of their friends is gone it blows their minds.* |
| 1. **Patients and guardians lack knowledge of patients’ diagnosis** | The issue of patients’ knowledge of their diagnosis was one issue that was raised by most sepsis patients and their guardians. It was clear that most patient and guardians were unaware of their illness, the treatment they are receiving and the progress of their condition. This questioned the expectation that clinicians are supposed to engage patients in their care delivery and the issue that there were some improvements on patient engagement as reported by some HCWs. There were situations where HCWs would just stop giving a certain drug without explaining, unless the patient asks. For most patients, they got discharged from the hospital without knowing what their problems was. There were situation where doctors would not be willing to would be non-committal in discuss their findings with patients/ guardians. However, there were fewer respondents who agreed to have been informed of their conditions by clinicians. | | *Iiiii, I can’t manage explaining what the patient is suffering from.* |
|  |  |  | *You are supposed to ask them questions. I was watching some event, I think they were giving nurses or doctors degrees at chancellor college and before giving them they was a need to sworn in, the reason they were doing that is for them to accept that they will be treating patients fairly and to satisfy them. You would find a patient so talkative and failing to describe how they are feeling due to illness, the doctors know how to treat them through what they learnt in class and sometimes nurses shouting at a patient. I feel like we are not given a chance to explain more of our problems at the hospital instead we are just given drugs.* |
|  |  |  | *Mmmmm, they didn’t tell me, they just said the problem is blood, we don’t even know whether they found malaria, but what I know is the patient was helped, started talking properly ,started hearing and also talking.* |
|  |  |  | *Unless you ask them for example reminding them why they haven’t given us other drugs, and they tell us that we will not receive those on that particular time but they should be informing us the reason why they are not giving us that medication and if they do that we can’t be worrying that they are not giving us drugs.* |
|  |  |  | *I’m going to talk about what I saw in that ward both about me and other patients. When patients arrive it’s not always that they are given attention right away, for example, there was this patient who was unconscious for two days, he wasn’t eating but the only treatment he could get was Panadol and other drugs. The same thing happened to my patient, for two days the patient was not able to do anything but they gave us Panadol. Sometimes we don’t know what the doctors are doing because we don’t have their knowledge so we may judge them wrongly (issue of communication of what is the diagnosis). But until the people doing this study came again on the third day we did not get any convincing treatment, as soon as they came to ask we were still interested everything changed, the care improved.* |
|  |  |  | *No, they never told us anything, they would come and I will welcome them, ask me how he is doing then give him drugs and then write in the file, they never explained what was going on.* |
|  |  |  | *They were supposed to inform us that they are seeing improvements so they are closing the file to see if he will continue getting better, if he continues to get better you will be discharged any day. They just stopped giving him sleeping pills then I discovered that he was becoming a normal person but still more we needed to know in advance to prepare.* |
|  |  |  | *In terms of engagement, doctors at XXXX hospital were better, even when we arrived there they told us straight away that x-ray machine is not working, they admitted us but we knew what was happening, after a few days of treatment the doctor explained to us that even though the patient seems to be improving, there is need to scan with x-ray machine to find out exactly what is wrong with him, since that machine was not available at that time he advised s to go XXX or XXXX mission to get the scans and come back for treatment. But here, they can take your temperature readings or something without telling the patient what is going on.* |
|  |  |  | *Every day he was getting treatment he was supposed to get, within those days some nurses or doctors would come to collect blood samples and urine samples but results from these samples were not shared to us, they just pinned them in his file. One day a doctor came to see so I asked him about the x-ray results, he said the same thing they told us the first day that there’s water on the left of his chest, I demanded to see the picture, and he opened his phone and showed us the picture from his phone.* |
|  |  |  | *I would like to emphasize doctors should be able to explain to patients or their guardian what the problem is and assure them that they are getting the right treatment, there might be situations not ideal for the patient to know so its ok not to tell them but every patient is supposed to know what is wrong in their body. People are being discharged without any idea what was wrong with them.* |
|  |  |  | *No, he is not back. Maybe the doctor knows the results but they are not telling us, on Saturday before going home I discussed with the doctor the importance of telling us what is going on, or any result from samples they take.* |
|  |  |  | *Aaa, am amazed that ever since they tested her up to date they haven’t said the condition and we just see them coming to give her injection treatment and some drugs to take without mentioning anything.* |
|  |  |  | *Yes, like maybe he was told but I just had a question of why guardians are not supposed to know what type of treatment the patients are receiving, like I said they just come and test things and write in the file without explaining to the patient.* |
|  |  |  | *No, everyone who was doing tests on him was not coming back. The day before he was discharged is the day he was told that he is fine, another nurse recommended another pint of blood but another nurse said no he is fine, so it was complicated to follow what was going on.* |
|  |  |  | *The nurse explained and I also explained very clear, but maybe the patient still didn’t understand and at times the patient don’t have enough information like maybe Sepsis patient maybe the doctor didn’t explain in detail to them what they are suffering from and why they have been hospitalized.* |
|  |  |  | *They were telling me everything, if the results are not ready I was told. The one who did malaria test told me I had no malaria, the one who checked salt levels said it was a little high and the BP test showed it was high too.* |
|  |  |  | *There was a time I was reading my file and I was surprised to see that there was a prescription about TB and yet they didn’t find TB, so I was thinking that maybe it’s a precaution. When I asked them they were not able to explain. I only heard the nurse telling the lead doctor and his team that I needed scanning, that was a day before the day I was discharged. I would read some things from the file too but they never told me what was wrong.* |
|  |  |  | *No, he did not explain, he just gave a paper with some information on it and told me to bring in the morning to receive medicine. He didn’t tell me what disease he found.* |
|  |  |  | *One day the same week another doctor came demanding to take water sample from the patient but my brother was not happy about it, he asked them why they did not bring the results from the sample they took on the first day, it was a painful process he wasn’t willing to do again but after discussing it he allowed it to happen, this doctor promised to bring the results the following day which was Friday but he never did, Saturday the same, I left Saturday evening to go back home. Am coming back today the results are still not here, I asked mum if there was any significant change while I was home she said no,* |
| 1. **Lack of proper coordination and working relations among HCWs in different sections affects continuity of care** | We learnt that there was poor coordination and working relationships among HCWs in different departments. Nurses in EMERGENCY accused nurses in the ward of not being will to receive patient from the emergency department. Likewise, nurses in the wards accused nurses from EMERGENCY of bringing patients to the wards without conducting full assessment as is required. This affects the process of handing over and continuation of care from one department to another. Clerks expressed lack of team work with front line care providers like nurse who always looks down upon them as having nothing to do with clinical work. | | *As health workers in different departments, we do our own work and if you want to ask questions, you may end up being accused/ being in trouble. One hospital attendant tried to inform nurses that there is a patient they need to attend to, they even wanted to beat het. So we just watch since nurses are our bosses here* |
|  |  |  | *No, we have a problem of team work, we as clerks, the nurses and doctors are our bosses so if you try to talk to them about patients you will be accused of undermining your boss.* |
|  |  |  | *I (XXXX nurse) don’t usually go to the wards but for those who do always complain that nurses there are sometimes unwilling to receive patients, they can even say that there’s no space while the space is there, sometimes they think we just want to give them more work, but EMERGENCY is not a ward to admit patients. But sometimes it may be true that there’s no space in the ward so we keep them in the resuscitation room.* |
|  |  |  | *Of cause on that the relationship is not that all good I would say because I would say the attitude part, because some of the part not doing, some of the nurses at the EMERGENCY they do that deliberately, you’ll see a certain nurse will do everything, including the capital, the Iv, the drugs, the samples, the whatsoever, they will do, and come and present that patient to you through handover while others will just send a porta with nothing being done. Go and take this patient to the ward* |
| 1. **TECHNICAL PROCESSES IN CARE PROVISION** | We explored how **processes** in the hospital during sepsis patients’ stay including; HCWs capacity to diagnose and prescribe, timeliness among HCWs and level of motivation among HCWs affected the care they received in the hospital. Participants responses covered a series of thematic areas including: processes of taking blood samples; delays during their care pathway including the waiting time; processes of identifying, diagnosing and examining patients; out of hours investigation; patient monitoring; experience with primary health care and trauma due to death in the wards.  In summary, as far as processes of providing care to patients with sepsis is concerned, participants raised the following issues: delays that happen in primary care facilities before referring patients to XXXX; no significant test that happen in PHC facilities apart from Malaria and a prescription of Panadol; Patients complained about the pain that is associated with taking of water from the back (to test for meningitis?) and wished if there was a lesser process of doing this. Their concern was that the process was repeated several time without succeeding; Some patients are discharged before they fully recover; Participants wished if the laboratory was quicker in giving results for testing blood samples; Some nurses deliberately miss while they are at work. They go to hide and when they are needed they won’t show up; Sometimes HCWs simply forget important procedures they are supposed to do to assist a patient. For instance, blood has come for a patient that needs it they could forget to do so.  However, in other PHC facilities, they prioritized very sick patients, attended to them and quickly referred them to XXXX. | | *She was pregnant, after giving birth we stayed for a week and I thought she was better. Then she started complaining backache, we went to XXXX health center, they said it was normal for new mothers to feel pain in the back it will pass, they gave us panado and we went home. There was no change and she started complaining about the stomach as well so we went back, we explained everything and they told us to wait for the doctor to see us but he was in a meeting by then, so we waited and waited then we asked again for the doctor and was gone and we were told to come again the next day.* |
|  |  |  | *First of all, if it is possible at all to find another way of draining water from the back of a patient. They say this water is what is making him to be talking senseless things. For the first three days it was not that bad, they could drain the water without issues but then it started getting tricky, attempting a few times to get it done, they insert the syringe on the same place every day and it’s a painful experience for me to watch, even the patient himself will tell you how painful it is. I have asked up to three doctors if there’s any other way to do it but they all said no.* |
|  |  |  | *At XXXX clinic I think that’s where they didn’t help up because the patient couldn’t walk, and they were suppose at least to say they have failed , they don’t have drugs, and maybe they could have sent us to another hospital, but the patient went back home. XXXX clinic they welcomed us well and we didn’t stand on a line and at XXXX we also didn’t spot any problem and they also welcomed us.* |
|  |  |  | *Urinary catheter that they installed brought some problems they did not inform us about, every time the patient passes urine blood comes along, this is still happening even though the catheter was removed, they should inform us on possible side effects.* |
|  |  |  | *When we got there, we were not well welcomed because as doctor with experience you would actually know that the person is severe sick, and this one is at least better so maybe if I could start helping the one severe sick, and if it was me I could have seen that the doctors treatment was delayed and the doctor was rude, I didn’t know what they were thinking about up to an extent that the patient almost fell from the bed.* |
|  |  |  | *The patient was complaining a lot, but we thought maybe the patient would get better because of the treatment, but with too many holes created by needles on his body it was a problem, for example one day a white doctor did the same on the another side, and then another doctor came again did the same thing, the time we were being discharged the patient had wounds despite him being sick and was complaining for pain from these areas but we were told that if we spot any problem apart from that we should report.* |
|  |  |  | *If he is improving it that means we have done our best, but if the patient is not improving despite being given the right medication and right care, that’s when you think we are missing something, so that’s one of the challenges,* |
|  |  |  | *The lab should improve on giving results. We do understand that they have too much workload in the lab because samples are coming from different departments but if the staff could be helping us by preparing the samples marked emergency, faster.* |
|  |  |  | *The time the guardians have lost their patients to the time that we asked them questions about like age of the deceased person is too short. Normally the guardians are asked while they are crying. It could be better if they were allowed to calm down after a while.* |
|  |  |  | *When death occurs we do have screams, before having screams we confirm, we assess the patient and when we see that all the parameters do not give us results then we confirm death to the right person then we cover with screams to lead the guardians out, they contribute some money to the guardians. Now we have death certificate, they provide us with the deceased information so that they can use to claim death certificate at DC’s office, so we prepare the body and take to the mortuary, guardians are asked if they want to use main mortuary or college of medicine. When we get to the mortuary we document everything and show them where the body is, if they were referred from a district hospital we call that particular district to send an ambulance to collect the body.* |
|  |  |  | *Sometimes it’s possible that a patient has called for help from the nurse and the nurse won’t show up, they go to hide, then I go to the patient to explain to them not to worry the nurse will come and also ask them what they want maybe if it’s drugs then I go to tell the nurse despite some running away from their duties.* |
|  |  |  | *The patient needs blood and the blood has come but they can’t help the patient whilst the blood is just nearby and they have forgotten* |
| 1. **Hindrances in management of blood samples** | Patients, guardians and HCWs raised some concerns to how handling of blood sample for testing was done and raised some recommendations for its improvement. Such concerns included issues of blood samples missing, repeated taking of the samples when they miss (raising suspicion and concerns among patients and guardians) and presentation of unmarked bottles of blood sample. We explore in detail these challenges in the follow up sections. | | *The other thing I was supposed to mention, there could have been improvement on giving results from the lab. We do understand that they have too much workload in the lab because samples are coming from different departments but if the staff could be helping us by preparing the samples marked “emergency” faster.* |
|  |  |  | *When sample results delay, you might treat the patient in syndromic way without knowing the real condition of the patient.* |
|  |  |  | *There are a lot of problems at the ward. One might get a blood sample and just drop it somewhere without taking it to the lab. We as patient attendants we do take it to the lab but nurses also sometimes take it to the lab. We normally are conscious to take the sample to the lab so that the patient is treated quickly. But sometime a sample might just be forgotten. A sample might be taken at 9am until 3pm before it taken to the lab.* |
|  |  |  | *The patient needs blood and the blood has come but they can’t help the patient whilst the blood is just nearby and they have forgotten.* |
|  |  |  | *When they arrive at EMERGENCY for them to get assisted it takes time, and also it takes time to go to the ward, and to get their samples because at times we want it to be done quickly but you would find out it was forgotten.* |
|  |  |  | *You also need to understand that in order to establish that this is sepsis, they need to test blood through full blood count at the lab. Unfortunately at the lab, they test lots and lots of samples for the whole hospital so there is a lot of delay. It could have been better if there was a way that samples for sepsis patients are tested quickly for doctors to quickly attend to sepsis patients* |
| 1. **HCWs and patients have experienced blood samples missing especially at the Lab and nurses’ office.** | We learnt that blood sampled miss in a number of ways including just not remembering where the HCWs placed the sample. This could be on the part of the staff in the ward or staff at the lab. | | *Sometimes it happens that they have taken blood for sample from the patient to be tested at the lab, unfortunately they lose the sample and come again to the care givers to tell them that they need to get another blood sample because they have lost the other sample, and it becomes very difficult for them to understand.* |
|  |  |  | *Sometimes it happens that the one who took the sample forgot it somewhere and they remember at a later time.* |
|  |  |  | *Sometimes we would follow it up, go actually to the lab or sometimes we call the lab manager to complain that we have experiencing this, whereby we would send a sample and we expect for the results, there is no result, so on that it’s really a challenge, and the other not really only our lab but sometimes it’s even the blood culture part* |
| 1. **Patients are irritated with repeated taking of blood** | Missing or mishandling of blood samples has several effects including repeated taking of blood samples from the patients. This normally does not go well with both patients and guardians who express not happy with such practices. Such mistakes affects and delays the treatment process. Sometimes there is repeated taking of blood samples (up to 5 times) without explaining to the patients what they are doing with the blood. This raises suspicion of what the intension of the HCWs is with the blood. | | *My brother complained about getting his blood, they have taken blood from him 5 times, by just saying they want to go and test something and it seemed like the person is donating blood.* |
|  |  |  | *It’s not only the delay, sometimes they will tell you there is no sample, and yet you collected the sample and you sent to the lab. They will say there is no sample so that’s really a challenge and I think I was talking to the nurse in the morning in XXXX, there is a patient they have been sending samples, I think they have sent two samples, the first one they say it was clotted, and it was difficult sample yet the amount was small, the second sample was demolished so we couldn’t test so such are the things that it will make difficult for either for the provision of care, for the continuity and even for the diagnosis in case there is other things that need to add on top of maybe the drug that was already prescribed.* |
|  |  |  | *The problem is that it looks like the patient is donating blood, because they come without telling us the reason why they are taking blood samples, what they want to test, and they don’t even give us report of the outcome and it looks like they are not doing anything with the blood samples.* |
| 1. **Unmarked blood sample bottles delays access to right treatment** | Taking of blood samples is also affected when HCW forget to mark the bottles for collecting the samples. This poses a challenge to people at the lab as they don’t know what to do with the sample. Some staff at the lab would just put the sample away there by keeping the patient waiting for nothing. There are some that would follow up with the doctor. | | *Sometimes it happens that the doctor has taken a sample from the patient but has forgotten to mark it blood culture in the book, and on the bottle and then at the lab they just leave it unless if it’s someone who’s willing to ask but in such way the patient keeps waiting.* |
|  |  |  | *Yes, forgetting a sample somewhere up to 3-4 day. Sometimes the sample being sent back from the lab because it was not properly taken, or they didn’t mark properly in the book or file that’s why the results take long to come.* |
| 1. **Factors influencing delays in accessing care** | We also learnt that processes for providing effective care for sepsis patients can be affected by delays in providing the care. Delays slows down the care process which is not in line with the recommended timely treatment of sepsis. We established that delays happen at different levels and for different reasons. It could be from home, as a result of perceived high work load of the HCWs, delays at the primary health care facilities whose initial prescription is usually malaria, delayed referral from the PHC facilities, and long queues at both the PHC level and at the EMERGENCY. We review the factors in the sections below.  Nurses at the EMERGENCY felt that delays are also as a result of long process of handing over a patient from one department to another. When nurses triage a patient, he/she is referred to an EMERGENCY clinician who reviews the patient before referring him/her to medicine clinician. The process could have been shorten if a patient was reviewed by one clinician who subsequently makes a decision for admission. | | *Yes, in the morning delaying to give drugs up to an extent that the patient went alone to get, I saw it happening 3 days and it was these patients who were sleeping on the floor. Maybe they forget to give them but every morning a nurse comes to give patients drugs, I usually find her, but some are always complaining that they have given them late, am not sure if the nurse doesn’t have them or maybe they are special drugs.* |
|  |  |  | *Ok, the thing that made us take so long is that previously he was going to clinics, and he was just receiving pain killers and taking them while at home without being referred to big hospitals so that he gets checked. When he has taken the drugs maybe sometimes he would feel better and think the treatment has worked and days could passed by and some days he couldn’t feel better again and go back to the clinics and then the last day when he was coming here, he also went again to a private hospital, and they gave him treatment without referring him anywhere, and that’s the reason why we went late and the clinics were not even telling him that they are not giving the required treatment he needs to visit big hospitals.* |
|  |  |  | *There is a lot of handing over a patient from one department to another which delays the process. When we triage a sepsis patient, we hand over to EMERGENCY clinician who reviews the patient and hands over to a medicine clinician. This makes the patient to stay for a long time in the EMERGENCY department. If the patient was only seen by one doctor which makes the decision to send the patient to the ward, it could have been quicker* |
|  |  |  | *When we are using EDS (patient registration system at the clerk desk), it may crash at night so for us to call a technician it takes time, so to switch to manual register is not easy like that so a permanent technician is required so that that anything happens he should be there to fix.* |
|  |  |  | *Sometimes they would ignore patients when it comes to giving treatment for example if the patient has explained the problem they would neglect it and start walking around randomly and see patient staying 2 hours without being helped. We had this old man, near myself, and it happened that his pipe used when feeding him needed to be fixed and it was reported to them, but they just said they are coming, and it took them 2 hours.* |
|  |  |  | *When we saw that I was not feeling any better we went to XXXX. At XXXX they sent us back to go at XXXX and at XXXX we were sent back to XXXX that’s when I met research nurses.* |
| 1. **Conditions, processes and beliefs at home may delay care** | Delay in the care process could also be attributed to delay that happen in the homes at community level before deciding to seek health care at either PHC facility or hospital. Participants raised several example of what constitute delays at home and these included: relying of traditional medicine, visiting prayer centres for healing prayers, buying drugs from the drug store on the basis that it was the same drug they could get from the clinic, lack of money to carter for transport costs to the hospital, and just the feeling that they will get better.  Because of such delays, HCWs noted that most patients present to the hospital in a critical situation (especially men), putting pressure on the HCWs for a miracle to happen. | | *The patient stayed for 2 weeks, and I phoned them to find out how the patient was and they said it was getting worse and the patient couldn’t talk properly, and l asked what sort of treatment they are giving the patient they said traditional drugs, and I told to them to bring back the patient here, so that we go to the hospital again and we went to XXXX while it was worse and we were referred here.* |
|  |  |  | *It was last month when it all got serious, but before, he was just having issues with his digestion, sometimes he would get a running stomach from food that would not normally do that to you, that was some years back. Now he started having stools combined with blood clots, so as Malawians we started looking at herbalists and prayer centers but then it got too serious that’s when we went to XXXX* |
|  |  |  | *We went to XXXX dispensary several times but it was the same treatment all the time so we were just buying the drugs from the pharmacies instead of going there and sit on the queue for three hours, drugs like fragile was able to control the running stomach but later he started becoming pale, like he has no blood, we tried to buy some juices, fruits but it didn’t work that when we went back to XXXX dispensary where they immediately referred us to XXXX* |
|  |  |  | *I think I don’t have much to say except maybe that some other patients come in to seek hospital attention or health care while they are in a very critical situation and they expect some miracle to happen for them to recover.* |
|  |  |  | *I told my relatives that I needed to go to a hospital but there was no transport, the next day they managed to get transport and we went to a clinic where they told us to go to XXXX.* |
|  |  |  | *We were just thinking maybe it was going to change, we thought blood was low so I was taking some drugs but there was no change that’s when I decided to go to the hospital* |
|  |  |  | *I had anal inflammation and it gave me hard times because It was producing some water fluids substance, and some said we should try traditional drugs, and I have tried them before and it didn’t work. I could feel fever but when I take drugs I could feel much better just that I was getting weak and that’s why we went to the hospital and when we reached at the hospital……* |
|  |  |  | *I was just weak; I was feeling dizzy in the morning hours. On 3rd December my friend suggested that I was suffering from sugar and he gave me some herbs to try because he had the same problem, this was before I went for sugar checking. After drinking the herbs for two weeks, I met with a certain doctor at XXXX clinic but he doesn’t work there, he works with health organizations, so I asked him about sugar. Just looking at me he also suspected sugar problem, he gave me some signs like frequent urination.*  *I decided to go for testing at the same clinic, I guess because of the herbs they struggled to find any problem with my sugar levels, it showed 113 which is normal according to them. They checked my BP that’s where they found a problem, it was a little high, they admitted me to the short stay area, they gave me a drip of medicine, in one hour the drip was finished, they checked again and they found it at normal at 112/69 so they thought I was scared when I was going to the hospital, they checked again after one hour it was the same. Now they took my blood sample for a full blood count.*  *After doing some tests they realized that I was no eating food which was true, I lost my appetite due to malaria, they also said my blood was low at 9, they gave me two bottles of drugs to help restore blood, they also tested for HIV of which I was found positive, they gave the drugs again and I finished them but the dizziness could not go away.*  *I went for sugar testing at a herbal clinic at round about, the first day it was at 157, I was asked to come again the next day and it was on 234 so they told me my sugar was high, they asked if I was having bad smell in my mouth and I said yes. They encouraged me stay for two days so that they can treat me but I told them I did not have money at the time, I would come back as soon as I got the money. I called the doctor at XXXX the same day and told him my sugar is high so we agreed to meet the next morning, we met the next morning and he advised me to back to the hospital.*  *I went to the hospital and explained everything about the smell and all, they checked again the sugar and it was at 200 but they said they could not start sugar treatment that time so they gave me the same drugs for blood and vitamin B and after a while the smell was gone.*  *After a while I decided to go and get myself checked at XXXX and I took a three days leave from work, I planned to go there on Tuesday but when I woke up in the morning I felt something was wrong, I wanted to write a certain report but I couldn’t get my head together to write anything, my memory was skipping so we started off to XXXX and I thought it would be a short trip but when I reached there they tried to give me blood, they gave 4 pints but I realized that things were getting worse, my memory was not stable, I could say something and forget it right away then I passed out, the next morning around 9 am I woke up in XXXX and I was like where am I, short breath they said*. |
| 1. **Long queues, high patient load and shortage of HCWs, leads to high workload for HCWs and delayed care for patients** | Participants felt that delays is as a result of shortage of HCWs as presented in previous sections), who attends to a large number of people. For the clinicians to effectively provide care to each patient, patients end up staying longer on queues. These long queues are both at PHC and hospital (EMERGENCY) levels | | *Sometimes it’s because we have lots of patients, there is congestion in the hospital and it’s not possible for 1 person to see 20 patients. The HCW get tired. If they could increase number of people working at EMERGENCY, especially those from medicine, it would really be helpful.* |
|  |  |  | *They told me to go to XXXX clinic. They said that’s where they also test other things, but I think they were tired.* |
|  |  |  | *When I arrived there, there were so many people, a lot of them, but after getting the chance to meet the doctors it was all good, they did everything they are supposed to do, run tests because they realized that I needed to be treated right away. It was exactly what expect when they go to the hospital except for the huge crowd. They also sent me to XXXX fast, they didn’t wait long. And also it seems there are few doctors, so it’s not easy for them to handle all these people.* |
|  |  |  | *After seeing one patient they would go out, I wouldn’t know what they were doing. But the only mistake that happened was that they left for lunch around 11am they came back around 3pm, all this time patients were just waiting.* |
| 1. **Initial prescription at primary care level that focus on Malaria testing, delays identification of sepsis.** | We learnt that delays at primary health care level may be facilitated with the focus on Malaria. The initial prescription is always based on malaria testing. When patients are found with malaria, they are given drugs for Malaria and sent back home. When are they not found with Malaria, they are usually given Panadol or Aspirin despite the patient presenting in a poor state of health. This result in the patients harboring sepsis at home and later presenting in the hospital as emergency. Participants suggested developing the capacity of primary facilities to manage sepsis conditions at the clinic level. | | *The patient was complaining about the back, and leg, and they (XXXX hospital) took blood for testing and they said it was malaria but they failed to take water for test from the patients back and we stayed there for 1 week and we came here on 30.* |
|  |  |  | *Alright, the patient started with leg pains, body pains, fever and headache, a week passed and the patient was still going to work, then it went worse and I was phone called and I went there the next day in the morning, and we went to XXXX clinic, and the tests showed nothing was wrong with the patient, the patient did not have malaria and we were sent back without medication, because they also never had the drugs.* |
|  |  |  | *At XXXX hospital they did not give us right treatment, otherwise we wouldn’t have come to XXXX, and they said he was diagnosed with malaria so am sure they were giving him malaria treatment, after seeing that nothing was improving then they referred us to XXXX.* |
|  |  |  | *Last clinic I went was in area 49 in Lilongwe, they gave me pharnsider because they were suspecting malaria but they could not find any, nothing changed though.* |
|  |  |  | *I was taking aspirin but It wasn’t effective, and I went again, found negative again and, on 7th we were sent to XXXX to get a test of Typhoid, and when we go there, they got our blood sample and how it all started.* |
|  |  |  | *Yes, I went to XXXX, they tested Malaria because I had fever, headache, and stomachae, and the results came out negative and I was given aspirin.* |
|  |  |  | *It was in September when I got sick with malaria, I went to a private clinic here in XXXX and they gave me a dosage, after that dosage I felt there was a change but I was not completely ok, towards the end of September and early October I noticed that I was losing weight, because I wasn’t like this.* |
| 1. **There is experience of delayed referral, leading to delayed care – several visits to PHC facilities before referral is made** | Further to developing the capacity of clinics to manage sepsis, participants suggested that clinics should quickly make referrals including having readily available referral facilities including transport. We deduce quick referrals would save lives, same time of the treating clinicians, cut on costs that patients have to incur towards their care and cut on hospital costs of treating critically ill patients. | | *They could have told us that if they will see that it’s not improving then we have to be referred to XXXX the first time and it could have been to us to decide.* |
|  |  |  | *I would like to say that our health centers should know that if a patient has come more than three times with the same problem it’s better to send them to XXXX to have a better assessment before it gets worse, like myself I have gone to XXXX health center countless times with the same problem but they keep on giving me the same drugs, I ‘m afraid it’s getting worse now.* |
|  |  |  | *At XXXX clinic I think that’s where they didn’t help up because the patient couldn’t walk, and they were suppose at least to say they have failed , they don’t have drugs, and maybe they could have sent us to another hospital, but the patient went back home. XXXX clinic they welcomed us well and we didn’t stand on a line and at XXXX we also didn’t spot any problem* |
|  |  |  | *We were given panado and they said she should be drinking lots of water it will end. We were also told that if it continues we should go back. After she started getting the drugs, it wasn’t changing and we saw her eyes turning yellow. We went back to the hospital, and they said the same thing that it happens when someone has given birth recently, and we went back home after being given drugs again and then another day that’s when they said she should meet the senior doctor at the healthy center, and we asked for the doctor, the doctor was in a meeting and we waited, and asked then we asked again, the doctor was gone, we didn’t know where the doctor went, we didn’t know whether the doctor went home or went to do some works somewhere, and we were told to come the next day, we went back without being assisted. We went again the following day and we were told the doctor came but left again, and we met another doctor who tested her urine, and we waited for 1 o’clock to open so that we hear the results, and the doctor said that the results were quiet confusing because he was using small equipment, maybe we should go to XXXX but it seems the problem is the liver and that’s why the eyes were turning yellow and that’s when we went to XXXX.* |
| 1. **Experience with processes of examining and diagnosing patients** | We explored how patients, guardians and HCWs experienced the process of giving care to patients with sepsis including examination and diagnosis processes. Several issues came up and we grouped them into the following key areas: HCWs ability to conduct correct investigations, availability of equipment for conducting the investigations, HCWs having the knowledge and skills of should be done, and willingness among the HCWs to conduct the required procedures.  We will explore these issues in the sections below. However, among other things, patients complained about the painful procedures they undergo in the hospital and yet HCWs have no regard of the pain they are undergoing. HCWs mentioned that one of the most pressing challenges they have to face is being able to conduct investigations, identify the problem, provide the right treatment and yet the patient conditions doesn’t improve | | *The level yes and also that they couldn’t reach to the water level like the first days, because this time around even after inserting a needle to reach to the water level they couldn’t find it.* |
|  |  |  | *The patient was complaining a lot, but we thought maybe the patient would get better because of the treatment, but with too many holes created by needles on his body it was a problem, for example someday a white doctor did the same the on another side, and then another doctor came again did the same thing, the time we were being discharged the patient had wounds despite him being sick and was complaining of pain from these areas but we were told that if we spot any problem apart from that we should report.* |
|  |  |  | *if he is improving you that means we have done our best, but if the patient is not improving despite being given the right medication and right care, and that’s when you think we are missing something, so that’s one of the challenges,* |
|  |  |  | *The first that people always complain is the time it takes doctors to see their relative. They wait only to be seen 2-3 down the line, yet the patient is very sick. Secondly guardians also complain if a doctor do asses them, you know there are some clinicians instead of taking detail history, they will just say, what’s your problem, fever or what, they check the temperature is high, ok ,malaria, without properly examining the patient, so lack of poor assessment.* |
|  |  |  | *From XXXX, we arrived at the emergency building first and then to room 4. After we went to room 4, I was given an injection and some other medicine, after that they were sending me home. Therefore, I told them that even though you are saying I should go home I am not feeling well and I am having some trouble with breathing and vomiting for 2 weeks. After examining me that is when they wrote that they have helped me but I need more help because I had other problems they could not handle that’s when I was admitted.* |
|  |  |  | *They did not help on this, they only stack their fingers inside my anus and after the blister busted, and I told them that I was feeling much better after it busted, and that’s why they said they wanted to check and that’s why they had to stick their fingers and I told them they were hurting me because I was so much pain.* |
| 1. **Comprehensive and right investigations may not be conducted.** | This is an issue that was mainly raised by HCWs. They were worried that the correct and recommended procedures are not conducted when identifying a patient with sepsis. Doctors would rely on physical examination ignoring the standard tests of vital signs like blood pressure, temperature, lactate, respiratory. In some cases, only a few of these would be conducted. There is a challenge of knowing what to do at the right time among clinicians.  Sepsis is usually a condition that is recorded when most doctors are unsure of the condition until test results are out.  Sometimes it is not an issue of conducting the right investigations but not conducting the investigations. Health workers accused their counterpart at the EMERGENCY of not conducting essential tests which are supposed to be done at the EMERGENCY before bringing the patient to the ward. When this happens, treatment is delayed as the ward would have to start all over again i.e. doing blood culture,  There are also times when nurses miss giving drugs to patients. Investigations can be done, diagnosis made, prescription made by a doctor or intern and a nurse would simply forget to give the medication to the patient.  There were situations that even patients were not sure if they were getting the right treatment. This was on the basis even the doctors accepted having inconclusive results from the tests. | | *During the days that I was there, what I saw that didn’t go well is the last process how it happened, it’s like we were receiving treatment of which doctors had doubts over it, and what I would say is they were not sure if the person has TB or not yet they told us that he should start receiving TB treatment.* |
|  |  |  | *I asked the doctor because at first he was saying he thinks it’s 50/50 that it’s TB or not, and they said maybe they test saliva and in the end they didn’t find signs of TB, and then they said they would start giving him TB treatment, myself and my brother asked them what if it in the end it will be found that it wasn’t TB, wont it bring any other complications? And they said the x-ray is showing some signs that they think it can be TB, but if we had tested saliva we would have had proof or if we had found some signs in the water that we tested though from what we have found we believe we can still give TB treatment, and we agreed with them and they gave us the details and I went to get the drugs and the next day we were told that we have to be discharged so that he start taking the drugs at home.* |
|  |  |  | *Most of the patients who are admitted there have Sepsis, because most doctors when they are not sure of what condition is after diagnosis they just write Sepsis. After maybe investigation that’s when they come up with real diagnosis.* |
|  |  |  | *When we say Sepsis, we define as presence of bacteria in blood stream, sometimes Sepsis patient has signs and symptoms of Malaria, but when tested it comes out negative, so sometimes we just write Sepsis.* |
|  |  |  | *Sometimes it’s the medication itself being missed not being given from the word go. They have seen a patient, they have done the investigations, they know the diagnosis, the intern or the doctor seeing the patient has prescribed the medication but either the nurse whosoever is supposed to give hasn’t given the drugs at the right time. Then the patient moves to the ward maybe the nurse at the ward level hasn’t seen that after maybe 24 hours or 12 hours is now say oh we were supposed to get the drugs, let’s give the drug. So sometimes is the delay of doing the right thing at that particular time.* |
|  |  |  | *Management in XXXX is poor according to my assessment, because they don’t do as per expectations, when a patient comes to the ward from EMERGENCY a lot of investigations are not done which were supposed to be done in EMERGENCY and the patients to me they don’t receive the expected care. And sometimes when a patient arrives at the ward from EMERGENCY, she dies an hour or so later.* |
|  |  |  | *The EMERGENCY normally they would attend to a patient and make a diagnosis do their plan but some of their plans are not done at EMERGENCY. For example maybe they would say typhoid fever or typhoid as a diagnosis or malaria as a diagnosis and then they will plan do either blood culture or malaria parasite but some of the things are not done. The patient is sent to ward then after a day or two another intern comes and see that this patient was missed. So we start all over again doing the blood culture, ABCD.* |
|  |  |  | *The first thing is the picking (identifying) the patient that this patient has sepsis. In the past there are a number of ways how you can pick that this patient is likely to have sepsis. Vital signs become very important. Of course these days people have changed, you can pick sepsis patient has got high respiratory at least 22, blood pressure is less than this level, or you also do lactate. In our setting we can only depend on physical examination of the patient than doing vital signs. What I have noted is that, sometimes people don’t do all the vital signs, the may do the blood pressure, but without checking the temperature whether patient has got high or low temperature, so that’s the first challenge, one identifying that this patient has got sepsis using the set standards of picking sepsis patient. Secondly, knowing what to do at the right time, so there are cases where people will just check temperature, without blood pressure.* |
|  |  |  | *They told us to sit on the bench then later we went for vitals tests, they run BP test and many other tests after that we joined a queue to see the doctor, after sometime another doctor came and took us to another room, the only problem I had was the first doctor tried to water from my back, it was painful, he tried three times with no luck but the second doctor did it once and it was successful as well as painless, he also took blood sample. That day we slept there then the next day is when they said we should go to the ward.* |
| 1. **Unavailability of equipment for triaging and examinations results in no or limited diagnosis** | Technical processes of examining and diagnosing a patient would not happen due to lack of functional equipment as reported earlier under amenities of care. We learnt that patients that were referred to district hospitals from clinics, could not be scanned due to non-availability of x-ray machines. At XXXX, other tests like blood culture are affected by having no blood bottles (although research studies are helping in this regard). HCWs would fail to test all vital signs because at times the BP machine would not be working or the thermometer might not be available. | | *They told us that we needed to do x-ray to find out what is wrong with him. Unfortunately a patient who require x-ray is supposed to find their own means of doing it as the hospital does not have such machine. We had two options to go to Mulanje mission hospital or come to XXXX. We came here because we could not afford a private clinic.* |
|  |  |  | *I have seen cases where a patient may come with sepsis but right investigations were not done. They would do full blood count, they may do MRDT or HIV because these things are mostly routine, every patient either they have got sepsis or not you have your HIV tested, MRDT tested. But maybe they may forget to do blood culture, reasons being they will tell you maybe there is no blood culture bottle. Of course these days there is some improvement because we do have research studies that are doing blood culture like Wellcome Trust but there are cases maybe during weekend or at night when research staff are not there, so you need to look for blood culture bottle, you’ll find that a patient was sent to the ward but without doing blood culture.* |
|  |  |  | *Despite knowing the standards, one could not carry out the required measurements because it could be that they don’t have a working BP machine, or maybe they don’t have a thermometer, so you see that maybe one vital sign will checked the other one not.* |
| 1. **Lack of Knowledge and expertise among HCWs of what should be done in the examination and diagnosis processes** | Proper examinations and diagnosis is also affected by lack of knowledge among the HCWs of what should be done. Doctors have experienced situations where certain important vital signs are missing in patients’ health passport books. Patients and guardians felt that trainees require a lot of support and supervision as they lack confidence in their work, as such they also do not have confidence in them | | *They just have to add more permanent doctors because they are using trainees most of the time without supervision. So a very sick patient they may hesitate to do something for the fear of causing more problems to the patient. At least one experienced doctor to guide them. You can easily tell that this is a trainee. You can see how they are behaving, it’s easy to know. Even my patient could tell me.* |
|  |  |  | *Normally it concerns nurses or clinicians, for example we can register a patient in the system (data clerk), they go at triage they are registered again but the clinician may forget to do the same so the system will have incomplete data* |
|  |  |  | *It’s just lack of knowledge of what you are supposed to do when you are examining a patient. Vital signs are supposed to be on each and every patient regardless of what the cause of their illness, be it sepsis or not. Vital signs are important but if you check on patients healthy passport whatever you’ll see that most of the times may not be complete for various reasons as I said maybe the equipment to do that they don’t have. So if the patient has not been properly assessed it means you’ll have problems on how to manage the patient. There are some patients that have got sepsis yet there blood pressure is ok, that patient though may need fluids, but it will be different in someone who has got very low blood pressure, that one will need the IV fluids and it’s a must that they have to get IV fluids, without the IV fluids it’s definitely that the outcome will not be that good, so that’s one problem,* |
|  |  |  | *The only bad experience I had with a staff is when a trainee failed to inject me with some drug. A lot of blood came out and he injected my mum with the same syringe, he was rude when we asked him why he did that. I told another doctor about this, she took her for testing and gave her pep to take for thirty days.* |
| 1. **There is perceived unwillingness among HCWs to carry out the recommended diagnosis processes** | This was particularly one area some patient experienced as a challenge. One guardian had the worst experience. When they arrived at night, the nurse at the EMERGENCY left them at the short stay ward and was unwilling to help them. They made several attempts to call the nurse but did not turn up until they informed another nurses in the morning. During that whole period they were in EMERGENCY, both the patient and guardian had not eaten/ were not given food. She believed that if the patient was attended to as quickly as possible, she could have been discharged the following day but the condition worsened and they were referred to ICU. | | *Another thing about infection prevention for example cannulas are supposed to be in, three days’ maximum but people don’t change them unless the patient is messed up then the nurses will follow that through. And I have seen a patient die, they remove all they luggage and they put another patient and the bed is not even cleaned.* |
|  |  |  | *Guardians also complain if a doctor do asses them, you know there are some clinicians instead of taking detail history, they will just say, what’s your problem, aa no am feeling, fever or what, they check the temperature is high, ok ,malaria, without properly examining the patient. So poor assessment.* |
|  |  |  | *The one I have noticed is identifying a patient who is very sick and who is not, because I have seen people who are supposed to be in the resuscitation room or XXXX who are not, so it’s the triage point. In the emergency room there is a triage where you have to filter patients, one who go on the queue, on who can go to priority or one who can go to the emergency. So the one who can go to the resuscitation area needs to be seen immediately, the one who goes to priority can be seen within the hour and the one on the queue can be seen within four hours. But we usually don’t wait up until four hours otherwise we will have a backlog of patients so they’re rapidly turned over to clear the bench otherwise you will not sleep at night because of queues. So the problem is that I have seen patients who sometimes even die who should have been resuscitated in the resuscitation area because someone didn’t triage them properly* |
| 1. **Challenges in processes of identifying patients** |  | |  |
| 1. **Perceived high workload among HCWs lead to missing of patients/ patients falling in the poops of care** | Participants expressed two varying experiences on how high workload/ shortage of HCWs results in patients being missed in the process of giving care. One scenario concerned high workload at the EMERGENCY during the day than the night. During the day, the emergency department has a lot of patients and some might be missed. At night, HCWs have enough time to concentrate because there are usually very few patients. Unlike in the ward, there are fewer staff to take care of the same number of patients and some patients are missed. Sometimes, HCWs working at night simply show unwillingness to help. Participants recommended increasing the number of HCWs at night in the wards and during the day in the EMERGENCY. | | *Yes, that’s why I was complaining about medication because we call someone to come help us its hard, they usually go to give drugs all around and for them to get back to us it take time and I follow them while it’s already late. So I think during night hours they should add more people, so that when some are giving drugs some should be treating us* |
|  |  |  | *I think workload is what affects, makes people to be missed, because at night that rarely happens, so at night there are less patients and triage works very well, in my opinion. During the day the emergency department is packed so some patients fall through the loops, but at night it works perfectly, there’s time to concentrate* |
| 1. **Nursing students ignoring challenges and procedures experienced when triaging patients** | The issue of students/ trainees is one that came up from both patients and doctors. Doctors have experienced challenges with trainees e.g. nursing students bringing up wrong measurements during triaging or failing to identify and prioritize critically ill patients. Earlier, we expressed how patients experienced problems with procedures performed by trainees | | *The other thing on the triage is that they like putting students there, they will do all the necessary things but some of them will not understand that this patient is very sick and needs urgent attention, the more experienced nurses will look for the doctor personally and say doctor, there is patient who is very sick in this cubicle, I have this, this, this but I am worried please come and see this patient.* |
|  |  |  | *Some nursing students would not want to approach a doctor. The medical ones will pressure you until you do what they want you to do. The nursing ones I don’t know if they are not really sure who they should talk to. I have noticed that sometimes vital signs are taken by student nurses and they were abnormal and they didn’t tell anyone until you find the patient* |
| 1. **Out of hours investigation are not efficient.** |  | |  |
| 1. **Investigation done during out of hours are delayed due to lab closure of the lab and unwillingness among HCWs** | Blood culture is one of the key investigations in identifying and giving correct treatment (antibiotics) to patients with sepsis. Unfortunately there are delays in currently processes. Staff are either not willing or are overwhelmed to do blood culture during the night. During the day, they are supported by staff that are involved in research studies. Results from blood culture usually come out after 72 hours. During this period clinicians are not sure if they are giving the correct treatment. This delays care in case there is need to change treatment based on blood culture results. | | *The other one is blood culture, after 5 o’clock people are not happy to do a blood culture, that’s what I think. During the day its easier as I said there are two competing studies plus the government workers so there are three groups of people who can do blood culture, at night it is just the government staff working at night and they can be overwhelmed because of the workload, so that’s the whole process of doing blood culture, sometimes it doesn’t happen but most of the times it will happen.* |
|  |  |  | *Unfortunately, blood cultures even if you take them correctly, they will not always show you what bacteria it is but they do help a lot in terms of exactly which bacteria it is and what type of antibiotic you should use because they give you sensitivity pattern but that result comes out pretty late, if you are lucky it will come back in 72 hours, but it usually comes out very late probably by this time you already realized that the antibiotic you gave them is not working and you have switched to something else. So that’s the experience if you are working in the ward, if you see a patient who has fever and you don’t really know what’s happening and the antibiotic you have given them is not working you switch.* |
| 1. **Factors affected effective processes of monitoring patients’ progress** | Patient monitoring in this regard refers to as the continuous care and support given to the patient from arrival to departure of the hospital. We learnt that consistent monitoring of patients was in various ways and affected by a number of factors. Patients experienced delay in changing of medical materials i.e. cannulas catheters, patients; files going missing in the hospital, lack to following up of processes that have been initiated, Lack of equipment to aid patient monitoring, and nurses’ non-compliance to prescription | | *Urinary catheter that they installed brought some problems they did not inform us about, every time the patient passes urine blood comes along, this is still happening even though the catheter was removed, they should inform us on possible side effects.* |
|  |  |  | *Sometimes it’s possible that a patient has called for help from the nurse and the nurse won’t show up, they go to hide, then I go to the patient to explain to them not to worry the nurse will come and also ask them what they want maybe if it’s drugs then I go to tell the nurse despite some running away from their duties.* |
|  |  |  | *The clerks who work in the wards do not work night shift so they usually miss on patients who come at night and then when making reports we have incomplete data therefore we give wrong statistics.* |
|  |  |  | *It’s crowded, maybe there only few nurses, maybe one or two doctors to take care of all the patients. This is EMERGENCY team only, but these are the guys that direct the patients, and you need surgical opinion. So if they are overwhelmed they are only few people, it’s difficult to monitor what is initiated on a patient i.e. Septic patient. There could just be inadequate knowledge that a patient with a septic this is how we are supposed to monitor. They think that IV fluids is enough but you need to monitor the fluids that you have given are adequate enough for this particular patient. The same thing happens when they come to the wards, the monitoring is not that adequate. The doctor may prescribe, ok this patient give them a bolas of IV fluids and maybe after this bolas I need to continue with maybe 3 litres 24 hours. He will come their the following day and find that the patient only received that 1 litre that was put in EMERGENCY the reason is maybe there is no one to continue with monitoring the patient and sometimes very understandable maybe at night there are only 3 or two nurses they need to take care of ward which has got 70 patients. So at the end of the day instead of doing the other stuff the only thing that they can do is to administer drugs to patients, therefore that thing of monitoring blood pressure it’s not possible, because there are only two nurses at night.* |
| 1. **Delayed/ ignoring timely changing of materials i.e. cannulas and catheters** | Use of cannulas was one example that commonly came up among patients, guardians and HCWs that monitoring use is not given the attention it deserves. The following examples points how cannulas stay on patients beyond the recommended period and left unattended for long periods of time. | | *We also had an issue with the thing they use to inject medicines on his arm, it could fall off now and then and the nurses will be shouting as if the patient was taking it off deliberately.* |
|  |  |  | *So in the emergency department a patient can vomit, and the vomit can stay there for 2 hours without someone cleaning until I go tell them to go and clean it. The mattresses are supposed to have a plastic cover but quite a number of them are torn so the flex foam absorbs all that. Just on my last call I sat on a mattress that looked clean I saw fluid coming from the flex foam and I felt wet and I was sure it was urine, I had to go home change. Another thing about infection prevention for example cannulas are supposed to be in three days’ maximum but people don’t change them unless the patient is messed up then the nurses will follow that through. And I have seen a patient die, they remove all katundu and they put another patient and the bed is not even cleaned.* |
|  |  |  | *When they are giving blood or water to patients using drip, they don’t come in time close it when it is full as a result the blood starts to come back into the drip.* |
| 1. **Patients files may go missing in the hospital** | Although there was a single patient that expressed that his clinical documents were misplaced/ went missing in the hospital, HCWs reported that this was a frequent occurrence in the hospital that happen especially with medical students who sometimes take with them the files to write case reports. However, this is improving as they are required to sign log books upon taking a file. This is a cause for concern as missing of patient clinical information would pose a challenge for continuing care. | | *I did not come with the patient on this day but when they arrived they gave him treatment, first diagnosis showed that there was water in his left rib, they tried to get it out afterwards they gave him a drip of water, after that drip they admitted us to the main ward and they said he needs to go to x-ray. Next day we failed to go to x-ray because the relevant document was misplaced, we had to process again, the day came for x-ray, we went there and they helped us, by this I was here, we asked for results but they said the results will come with the doctors.* |
|  |  |  | *Sometimes patient files will go missing in the wards after doctors, especially students have misplaced them, it disturbs our work flow but now we have a log book for any doctor taking a file to sign* |
| 1. **Lack of following up to initiated processes among HCWs and nurses noncompliance to prescription.** | Effective patient monitoring can be affected by HCWs’ inability to follow up clinical processes and procedures which they have initiated. This is also related to the issue of patients falling out in the loops of care. Participants raised cases of following up with nurses several times to check in IV fluids that have run out and only to come hours later; not receiving the drugs that a doctor has prescribed, and starting a process and only to come hours later to continue. However, HCWs felt that forgetting/ taking a long time to follow ups on an initiated process might be as a result of high workload where a HCW is attending to several patients at once. | | *It was reaching a point that we could search for them and sometimes we could argue, and they would say am rude, but the problem was that they would forget us at times but the relationship improved with time, and the problem as I have said sometimes they would put the drip and the water would finish but they would forget to remove the drip tube.* |
|  |  |  | *Because the problem is that we are treated by different doctors. Someone would start it but it would end up with someone else who has a different view from the first one, instead of finishing it alone.* |
|  |  |  | *Monitoring of patients that come with sepsis, they are very sick. As I said there are guidelines for patients with sepsis, and has septic shock, you need to give this much of fluids, within this time of period with a target of achieving this level of blood pressure. You’ll find that a nurse or physician that they may start with IV fluids, but they can titrate or they will just open it, but they will not come and see whatever we did, has it made a difference on blood pressure or not? Do I need to do something or not? There are several reasons, one of the things is possibly the staff, which we don’t have adequate number of nurses, doctors to take care of our patients. If you go to EMERGENCY ,sometimes, especially Mondays, its overwhelming* |
|  |  |  | *The drug might be there but sometimes the problems is the frequency of giving drugs i.e. 6 hourly. There are other nurses who don’t give according to the prescription time so it’s the attitude part now because the 6 hourly is 6-12 6-12, or 10-2, 0r 10-6 . Roughly it’s 6 hourly. The gap of 4,10,2,4 like that, some would think that aaaa, 12 midnight maybe they were resting , waking up go give the drug, so on that part it also affect the management because likely the patient will not get the medications required. When we are giving the drug we also consider that on the half-life that when the drug is in the system when it ends another drug has to come in. Regarding that it’s a challenge so the attitude part to me it’s something very difficult to say that you can end it. I would give you an example - last week there was a patient who needed mobiliser for hourly, some of the nurses were doing that but some others were not. Despite talking every day you would hear the doctor complain the patient did not get the drug, why? We forgot and yet the handover was Cleary written and they did the other things except for this patient with 4 hourly mobilize. You mean you didn’t see? So on that part I think it’s a challenge.* |
|  |  |  | *Guardians have also come to us to complain about IV fluids, either the drip finished long time ago but no one has come to replace it and guardians will always come, doc his drip finished some hours ago but we tried to complain to nurses or clinicians, but they are not coming, they are not coming, they not helping us in any way or drugs. If the drugs are missed they always tell you that, doctor prescribed this drug yesterday, received an injection, but today has not received any injection, we ask the nurses or the doctors they say the medication is not available, is out of stock so it means that day is missed.* |
|  |  |  | *I only remember the day before I was discharged I asked a nurse to look at my cannula as it was not sitting properly. She checked it and found that it was out of its place, it was not connected with the veins as intended so the drugs were not going in, she removed it but she never came back. The following day when other nurses came, they thought I removed it myself but I told them it was another nurse who took it, they insisted though they fixed another one and told me to take care of it.* |
|  |  |  | *I think they should be able to tell patients why they are being kept in the hospital, I am saying this because when they started treating me on Tuesday then Wednesday was a public holiday so they continued on Thursday afternoon, when the doctors go away while the process is still in progress they take too much time to come back then you start to wonder what is going on.*  *A patient who is much better is being kept in the hospital without any explanation. The day I was discharged I was scanned around 11am but the doctor could not finish writing in the file because he received a call to attend a meeting, so he told us to wait until he comes back in the afternoon to finish file and discharge us. When he came back around 4:30 another doctor had already helped with the file and instructed us to go get drugs from the pharmacy.* |
| 1. **Patient monitoring may be affected by lack of equipment to aid the monitoring processes** | Patients monitoring is also affected by lack for equipment that specifically are key to monitoring functioning of organs in sepsis patients. Such equipment which is not available at XXXX include Arterial Blood Gas. | | *Lastly on the investigations you may want to say, ok, a patient who has got sepsis, it’s important to monitor renal function. Here at XXXX currently we can only do urea, we cannot do the other side, and we can only do urea. Patient with sepsis we might want to do Arterial Blood Gas, but because we can’t do that.* |
|  |  |  | *We don’t have that type of equipment but Arterial l blood Gas very important to patients that are septic. It helps to decide that maybe am not giving adequate fluids or but also its one of the monitors that you use came in with acute kidney injury because of sepsis. You are giving treatment, antibiotics, fluids or whatever you would want to know, is the renal functioning improving or not? But at XXXX you can’t do that. So there are challenges at multiple levels, but at the same time it does not mean that every patient that comes here does not have a good outcome and majority they come in, we treat them, they go home but we do have some of those challenges in our setting.* |
| 1. **Patients’ experience of processes at PHC facilities were largely unsatisfactory** | In exploring sepsis patients’ pathway to care, we recognized that the primary health facility is the initial important entry into the pathway before referral is district or XXXX is made, hence forming part of the car delivery. We asked patients and guardians of their experience with PHC facilities and how that experience affected their care. The following statements highlight some of the positive issues and issues that require improvement in the care of sepsis patients at PHC level. Among them include the need for: Going to the health centers several times before referral is made – the facilities (regardless of no proper diagnosis sometimes takes time to make referral. Sometimes patients literally asked the clinicians at PHC facilities to write them referral letters; Since there is not proper diagnosis, patients are most given Panadol for long periods of time, and in some cases, they are not given any medication; Such medication is only given on assumption as no examinations are conducted; Lack of equipment for testing and the common test that is performed at PHC level is for malaria; inability of HCWs at the PHC to identify and prioritize very sick patients; Patient communication with HCWs differed from one HCW to another with others being welcoming than others. This gave the impression that it was a reflection of individuals than the system; PHC facilities have few staff and patients waits for long periods of time when a clinician goes out; lack of ambulances for transporting the patients once referral is made; toilets that are not clean and perceived favoritism  Participants wished if they go to clinics, as a sick person, they have to run some tests before giving any medication. A patient might show some signs but if they don’t examine the patient they may end up missing the underlying cause.  However, there seem that PHC facilities that are urban based like XXXX and XXXX, had better facilities, presences of doctors and equipment. They are able to conduct preliminary test and make quick referral decisions. Patients did not want to put any blame of PHC facilities as they understood that whatever they do, is according to their level of resources and capabilities. | | *It was end June, we had gone there several times but we couldn’t get proper help until one day I realized that her eyes were becoming yellow so we went to a private clinic but they also said it was normal for women who just gave birth but I wasn’t convinced so we went back to the health center, then the doctor took urine sample that’s when he realized something was wrong and referred us to XXXX.* |
|  |  |  | *She was pregnant, after giving birth we stayed for a week and I thought she was better and I was about to go back where I stay then she started complaining backache, we went to XXXX health center, they said it was normal for new mothers to feel pain in the back it will pass, they gave us panado and we went home. There was no change and she started complaining about the stomach as well so we went back, we explained everything and they told us to wait for the doctor to see us but he was in a meeting by then so we waited and the we asked again the doctor was gone and we were told to come again the next day.* |
|  |  |  | *When we got at XXXX we were helped, the patient couldn’t move so we had to hold the patient. We met a doctor and went straight to the room, we were instructed that the patient needs blood test, and the patient was given injection and told us to rush to XXXX.* |
|  |  |  | *Then it reached a point where the patient would just lay down and not even going to the office. Then I went with the patient to XXXX clinic and was found without malaria but they didn’t have drugs despite not finding any infection and we went back.* |
|  |  |  | *I was not at the health center but still they did a good job by quickly sending him here because they knew they could not manage despite that it is a mission private clinic.* |
|  |  |  | *. His friends carried him in a wheelbarrow to the clinic, I rushed there and called one of the doctors to help him, the doctor came and treated him until he started opening his eyes, moving his body and then they told us to get ready to go to XXXX. We waited for some hours for the ambulance and finally we arrived here around 4pm (he was a HCW at the clinic), they checked him and sent him to XXXX for examination and they found that one side was damaged. They also examined his chest only to find that he has TB. Now there is an improvement* |
|  |  |  | *At the health center they really helped us, he was given treatment to regain his conscious, they also advised him to stop his habit of sleeping with different women, drinking and smoking because this is a third time he has been diagnosed with TB* |
|  |  |  | *If we say we were not satisfied with the health center, that would be harsh because they acted according to their abilities, they didn’t want to waste time on assumptions* |
|  |  |  | *At XXXX clinic I think that’s where they didn’t help up because the patient couldn’t walk, and they were suppose at least to say they have failed, they don’t have drugs, and maybe they could have sent us to another hospital, but the patient went back home. XXXX clinic they welcomed us well and we didn’t stand on a queue.* |
|  |  |  | *When we got there, we were not well welcomed because as doctor with experience you would actually know that the person is severe sick, and this one is at least better so maybe if I could start helping the one severe sick, and if it was me I could have seen that the doctors treatment was delayed and the doctor was rude, I didn’t know what they were thinking about up to an extent that the patient almost felt from the bed.* |
|  |  |  | *We are very thankful to the staff of the clinics, they were able to help us until they referred us to XXXX, without them we would just be buying antibiotics from the stores, there was a time when our patient could not move and the doctor from the clinic followed him home, he showed that he cared.* |
|  |  |  | *I would say there is a good relationship but sometimes they are moody as if they had a an argument with their wife, and when they come here and if the patient is doing something they don’t like, they can easily shout at them but sometime would talk to the patient nicely and it just depends with the mood of the person on that day. You would be welcomed nicely sometimes, they ask you questions of what you are suffering from and write them down while sometimes others will just write down and give you the file even before you have not finished explaining.* |
|  |  |  | *Most of the times they don’t have drugs, if you have gone with a problem, drugs like Bactrim you have to buy alone and sometimes we would go to XXXX, sometimes at XXXX, at least there they do help, XXXX and XXXX they help but at XXXX we go because we don’t have transport and they have so many problems* |
|  |  |  | *The number of staff is small especially the cleaners, what happens is that when a patient vomits on the flow these cleaners will not do anything until the next time they are supposed to clean again, if they clean in the morning, they will wait for 12 noon to clean again and after 5pm, so if a patient mess up the flow it will stay like that until it’s time to clean again.* |
|  |  |  | *Hygiene, the place is too small but the toilets are not clean, cleaners are few I think. The toilets are not clean; you can’t even help yourself in a toilet that is making you uncomfortable. I once got in the toilet but I came back without using it, it was bad.* |
|  |  |  | *Another issue, it’s not good that they should be giving prescriptions on assumptions, at XXXX they were able to follow me properly.* |
|  |  |  | *The clinic is always packed, like more than XXXX and the people working there are just few, if you can go now then you will be coming back around 2 or 3 pm. Because of the number of patients waiting to be assisted, there no time for them to be explaining everything to patients* |
|  |  |  | *When I arrived there, there were so many people, a lot of them, but after getting the chance to meet the doctors it was all good, they did everything they are supposed to do, run tests because they realized that I needed to be treated right away. It was exactly what expect when they go to the hospital except for the huge crowd. They also sent me to XXXX fast, they didn’t wait long.* |
|  |  |  | *I would say the treatment they give to patients, because it seems like it’s a challenge, sometimes the doctor come late and sometimes bribery is involved, some people it happens that they don’t get on the queue, they just go straight inside leaving other people behind on the line.* |
|  |  |  | *We were welcomed well at XXXX, but the care we were given, hahaha, especially the drugs it was a problem, we were given aspirin, and once I took it would work but when it starts working, the pain would come back.* |
|  |  |  | *The only problem we have there is when a patient is very sick and is referred to come here most people struggle to find transport, I think transport is necessary because this clinic cannot manage some patients so they send them here, so many people are poor they find it very hard.* |
|  |  |  | *Another thing is time, you might go there around 8 and you will get treated around 11. It happened when I went there* |
| 1. **Patients were emotionally traumatized with frequent deaths in the wards** | We learnt that frequent deaths that occurred in the wards caused trauma, anxiety, fear and panic. Patients felt such experiences worsens their conditions. We had experiences of some patients that run away from wards due to frequent deaths. Participants raised several recommendations to address this including having adequate space in between the beds and quickly removing the dead body to the specific preparation room without any announcement. HCW explained that relatives are engaged on the condition of the patient when it becomes worse in order to prepare them. When death occurs, they prepare the body before send to the mortuary.  Patients and guardians complained of frequent deaths in the ward. Here are some concerns that participants raised as a result of frequent deaths: patients refusing to be put on oxygen since they have since some people dying on oxygen; Patients conditions would worsen, feeling hot and BP elevating. | | *It happens like that most times, people are told in advance so that they should be prepared, because it clearly shows how difficult it is for the patient to survive. If the patient dies we write in a book, and people should also know what condition is taking many lives of people, then we clean the body, and we write the condition that has lead the patient to death then you take the body to mortuary* |
|  |  |  | *Ok, sometimes it shows that despite our hard work to save the patient’s life, it will be difficult not to lose them, then we explain to the guardians that we should expect any outcome.* |
|  |  |  | *When death occurs we do have screens, before having screens we confirm, we assess the patient and when we see that all the parameters do not give us results then we confirm death to the right person then we cover with screens to lead the guardians out. Now we have death certificate, they provide us with the deceased information so that they can use to claim death certificate at DC’s office. We prepare the body and take to the mortuary. Guardians are asked if they want to use main mortuary or college of medicine. When we get to the mortuary we document everything and show them where the body is, if they were referred from a district hospital we call that particular district to send an ambulance to collect the body.* |
|  |  |  | *When a patient has passed, we don’t just carry the body, we wait for the doctor’s confirmation, then we call the guardians in our office and explain to them, then we clean the body and take it to mortuary but the guardians are explained in a proper way so that they should accept it.* |
|  |  |  | *I can say both, people die in that ward every day, maybe four people a day. Others not very sick, just having breathing issues, two minutes on oxygen he is gone, someone who was walking by himself five minutes ago dies like that.* |
|  |  |  | *If they see people dying they always think of escorting the dead to mortuary and they think they may be the next person to die and then they want to be discharged* |
|  |  |  | *It was the noise of the guardians crying that was disturbing me and looking at the deceased person as well. My mother was covering me so that I should not see* |
|  |  |  | *Yes, it’s a challenge, but when death occurred in our ward, I would be so afraid that I wanted my mother to sleep on the bed with me, looking at each other all night.* |
|  |  |  | *When a patient dies at the EMERGENCY, the clinician who is available confirms and fill the death report and the body is taken to the mortuary. The problem I see is that there is no grieving space. It could have been better if there was space for the guardians to wait because there is a lot of noise in the whole EMERGENCY. This attracts attention of everyone in the EMERGENCY* |
| 1. **Patients deplored long periods of waiting time** | Most patients and guardians complained about the amount of time they wait to be assisted – they always would like to be treated in the shortest time possible. Participants complained about long waiting time from the primary care facilities to the hospital. In this section we explore the issues of workload, approaches to work and how they are associated with long waiting times; long waiting times at EMERGENCY and PHC levels and triage and consultation times. | |  |
| 1. **Mixed views on whether long waiting time is as a result of approaches to work or high workload** | Some patients and guardians had contrary views to HCWs who felt that long queues and long waiting times are as a result of high patient load in the hospital. Some participants expressed that even with fewer people on the queue, HCWs would take a long time to attend to patients. This raised questions of whether it is really related to high workload of poor attitude and approaches to work. | | *I don’t think there was a queue, but I can’t tell why they took time, when they came they asked how it started and then they took a blood sample for tests, they came back with an injection* |
|  |  |  | *The only problem I saw at EMERGENCY is that they take long to check on a patient. They have to check patients as soon as they arrive to see if there’s need for urgency* |
|  |  |  | *And the other issue is the conduct of staff, most of the times are guilty of negligence, they always make unnecessary delays when a patient is calling for help* |
|  |  |  | *Another problem is about staff not being committed to their work. When a patient arrives at night, nurses will just sit down and not attend to the patient as urgently as possible. A lot of people complain about our department (EMERGENCY) because of delays. When patients look at us (clerks) they feel like we are not helping them and yet that is not our job.* |
| 1. **EMERGENCY and PHC are the areas where patients wait the longest** | Of all the processes along the sepsis patient care pathway, respondents complained of long waiting times especially at the EMERGENCY and PHC clinics. Patients normally have quick triage at EMERGENCY but it takes a long time to be seen by a doctor (consultation). Sometimes it is understandable that there are only 2 doctors while in some cases. Although HCWs expressed that they prioritize and attend patients according to the severity of their condition, this was contrary to what patients said. They wished HCWs at EMERGENCY could firstly attend to patients are very sick. Patients had similar experiences in PHC clinics where clinics usually open at around 9am when patient arrive at around 5am. However, this was not a common experience among all patients. Some had positive experience with HCWs attending to them urgently.  HCWs expressed that they normally have to attended to large numbers of patients that the required 3-5 patients per nurse. Usually there are also 2 clinicians attending to long queues | | *At this point it’s not clear whether the doctors are busy with other patients in the cubicles or not, but there may be few doctors working so they have a lot of patients to look at.* |
|  |  |  | *The only thing I would like to see change is the time we take waiting to see a doctor reduced. You will find that there are only two doctors and a lot of patients waiting on them.* |
|  |  |  | *At EMERGENCY what happens is that they will register you very quickly and take the tests then they tell you to go and sit on a chair where a doctor will come to see you, then you wait until you get tired while the patient is in pain. It may take up to 3 hours* |
|  |  |  | *We didn’t wait for so long, we gave them the hospital passport book, and we were told to wait, and it didn’t take long they took the patient to the bed, and everything that was happening, was happening while the patient was being assisted.* |
|  |  |  | *What I want to change is that they should see the condition of the patient who has come, because some maybe they just have headache, and someone is heavily groaning and staying long without being helped, we tend to get worried that maybe something might happen, but this is a big hospital they should have many people to assist us in different ways.* |
|  |  |  | *When we rush to the hospital with a patient, we expect treatment to come right away, but often times we don’t know what is required in order to properly manage the patient but them nurses know what to do and when to do it.* |
|  |  |  | *First thing, I heard that those working in government they start work 7:30, but at the clinic we go around 5 o’clock and they open around 8 , and for them to start working it might be 9 or 10, so we waste time struggling to wait for nurses.* |
|  |  |  | *We were given a bed at short stay, and we were helped quickly after your group came (ARCS study) but otherwise we were just given a bed and waiting for a doctor. When your group came they asked if we could join the research programme, and she said yes. The first doctor said she should be given drugs, and then your nurses were the ones who gave her the drugs and injection, and then another patient beside asked me why they were not being assisted, and I said maybe because our patient entered the research programme and they want to assist her quickly.* |
|  |  |  | *We reached XXXX trading and boarded a bus to XXXX district. When we got at XXXX hospital it was around past 4 and we were assisted but after some time, because those who were working during the day were about to knock off, and those on night shift were about to start, and only one person showed up and the process was being delayed, from past 4, we were assisted around 7.* |
|  |  |  | *They need to improve on time they take to treat patients. When we arrived registration and checking of vitals was quick but waiting to see the doctor now was a problem, we waited almost two hours, but once they started seeing him around 1pm it was a fast process until they moved him to XXXX ward.* |
|  |  |  | *On the first trip, we met the doctor very late, as I said that we got around 12 noon but we met the doctor after 4pm, and he took his time as well to understand how it all started, he took samples for testing while we were waiting for a bed in the ward. The second trip we arrived after 9am, after taking the vitals they found that my temperature was high so I was given Panadol, after that I was given another dose after 2pm while I was still waiting to see the doctor. The doctor came after 3pm, I slept and I was shaking so he asked what was wrong and he left. Another doctor came, he started the treatment process then he left and we waited again, when he was coming back it was after 4pm so he handed over to another doctor who started the whole process again then he admitted me.* |
|  |  |  | *When I arrived there, there were so many people, a lot of them, but after getting the chance to meet the doctors it was all good, they did everything they are supposed to do, run tests because they realized that I needed to be treated right away. It was exactly what expect when they go to the hospital except for the huge crowd. They also sent me to XXXX fast, they didn’t wait long. And also it seems there are few doctors, so it’s not easy for them to handle all these people.* |
|  |  |  | *Yes, they just didn’t pay attention that patients are in pain they have to be treated fast. They were just slow* |
|  |  |  | *They were moving a lot, after seeing one patient they would go out, I wouldn’t know what they were doing. But the only mistake that happened was that they left for lunch around 11am they came back around 3pm, all this time patient were just waiting.* |
|  |  |  | *We got there around 10am but the treatment begun after 4pm, we were told to join a queue and then they said we should go outside to another area (XXXX clinic) where we will get treatment but my mother said no, we have been here since morning and you want us to go there and join another queue? Then we met the team doing the research and they helped us. Yes, that’s when they started to say we should go to XXXX but for us it was not practical, seeing how late it was at 4pm that’s when they handed us over to the research people* |
|  |  |  | *Normally one nurse is supposed to attend to 3 to 5 patients. Due to shortage of HCWs, you are usually 2 nurses at the triage. You have to test the patients, enter their details in the computer. Then you have 2 nurses in the cubicles. So we are very few of us. Clinicians as well might be just 2. So even though a priority patient is supposed to be seen within one hour, the condition starts worsening while on the queue. Those that are supposed to be seen within 2 hours, can stay up to 4 hours. Some give up and return home* |
| **The pathway for sepsis patients – what health care workers know** | HCW participants provided descriptions of the pathways to care for patients with sepsis. In this section, we provide what would be seen as a typical pathway of sepsis patients from a perspective of a HCW. We further explore what happens at each stage of the pathway, and whether improvements need to happen.  A patient is usually referred from a health centre, a district hospital or might decide to come straight to XXXX. On arrival at the EMERGENCY, they first register at the clerks’ counter. They are then checked vital signs at the triage when nurses sort out the patients – those that are very sick are prioritized. At the EMERGENCY they are assessed by either a medical intern of medical clinician.  In case of anything suspicious, there are some tests that are ordered like blood culture, full blood count or x-rays. Sometimes sepsis is confused with Malaria because they have similar signs and symptoms. So Malaria Test is also conducted - MRDT or Microscopic. If the test is negative and microscopy is showing no sign of Malaria then they take other test like blood culture and sometimes they can take sample for blood peripheral and if it’s in severe cases, they conduct LP for cell analysis, central cell side analysis, to check if it’s not meningitis and then drugs are ordered. If it’s a severe case, the patient is kept at the EMERGENCY in the short stay ward to stabilize. If it’s a severe case the patient is kept at the resuscitation room at the EMERGENCY  When a patient arrives at the ward, he/she is given a bed and if those tests were not ordered at the EMERGENCY, then blood tests is taken before being give medication. They assess the patient that is in the ward, and then if there is a need of IV fluids, they are given IV fluids. If the patient is having high grade fever they manage the high grade fever, and then give the patient prescribed drugs. Is the patient’s conditions does not improve, he/she is taken to ICU or XXXX. Sepsis are referred to XXXX or ICU when they are having difficulties in breathing.  The patient is brought to either ward XXXX or XXXX. There are demarcations in the wards according to condition of the patients. The first bay is for patients with malaria, diabetes, anemia; the second bay is for patients with meningitis and other conditions or patient suspected of having TB. The third bay admits patients with the presenting complaints which likely might be TB. While the XXXX is for patients who need close monitoring on oxygen. Because of lack of space, sometimes, patient who requires XXXX are kept at the first bay to be seen more frequently as it is a close bay to the nursing station.  In the medical wards XXXX and XXXX, there is a consultant who comes on Mondays and Thursdays. Tuesday, Wednesday and Fridays or Saturday and Sunday are meant for interns either on call or on that particular department. They do ward rounds, discharge patients who might have waited for 2 to 3 days for their results to come in or they are asking for a discharge they feel they are better. During ward rounds they discuss, teach and make additional investigations which patients require on top of the investigations which were done at the EMERGENCY, so either changing of the drugs or addition of the drugs depending on the results which came.  A consultant is a senior medical person on that particular department. They may be 3 or 4 and they will allocate themselves according to the bays. One consultant may be on bay one where there is anemia, malaria, diabetes or hypertension, the other consultant would be on bay two where they normally admit TB, meningitis or the septical meningitis or query the TB. The third consultant will be on bay three. Sometimes another consultant will be doing rounds in XXXX. A consultant guides the other teams of junior doctors.  At the EMERGENCY, if the patient is having Sepsis but is not severely sick, after all the tests and the patient is confirmed that it’s Sepsis then the patient gets medication and then he goes home. This usually happens to patients that present to the hospital in good time. | | *Yes, that’s when they look at the signs and symptoms that the patient has provided them like fever, dizziness, tiredness, reduced urine output, and reduced levels of consciousness. If there are bacteria in the blood stream, white blood cells are produced in large amounts to fight against the infection, and when the alleviation of white blood cells they conclude it sepsis, then the patient is sent to the ward, like in our ward we have bays, bay 1 we have patients with Diabetes, TB, Stroke, and hypertension because they need attention, malaria, Sepsis bay 2 depending on the condition, mostly on bay 2 we focus on checking temperature because these patients mostly have fevers. And then highly fluids are mostly maintained 24 hours because the patient mostly have reduced fluids output, we make sure we have urine monitoring charts because we need to balance fluids input and output, and you monitor the patient because in the ward they are put on strong antibiotic and that’s the most treatment patients in the ward receive.* |
|  |  |  | *Normally septic patients at this hospital, pass through EMERGENCY which is emergency trauma, so from there they are assessed, if she’s a female she gets admitted at our ward XXXX but if she is seriously ill and there is no space in our ward she is kept there in a room called short stay. After being stabilized then she is transferred to XXXX. At XXXX, they are supposed to do different investigations FBC, Blood Culture, Urea and Creatinine Chest X-ray, then she is sent to XXXX, where we do further investigations, we repeat FBC, Blood culture, chest x-ray, grouping and cross matching, maybe she is anemic, we give her blood and sometimes we start giving them iv fluids like normal Sala lain and lingerslatate. If she has been prescribed antibiotics from EMERGENCY we do continue, sometimes we give them analgesics because they feel pain. Normally patients with sepsis are supposed to be isolated to avoid further infections or infecting other patients so we keep them in the side rooms, we restrict to one guardian per patient. And we give them masks.* |
|  |  |  | *Normally patients with sepsis are referred to XXXX or ICU when they are having difficulties in breathing, sepsis means infections in the blood so when the infections are multiplying they affect the breathing system which is called respiratory system as a result the patient start breathing faster and they use accessory muscles to breath then that patient needs supporting machine to help them breath as a result the patient is sent to ICU, but if we that they are just breathing fast that means we will put them on oxygen therapy which is in XXXX* |
|  |  |  | *The diagnosis that can confirm that a patient is having sepsis: 1 is the blood count, what will happen in full blood count is that the patient will have raised white blood cells after ruling out MRDT and MB, if MRDT is negative and MB is negative and white blood cells are raised you expect sepsis in that, as well as blood culture will be able to tell us this patient is having sepsis.* |
|  |  |  | *My job is to register patients, when a patient arrives we record their name and where they have been referred from, then we send them to triage where all vitals are tested after that they go see the doctor for consultation. When they are done they come back to be registered in a manual book, in this book we record name of patient, age, doctor who attended them, their diagnosis and treatment given.* |
|  |  |  | *The OPD we have now is much different with the one we had before, which allowed patients to be treated very fast. But now patients will wait in line to be given cards then they will come one by one at the reception to be registered, after being registered they will join another line to meet a nurse who will test them for all the vitals like BP then the information is entered in a computer, the nurse will then tell them which area to go to see the relevant doctor. Mostly this process starts somehow late which makes it quite painful for patients, sometimes patients will just give and go home before seeing the doctor.* |
|  |  |  | *Sepsis patients are mainly in wards XXXX and XXXX. After they have been assessed or they have a different condition which warrants them to be transferred they would be transferred either to the XXXX or to 2A. XXXX that’s a TB ward while 2A it’s an oncology ward that’s cancer ward. But firstly they would come to XXXX that’s female medical ward and if it’s a female patient, and if it’s a male will be admitted in XXXX.* |
|  |  |  | *In the night it’s a different story, all these people are not there and people avoid the health center in the night so they will come straight here, so when they come the nurses in the emergency department who are on duty do realize that the patient has come with fever and will do a malaria test and full blood count and if its negative so they know something is going on, this is not malaria, if its malaria and they are very sick even before the doctor sees them they start giving them malaria drugs, the overzealous nurses if they see that the patient is very sick they give them the first shot of antibiotics. The problem comes in with the blood culture now because the blood culture test is supposed to be taken before the antibiotics, what I have noticed is that during the day this works very well, during the night when there’s shortage of nurses some of them are unwilling to do blood culture because it’s a bit more involving to take that sample, so by the time you get to see the patient at least they have preliminary results. My numbers are good in terms of the health workers that we have we do get the full blood count maybe within an hour so at least you know, it helps you to make the diagnosis, then depending how severe they are then we give the necessary treatment, if we know the source of the infection then we give the antibiotics.* |
|  |  |  | *By the time they will be coming to the ward most of the investigation are already done, the first management has already been done, so ideally they come to the ward when they are stable, so you need to send them down there when they are stable and you know they will survive till the next day. If they are in shock, then we admit them in the high dependence unit in the ward, if not in shock you send them to the main ward. In the high dependence unit you are sure they be seen within 4hours because there the clinician is usually around, but in the wards they go and see the patients once and they see them again the next day.* |
|  |  |  | *Like for me I don’t mind if they have given them the first shot of antibiotic so long they have taken the blood culture before the antibiotic, I don’t mind, because sometimes maybe you are busy with some other patient, so why should a patient wait for you to make an order? So for me I don’t mind so long as they did the blood culture, if they haven’t then that’s an issue with me.* |
|  |  |  | *When the patient comes to our ward, some say that this young doctor did a very good job, making sure that this patient received this much of the fluids, and managed to achieve this target of blood pressure, this target of urine output, they managed to make sure that patient levels of conciseness has improved after that initial resuscitation. With other clinicians that doesn’t happen, so it’s different. A patient who has got sepsis is seen by emergency department, then medical team on call, they admit the patient depending on the condition. If a patient also requires oxygen, requires continuous fluid resuscitation, they usually admit the patient to XXXX, high dependency unit. If the patient has received adequate amount of fluids but the blood pressure is not improving they send the patient to ICU, because they can also add other modes of treatment like inal troops. If a patient comes to medical ward or XXXX, then there is a team that continue with the treatment.* |
|  |  |  | *Usually patients that come to medicine department they need to pass through the EMERGENCY which is an emergency that admit all adult patients to different departments, they may be ganorogical problem, medical problem, surgical problem, all those problems sometimes may complicate into Sepsis. Patients are firstly seen by the casualty doctor, the EMERGENCY doctors. Nurses triage the patient to determine whether it’s a severe disease, or life threatening, or mild. Depending on the condition of the patient they maybe triaged as life threatening condition and they need to be immediately seen by a doctor. So the first person to see that patient is usually a nurse in EMERGENCY, triage them and then inform the doctor. One of the indication is sepsis, some may come with sepsis and that sepsis may come extreme whereby their blood pressure is very low so it’s a septic shock and that patient needs to be seen by the doctor immediately. Staff at the EMERGENCY resuscitate the patient depending on the condition of the patient. For Sepsis, usually they do full blood count and they take blood culture. The patients might have low blood pressure, and we start IV fluids. Depending on the condition of the patient they might take the patient to resuscitation place, especially the patient whose blood pressure is very low, oxygen is low, they need oxygen supplement, but also needs IV fluids, and thereafter they refer the patient to the medical team on call which is also based in the EMERGENCY. Depending on the experience of the one who is taking care of the patient, some will have a clear cut plan that this patient has got sepsis, these are the targets that I want to achieve, if this patient has this blood pressure this low, my target is I need to achieve this blood pressure within this time of period, if this patient is Septic I need to make sure by this time this patient has received antibiotic, and I have done all the necessary investigations. All that depends on the level of the attending clinician, some they know if I have got a septic patient, this is what am supposed to achieve within this period* |
|  |  |  | *If you think the source is chest that’s the one that is causing the sepsis, you look at the antibiotics, the antibiotics appropriate for the likely organism or not, if they source is in the urine you also know the common organism associated with urinary attract infection, so you look at the antibiotics, is the right antibiotic, or not, and then we also evaluate the initial condition of the patient and the current condition, is there any improvement or not, do we need to change something or not, we also evaluate the investigation that were done at the entry point, EMERGENCY. Is there anything we can do more for the patient? Basically it depends on where is the focus of infection. We always make sure that we check the full blood count, the result of the patient because some patients may even require blood transfusion, if the hemoglobin level is low, we always check blood culture to make sure that the organism that we initially thought is the causative that is right one or not, we also would want to know whether the antibiotic that we are giving is the appropriate one and also resistance.* |
| **The pathway for sepsis patients – the experience of patients and guardians** | We learnt that HCWs presented what should be an ideal pathway for patients with sepsis. In reality, patient experience differed among the patients. Despite following the same pathway, some patients complained of staying in the short stay overnight without being attended by a nurse or doctor despite the patient’s condition being critical and despite inviting the staff to come over. Some patients got a quick attention, doctors run several tests at the EMERGENCY, got admitted to the wards and got discharged.  We also learnt that the patients were being treated for different conditions that led to being septic. For the patients we interviewed, such conditions included: Tetanus, Hepatitis, Tumors in the kidney, Low blood, HIV, Meningitis, TB | | *We went there on 10th July, they gave us a bed and started examining her, and they thought it was to do with child birth but that was not the case. A surgeon was consulted and he moved us to 5B, one day a certain professor came to the ward and started seeing patients, he came to our bed, they explained everything to him and he recommended that we should be sent home, it was hepatitis. They gave us diclofenac at the pharmacy and told us to come back tomorrow at the clinic, when we came back the next day they didn’t give us any drugs. We stayed home even though she was in pain until one day I decided to come back here, we went straight to 5B and met the doctor, I told him that I had read somewhere that a hepatitis patient can take ARV’s and he agreed that it is true but refused to give them to us because she was HIV negative. So we went home empty handed again. Now she is not taking any food, when she does she vomits it right away that’s why we have come back. From Thursday they have been checking her and giving her drugs. On Saturday and Sunday doctors from college of medicine came and on Monday a professor came again and wrote us a prescription, he ordered that she needs to be screened again only to find out that there were other problems. Now they have to take blood samples for further tests because they think she has a tumor in her kidney.* |
|  |  |  | *They told me to sit on a certain chair, they run a test for BP, then they took me to one of the rooms there where nurses were doing more tests then I was handed over to doctors who recommended that I should be given a bed in the main ward, the same night we got transferred to the ward. In the morning they came to ask me questions on how it started then I explained.* |
|  |  |  | *We arrived at the EMERGENCY at around 6pm. They quickly sent us to the short stay ward by admin staff and given a bed. We stayed till 10pm without being attended to by a nurse. We approached a male nurse in the EMERGENCY who promised us that he would come but did not till morning. In the morning we approached a female nurse who later talked to the male nurse. The male nurse came and shouted at us, became reluctant to treat us because we had reported him to his supervisors. Monday morning, the patient’s condition escalated and other nurses attended to us. We were referred to ICU at midday where we stayed until Tuesday afternoon. We were then sent to XXXX ward XXXX.* |
|  |  |  | *They took us on ambulance, when we arrived at the reception they welcomed us, the doctors and nurses were there, they asked what was the problem so the patient told them, they gave him first treatment which I believe was to manage his pain then after a while they recorded all his particulars.* |
|  |  |  | *They did, but that time I was not around so it was my sister who told me the results of the tests. They found he is HIV positive and he has meningitis, they also said these other diseases come when immunity is critically low in the body. So we don’t know if he knew already that he was positive or not, for us we heard it the first time there.* |
|  |  |  | *Yes, we were helped, with injections, medicines, they said she didn’t have enough blood, they were going to observe her so that she receives more medicine but they observed that she was not complaining, her legs were no longer swollen, and she was eating properly, no headache and the dizziness she had stopped.* |
|  |  |  | *It started with fever, headache, and then heartbeat rate change also, it was beating faster and then legs also started hurting, that’s how it started.* |
|  |  |  | *They concluded that it seemed as if the patient had a swollen liver and blood was supposed to taken, and the blood isn’t tested at XXXX but at Blantyre diagnosis. They did write but it didn’t happen, until the child died, the only thing that I had in mind is that she had a swollen liver and no one came to get blood sample so that we go for a test and the other thing is we were at government hospital where they have all necessary equipment, for testing but they said we should go and test outside the hospital and that’s what happened.* |
|  |  |  | *When we arrived there we produced the letter from Sochi, they registered us then we went for blood test. They asked for his wife and I was told to wait outside, whatever happened there I don’t know but I thought it was right for me to know as someone who is taking care of the patient, maybe he is HIV positive, I have to know so that I should be able to protect myself. From there we went to one of the rooms where they performed more assessments, they asked if he smokes or took care of a TB patient before or any family member who suffered TB before after that they admitted us to ward XXXX.* |
|  |  |  | *I started having this problem of blood shortage when I was in form two in 2012, I went to the hospital several times but they were just giving me pherasaphate, most of the times they would find that I have malaria too. After that I didn’t get sick again until 2018. In 2018 I got sick again but at the hospital, they couldn’t not find anything. We switched to a private hospital where they discovered that my blood was low, they gave me pherasaphate again and I recovered. From July 2019 it started again, I have been sick then. They tested for malaria but there was no malaria. They gave me Bactrim but it didn’t help. I was so sick and brought here at XXXX, they said my blood was low at 3.8 so I was admitted.*  *The following two weeks I got worse, especially after they extracted my bone marrow I was so weak. I should say they treated me well that time only that I had malaria but they couldn’t see it. We decided to go to XXXX hospital, we have a relative there so we hoped they would pay more attention there and give me a special treatment, they found malaria, they gave me LA so I was taking it and I wasn’t sick in November and December until last week and on Friday last week I was admitted again but they haven’t done anything about blood because it seems the blood level at 8.6 is better. I had fever, cold and a running stomach.* |
|  |  |  | *Yes, at short stay, that’s where he was scanned and tested, so that they know what’s needed and then he was given a drip of water (IV fluid) and then we went to ward XXXX.* |
| In this section we summarize the pathway of a patient with sepsis and problems along the pathway that might lead to loss of patient from care. This analysis help to identify obstacles at each step of the pathway, how to address it and expected outcomes. | | | |
| **Pathway of care for sepsis patients** | | **Problem along the pathway that may lead to loss of patient from appropriate care** | |
| **Step 1:** Sepsis patient in the community | | **Problem 1:** Patient doesn’t go to the clinic for any reason including lack of transport (high transport costs), use of traditional medicine and buying medication from drug stores without prescription. | |
| **Step 2:** Sepsis patient goes to a clinic/ primary health facility | | **Problem 1:** Patient waits for a long time to meet a clinician  **Problem 2**: No other test is conducted apart from Malaria test and patient is only given Panadol  **Problem 3:** No referral is made and the patient is sent back to community | |
| **Step 3:** Patient is referred to XXXX | | **Problem 1:** There is no transport to ferry the patient from the primary health facility. Transport costs are very high for the patient | |
| **Step 4:** Patient arrives at the EMERGENCY at XXXX | | **Problem 1:** Certain measurements are not conducted during triaging because equipment is not functioning (i.e. BP machines) OR the measurements are conducts but with wrong readings  **Problem 2:** Patient waits for a long time to assessed my a clinician after triage by nurses  **Problem 3:** Patients is sent to short stay ward but there is no monitoring on how the patient is progressing  **Problem 4:** Patient is not informed of the outcomes of the examinations and what treatment they are undergoing  **Problem 5**: Patient is sent to the ward without conducting other necessary initial steps including taking sample, giving IV fluid due to lack of resources or HCWs not willing to do so. | |
| **Step 5:** Patient is sent to the ward | | **Problem 1:** Nurses in the ward delay to receive the patient and the patient the required attention and continue care (due to poor work relations with nurses from EMERGENCY)  **Problem 2:** The ward if full and the patient sleeps on the floor and along the corridor which makes provision of care difficult. Patient is offered a bed but there are no beddings.  **Problem 3:** Further tests are conducted on the patient but the patient never get to receive the results nor is he/she informed of the progress.  **Problem 4:** The hospital is unable to conduct certain tests i.e.…. and the patient is referred to a private hospital. The patient is unable to go due to high costs  **Problem 5:** There is no medication in the hospital for the prescribed drugs i.e. …….and the patient is told to buy. The patient is unable to buy.  **Problem 6:** There is poor sanitation and hygiene in the hospital including broken and nonfunctional toilets, poor disposal of wastes and sharps, exposing the patient and guardian to further infections  **Problem 7:** The patient is exposed to non-nutritious and not well prepared food**.** Patient has no appetite for food to support treatment  **Problem 8:** Poor monitoring of the progress of patient due to limited HCWs and poor relationship among patients/ guardians and HCWs in the wards | |
| **Step 6:** Patient is discharged from hospital | |  | |
| **The priorities of patients** | In the midst of a complex experience of sepsis care, we asked participants to prioritize on their needs in relation to the care they receive throughout their care pathway. The following is the list starting with the preferences that were commonly raised by the majority of the participants and some selected quotes:   1. Feeling welcome at the hospital and given all the attention – when that happen, they become relaxed and happy. Participants hoped for a proper engagement with HCWs than being shouted at. Being able to understand patients’ complaints 2. Receiving proper medication in relation to their condition and in a timely manner. They should feel better when discharged and given other medication to continue at home. Patients should not be given medication when clinicians are in doubt. It should be evidence based and there is proof. Patients should not be told to go buy medication 3. Accommodation for patients need to improve including providing beddings as previously done. This would address problems that people from far and poor households face when they have been inexpertly been admitted. The hospital should work on avoiding patients sleep on the floor. Expand the wards 4. Participants expects a clean environment including ensuring that bathrooms and toilets are not dirty. There should be enough cleaners than letting cleaners clean the bathrooms. Supply of water should always be consistent. Water shortage affecting cleanliness and toilets and bathrooms become a mess 5. Proper communication between patients/guardians and hospital staff. They should be able to understand one another. They should great us, then guardians should be updated on the progress of their patient, not hiding information from them. Communication and engagement include being explained to of the results of their tests and being given confidence that they are getting the right treatment and they will be healed. 6. Patients prefer timely services. There were case where patients were attended to 4-5 hours later of their arrival in the hospital. Sometimes patients stay up to a week without receiving any treatment 7. Clinicians should readily be available in the wards every time a condition of a patient changes or an emergency crops up than waiting to be seen when doing ward rounds. There should be enough doctors 8. There is need to improve the food in the hospital, so that there is variety of food (not just nsima, beans and cabbage). This can slow the healing process   Other preferences included: HCWs should run all tests based on what a patients has complained than giving medication without any tests; Doctors being able to follow through all their patients when they have been referred to other departments and see the outcomes; Patient/ nurse ratio should be reduced. A nurse can provide better care if she/he is able to manage 10 patients; Treating patients and asking them questions in privacy and Availability of transport for referring patients to XXXX. | | *First of all when you arrive at the hospital you should feel welcome, secondly nurses have to do all require tests regarding you complaints, not just giving out drugs and send them back no. another thing is that doctors should follow through on any patient they take, if you send a patient to have TB test for example you need to make sure they are given proper treatment when they come back with results.* |
|  |  |  | *Patients should be given medicine in time and proper medication as well, when discharging a patient, they should be sure that he or she is better indeed and they should be giving enough drugs so that when we go home the dosage will continue.* |
|  |  |  | *If a patient is having any trouble even when it’s not time for doctors to see patients, they should be available to help all the time. Another thing is communication, staff and guardians should be able to understand one another so as to give the best care to patients.* |
|  |  |  | *When you are sick and when a nurse greets you, you feel it deep down your heart that you have been welcomed. It shows that the people are friendly, I have said this because at lighthouse, haha, I am referring there because you feel it that you have been welcomed, nurses greeting you, asking how you are feeling so even if you were seriously sick it slowly improves thinking they have helped you.* |
|  |  |  | *First thing patients want at a hospital is love from the doctors and staff, the second thing is the availability of treatment like medicine and the third thing is clean environment, all these three will make a patient appreciate.* |
|  |  |  | *The second thing is I wish they could improve is that the patient should not sleep on the floor, if they are many people sleeping on the floor that means the guardians will also sleep together with the patients* |
|  |  |  | *Proof should be there from the doctors when giving drugs and treatment to patient because when patient knows that they are receiving the right treatment those are the things that would make be satisfied.* |
|  |  |  | *one; I think the best care would mean on nurse against ten patients whereby that nurse could be able to respond to my needs, yeah when I say can you come I am not feeling good at such she could come and assist me. Two; mmmmmm I would wish the nurses or doctors to understand better to my complaints everyday which is difficult in our setup.* |
|  |  |  | *To them the majority of patients expects to be reviewed by the doctor almost each and every day, more frequent doctor comes and examines them and see what is there problem. To them they would see a student or junior doctor and say aaa yes I was viewed but aaaa but some would say that maybe that one should see me (pointing to a senior doctor),so to them some they realize that this is a junior worker, this one is a student* |
|  |  |  | *They will meet a student some they even call you, but you ask them that someone was attending you , aaa no I want you, so it’s like they will value that they are being seen by senior doctor and the care will be different ,of the different standard and some would say that maybe the frequency of the drugs, despite that they have different conditions they have different drugs to get, so I would normally see this when we are dispersing drug during drug time, whereby we would , some would come and queued like the guardians, the very sick patients would go on the bed side, so when you tell this guardian that aaaa , right now we are not giving them drug, we will give them tomorrow, and seeing their friends getting those frequently they would say that aaaa I think we are not being helped, some would actually say, since I came 3 days ago I have never be reviewed but a nurse went there checked the vital signs, get the drugs, maybe the doctor said let’s wait for a particular result for us to come and see the way forward so to them it’s like they are not being attended.* |
|  |  |  | *For me, from experience, patients are a bit appreciative if you show that you care. I have noticed that patients that I have given much attention to in terms of the number of times that I see them they tend to be appreciative compared to those that I haven’t seen as much often, they feel like you are ignoring them.* |
|  |  |  | *I think it’s our attitude, they’re more open to you if you show them respect. I don’t really know if its culture in Malawi but people don’t respect patients in my opinion, we tend to dismiss them, we don’t talk nicely to them, and we scold them far too often. But if you show that you respect them and you care and you understand their circumstances, find out why they have made the decisions they have made instead of telling them off: you made the wrong decision, why did you do this bla bla, but if you ask them how come you did this then they start telling you and they become more open they even tell you things that were not on the file then you start to understand what’s going on. With me, I have realized that I do this when I have few patients but if you have a lot of them I don’t have the time to sit down and talk, I just do what is necessary at that point.* |
|  |  |  | *.The healthy workers should be able to do all the necessary test and give me adequate treatment, that will make me get better so what I want is me to get better, now when patients come or go to different healthy facilities, most of the times maybe the clinician is not there, or is there but has got so many patients to see, the patient may come 8 o’clock in the morning, will only be seen 12 noon, not deliberately but because he has got lots of work, so as a patient what I need is when I go to hospital am seen in good time, all the necessary investigations are done, and appropriate treatment is given to me in good time and I get better as soon as possible, does that happen in our setting? Sometimes it does but also other times patients get frustrated because they feel they have come to hospital in good time but they are not receiving adequate treatment in good time.* |
|  |  |  | *You should give them medicines, once start saying we don’t have this type of drug, its challenging for them, some of these drugs for example meropenem is super expensive, if the pharmacy say they don’t have it asking a patient to buy is difficult for them, so you can tell that they are not having a good experience* |
|  |  |  | *Experience of care depends on a number of factors. If they came in the hospital very sick, and whatever treatment they got made them feel better, obviously they will have a positive feeling that the hospital made a difference to my life. If they came in the hospital and things didn’t improve, obviously they will feel bad about it as hospital or if there were investigations that the hospital could not do, they always feel bad about it, and worse if they came in the hospital, people investigated, they have a definite diagnosis, but there is no treatment for that diagnosis, and the doctor didn’t take time to explain, to the relative and the patient, obviously they will come out of the hospital saying these guys were just taking blood, they were just doing all sorts of things yet at the end of the day I didn’t feel better, so lack of communication will also determine how patients or guardians feel about how they were treated in the hospital, but if you had time explaining to them in the hospital that this is how far we went and this is the diagnosis unfortunately there is no treatment for it for example cancer which is not treatable at least they understand but if you don’t explain to them you just leave them like that, they go home and they speculate, I had this problem those dangerous doctors couldn’t even come up with a diagnosis, they couldn’t even treat me.* |
|  |  |  | *I will start from the clinic, the clinic should have a means of transport for patients who are referred to other hospitals, here at XXXX everything at XXXX is ok but when you go to the ward, they need to have beddings ready whenever a patient is coming, the toilets are in bad shape they have to be fixed, there can’t be a single bathrooms for the whole ward, we have male guardians and we are using the same bathroom that has a broken door too.* |
|  |  |  | *To my side, my wish is to have the results of any test they do on me, and the doctor should give you confidence that the treatment they are giving you will heal you. There are some doctors who will shout at you if you remind them that you haven’t received your drugs and there are others who will help you get your drugs.* |
|  |  |  | *The things that I would love to see happening are that when tested, they should tell you the outcome or maybe explain what is happening, because sometimes they might be discussing amongst themselves while without your knowledge.* |
|  |  |  | *Patients should not be waiting too long on the queue, someone who stayed too long on the queue started puking blood, maybe if they had checked him earlier this could not have happened. My card was checked by four doctors but every one of them was saying just wait here and had it been there were no research people around it could have been worse, they intervened and helped.* |
| **Things that are working well** | Patients and guardians had myriad of positive experience in their sepsis care pathways. HCWs also give their perceptions of what is working well in the hospital in the care of sepsis patients. We focus on these factors as well to help the hospital management maintain such standards and motivate HCWs feel appreciated in their work. We summarize the positive factors below:   1. Nurses in the wards pay urgent attention to the patients. Whenever they are called, the do come by and assist without hesitation. They even felt sorry for them as they have no time to rest especially at night. Despite the congestion in the wards, they make sure everyone gets the required attention. Doctors as well assist the patients with examinations and treatment in a quick manners. They are able to engage the patients when they come to the bedside to find out how they are feeling and discuss progress of their treatment. 2. The clinicians in the hospital are able to carry several tests to establish what the problem is. They are only let down due to some of the non-functional machines. 3. HCWs indicated that despite several challenges, most of sepsis patients respond positively, they quickly get better are discharged from hospital comparing to the experience in clinics, private clinics and district hospitals. This was also one positive that was mentioned by most patients when we interviewed at home. 4. Some patients commended the good relationship that existed between HCW and patients/guardians. They never experienced and shouting or scolding from HCWs. They expressed having experience a warm reception from the HCWs and quick help. They compared with previous experience that things are now changing. HCWs are showing love. 5. Participants appreciated the fact that they were given a date to come for checkup. This showed the HCWs commitments to follow the patient and monitor progress. 6. Patients appreciated that HCWs are always abiding to the exact time of giving drugs according to prescription. There is no delay in giving medication. They come at the exact and recommended time. All other thing happened on time as well including making ward rounds and cleaning the wards 7. Patients and guardians commended community clinics for quick referral to XXXX when they noted that they couldn’t manage the condition 8. HCWs felt that they are trying their best. They felt that guardians do appreciate that as staff, they try their best and they do understand when they are told that such and such a drug is not available. 9. Some HCWs felt there is team work in their approach to work. Nurses and doctors work in support of one another including cleaners who try to keep the environment tidy, avoiding infections. 10. The screening process is one element HCWs felt work well in the hospital. Triaging at the EMERGENCY helps to make sure that the hospital is not congested as some patients are sent back to clinics. So every patients is triaged before seeing a doctor.   We noted that although most of these positive experiences are in contrast to what participants raised as areas requiring improvements, these positive experiences were raised by fewer respondents. We deduce that relational aspects of care differ among patients and HCWs depending on several factors including personality and availability of resources. However, amenities of care and technical processes may be constant. | | *They tried their best by doing the tests, had it been that the machine they needed to do the tests was working the first time we visited the hospital, then we would have been assisted earlier.* |
|  |  |  | *I am not fearing anything, I am telling the truth without hiding anything. The relationship between us and the healthy workers is great, ever since I came I have never heard them shouting at us or speaking something bad about us.* |
|  |  |  | *We were helped quickly, given two drips, injection and around 3pm the patient called for food yet it’s been a while since we saw the patient eating apart from taking water, porridge but the patient asked for food due to hunger and that’s we realized that hospital is better.* |
|  |  |  | *Yes, the improvement is there because the time we were at XXXX we weren’t sleeping, groaning all night, and we would pour water on the patient to feel better, and the leg could be up but after 2-3 days it stopped and we are sleeping very well.* |
|  |  |  | *At first he could not talk, he could not hear us, he wasn’t eating, only we could force his mouth to put in some food. After we stayed for three days then they started giving him proper treatment for meningitis, drugs, water and glucose that’s when we noticed some improvements, he could open his mouth and talk or eat. The only issue now is loss of memory he completely lost it but now he’s getting better.* |
|  |  |  | *I think things are improving, previously he was coughing very much and I reported to the nurses who said it is because he has TB but it will improve, he was also having difficulties in breathing but now everything is improving, he can sit upright, eat.* |
|  |  |  | *I thank them for they welcomed us very well with love. When we arrived they didn’t take time help us, up to the extent that the patient had appetite to eat and was hungry since the patient was eating and that’s when we knew that they have really tried assisting us and if we were not late the patient wouldn’t have reached this stage.* |
|  |  |  | *The relationship was good generally, the cleaners would come in and ask politely to clean the room, the doctors were always available and the communication was good too. There nurses were there all the time, it could happen that the nurses would act somehow questionable, like they could leave the medicine on the bed and come back later but that’s not a big concern they are human after all, they get tired as well. Overall it was a good relationship.* |
|  |  |  | *Here they are able to do tests and scans if there’s a need to unlike at XXXX, they don’t have enough equipment. Treatment I think is based on what the doctors know, so on a scale they are doing more here because they are able to find out any issues.* |
|  |  |  | *I appreciate that they don’t change time that they give drugs to the patients, if they say 8 o’clock it’s exactly 8 o’clock, if its 12 o’clock it’s exactly 12 o’clock and the same thing happens in the evening.* |
|  |  |  | *I should talk about doctors and nurses in XXXX, I had no problems with how I was assisted because the doctors would come on our bed and tell us what to do on daily basis and also to write our files so I didn’t spot any problem with them and nurses and even the second trip I didn’t see any problem. The doctors were asking us if we have spotted any problem on each day.* |
|  |  |  | *I haven’t seen any issues in XXXX, the doctors are showing love to patients and are cheerful, I have been here before as a guardian and that time doctors were shouting at patients but that is not the case now, there are improvements on the part of doctor’s treatment of patients. Now they take patients as kids who need love.* |
|  |  |  | *We are very thankful to the staff of the clinics, they were able to help us until they referred us to XXXX, without them we would just be buying antibiotics from the stores, there was a time when our patient could not move and the doctor from the clinic followed him home, he showed that he cared.* |
|  |  |  | *I just want to commend the doctors and the nurses for job they are doing, they are few but they work hard, they try to whatever it takes to help everyone despite that the wards are congested. Even in time of death they are available to help those passing, they don’t stay away from the ward, anytime you need them they will come.* |
|  |  |  | *Even the guardians do appreciate that we are trying our best, because we tell them we don’t have this medication and we have thought of giving you another but that’s if they can’t manage buying the drug we want which could have worked faster and condition could have improved, and they do appreciate for the sharing them the message, and the care we lender to them some do understand that the government is going through such crisis.* |
|  |  |  | *I only see positive things at EMERGENCY because a patient when has is kept there at short stay, they do all the tests of samples before the patient goes to the ward, and at times if you are lucky the patient comes with sample results to the ward.* |
|  |  |  | *Each person has a different attitude but as a manager I do have meetings with them and advise them how best we can interact with patients. Normally I do advise them not to be offended when somebody talks badly to them because these patients or guardians are confused sometimes so if you talk back badly to them you may regret. They should not expect any respect as if they are at home, so the relationship is a bit fine.* |
|  |  |  | *In our ward we are managing patients well. We choose a shift leader, regardless of having an in-charge to monitor every nurse to make sure that he/she has done the job well, who hasn’t done the job well is not supposed to knock off until the job is done, so we manage patients accordingly, we also make sure that resources are available all the time, both human and material so we do follow ups to our nurses, doctors, clerks and patient attendants, when they are absent we find a replacement to cover up until they return so we don’t have gaps.* |
|  |  |  | *When I get to the office I start with mopping, dusting, cleaning nasal tubes, cleaning the bed if they are no patients there, because if a patient sleeps on a bed that is not made then I am not fulfilling my job roles. I am also supposed to take the beddings for laundry then bring them back clean, and sometimes you might find patients without guardians and I also help them. Instructing people in the ward on what they are supposed to do and follow though some don’t listen. Receiving patients and guardians because some guardians they don’t know what to do once they reach here, some would go to get the chamber without gloves, but I instruct them to get gloves and after they are done with the chamber they should clean their hands, because they don’t know what the patient is suffering from.* |
|  |  |  | *The best things happening at EMERGENCY are testing of vitals and registration. Some patients may run away from hospital for example but we will be able to locate them since we have their information in a computer. Testing of vitals reduces the risk of giving wrong treatment to patients.* |
|  |  |  | *Sometimes you would see a serious patient struggling but they would still help them up to the extent that the patient gets better and with that we appreciate them and it also makes us happy.* |
|  |  |  | *Sepsis patients to me I think I see them getting better, if I see patients that come in with opportunistic infections that’s the HIV, because the sepsis patient will normally come in the hospital when not in a bad condition when I compare to patients that have opportunistic infection, so it’s like a sepsis patient will come in as an acute patient not as a chronic one, so it’s very easy to manage a patient that has come in as an acute then this patient will recover very well, comparing to the chronic patient who have other conditions.* |
|  |  |  | *Of cause I would start with the monitoring of the vital signs, because the majority of patients with Sepsis some would come with high fever, whereby we need to manage that by through monitoring the vital signs you would detect that the fevers are high and then you give the planned care either by the sponging, exposing, giving the antibiotic drug that’s the paracetamol, aspirin depending on which one the patient will take, and then you would monitor seeing the temperature dropping, the heart rate dropping meaning that the sepsis is decreasing, with the drugs given you would see the patient improving as well and being discharged. So normally we would see if the patient who came in very sick and then 2 / 3 days improving and later on being discharged and we become very happy because it’s something sad seeing patients dying now and then, it gives different impression like we no longer care or as if we are happy seeing people dying but it really concerns us but whenever a patient improves we feel so great.* |
|  |  |  | *I think our relationship is pretty good, I can give an example of the emergency room, if you guys don’t relate very well things don’t move, like for a doctor I need to have a very good relationship with the nurse and the potter, a potter is the one who will be taking the patients out of the cubical for new patients to come in, so if there is no communication among three of us, the patients will pile up and the nurse will alert you who is critical to start first. If you don’t continuously engage them they will just sit and have tea, so the relationship is good. In the ward is the same thing, first thing I find out who is stationed on my place because even if I write in my file do this do that still it will not be done, so in terms of relationship is good.* |
|  |  |  | *So equipment is a problem but even though it is an issue in the emergency department each and every patient has vital signs because they cannot get to bed or anywhere without someone taking vital signs, it is also a way of sending people back, because if every Jim and jack went through the place would be overwhelmed, there are some who are sent back home, some to the clinic and others to the health center, so there’s someone assigned to make sure this happens and that helps that no one goes to see the doctor without having vital signs* |
|  |  |  | *My temperature was high and I was vomiting. I called for the nurse around 10pm but she came around 12mid night, she delayed because by the time I was calling her, she was already attending to another patient so she forgot afterwards and she apologized, she treated me just fine.* |
| **What HCWs want to know from patients** | We inquired from HCWs if they have any uncertainties of anything they would like to know from patients and their guardians concerning the care they receive the hospital. Most HCWs wished to understand how patients and guardians feel about the attitude of HCWs and how they HCWs interact with patients; how do they expect the nurses to work? Including cleaners. They also wanted to know what areas the hospital should improve as a way satisfying patient needs and expectations. They also wished to learn about patients’ waiting time, how long it takes to see a doctor and how free are they to explain their problems to a doctor (engagement). | | *I would want to know how we interact with patients and guardians, how do they look at us in terms of care, what comes to their mind when they see us. I can give you an outside example, previously civilians and police were in good terms but now they are enemies. So I don’t nowadays how these patients look at nurses, do they look at us as people who could contribute to death? Sometimes when death occurs you listen to how the people are crying, like they wish they did not come to this hospital.* |
|  |  |  | *Ask them the time they have arrived here; what time have they seen a doctor? And ask them about patient care or if they were free to explain their concerns to the doctor.* |
| **Sepsis is a common occurrence in the hospital and a concern** | We learnt from HCWs that sepsis is a common occurrence in the hospital. Doctors expressed that it is a common condition they admit to the wards every time they are on a call. It is thus important the hospital reflects on ways of improving the care rendered to the growing number of patients with sepsis based on the experience of sepsis patients. | | *Sepsis is very common, it’s very rare that you are on an emergency call, where we admit patients in the emergency department, it’s very rare not to admit patients with sepsis, there’s usually a patient every day. So it’s very common condition, it’s very common to have on your bay, in the ward, so it’s a condition that I meet on a daily basis.* |
|  |  |  | *You can see patients that come with sepsis when they are already admitted in the ward during ward round but also in XXXX (high dependency unit), so I do see patients that have got sepsis, either as new patients in the EMERGENCY, Emergency department so they could have referred to our medical team on call, so when you are doing postmortem you can get into contact with the patient that may come because of sepsis. In the wards sepsis it’s a common medical problem, it’s one of the reasons why patients get admitted in our department, but I also sometimes see patients that have got sepsis when am doing chest clinic so you’ll find patients that may have come with signs of pneumonia and then on initial evaluation you peak that they have developed sepsis because of the pneumonia so we refer them to medical team.* |
|  |  |  | *Yes, sepsis is a common occurrence, people cannot rule a call without admitting a patient that has got Sepsis, for some different reasons, some of them have got maybe meningitis, others may have pneumonia, others may even have urinary tract infection, some even come with surgical conditions and they become septic because of that focus on infection, some may come with a bima that may push them to Sepsis, so it’s a common occurrence.* |
| **Recommendations** | The following are some of the recommendations that participants raised as a way of improving the care of patients with sepsis:  ***Recommendations to patients and guardians***  ***Recommendations to HCWs at XXXX***   - Patients’ engagement and communication about their diagnosis, prognosis and decisions that HCWs are taking. Otherwise non communication breeds unfounded speculations among patients and guardians. - Work towards reducing patient waiting time. Timely attending to patients and quick handovers when HCWs change on work shifts.   ***Recommendations to primary health facilities***  ***Recommendations to the health system***   - Make basic resources to treat sepsis readily available in the hospital including drugs like antibiotics. - Improve on primary care facilities to be able to diagnose and manage patients with sepsis. XXXX become overwhelmed and patients know that they can only get help at XXXX. - Facility audits on the use of antibiotics based on recent trends of drug resistance as a result of abuse of antibiotics. - Enhancement of capacity, knowledge and skills of frontline health care providers through refresher courses, CPDs and emphasis on sepsis during medical student training programmes. | | *I’m going to talk about what I saw in that ward both about me and other patients. For two days my patient was not able to do anything but they gave us Panadol to force him, maybe they could have given him the drugs in form injection. Sometimes we don’t know what the doctors are doing because we don’t have their knowledge so may judge them wrongly (issue of communication of what is the diagnosis).* |
|  |  |  | *The waiting time needs to be improved, they should be able to treat patients in good time and also according to what they are suffering from.* |
|  |  |  | *I should say they should continue on this level they are, they operated on a fast pace and everything happened quickly inside the curtains only that we found them switching, it was time for another doctor and that’s why we waited.* |
|  |  |  | *If we improve the care in the healthy centers, possibly some of these sepsis patients can be managed at healthy center level so possibly here resources like bed capacity, will be somehow adequate.* |
|  |  |  | *Availability of resources to treat, like the IV fluids. IV fluids is important. Sometimes we run out of normal ceiln, and if you ask the pharmacy they always tell you no there are people that press orders on monthly basis, we need this amount of ceilin, we need this amount of antibiotic be it cafetraxon or what but at the end of the day you’ll find that whatever they are ordering they are not getting that amount.* |
|  |  |  | *I have also noted is drug resistance, we do get some cases that have got sepsis, but the organism is difficult to treat because of the resistance that it has developed. There are a number ways why people develop resistance but one of the things is misuse of antibiotics. We had an audit recently and our use of antibiotic, cafetraxon is really being abused. Everyone who says I have got what, even without evidence that this patient has got sepsis or whatever infection they get cafetraxon, , so we also need to have these audits and see how we are using our antibiotics, are we using them for proper indications or not?* |
|  |  |  | *Improving the knowledge of frontline care providers - everyone who’s taking care of patients including sepsis, so like nurses, medical students, or medical doctors. There are some that are in training they have to make sure that whatever they are learning they also include diseases like sepsis. Sometimes refresher courses do help, especially in conditions where things are changing every time because of whatever evidence is being gathered. So management of sepsis is not the same, it keeps on changing, so when you do refresher courses for nurses or doctors for few days or hours to discuss about Sepsis, or it may not be necessarily refresher course, just CPD - continuous professional development.* |
| **Patients’ post discharge experience** | Of the 10 patients and 10 guardians we interviewed, one died a week later of being interviewed at home (post discharge) and another died while admitted in the hospital. Most of the patients, though at home, had not fully recovered. However, they expressed getting better than previously. | | *The time we were being discharged the patient was walking, and went to get drugs at the pharmacy and also he walked to the bus stage then home and he never rested even when we got home.* |
|  |  |  | *He is now getting better, since we got back from the hospital, he has never complained of any other problem again, he has some pain to his left side, but he is able to walk though slowly, and he is able to go and watch football at the pitch near just that he needs to be walking carefully.* |
|  |  |  | *He was feeling better, he was happy as that was what he wanted, when we came at the hospital he didn’t have enough blood, he was having heart problems but after giving him two pints of blood he got better, he was supposed to get a third pint but the doctor checked him and said two were enough, he’s just waiting for a checkup on 26 January. He has improved a lot; he wasn’t like this. No more breathing issues, he is even doing some work around the house.* |
|  |  |  | *Right now I feel better, of course my feet started being numb way back maybe because of low blood, but compared to when I was going to the hospital I’m better now. Before I went to the hospital I was sweating at night very much, I would change beddings four times one night, not that I was feeling hot no, I was just sweating, my heart was beating very fast as well, just a small walk and the heart will be beating fast for hours. Now all that stopped.* |
|  |  |  | *There is some change, the vomiting stopped. They scanned my stomach and they found that I had some swelling in the kidneys, by the time I was being discharged, they tested my blood and they found out that I had low blood but they did not gave me any blood transfusion just the pills. The day they discharged me, they told me to get the pills at the pharmacy, and at the pharmacy they gave me the pills for blood only, and they did not have the other medicine in stores.* |
