# Supplementary material for: Facilitating high quality acute care in resource-constrained environments: Perspectives of patients recovering from sepsis, their caregivers and healthcare workers in Uganda and Malawi
Source: PLOS Glob Public Health. 2022 Aug 15;2(8):e0000272. doi: 10.1371/journal.pgph.0000272 (PMC10021962; doi:10.1371/journal.pgph.0000272)
Supplement: S2 File — (DOCX) [file pgph.0000272.s003.docx]

|  | | | |
| --- | --- | --- | --- |
| **ARCS patient, caregiver, and healthcare workers’ experience of sepsis care report (Uganda)** | | | |
| **Thematic areas** | **Summary** | | **Quotes** |
| 1. **AMENITIES OF CARE** | This defines sepsis care delivery **context** including equipment and materials, availability of medication, the hospital environment and, adequacy of HCWs. | |  |
| - 1. **Accommodation in the hospital is limited and lack necessities to make it conducive** | Not enough beds  Patients sleep on the floor  Beds only for patients  Attendants can’t share beds  Ward is full  Attendants sleep on floor  Inadequate space on the ward to accommodate both patients and caregivers.  No beds, some patients and caregivers sleep on the floor and other sleep on the open outside the ward.  Patients felt that lack of enough beds sometimes compelled HWs to discharge patients to create space for new admissions. | | *The other thing that makes me unhappy is that there are beds, and the beds are not enough. You find that other patients are sleeping on the floor and the other thing when patients are sleeping on the ground* |
|  |  |  | *In the hospital, there are beds only for patients. Sometimes we caretakers also feel like sleeping. At times you may find yourself sleeping with a patient on the bed and when the health worker comes and finds you there, she starts to shout and abuse you like that.* |
|  |  |  | *I think that’s why they discharge you early so that new patients can also get a chance to have a bed. Otherwise, if you stay here for 10 days, it means the patients on the corridors will be very many and the HWs won’t manage* |
|  |  |  | *Not inside the ward, they were sleeping outside the ward on the verandah but inside the hospital. They would come and check on me at night and after that they go back to sleep. That also gave me the reason to get better. To know that my family is caring about me. I would wake up and find them, I would sleep and leave them there* |
|  |  |  | *Yes, because the ward is always full, and the beds are few so they don’t have where they will put other patients. So, the patient and the caretaker are both put down on the floor. At least if the patient is sleeping up and the caretaker even if you sleep on the floor, it is okay you will understand it.* |
| 1. **Non provision of and/or availability of worn-out bedding materials** | Broken beds  Attendants sleep on floor  Quick discharge to create space for other new patients  Few beds,  Overcrowding  Need curtains, beds, no privacy during the doctor’s review  Some caregivers provide own mattresses  Vulnerable patients unable to get basic needs during admission  Hospital should provide more bedsheets and curtains  Expect that hospital provides beddings and admission requirements  Poor patients admitted without bedding  Some mattresses lack mackintosh  Patients fear to get other infections  Bed in HDU not enough | | *No, I never hoped to find them there even the issues of the beds that are spoilt, I wasn’t hoping to find them there* |
|  |  |  | *Yes, it happened to me, I used to put a mat down and I sleep there. And even what surprised me was the day when they were going to discharge us, they removed us from the bed and put us down and they put another person on the bed.* |
|  |  |  | *The way I told you, the buildings are not enough, the care is not very good because of overcrowding on the ward, because the beds are very few, they should at least add more buildings to cater for those problems, they add beds, and curtains. That one of the curtains, I forgot to tell you. Here, if the patient is naked, it is you to look for a shawl and cover her or may be with your bedsheets, they will always work on her in the open. At least if they could put curtains so that when the patient is being seen by the doctors or by the nurse, we pull the curtains down.* |
|  |  |  | *Then in the morning they said that if you want your patient to be attended to, you go and get for her a mattress and the bed. I also ran very quickly and bought the mattress* |
|  |  |  | *Then another thing that I saw in the hospital is that people are vulnerable now like me I would want to go to the hospital, but you find I don’t have even a small piece of cloth to cover myself in hospital. So you find I have come to the hospital and they have given me the bed but I just sleep on it like that without a blanket or sheet. So that’s why I requested that if they can add more bedsheets in the hospital, so that if a patient comes like me, they can give me the hospital bedsheets and I also cover myself.* |
|  |  |  | *When you enter you see the neighbor who seems to be okay has only one bedsheet, when they say let’s change the bedsheets, you really see people don’t have anything to use to cover themselves. So that’s why I was requesting that they should put for us more bedsheets in the hospital so that even a poor person can also come to the hospital and find a bedsheet to cover herself…* |
|  |  |  | *Like those for people using catheters, and things like that, there should be beds to sleep on, curtains for people not to see you if you are being examined* |
|  |  |  | *We have HDU but HDU only have six beds and many times they are full and patients here are very sick and need to be in a critical area. Many of them cannot manage to go to xxx hospital because they cannot afford. If we had an emergency unit that handles septic patients, it would be good for health care.* |
| 1. **Congestion/Overcrowding on wards** | Overcrowding onwards, sleeping on the floor, caregivers jumping one another  Caregivers thinks patient not ready for discharge  HW discharge patients early due to fear of covid  Before Covid, the ward was highly congested  Some patients sleep on the floor and along the corridor as they wait for admission by the clinicians  Patients put on IV fluids on the floor  Beds close to one another  Fear of infection and injury  HWs talked of the need to expand the wards  Space of two meters not met | | *The other thing that makes me unhappy is that there are beds, and the beds are not enough. You find that other patients are sleeping on the floor and the other thing when patients are sleeping on the ground, we find ourselves overcrowded and we start jumping other patients because as you are attending to your patient, the other person is busy sleeping on the mat and is also on the drip while sleeping on the floor. If this facility could be expanded with more beds in the hospital, you will find that the services are improving very well without having to jump patients with your legs when someone is very sick* |
|  |  |  | *Considering that the hospital is full, he was still too weak to be discharged. He has not yet regained his energy. But that the hospital is full, let him go, he will gain his energy from home. We are going to get for him what to eat and what to drink so that he can regain his energy* |
|  |  |  | *What it means is that the patients are many on the ward and each person wants to get treatment. And since we have been treated and given some medicines, that’s why they discharged us, we first go home and bring him later* |
|  |  |  | *I said but the old man is still very weak why have they discharged us very early. I asked the doctor why they discharged us early, yet my father is still very weak. He told me that he will regain the energy but also that we have now been attacked by Corona virus we don’t want very many people in the hospital. I also said its okay.* |
|  |  |  | *Remember the health workers are telling you to sleep under a mosquito net but there is no where you can place it. I was requesting that the beds be increased, or they get another ward, and they place patients there* |
|  |  |  | *So, this bed is here, and this bed is here, injection can easily pass from one person to another, so we need to have some distance between. The patients must be well spaced, and, in this period, we even need to space them further to prevent them from getting infected with Covid-19.* |
|  |  |  | *By the way even a nurse or a health worker can get infection from those patients because when you look at that ward, we have the septic corner on both sides but what is the space between the septic corner and the non-septic corner? There is even no demarcation, no space and the beds are almost torching each other. Leave alone lack of privacy. A hospital like this should have curtains or partitions.* |
|  |  |  | *Other maybe in the long run for example when you look at the medical ward, sometimes you find it too congested, beds are close to each other and sometimes you find those patients on the floor. So, for me, I think there is need for expansion of the infrastructures so that the space between beds can be on standard. This is because the standard space between beds has to be two meters but now the beds were squeezed to get a bigger number* |
|  |  |  | *The high influx of patients is causing that congestion and also the ward is small yes, compared to the number of patients that we see every day. The congestion has been all over. Actually, before covid you used to find floor cases but also before covid there were different doctors here so one would argue that the doctors who were here were doing things differently like we are doing them* |
|  |  |  | *I also forgot to talk about the crowding on the ward, you saw patients sleeping on the floor. So, when you discharge those ones, they go and those sleeping on the floor occupy the beds.* |
|  |  |  | *Their target is always on care. When they come and there are no beds, they say, “we are lying on the ground” yet even the ward if full of beds and it is only that the number has over streamed and there is nothing, then they will say, “Why can’t I stay in the side room?” You then tell them the side room is for serious ill isolated patients. They will try to tell you to discuss with the other staff yet those rooms when we get Ebola cases that is where we keep them. There they leave you with a freely heart but if you do not tell them that they may think you are reserving it for someone for achievement* |
| 1. **Non-provision of meals to patients and caregivers and other necessities** | Hospitals should provide porridge for patients and caregivers who can’t afford  Poor patients and caregivers starve in ward  Concern that an attendant must carry many things for admissions such as cups, jerricans etc.  Provide meals as was bring done previously and in other government hospitals  Patients ask hospital to provide food  Food given in TB ward to limit movement of TB patients  Vulnerable patients are supported with food through the social welfare office | | *C4Since we are not well off, they should at least give us some porridge, because if you are a man, you have come from very far to bring the patient here. Your people at home may not have the money to buy you food or even come to see the patient. And a man cannot cook, you are the only one on the patient. So, you starve, they ask you to buy drugs and you remain with no money to buy food. But if they can give us porridge, at least you stay the whole day and eat once!* |
|  |  |  | *Now to go just here at xxx, you need to go with a cup, plate, spoon, mattress, and bedsheet to cover yourself, so you have to go with everything and sometimes they bring for you some food and you cook from there and you see even the firewood you have to buy them, everything you have to buy it* |
|  |  |  | *Now I told you that for us those days you would go to the hospital, and you wouldn’t take anything there not even a bedsheet, you wouldn’t even take a mattress. You would find there everything, mattress, bedsheets, even food, when you would go there with your caretaker, they would prepare food and give them, but now if you don’t have money you die of hunger.* |
|  |  |  | *From Monday up to Friday it would be posho/maize bread and beans, Saturday, and Sunday it was banana, rice and meat. They would serve a lot and you eat and get satisfied, but now all those things are no longer there. What ended all that is that when they brought the food, the cooks started to steal the rice and take to their homes, the banana they carry and take, beans they take, so that is what removed all those things.* |
|  |  |  | *Others suggest food because in xxx they used to give food. Others say that they were in tuberculosis ward, and they were giving them food, yet they gave them food to limit their movements.* |
|  |  |  | *There are patients who come without caretakers, and they are either brought by police or someone walks in here without a caretaker, yet the condition is badly off. So, we go through our nutrition office with support from the social worker to produce them some food and milk while other will ask why they are giving specific people* |
| 1. **The effect of Covid on patient Care** | HWs treated patients with care  Patients being able to access the bed  HW were not being overworked during Covid  Patients discharged early to reduce congestion  Patients questioning why they are discharged early  Patients not comfortable to be discharged early, they feel they are not very well  With Covid, care has improved with reduced number of patients  Doctor able to give patients more time  Staff being able to take leave  Patients restricted by the transport to the hospital | | *Covid did not affect the care, I think patients were treated with care, may be being able to sleep on the bed or may be the healthy work was not being over worked and but usually when there are many people and the ward and there is only one Doctor on the ward to see everyone that is proportional to the quality of the care in some sense* |
|  |  |  | *I have seen a few cases where HWs say your stay in the hospital is done be treated from home with tablets, you see people are uncomfortable they feel they are not yet well; they feel treatment has just started and they are being told to go home. And I think that is usually in the need to reduce congestion in the ward to have manageable cases in the ward. Some of them are discharged earlier a day or two before the ideal discharge. Even yesterday we had that case the participant was told to go home and was asking but really, we are not yet well why we are going home? Yes, but usually the clinicians do this to reduce congestion* |
|  |  |  | *It has improved because right now when the patient number reduced, work is not overwhelming, it helps me as a doctor. It enables me to see every patient give a patient time. It also helps the nurse to attend to that patient but when they are overwhelmed, they end up selecting and attending to emergencies. When the numbers are few, it helps but again many people may die at home because we do not know what is happening to them because their main form of transport is boda boda and they have been stopped.* |
|  |  |  | *In fact, today, our in charge was saying that we can even take leave because the patients have reduced. You can see for yourselves that some beds are empty especially on the male side. People can’t come here without transport* |
| 1. **Security concerns** | People staying on ward without patients  Caregivers expect security of their patients and property  Safety concerns over caregiver’s motorcycles  Theft within the hospital  Attendants complained about having to leave their patients alone to go outside the hospital and requesting neighbor to watch over their belongings  People sleeping on the ward when they are neither patients nor attendants | | *Another thing, there are people who come to the ward and sleep on ward, yet they do not have patients…they come and sleep there under the beds. People come without patients and sleep there on ward… The one of yesterday we also called the health worker who chased her away after a while, she came back. They later called security to take her away. Yes, I expect security to be tight without getting any problem. There is when a patient comes and wants to kick people and health workers just look on* |
|  |  |  | *Another thing that makes the services poor, is the transportation issue. In other cases, to enter the gates of xxx, there are some people who go in with their safe transport means but for us we cannot afford it, to use safe means you must have money. But in other private hospitals when you go with your means, there is no one who can take it, but in xxx any one can just enter and take your transport means. So that one also makes the hospital to be of poor quality.* |
|  |  |  | *Sometimes you must go outside the gate. And if you are alone as one attendant, you first leave your patient and request your neighbors to keep watching over your patient. Such a situation doesn’t make the care very good* |
|  |  |  | *When I was taking care of my mother and a person came slept under the bed. When I asked that person where his patient was, he told me that he is the other side. In the morning I found that he had no patient he was taking care of.* |
|  |  |  | *we found people sleeping on this ward and yet they were not patients and not attendants. Yes, it happens, I don’t know their plan, maybe they come to steal, I really don’t know. They come and sleep here and leave in the morning. So, patients value security for their property and their health. When they are here, they want to be safe.* |
| 1. **Hygiene and Sanitation** | Caregivers were happy about the way the ward was kept clean  Compound appeared well maintained  Hospital generally clean  No worries when the hospital is clean  Want HWs educate the caregivers to keep the ward clean and how to use the toilets  Cognizant that caregivers and patients were not observing hygiene standards  Fear that poor hygiene causes more infections  Some patients appreciated the general cleanliness maintained at the hospital  Hospital sanitation improved because of fear of Covid.  Hospital observant to Covid prevention guidelines  Patients commended the hospital for maintaining a clean environment  Recommended Health education on infection prevention and control  HWs feel that maintaining hygiene of the ward is their responsibility  Nurses ensure that patients are cleaned, and their beds are clean  Presence of and active IPC committee  Sensitizes patients on handwashing  HWs lack protective gears  Availability of Alcohol based hand gel.  HWs not taking IPC very seriously, not practicing handwashing  Some patients with sepsis are isolated as a way of infection control  Patients are educated on sanitation and hygiene | | *When the day starts, you see all of them coming to clean the ward and after their day, they go back home.* |
|  |  |  | *The services were good; the place was clean; we were staying in a clean place. They have been waking up very early in the morning to clean all the patients, mopping the ward, so we have been staying in a very neat place* |
|  |  |  | *Also, they have been trying to keep this place very clean. They mop the ward, they scrub the ward, I see them cleaning it. Even in the latrines, they have been cleaning there, they have been picking rubbish in the compound. Those are the good things that I have observed here* |
|  |  |  | *The other thing that made me happy is that I found the whole hospital very clean. When you have a patient and you go somewhere and find that it is not clean, you get worried. So, when we found that the hospital was clean, there was not any worry because the hospital is good and everywhere is clean* |
|  |  |  | *I want the toilet to be clean with water. Remember the toilets are for flashing and there are people who do not know how to use such toilets* |
|  |  |  | *Long ago, there used to be a heap of rubbish just near here, everyone would get whatever they have and dump it there. The child would want to ease themselves and the mother would dump there, even adults would throw feaces there and yet they are mature people. But right now, all that is history* |
|  |  |  | *What would I really tell you? Hygiene is very important for patients like us. All these infections are caused by poor hygiene, and they should teach people on how to prevent these infections by being clean at home. And for us who have pressure, they should tell us which foods to eat, which ones to leave and also tell others about how to avoid getting pressure.* |
|  |  |  | *Yes, the cleanliness of this hospital and the ward in general, it is a requirement that we maintain a descent environment, mop, make beds, bathe those that can’t sit, for example if a patient passes stool on the bed, we don’t allow the caretaker to clean him or her, the students must do it as part of the training. our students have done this work very well. They really show care to the patients as part of their training* |
|  |  |  | *There is also an infection prevention committee which is very active. It is active in the sense that the members of different wards have been sensitizing patients like on washing hands since there is clean running water.* |
|  |  |  | *There are some things you need at the emergency such as protective gears, sometimes we don’t even have gloves. If you look at the other side, you will also realize that some of them do not even have masks right now. When you facilitate me to work and given me the logistics I want, then I would also work well* |
|  |  |  | *So, hand washing is important. Another thing maybe to use alcohol hand-based gel and they should have it in their pocket, trolleys and in the wards so that after torching each patient to reduce microorganism* |
|  |  |  | *Infection control especially with hand washing because what we have seen here, health workers are not taking it very important that they should wash hands every time they are working on patients. Most times you find a health worker started working from the beginning up to the last time then washes hands. The same health worker may go home without washing hands* |
|  |  |  | *Yes, we tell them to prevent like hand washing because we always put water at the corner of the toilets. We tell them wash hands before eating or touching anything even after visiting the toilet. We also tell them to keep the nails short, leaving food covered. We tell them not to eat leftover food* |
|  |  |  | *There is also infection control because if this person is septic, sometimes there are people who are septic and the disease they have can be transmitted to another person so there is need for isolation if the condition she has is also infectious. There are some who have sepsis but not transmitted to another person.* |
| 1. **Shortage of water and power fluctuations** | Limited water supply,  Attendants carry water from home  Caregivers unable to bathe or clean their patients and to maintain hygiene  Fear of infections  Caregivers unable to change patients’ beddings when water is gone  No alternative light when power goes  HWs use torches to treat patients at night | | *Secondly, I expected that when going to bathe, I would find water, but I found out that water not available* |
|  |  |  | *I was carrying water in the Jerri…can from home to here. I put it in the jerrican and bring it here every day.* |
|  |  |  | *Other challenges are shortage of water because of no water for bathing, no water in the toilets, so I fear going there, in fact I can get more diseases.* |
|  |  |  | *Sometimes you want to bathe her and many times you can get the water from the taps up there as long as you want to wipe her or bathe her. But now you find that there is no water, it becomes difficult to manage the hygiene* |
|  |  |  | *Another thing is that even the water is not there sometimes, the water in xxx keeps disappearing. So, the water can even make you sick yet you are already sick. Even the toilets are dirty, when the water disappears, because some people don’t know how to use them you can suffer* |
|  |  |  | *The other thing is that sometimes there is no water, when you are about to start washing the patient’s clothes, water just goes. Now the water has gone, and some people have patients with severe diarrhea. And sometimes they don’t have other bedsheets. So, when water goes, what do you do? Those are the services I saw here* |
|  |  |  | *Except that there is one thing I have observed that is here; that when there is no power, they don’t have a generator that they can use when power is gone. They don’t have a standby generator. When it reaches night, and the power is gone, health workers treat patients using torches, that’s also a big problem* |
|  |  |  | *The same as water…eeh when water goes, people suffer. My son had to buy mineral water to be used to brush and clean me. In the end, you cannot use it to wash clothes and yet the nurses have been emphasizing that we maintain cleanliness. So, water is a big problem, you would see that everyone was disturbed. They should have water tanks so that when tap water has gone, they give us water in the tank.* |
| 1. **Concerns about Poorly maintained toilets** | Attendants responsible for poor hygiene  Use of hard paper, no water poured or no flushing  Toilets shared by both male and female  One entrance  Women’s privacy and security  Ease on top of the toilet  Patients asked to use basins for caregivers to pour in toilet  Pampers, papers, food, pads, polythne bags thrown in toilet by users  Patients buy buckets to help patients ease themselves and pour in the toilet  Fear of infections. Patients unable to use the toilets because of fear of infections  Cleaners are available, patients are taught how to flush but not adherent | | *The problem is that the hospital has made us a toilet but there are times when we the people using it spoil it. The truth is that the toilet is ever dirty because of us people. Those toilets are for flashing, they come and teach us that when you are coming to the hospital, you should come with toilet paper. After using the toilet, you use toilet paper and pour water so that the stuff can go. However, in the morning, you find that the toilet was cleaned in the morning but ourselves…you find that someone goes there with a hard paper without toilet paper and he places it on top of the toilet. You find that the toilet is blocked and those are not things done by the hospital but us* |
|  |  |  | *I still find it hard to talk about is that we were dirty we as care takers and the patients don’t know the word cleanliness. That one annoyed me so much because when I when I went to the toilets, I found them very dirty because even now I can tell you that it didn’t make me happy at all.: That what I saw that needs to educate the patients and caretakers about it because we don’t know about hygiene. Because you can’t be here in the hospital as a caretaker or a patient and the toilets are there and you go outside, that one annoyed me so much.* |
|  |  |  | *But the services that are here, we are badly off, there is one toilet, the rooms are broken, and the entrance is one like this. The entrance to the toilets for this ward is one. When you are coming out, you meet a man as he is also going to the toilet. Such a service causes unhappiness amongst patients and attendants like us especially women. Like any other human beings, women should have their own toilets and men should have their own toilets. Therefore, I have seen that, and I can say that the service of toilets is not very good. It is not nice at all generally* |
|  |  |  | *The toilets look so bad whereby people defecate on top and there is nowhere to pass. I even refused my wife to go there. I told her to use a basin at least and I pour the stuff in out in the toilet myself. That is the worst challenge I have faced. They smell, they are not cleaned and are in a terrible state!* |
|  |  |  | *You find that they have poured there pampers for children, they throw pads there, that means us the attendants are dirty, the truth is the cleaners of those toilets always come here and teach us on the ward. There is even a lady who usually comes here selling toilet paper and says that if you don’t have toilet paper, I’m selling some. But you get surprised to get out and go to the toilet and find that it is filled with polythene bags, someone gets the child’s stool and wraps in a polyethene bag and has thrown it in the toilet and yet they have just cleaned it* |
|  |  |  | *Yes, they should improve on the sanitation. Sanitation is very important. For all the time I have spent here as a patient, my husband stopped me from going to the toilets of here. It seems he went there and after going there he found that they were dirty* |
|  |  |  | *And my husband would give me a bucket so that I ease myself there, and he kept taking it to the toilet to pour it there. Even washing...I was not bathing because I feared to get more infections. I decided to wait and will bathe at once* |
|  |  |  | *Another thing is sanitation and hygiene…they want good hygiene to find a shade is cleaned because that’s where they sit when we are doing ward rounds. When we reach toilet, we know that they want to find it clean. But we have a challenge with our patients, they throw pads, papers, food in the toilet. We try to teach them how to flash, there are those that do those bad things. We have cleaners every day, but patients are the ones messing the toilets.* |
| 1. **Limited staff** | Not enough health workers  One nurse working long hours, every day, on weekends  When nurse is tired, she becomes unfriendly  Need to increase the number of health workers  Attendants’ wand nurses to work in short shifts  Few health workers mainly assisted by intern students  Each ward should have its own HWs  Health workers working in both male and female wards  Few HWs can’t handle multiple emergencies  Intern nurses cannot manage sepsis patients without senior nurses  There are few nurses at triage area causing a delay  Patient HW ratio affects the time HW spends on each patient  HWs not able to review patients on time  Many patients being attended to by one staff  Increasing knowledge on management should go with increasing staff  Individual staff working  Staff are overwhelmed, willing to give enough time to patient but cannot  Patients may not get appropriate care whether written or prescribed because they are few  Sometimes there is one nurse on duty handling two wards and therefore cannot give the best care  In extreme cases, intern nurses help  One nurse on duty not able to take observations  Heavy work overload, nurse cannot do observations as required  Clinicians are not enough  Patient sent to ward early but decision to admit is made late because clinicians are few and patients are many  Clinicians can’t see patient on time  Patient delays to start treatment  Staff not spending enough time with patients  HWs only interacting with patients when prescribing or giving treatment | | *It requires more health workers in that area. Sometimes, you find only two nurses, whereas one is registering patients, that is not so important but you find that the number of patients waiting to go to the room to see the doctor is very big and so you delay. So after delaying there for hours, they sent us to the clinic for patients with pressure* |
|  |  |  | *There should be enough health workers, the health workers are not enough right now. Like since I came here, there is that nurse that I was talking about, she has been here. I sometimes realize that she sometimes shouts at people, but it could be because of tiredness. Even since I started coming here, that sister is the one who spends the whole day on the ward. In many cases that sister is on ward and if am not mistaken even on some Saturdays. She sometimes comes many times and she is the one who is always on the ward in most of the afternoons, very many times ever since I started coming here* |
|  |  |  | *If the person has worked since early in the morning, she should at least stop at mid-day but when she comes in the morning till evening. The one who comes in the evening works till the following morning. That sister stays there with those children {students} and she goes in the evening. The one of evening comes she stays the whole night with those students, and she goes in the morning. Let them at least work till mid-day, the person of mid-day stops at six like that till morning. But I see that nurse, she really gets tired. They should add us health workers* |
|  |  |  | *I want to insist on that thing of health workers, they should be increased, they are needed because you can see that they are two and may be each doctor can handle cases of one ward. The female ward should have its own doctor to handle patients in waiting and for the male ward.* |
|  |  |  | *You have to first see the doctor; they assess you and then they give you a number. We all get numbers but at times there is when there are many patients compared to doctors’ available giving numbers* |
|  |  |  | *I think it is because of the few doctors working there and the truth is that there are only two student nurses; they are measuring the heart, the pulse, asking you questions* |
|  |  |  | *Now just imagine I am talking about patient caretaker ratio, or one clinician has to review 40 patients in the ward and each review will take ten or more minutes, yet he started at 9 am because you have other procedures to do. By the time he reaches the last patient, the time for administering the medicine is gone. You find much as I would like to move with a physician to review a patient and listen to what care we are supposed to give, again I must put it in mind that after that round, I have to give treatment whereby it has to be timed. You will find I have few staff and most times we do not finish on time. The patient health provider ratio is too low.* |
|  |  |  | *Still human resource remains a big challenge even though we improve their knowledge because you may find a situation whereby a ward has so many patients and being attended to by one staff.* |
|  |  |  | *The other challenge is the place is understaffed. I can do a ward round in the morning, and I finish, and another staff will come at around 11am of one staff on both wards.* |
|  |  |  | *I would say the staff in the ward can I say they are few sometimes when you are overwhelmed with patients as you try to see everyone you may not see everyone. So, I would think the staffing even if you have very many and you want to do something for this one and then got to the next but if they were enough, it is easy to give time to a patient.* |
|  |  |  | *The stuffing at least if two health workers on a shift, quality care will be given since this is a big hospital. If they get one worker on the male side and the other on the female side and one in charge of the inpatient side, then things will be okay. But if there is one worker, one person cannot give quality care to everyone on all the sides. That is why I think that if the staffing is adequate then there is improved quality care.* |
|  |  |  | *Another challenge maybe staffing whereby you find yourself on duty alone yet there are supposed to be 3 to 4 nurses on the shift because I said we have two wards and at least each ward with two nurses. At times you get surprised, and you have to handle all this patient alone, yet our admission capacity is 40 patients. Can one nurse give the best care to patients?* |
|  |  |  | *I would think health workers only relate with the patients when they are prescribing or giving treatment. But I see the need for health workers…may be just spending more time with the patients explaining to them the condition because I noticed since our recruitment is a bit intense like the first recruitment, we really do a lot for the patients then us reviewing them daily we get an attachment, we build rapport. Sometimes the patients tell us. Like one patient told us, I wish you were the primary care team”. Because for us the study staff, we spend more time with patients explaining, reviewing them daily even when we take off samples until day three but still see the patient needs the discharged since we see them daily, ask them questions they tell us more than they are supposed to tell us instead of telling the primary care team.* |
|  |  |  | *The other health worker you will see is that nurse coming to give medicine and it may take five minutes meaning of the 24 hours the patients the time she spends in the hospital, the maximum contact is one hour on these several intervals when nurses are giving medicine and 5 to 10 minutes the doctor gives you when they are seeing you once a day in the morning.* |
| 1. **Staff absenteeism** | Roster not being in harmony  HWs on the roaster yet they are a way for leave  Need to improve the roster  Absenteeism of key staff without reasons  Less commitment at night, patients may die  Nurses refuse to wake up and help patients  HWs asking their colleagues to cover their work while absent  Pretending to be sick  Hard to get a service or replace critical human resource if their services are needed and are not around on some days. patients must wait till such Staff comes back | | *The issue here could be on their duty roaster. Some of them you find they are on medical studies, annual leaves, sick leave, maternity leave, etc. You may find so many but ideally on the ground they are few. And if you are to ask how this can be improved, I think management can ably ensure that the rosters are followed.* |
|  |  |  | *The shortage is mostly on the side of nurses and not doctors. Actually, I do not know how they make their timetable. Sometimes you find all the nurses around, sometimes you find one nurse is working both sides so usually their timetable is not regular like it has certain formula it follows because sometimes you will find they are many, the other time you will find only one is around* |
|  |  |  | *There is a very big gap in this ward. Even a team leader can decide to be absent without any of the above reasons. So, patients not only miss treatment, but others also die because at night, there is almost no commitment. Patients, including the septic ones will tell you in the morning that they were nit helped. You find a patient needed oxygen at night, but the nurse was sleeping and refused to wake up…and some of them die not because we didn’t have the capacity but because of that problem I have talked about.* |
|  |  |  | *This is because you may find that the staff are not sick but asking their colleagues to work for them something like that. So, someone is absent because they are doing her work outside the hospital. So, one friend agrees to do work for two when they cannot afford and the person who suffers in the end is the patient.* |
|  |  |  | *At times they may not be around. We do not have so many on the ground for example if you have one senior laboratory technician and when he is not there, you cannot replace his expertise. We have senior gynecology but if he is not there, the medical doctors cannot do anything. It affects in that their absence the patient’s condition worsens. When they are around, everything is smooth* |
| 1. **Shortage of equipment. Thermometers, BP machines, Pulse oximeters, and catheters. In short supply.** | No syringes, cannulas  Attendants buy for their patients from private pharmacies  Shortage of patient forms, patients provide own books which are sometimes used as patients’ medical forms  Oxygen was available and given  Reagents, cannulas giving sets in shortage.  Patients asked to buy their own  Vital signs not monitored regularly  Limited staff to monitor  Shared equipment  Vital signs not monitored as recommended-few nurses, 1 shared BP machine, 1 thermometer, pulse oximeter with no batteries. One BP is shared between wards  Many patients need oxygen at the same time and cannot share oxygen cylinders  Patients who need CT scans are referred to xxx  Hospital has equipment to do several tests but occasionally runout of specimens  No needles to pick samples, caretakers asked to buy own syringes, takes time to come back  No reagents in the lab, attendant asked to go back to pharmacy to get own reagents  Most units lack equipment; no thermometer, BP machines, sometimes one thermometer is shared between wards  Shortage of cannulas  Shortage of stationery, gloves, masks, sanitizers, patient forms  Patients provide own books which are used as in-patient forms | | *They put her on oxygen, because she had something that would come and block her and she would struggle to breath like a child who has asthma, you would hear that sound like for an asthmatic patient. So, they would put her on oxygen, she would continue breathing like that until she stopped. Whenever it would stop; they would remove the oxygen. When it starts again, they bring it back and put it on her again* |
|  |  |  | *They were only telling me that they don’t have the cannula that can draw off blood. They first took the patient in the room before they started to work on her. So, I said let her be sleeping there, and then I went and asked the doctor that how much are all these things going to cost? He said that you go and find out, so I went and bought them from the pharmacy outside the hospital.* |
|  |  |  | *And the hospitals also…a person comes and is sick and he or she is also buying the forms, it seems they also don’t have hospital forms for using. We come with our books, that’s where they write all the information for the patient. They pluck papers from the patients book and they use it as a patient’s medical form* |
|  |  |  | *When we went to the hospital with my mother, they first sent us to the scan, there was no scan in XXX. They called someone from outside, he came and picked us on the boda boda. That health worker connected us to a certain scan outside the hospital. We went out and did a scan and brought the results.* |
|  |  |  | *The second time after doing the scan, they said that the heart was swollen, they also wanted to understand the size of the heart and how big it was. They sent me to the x-ray of here. When I reached there, they told me that the x-ray did not have a film. The health workers there asked for 20,000 to go and buy the film. I paid, they took the x-ray, and we came back. And as you can see, they are all necessary for patients like my mother; x-ray, scan that they are talking about are at hi-tech not in xxx. I think it is because they have a lot of work during the day, but us who never went to school have nothing to do! Just imagine that xxx lacks simple machines like x-rays and scans.* |
|  |  |  | *For the other tests, they said that they did not have the reagents, all those things were for buying on my own. For example, you have to buy cannulas, giving sets for the drip, and even drugs that they injected in my hand, we just bought them the following day, all those things that they use to put someone on a drip, we just bought them. It’s only the drip [water] that we didn’t buy, the sets were bought by us.* |
|  |  |  | *The machines are not there…they are not working; they should put the things which they put to test. I heard here my neighbors saying the machines are there, but there are things you have to buy that they use on those machines.* |
|  |  |  | *Sometimes, there syringes are finished, and they tell you to go and buy then. They usually tell you that syringes are not available, they tell you to buy them; when some drugs are not there, they tell you to buy them* |
|  |  |  | *Sometimes oxygen is not all that enough since we have very many people. At times in a day, you can receive like 4-5 patients who need oxygen and yet you have two-cylinder heads. In the end you find one person missing the care of oxygen. However, much you run around to get one, on such case you give up and surrender to God. Because you will want to change from the one, but you find that person is also badly off. In such a moment you just remain there* |
|  |  |  | *Yes, and most times have challenges because we do not have CT scans. When we get such patients, we also refer them to xxx but the moment they are referred here they know that it is a government referral and has everything, yet it is not the case. Those who need urenodialysis, with kidney problems, we also refer them because those services are not done here.* |
|  |  |  | *We do urine gram style and identify microorganisms. We also do spectrum analysis, the gene x part. We were also doing culture and sensitivity, but the machines are down now. We can do CBC. Occasionally the specimen runs out. Those are the only little time when we do not have to investigate* |
|  |  |  | *Now this is where you find you want to pick a sample, but you cannot find a needle. You order the caretaker to bring a syringe; he moves the town and spends there one hour. When he comes back, you do the samples on the patients. When you reach the lab, they tell you that they do not have reagents. Then you said the send the same caretaker to town to look for it in a pharmacy around.* |
|  |  |  | *Then also sometimes there are stock outs in the hospital. stock out of sundries, to some people when you have very sick patients even a cannula. Seriously, a giving set is missing* |
|  |  |  | *The other challenge is like any other hospital, stationary sometimes we work without gloves, some time we work without masks, sometimes we work without sanitizers, sometimes patient buy books or papers where to write the patient notes so they must buy books even right now we do not have stationery and it is a very big challenge.* |
| 1. **Non-functional laboratory equipment** | Machines available to carry out tests but faulty  Lack reagents to conduct tests  Hospital laboratory performs very limited investigations No Liver function tests,  Yet patients do not have money to go to private laboratories  Unable to prescribe appropriate treatment  Affecting improvement or recovery of the patient  HW thinks lab investigations should be number one priority  Patients treated based on clinical observations  If tests are prioritized, HWs would be in better position to give correct treatment  Treatment is started blindly  Fear that it might cause microbial resistance  Not able to do culture tests  ARCS paying for such tests for its patients  An expensive test | | *We have investigations which include the lab tests. We have radiology investigations which include X-ray, ultrasounds. We also have physical examination and general assessment by the doctor, nurse, or clinicians. We have observations like temperatures, respirations. We also have drug treatment.* |
|  |  |  | *Of course, with lab investigation, out laboratory here performs with very limited investigations for example if patients want CBC, most of the time our hospital cannot do it. There are no reagents. Sometimes for the culture and sensitivity are rarely done and, in some reports, they have given that they have limited drugs. This is because for culture and sensitivity you need a lot of drugs but here, they have limited drugs to test with. There is not liver functional test, no electrolyte tests* |
|  |  |  | *I earlies said that we have a good laboratory, but reagents are a problem and your organization and IDI in this study should really help with reagents. The machines are there; the staff are there but shortage of reagents.* |
|  |  |  | *These days we have not been doing investigations like LFTs (liver Function tests), CBC on because our machines are down. These investigations take a lot of time because you will get a patient who is critically sick do CBC, LFTs, RFTs but the patient has no money to go to a private laboratory to be investigated. There are others who die before they are investigated. Yes, that’s what happens.* |
|  |  |  | *Yes, one of them is the drug and then the other is sometimes our lab break down. When it is completely broken down and nothing is being done in that lab so and sometimes the investigations are very expensive, and patients cannot afford them and it becomes a challenge. Yes, if you cannot investigate someone, it means you cannot put up a right diagnosis therefore, you cannot prescribe the appropriate drug and the patient cannot heal* |
|  |  |  | *According to me, I think lab investigation should be the first because it guides us to the real diagnosis. Sometimes we have treated this people basing on clinical observation. Sometimes the antibiotic given, it is not the correct antibiotic for it. If we do observation, lab test, x-rays in time, we shall be able to give correct treatment to the patient. Investigation should be our number one the drug administration second.* |
|  |  |  | *The other is that other resources like sundries, medicines they are not available and in line with that also investigations like testing blood or doing these things of tested tray some of these things are not available here. Hey, also delay the decision to start treatment. So you have a case you just want to investigate here and there and confirm what you want to confirm to come and start treatment so in some situation you start treatment blindly when you are not sure because that is how we get histories of microbial resistance.* |
|  |  |  | *If the hospital labs would be free for the patients and if not free at least available because one of the investigations, we really wanted was patient blood culture. If I am not mistaken it is ARCS doing it for the patients. I am imagining those who are not in ARCS study do they real get this culture done? I think now because it is an expensive test.* |
|  |  |  | *I think this is to do with the labs would…if the hospital labs would be free for the patients and if not free at least available because one of the investigations we really wanted was patient blood culture. If I am not mistaken it is ARCS doing it for the patients. I am imagining those who are not in ARCS study do they real get this culture done? I think now because it is an expensive test* |
|  |  |  | *Then I talked about lab, we are in crisis if we can’t do LFTs and CBCs, it means we are not properly identifying sepsis. And yet it may be common. I can tell you that most of the patients here are anemic, have severe malaria, pneumonia, cardiac failure, all these lead to sepsis. Some of them we are just managing clinically, we don’t test. You see a patient dying and just treat* |
| 1. **Shortage of medicine** | Drugs for malaria pain killers available  Attendants told no drugs, later gets the drugs  HWs wanting to sell hospital drugs  One attendant received all drugs for free  Feel HWs don’t want to give patients free drugs  Attendants feel disorganized when told drugs are not there  Feel that the only free service is a bed  Want enough drugs stocked  Buying own drugs is painful  Patient wait at the pharmacy and later told that drugs are not available  People wait for long hours to be told to buy their own drugs yet they cannot afford  Patient thinks that availability of drugs would increase HWs confidence to communicate with patients  Complaints that critically ill patient had no time to go look for drugs. One patient gave an example of a fellow patient who died on ward after failing to raise money for drugs  Few people are lucky to receive free treatment  Patients promised to get free drugs when available  Drugs always available but shortages too  Patients are informed about the drug shortage and asked to buy their own  No drugs are kept for emergency patients because HWs don’t know when the emergency will occur  A delay from the time prescriptions are made and the time when patient starts treatment  Many people are poor and find it challenge to buy won drugs  Sepsis drugs are sometimes expensive  shortage of drugs affect patient improvement  Patients can be supported with management decision  Shortage of IV fluids.  Not able to give fluids as per the required standards  HWs get the right diagnosis but do not have control over administration of drugs  Some types of drugs not supplied by the hospital-HW has never seen one type of drug for sepsis  Patient can die before starting treatment  Delays in funding and dispatching of drugs  Patient can take drugs available in the hospital even when they may be resistant to microorganisms | | *He also asked me that do I have the medicine for the patient and I told him that I don’t have it. He told me to buy another set of drugs* |
|  |  |  | *There is a time when I went to pick drugs from the pharmacy and they told me that the drugs were finished, because they wanted me to give them money. I stayed there and waited, when another health worker came, he asked me and I told him that I’m waiting for drugs but they said they don’t have them... These health workers… can confuse when he saw my paper he said the drug is there yet the first one said that it is not there.* |
|  |  |  | *II went to the hospital with hope that everything that is related to treatment services will be there. I thought even blood will be there and all other things will be given without me touching my pocket for money even drugs I thought were for free that’s what took me to XXX hospital but what I found there with issues of buying drugs,* |
|  |  |  | *That the health workers ask for money so that they go and buy drugs for your patient, they pretend that the drugs are out of stock. But I’m personally thankful that we did not buy any drugs, and there is no person that asked us to give money. This work was very easy for me because I did not find anything difficult, everything was very good for me.* |
|  |  |  | *I was expecting drugs that are being used to treat my patient to be provided by the hospital for free, because this is a government hospital. But what is here; if they want this type of drug; “go and buy it”. Yet I don’t have the money but I want my patient to recover. Because asking me to buy drugs requires me to first go back home and first mobilize money, then come back and buy the drugs for my wife!* |
|  |  |  | *What has been happening on the ward, we have been buying our own drugs, they tell you bring this drug and you go and do what? And bring it. All the drugs that they have been injecting her, we have been buying them. We have been buying everything except fluids, those are the only ones that we are not for buying, we have been going to the pharmacy to buy all the drugs the pharmacy is inside the hospital, that is where we have been buying them* |
|  |  |  | *Now they refuse to give the patients drugs and we suffer yet the drugs are there. Even the other day I was there because of this boy they wrote for me six bottles of drug, and every bottle was for 4,000shs but there was another girl who also got it for me. The medicine is there in the store and they tell me that it is there.* |
|  |  |  | *The most important thing is that if you haven’t bought the drugs they asked you to buy, they were bypassing you without treating you. But if you have your drugs, they were treating you. If you don’t have drugs, they bypass you and go to another patient. What they do, they tell you to go out of the hospital to buy drugs, they prescribe them for you and you go and buy them, when you bring them, they come and treat you* |
|  |  |  | *When the drugs are there, even you the health worker, you feel happy even when you are going to talk to the patient, you feel that you have taken good news to him or her.* |
|  |  |  | *Sometimes, if you are lucky and you have come when drugs are there, you can still get the drugs for free. But when they ask you and you tell them what you do, that’s when they tell you the things are for buying. Next time, I will come here begging and see if they won’t test me for free!* |
|  |  |  | *At that time, they had not done anything on me. They said that since my legs were swollen, they will start the treatment. However, there was a drug that I had to buy so that it can help me to reduce the swelling in the legs. There were other drugs that they wanted me to buy so that they can start treating me…all these were done even before seeing the clinician* |
|  |  |  | *There is a challenge of drugs since at times patients come when there is no medicine, others miss them. Most times we tell them that once we have drugs, we shall give them. When we do not have, we remind them to go get from outside* |
|  |  |  | *We are also doing well on the antibiotics though not very consistent. When the government sends us medicine, we distribute it to patients. When it gets finished we tell patients to buy. So we don’t sell drugs, patients don’t pay consultancy fees.* |
|  |  |  | *There are not so often. When we have drugs, they are always there. But there are cases when we have a shortage then that is when we tell them that we don’t have drugs so they should go and buy outside* |
|  |  |  | *Or even sometimes prescriptions are made but there is a delay maybe patients do not have the money to buy medication. Even stock out of medication because this is why they come to a government hospital because they assume they will get all the treatment here. So, sometimes we even see patients coming to use asking us can to also give them treatment. But remember, as a study, we are not administering any medication then they ask us apart from doing investigation for us, can you give us also the medicine?* |
|  |  |  | *There is a challenge of not having IV fluids and at times even if you admit a person with sepsis. These sepsis patients need like two liters of IV fluids in one to two hours. You may have the right diagnosis and everything but no IV fluids. Most times we try our best.* |
| 1. **Unpredicted consumption of drugs** | Patients much more than predicted  Delay for national supply  Under budgeting for the local population  Increasing population  Supply for emergency drugs should tally with the demand on ward | | *It is quite often to have stock outs because of unpredicted consumptions that we have in the region. The population is ever increasing. They predict this and we get overwhelming.* |
|  |  |  | *Well on many occasions thing stock out due to the number of reasons such as the supply because at times there maybe national supply delays to come or maybe there may have been under budgeting due to an increasing population.* |
|  |  |  | *Because as you budget for the local population, remember there are also refuges coming in and this is a referral hospital whereby you will get IDPs being referred here by their management, and you have to receive them* |
|  |  |  | *The first month and second we are okay but the third month, things are totally not there. Things usually run for two months because of the overwhelming numbers. Over population here also affects the supplement because it is dynamic, not stable. We get refugees, and so on.* |
|  |  |  | *And IV fluids are also not enough most times, even ceftriaxone, even other drugs, patients are too many as you can see today. So they should increase the volume of emergency drugs like 50% dextrose according to the population that comes here and need emergency drugs* |
|  |  |  | *But it’s a challenge, patients are too many, one consignment lasts for a month and it’s over. So it’s a big challenge, seeing a patient die because they failed to buy drugs and you the nurse can’t also help them!* |
| 1. **Costs associated with care for sepsis patients** | Worry about cost associated with buying drugs, conducting tests due to lack of equipment and drugs in the hospital  Attendants pay money for test to HWs  Costs like those in private facilities  Blood sometimes is paid for yet free  Patients who cannot afford to pay stay in the hospital longer  Costs between 30,000 to 45,000  Delay by the attendant to purchase the drugs  Delay by the HW to administer the treatment | | *The first time when we had started coming, they used to inject her about three types of drugs, they would ask for 42,000 if you have gone to the pharmacy. That 42,000, the drug would be injected on her in the evening, so you spend a lot of money* |
|  |  |  | *Like yesterday, they took blood samples from her, they asked us to pay me to repair …the machine which was down…the one which tests blood. They told me to go out but I told them that am alone in the ward. And as such, I cannot manage to go to take blood samples where they should be tested. So when they said that the machine was down, I asked how much we should pay for the tests from outside. They said 50,000. We gave him the money, and the samples. He said that the results are out and are written in the reports. The health worker came to me and said that he went to the lab, they tested the blood and the doctor would be telling us the results from that test. After that she went home.* |
|  |  |  | *The first time, I paid fifty thousand shillings, the second time, I also paid fifty thousand shillings. And the last time, when they told me to test for an infection, I paid thirty thousand, there is a time when I paid fifteen thousand shillings* |
|  |  |  | *The money that you are spending…you find that you have spent similar amounts like the one in the private hospital. Because just carrying out the tests…since they told us to go and test from out, we go out and test, then we come back and yet the patient is admitted here on a government bed.* |
|  |  |  | *Not me alone. My God may people were suffering on the ward. What I observed is that most people on the ward found it difficult when it came to spending money. Even last time I told you that there was a woman on my right side, that lady was first admitted in gynae, they failed to get money to treat the patient, they stayed in the hospital without treatment and unfortunately, their patient passed away* |
|  |  |  | *Ah that word reads “blood is not for paying, it is free”. But the health workers honestly asked that woman to pay money for blood. The lady said that she didn’t have money. Previously, one of the daughters had paid for the first bottle and it was now finished, and they wanted to put more. The health worker came and told her; “bring the money”. The woman came and asked me; “What should I sincerely do? But I used to talk to the health workers quiet often because I was easy for them, I would pay for everything they asked for* |
|  |  |  | *You pay 25,000 for a film because the hospital has not considered x-ray as an important thing for the treatment of the patients. After the x-ray, the hospital cannot even afford envelops for keeping your results. They also tell you to go and buy the envelop for putting in your x-ray results and it is worth 1000. You have paid 25,000 for buying the film and after the x-ray, he tells you that he has no-where to put it. They sent you to the hospital canteen to buy the envelop!* |
|  |  |  | *Yes, it includes sepsis patients and like I said, they stay longer. To be able to do certain tests to confirm severe infections, you need to do LFTs and CBC and machines are down, so they can’t afford. So they stay longer. Those with pneumonia, severe anemia, and other complications require advanced tests* |
|  |  |  | *Eh it is a lot. We normally give them medicine for two weeks each varies around 30,000 (thirty thousand) and in total they can spend like 450,000* |
|  |  |  | *And so, if patients are coming here and they are still paying for tests, they will be frustrated because the service is not free as it is supposed to be generally for the hospital even the drug also absent. Even when prescribed there can be a delay, the nurse has not administered or there is a delay they have sent the patient for the drugs maybe they have not brought it timely, or they cannot afford them.* |
| 1. **Referral to private hospitals and pharmacies and laboratories affect patients from poor households** | Testing equipment in the lab not functional  Patients asked to test from private facilities  x-ray, ultrasound  Drugs are purchased from private pharmacies  Attendants leave their patients to go seek services outside  Costs not different from private hospitals  Feel pain about paying for services outside  Sometimes not told what is being tested  Worried that the patient can die while seeking lab services outside hospital  HWs have their own preferred private labs and pharmacies  Patients have less choice regarding where to purchase drugs or do the tests  Attendant bought a film for x-ray at the hospital  Patients asked to purchase drugs from outside pharmacies  Patients advised to go to private facilities to conduct tests such as CBC, LFTs, X-ray, Ultrasound, and other tests because hospital machines are not functional  Tests outside the hospital were considered costly in addition to transport costs  Patients cannot afford to pay for the tests outside the hospital and fore go some of the tests  Patient cope with the issue of buying drugs  HWs have nothing to do when there are no emergency drugs  HWs advise caretakers to move to private pharmacies  Patients/attendants don’t like to be told to go and buy  Samples taken outside delay to come back  Patients/attendants do not want to listen to issues of stock outs  Patients/attendants think services should be free  Wanting to bribe HWs to get drugs thinking that HWs aren’t telling them the truth | | *Even when it comes to testing of blood, they tell you that the hospital doesn’t have machines for testing blood. They give you a form and they send you to xxx that’s where they will test you from. Eeh because the last time to test her, they told me to take blood so that they can test for infections. The second time they told me to go and test for the kidney. Sometimes, they just give you a form and they tell you to go and test and whatever comes out, you will go and ask the doctor. He is the one who will explain to you the results. So we test this and that but the hospital tells you that they don’t have the equipment to test. Whenever they send you for testing, it has to be xxx and yet xxx is for money* |
|  |  |  | *The drugs that we buy…we buy them from the pharmacy up there. That’s where we have been buying them. Every drug, we almost buy all of them we just buy.* |
|  |  |  | *Because we had already spend a lot of money on those tests especially the scan. So we went back, they worked on us freely in the scan but the x-ray was not working and we did it from outside. When we went to get x-ray outside the hospital we paid 25,000 at doctor’s clinic* |
|  |  |  | *We buy medicine from the pharmacy there. There are other pharmacies outside the hospital but because they always want the drugs very urgently, you buy it from that one [points at the[pharmacy]. You cannot leave the doctor standing on you patient and you insist on going to the buy drugs from outside the hospital. You just have to leave him there with your patient, you rush to the nearest pharmacy that is within the hospital, you bring drugs and they treat your patient* |
|  |  |  | *In a private hospital health workers care for you, they even tell you that this and that will cost you this amount. Or they work on your patient and they give you a bill and you pay it well knowing that you took your patient to a private hospital. What is surprising is that when you are in a government hospital and the patient is critically ill and needs much care, you are not different from the one who went to the private one. I felt very bad about what happened because I did know that such a thing would happen.* |
|  |  |  | *When I heard how they were explaining the situation that is in that hospital, I remembered the experience I went through. That thing of taking us there to be tested, I would feel that it was making me run mad! They would tell you that go right now, they collect your blood and they tell you to go and have it tested. Unfortunately, they wouldn’t even tell you what they are testing. They would ask you to do the same thing the following day and I would still go and do the tests.* |
|  |  |  | *The health workers there asked for 20,000 to go and buy the film. I paid, they took the x-ray and we came back. And as you can see, they are all necessary for patients like my mother; x-ray, scan that they are talking about are at hi-tech not in xxx. I think it is because they have a lot of work during the day, but us who never went to school have nothing to do! Just imagine that xxx lacks simple machines like x-rays and scans.* |
|  |  |  | *The second time after doing the scan, they said that the heart was swollen, they also wanted to understand the size of the heart and how big it was. They sent me to the x-ray of here. When I reached there, they told me that the x-ray did not have a film* |
|  |  |  | *The doctors will tell you that the scan machine is broken down, it is not working. You don’t have anything you can do except to go out and have the scan. Because me when I was here, I saw almost everyone carrying out their tests from outside clinics and I wonder why they can’t buy or repair those machines,* |
|  |  |  | *It was hard for me to get a scan, they wanted a scan to see what is inside, but getting the money that they needed was a hustle and the money was too much on me.* |
|  |  |  | *They didn’t have a scan; they didn’t have drugs. I also saw other people being asked to go and do certain blood tests outside that the machine was broken down, but it was not me. It was other patients on the ward* |
|  |  |  | *Because when we have it, we also give. We give drugs after checking around and see if the case needs the care which we give and the patients goes back home. Patients do not normally complain so much especially during my duty. Some of them complain that there is no money but normally they go and buy. Though there are few who luck but with time they also cope up* |
|  |  |  | *What happens is we normally run around to check from the emergency where adult departments but sometimes we even fail to get. Where we get, we support the patient. Where it is not there completely, there our hands are tied up and cannot work beyond.* |
|  |  |  | *If it is not there, you come back to the patient and discuss means; that this is what we wanted to give you because it is the choose for your treatment however it is out of stock. There you advise the patient or the care taker to move to a private pharmacy of his or her chose and she buys. When they bring then we start the we start the care* |
|  |  |  | *Once you give them medicine that brought them, they are happy. What they do not like is to tell them to go and buy medicine yet for them they know that they already paid taxes.* |
|  |  |  | *These people do not want to listen to what we call stock outs. What they know is that they have come to a government facility and everything is entirely free so telling them to go in a private pharmacy to buy is like you are hiding medicine and you want to sell it which is not true. Some of them can tell you that “in whispers” can’t I give you something and you give me this thinking you are just framing from him. You find hard time to explain to them that there is entirely nothing* |
|  |  |  | *Even when you ask for tests they have to pay for them from outside the hospital and some people do not have money really. They come back crying I cannot do this test* |
|  |  |  | *You see how to go about it without tests like now the people we see in research they are happy because all the tests are done. They are happy because all the tests are done for them, decision to treat and the quality of care is better. They do not go through the hassle of doing all of this because of the several tests we do for them, and you find for them they are seen timely. Us in research we are there full time okay, most of the time and we also treat them clinically as well. So you find at least for them they are cared for they look to be special or different from the others.* |
|  |  |  | *Okay, I have seen them usually when they have a poor patient who cannot do any test. I have seen them trying to lobby us, “can you recruit this one because that is an indicator to show that they are happy about the sponsored tests and investigation so that is an indicator”. Another indicator is that some of them call us when someone has just come, “would you mind if they can be recruited”.* |
|  |  |  | *This patient had cellulitis, which is another form of sepsis, yes I told them to buy diapersilim but could not afford it she is called a social worker. So I informed a social worker they were able to get her ampicillin. So, usually those patients who cannot afford, we try to inform the hospital and social worker, they come in and help if the drugs are not within our pharmacy* |
| 1. **Limited number of ambulances affects and delays referral to hospital** | No hospital ambulance to rescue emergency patients | | *Then another thing is ambulances, the ambulances should be there so that if you get a problem from anywhere, they can come to your rescue.* |
| 1. **RELATIONAL ASPECTS OF CARE** |  | |  |
| 1. **Perceived poor attitude towards work and towards patients among some HCWs** | HWs not responding quickly when called upon (few staff)  HWs not helping without being given some money  Receiving care requires endurance  Patients think that some nurses are lazy  Nurses communicate rudely to patients when called to help or when they ask questions  Nurses refuse to talk while attending to patients  HWs attitude shows lack of commitment to work  Staff refuse to wake up at night when patients need help  Staff need training in customer care  Some HWs are rude, absenteeism  HWs unable to give clear information to the patient | | *Yes, there are some health workers like the recent incident whereby I called the nurse to come and remove the drip and he told me that he was busy. I then requested another nurse who helped me.* |
|  |  |  | *But there are those when you come, they look at you like rubbish and they just by pass you. Now that means what? Even if you go and plead please doctor help me my patient is very sick he will just turn his back and go.* |
|  |  |  | *There is nothing you can talk to the health workers about if you do not have money. All you need from them is medicine nothing else. They do not have time for other discussions.* |
|  |  |  | *But haven’t I told you that the doctors just come and bypass you and turn their back on you and even the patient they turn and go. Now if you have seen this one and you have just by passed, you see another one you also by pass them now haven’t you turned your back on them? Wouldn’t you have despised me?* |
|  |  |  | *But that was that day he never came back I didn’t see her again, but the other ones were not caring* |
|  |  |  | *The truth is that things here are for endurance because is if you are not patient, someone can say to you; “if you think you are not cared for, carry your patient and take him or her”. The health workers of this very hospital said it to me one day that if you think your patient is not cared for, carry her and go away. I ended up saying to myself; “where else will I take her?”* |
|  |  |  | *One of the health workers answered me with a very high tone and a lot of energy and said; “Do not disturb me. I’m working on this patient that is in a very bad condition, he is not like you”. Yet I also felt that I was very sick and if I spend a night without treatment, I may become very sick. But she answered me with a high tone and I even failed to get what to answer her!* |
|  |  |  | *But there are also these ones, they come and treat you without talking to you, you call her to tell her that the drip is used up and she just looks at you like that. We also had those ones on the ward.* |
|  |  |  | *Not only that, you can clearly see the issue of attitude, we lack commitment. We are always complaining, I think someone imagines her colleagues sleeping and she or he is here suffering with patients and nobody is supervising them. In the end, she sleeps, she refuses to open when called. There are staff here on top of refusing to wake up, they should, they grumble, they absent themselves, but given that we are all government workers, that’s the situation we are in.* |
|  |  |  | *But what I also suggested earlier is that they should be trained in customer care…in that way, they will reduce delaying the patients* |
|  |  |  | *And of course bad ones are also there, they come with problems, they are sometimes rude, they absent themselves, pretend to be sick, am sick, oh my child is sick, oh, I have lost someone. All those are there. But generally our relationship with patients is not that bad.* |
|  |  |  | *Because what they complain about is, “Eeeh that person is rude”. He cannot give you clear information when you ask him, they can’t tell you what the patient is suffering from”. So what they need is good communication from health workers. Someone just goes to the patient “go and buy that drug”. How do you expect the patient to understand? So they need explanation* |
| 1. **Friendly and caring and hard-working HWs. Good communication and relations between HWs and patients** | Feel that their patients were well cared for by HWs  HWs were sympathetic, able to communicate and took time to explain to the patients and check understanding  Nurses and doctors act quickly but ask for money when patient gets better  Few HWs attend to patients on weekends, doctors and nurses are limited on weekends  Some patients not reviewed on weekends  HWs are quick when called upon  Good care by HWs  HWs not getting tired of treating patients on ward  HWs Responsive when called upon  HWs quickly attend to critically ill patients  Appreciation that HWs took good care for patients  Doctors are committed, the do ward rounds to the end, till late in the evening  Cared for the patients  HWs get tired  Behavior of attendants make HWs tired too  Attendant wants HWs to be increased  HWs are friendly to patients and attendants  HWs tell the attendants to change patient’s drugs  Attendants being obedient and following instructions of the HHWs  HWs treat patients very well, HWs sent fellow workers to buy drugs for patients  HW encouraged attendant that patient would be okay  Patients feel free when HW is the one on duty  Patients ask for a discharge because favorite HW is going off duty  HWs try their best to build rapport even when investigations cannot be done or when drugs are not available  Patients thank HWs for treating them  Discharged patients call from community to inform their HWs of their progress  HWs re-assure the attendants that the patient will be fine  Patients would buy drugs as long as HWs talked to them well | | *There was also another doctor who would come at night and check on her. He would ask whether she has received the medicine or not. And one night he told us that this patient has received her last drip. One day he asked me that has your patient received her last drip, I said no, and he told me to go and tell the doctor to come and put her on drip.* |
|  |  |  | *All the health workers cared for us very well because the one who cared for us in the afternoon is not the one who cared for us at night. At night we had other nurses but they were caring for us very well. They come and ask us how the patient is, we also explain to them and they also assess the condition of the patient and the health workers also feel happy because they have done their work very well. But the good thing is that when you are taking care of a patient, health workers are sympathetic. Because you would start taking care of your patient* |
|  |  |  | *I would ask that how do I do this and that? I haven’t understood, how do I go about it? They would explain to me and sometimes they say; if you have not understood, please ask the nurse or doctor. So I would also stand up go and ask any nurse that I come across, I would ask; “Nurse, I have understood this one”. And the nurse would explain for me.* |
|  |  |  | *When you reach there the nurses and doctors care about you very quickly but when they finish in the end that’s when they ask for their money. But they are really quick to care for you. So when you reach on the bed, they really hurry to attend to you but when it comes to weekends, the person can even die. You find the nurses are not there, the doctors are not there, they are just there the way you bring in the patient, is the way she/he will spend the day there like that, even on Sunday it’s also like that. But on Monday, no I cannot accuse them falsely no.* |
|  |  |  | *Every time you go there that drip is finished, my patient is badly off or my patient is not fine, they come quickly. And when they see its beyond them, they call the doctors and they also come quickly. Yes, even at night the doctor will come. I saw that man with a bold head, he is called xxxx, they cold him at night and he came and attended to the patient* |
|  |  |  | *Like what was happening on the ward people have been good, you reach out to them and they tell you to go and buy the drugs. You go and buy it, you come back and tell them that I have brought the drugs and they treat the patient, that is the good care that I have seen* |
|  |  |  | *Those health workers don’t rest…Ah [no], they work day and night; this one gets tired, another one comes, that’s is to say there is a nurse all the time and if anything happens, you can easily go their room and call them; “My patient is like this and that, can you please help me?”. They will come and help your patient. I thank our health workers for that good heart* |
|  |  |  | *When you come and you are very sick, there are many patients here who come and they are very sick indeed. So when you come and bring a patient like this, they are very quick to work on that patient, they don’t make you wait and die here, they take you to that doctors’ room and they put you on drips when you get better, you come to the ward* |
|  |  |  | *So I had those health workers who were my friends and they kept on coming to me especially the intern doctors. They were really my friends. They are the ones who would come and tell me to buy this drug and I would go and buy it and they would come and inject it on her. They would come and ask me to change the drug, they tell you lets change this and we would change it. So I was making it easy for them and they realized that am an obedient woman and they were also easy for me.* |
|  |  |  | *Let us they really took care of my mother because I even have doctors who gave me their phone numbers and they said that if there is no change in the condition of my patient, I should be calling them. I have the phone number of the doctor who mostly cares for the patient and whenever I get a problem, I call him. They have really taken care of her* |
|  |  |  | *This one is extreme. He worked more than he could. He checked on me several time, even the nurses did a good job but this doctor was extra ordinary. He would come and greet you all the time, he asks how you are, how are you feeling? What about this time? Is there a difference? Where is the pain? Has it changed? Has it reduced? Is it in the same place? He would press my stomach and he tells you to tell him if he touches where the pain is and he would spend a lot of time on me. He wouldn’t panic,* |
|  |  |  | *Just that the health workers were caring and they treated me very well. I may find it hard to differentiate them because I don’t know their names and they are many. They have different uniforms; some are men others are women.* |
|  |  |  | *They worked…they worked hard and no one ignored me. Every morning, they would come and greet me and ask how I was. There is one health worker who made me laugh! He said that I have recovered, I can even go back and start planting beans and sweet potatoes and he was laughing too* |
|  |  |  | *These patients always call from their community to tell me that there is some improvement while other call that they are coming back for review. Most times we do not remember unless if they have stayed for a very long time and you were directly in their care then you can remember them* |
|  |  |  | *By the way even if you talk to them in a good way minus the drug, they will buy it willingly. I have seen this work.*  *As long as you take time to understand the importance of this drug, and its contribution. Sometimes health workers have no time to explain that the hospital has many patients and at the moment the drugs are over and we haven’t got a new consignment* |
| 1. **The role of the caregivers** | Attendants help to inform HWs when drips are used up  Attendant showed how to manage the patient’s pain  Explaining to the HWs how the patient is feeling, Informing the doctor about the patient’s condition  Helping the doctor to understand the patients’ illness  One patient felt that severely ill patients should be left with their guardians during the cleaning process because they can need help anytime | | *Yes, like how to close a drip when medicine is done or at times when the drip is not moving yet the patient is in pain. The health worker said, “Eeeh you don’t know how that thing works.” the truth is that I don’t know it because I did not study it. Now like yesterday the drip was not moving then I called the health worker. He explained for me to rub the hand of the patient then I told him that it has still failed. He told me how it can fail. I told him me I did not study it all I know is mechanic.* |
|  |  |  | *It is also up to you the person to explain yourself; “I have cough, I have been with it for this number of days, or this number of weeks or this number of months or years…so that the doctor can know. If you don’t explain to him, he may leave you without knowing or understanding your illness, you should explain to him so that he knows what to test before he decides to send you to the lab for testing the disease* |
|  |  |  | *And in the morning, they wake you up very early in the morning, they chase away the attendants, the cleaning team comes, and they clean the place when there are no attendants. The attendants carry all their belongings outside through the windows. Even when the patient is very hungry at that time, they wait and stay hungry, they can’t allow attendants to enter. Even during the ward round, attendants stay home. So if you need help, the attendant sometimes has to serve you from the window and there, the patient can eat* |
| 1. **The role of the HWs** | Nurses and doctors considered important people in care for sepsis patients  Attendant inspired by HW roles to educate own children  HWs help attendants to make patient beds, | | *Okay, what I saw that these doctors and nurse are important people. I will also educate my child because I see what he or she tells you, you do because you can never understand them. That is the thing I saw which inspired me to teach my children.* |
|  |  |  | *To keep the toilet clean and that an attendant is not allowed to make the patients beds, those students are supposed to make the bed. If the patient can leave the bed and stand somewhere and they make their beds. And after, the patient goes back to his or her bed.* |
| 1. **HW competence** | Some HWs did not care much about patients  Questioned the competence of some health workers to manage patients  Cannula put on a patient and left blood oozing. Some HWs don’t know how to remove cannulas  Hospital has capacity to treat patients given its capacity  Availability of Senior Doctors  Happy that the doctors would not treat the illness that they do not know  Not happy about being left only with intern doctor  Example of how an intern student badly injected a sepsis patient and the patient’s hand got swollen and burnt  Attendant wants intern students to be supervised by senior staff while they attend to patients  Qualified Medical doctors  Happy that the patients are reviewed by doctors who know what they are doing  Trust their doctors because hospital cannot hire unqualified staff  Senior doctors not available all the time  want senior doctors to be available  Happy that her patient was reviewed by a senior doctor from XXXX and explained the patient’s illness in detail  When HWs are available, attendants enjoy their presence  Attendant can think HWs are not competent is they don’t explain the patient’s illness  Patients expect to find highly qualified staff  Patients would be assured of being reviewed by qualified HWs including specialists | | *What I saw with the doctors is that they were not caring I think some of them were just learning I don’t know because there was one who put her on the cannula and it failed and the blood kept coming. So there was an old doctor she asked me to how the person who put it and I showed her that she is there, so you find that it hurts you to go to the doctor but you find that what they are doing are not right. And me I don’t know how to do them, so you find there other one went but left after she had put in the cannula and left the blood coming. So they also don’t know how to remove the cannula.* |
|  |  |  | *When you come to xxx Regional referral like this one, you know that I have been referred to a bigger hospital, I will find there all the doctors who have the capacity to treat my patient., a doctor who can be consulted to confirm that the patient is suffering from this and that. Because we know most of the doctors that are senior and experienced are in these big hospitals of government. They are the ones who are here* |
|  |  |  | *To be able to give you any form of treatment, you must be weighed, and measured the kilos, they should know that the patient understands the illness that she has so that they can give her appropriate drugs. They cannot just give you pills anyhow without examining or testing you.* |
|  |  |  | *The other thing that is there is that sometimes, they leave us with intern students. You find that sometimes, they have given us a night nurse after the doctors have gone home. The nurse eventually sleeps and leaves the students alone to come and inject drugs to the patients.* |
|  |  |  | *Like for me, the students badly injected my mother and the drugs caused her a problem. Instead of using the locking part of the cannula and bringing drip water to flash, they used the real medicine to flash and the medicine burnt my mother’s hand, it is now swollen, you can think she was burnt by fire. I also didn’t know what it was but when the doctor came, he asked me “what happened to the hand?”. I told her that I also don’t know. He said “It seems they flashed her cannula with drugs”.* |
|  |  |  | *But in all that, the good thing was that the when you are on the ward, you are sure the doctor will see your patient, you feel strong, atleast you know that the doctors know what they are doing. Except the students. I trust our doctors and nurses; they can’t be employed here if they are not qualified. So when the doctor comes, you feel relieved. And when they enter this ward, they don’t leave until they finish seeing all the patients, that’s what I thank them for.* |
|  |  |  | *The other thing I see here is that there are doctors whom you would call senior doctors. But I don’t usually see the senior doctors. I usually see intern doctors. If there were senior doctors that we are used to like Dr. XXXX like now me who is caring for a patient with a heart problem, I have been taking her to Dr. XXXX and whenever I would ask, they would tell you that it is that doctor who is supposed to be seeing such patients. So they should get for us senior doctors to be here all the time. If these children were not there {meaning students}, we wouldn’t have senior doctors in the female ward. Like in the children’s ward, we know that there is xxx. But when I asked about the senior doctor in the female ward, I didn’t see him and I don’t know him, I only know intern doctors. And if there were senior doctors, at least things would have been well. And I also learnt that the girl also didn’t know what she was doing, eventually my mother ended up getting another illness that we didn’t understand. The drug which was supposed to pass through a vein ended up not moving, the whole body had got sores. But if there was a senior staff to supervise her, that wouldn’t have happened. But in most cases, they would leave us with intern nurses but you find that experienced doctors are also not there. That’s why I said that the intern students didn’t know what they were doing.* |
|  |  |  | *It is possible that it is not only health workers but most of the things… it is those that are teaching them that should be available to see if they are doing the right thing. It means they are not teaching them well. If they have taught them, I don’t know why the students would be doing such a thing to the patient* |
|  |  |  | *They also expect to find highly qualified personnel which are not in the local clinics. Right now we have physicians, consultants so they also expect good care from those. But the doctors and intern doctors are always here except the physician is never around, he works two days a week and that’s a big challenge.* |
|  |  |  | *Not really, we have for example people at the fore front the interns, nurses and apart from a few departments which do not have specialized but specialist are also available and we are seeing these patients promptly* |
| 1. **Different perceptions about the hospitals cleaning procedures** | Happy about sending caregivers outside the ward during the cleaning period  Ward is left to dry  Irritated attendants aren’t alerted before cleaning  Feel that ward should be cleaned more than once a day | | *They have also been cleaning the hospital on time, they do that every morning at bout six thirty, they ask us to get out so that they clean the ward. We leave the ward and they clean the place where we care for our patients.*  *They clean and after it has dried, they tell us to go back. So for me, I remain in the ward given that my patients couldn’t talk with the doctor that she is feeling pain here. He will ask for the attendant who will have observed any changes. So after they clean, I come back to the ward and sit near my patient* |
|  |  |  | *What I have also seen is that the cleaners do not know their work. They clean at 9;00 and they don’t do it again.* |
|  |  |  | *Secondly at time the cleaners just pour water on the floor minus enough warning since many were sleeping on the floor.* |
| 1. **Discrimination and favoritism increased waiting times and unequitable delivery of care** | HWs prioritize treating people that they know  Some patients skip the queue while others stay longer  Long waiting lines at the pharmacy  Example of an attendant whose patient was quickly attended to because the HW knew them  Attendant thinks his patient was ignored because he was poor  Patients felt that those who paid money were given priority  They cited act of bribery by some HWs to help some patients to skip long lines | | *It needs when you reach the hospital whether they know you or not, they should work on you and not working on people they only know. You put your medical forms there but some people in the line are just called and worked on while you are seeing. That thing makes me feel bad!* |
|  |  |  | *I went with another caretaker to place in our forms but as we were there, there came two people put in their forms yet ours were the first. As we were there they stood in the window and got drugs yet for us we were seated there. Remember they had not even worked on the people we found there.* |
|  |  |  | *So when we reached here, we found that the health workers already knew us since I was coming here most of the time. Most doctors were there previously whenever; I would bring my mum. So the doctors helped me and we took her to the bed because I found the health workers on duty* |
|  |  |  | *Yes, they first stopped giving her blood. But after they first brought the water and put her on drip. After they had put her on drip then they brought the blood and gave her. There was no one coming to attend to her all were just bypassing her and going to attend to other patients and then I asked myself that do these health workers need money or what? And still they said that we go back home.* |
|  |  |  | *Then I said that these people have socialism, or it was because they saw I was poor hahaha so all the thoughts were annoying me. Then I walked away and sat far away outside on the veranda and kept quiet but I said that this is because of this poverty.* |
|  |  |  | *In x-ray they also want money, that screen they took it from EDPA then they sent you to this one of here, when you reach there, they also tell you that the scan got spoilt. But it didn’t get spoilt when the person they know comes, they scan them, and as you are still there you see them telling you that they have been screened. But you when you go there without the money, they cannot.* |
|  |  |  | *We suffered when we were looking towards reaching the doctor’s desk in the line, we were very congested but they should also stop segregation* |
|  |  |  | *And there are other people who are being attended to but when the doctor asks them to give her drugs, they don’t ask anyone to buy it, the hospital provides it. But for the rest of us, if you don’t have cash to buy drugs for your patient, then it means your patient can die anytime* |
|  |  |  | *That’s when corruption starts from there, you have to give some money to be able to beat the line. What I saw is that while we were there, they had withdrawn our request forms and taken them to the x-ray room. They covered them because they did not want to work on us without money, so people would come and jump the line. You see that someone who has just come is being called and you who came very early has been sitting fir hours. Sometimes you would see that there are patients who pass through the other side of the room, they first work on those and then they leave before they come for you.* |
|  |  |  | *What I observed is that when you come here, they will not come at once and treat you the same, you will see individual health workers going to see specific patients and leaving you there. They jump beds and go to others. That one didn’t make me happy* |
|  |  |  | *It seems that every nurse comes and has his or her own patient, I really don’t know. I wonder if it means that the nurse has been given something…{meaning money or a bribe]. But you see someone is bypassing you even in the morning, you will see that nurse coming straight to treat that very patient* |
|  |  |  | *It is a problem because the way I saw… two days ago, if we didn’t know the other man from our village who had a patient here, and if we also didn’t know that nurse who recognized my grandmother, I was going to die at home or on the way home. It also means, If I hadn’t got that severe fever, I was going to get treatment the following day on Saturday or I would have died here on the ward.* |
|  |  |  | *We finally went there and sat! We waited! time and I would see the lab people passing us and working on other people. We stayed in the lab till evening. They would work on others and leave you here. By good luck, one of the health workers was coming from the lab, I went and asked him why they were working on other people and skipping us. That was at around three in the evening* |
|  |  |  | *We finally went there and sat! We waited! time and I would see the lab people passing us and working on other people. We stayed in the lab till evening. They would work on others and leave you here. By good luck, one of the health workers was coming from the lab, I went and asked him why they were working on other people and skipping us. That was at around three in the evening* |
| 1. **HCW engagement with patients and guardians on information about their diagnosis and prognosis.** | Positive:  Attendants counselled about how the patient would be taking drugs for the rest of their lives  Test results given and illness explained  Attendant asked to ask questions about the patient’s illness  Attendants allowed to observe the patient while doctor examines the patient  Patients were happy that the clinicians took time to review all patients and gave them enough time  HWs are committed to their work  Few Patients told about their illness  HWs ask questions and give patients opportunity to explain how they feel  Laboratory staff explain the tests to be done on the patient before taking samples  HWs explain the infection that the patient has and ask them to buy drugs  Some patients don’t understand sepsis even after explanations  Want to know if drugs will work for them  Patients are taken by shock when told they have sepsis  Patients concerned about whether they will be fine  Patients ask many questions when told  Illness is explained in the local language  Re-assurance after explanation  Patients ask whether they will infect their families  Patient may not buy drugs if they don’t understand  the illness  Patients understand the results and jointly discuss care for the diagnosis  HWs keep results to themselves  Hard to explain sepsis to patients but explain the underlying condition | | *When we brought her to this xxx hospital, they gave her some help, they sent us to the scan, we went there and they tested her, we went to X-ray, they also tested her there. They found that the heart was swollen and the whole stomach was yellowish, we started from up there, they tested us and later sent us here for admission. They sent us to all those places so that we can be tested and they found that the heart had Water.* |
|  |  |  | *After the doctor had tested, he showed me, he didn’t stop me from seeing. I actually saw how the heart was swollen and in the heart, there were blood clots, he showed them to me, I saw them while he tested, they looked like red fire and blue. The doctor allowed me to ask him and I asked him why there were red and blue things. He explained that when the heart is beating it releases red, and why you see blue. Since red was going, it is now blue that is stopping the blood to move* |
|  |  |  | *He told me that your patient’s liver is sick and the kidneys are also sick. I asked him about this disease, because there are diseases caused by…sometimes you hear in radio when they are talking about hepatitis B*  *He also said that the patient’s liver is spoilt and it’s not a curable disease. So when he said that the liver has no cure, she has to be on drugs for the rest of her life. And yet we would buy drugs and she didn’t want to take them.* |
|  |  |  | *When that doctor starts to work, he works a lot. When he starts to work on patients, he can’t rest until all of them have been seen. He never rests, time for lunch come, he finishes the female ward and goes to the make ward until he finishes all of them* |
|  |  |  | *He takes his time to review you, he examines you, he asks you, he asks your attendant, he first consults the nurses he is with, and even asks if you have understood, he is the one who was asking how you feel and he adds you more doses if the drugs are there. And if they are not there, he still explains why it is not there and why you should buy it. You also try your best to see that you do what he has told you* |
|  |  |  | *Even with other patients, the doctor asks, is this malaria? What is causing the fever, if you have cough like me, they ask when the cough started, how often do you cough and whether you have anyone with TB at home. They ask so many questions. After that, they send you for blood and urine tests or x-ray* |
|  |  |  | *But sometimes, they would talk to me, telling me the following day, they came and asked how I was feeling. I also asked why I was feeling severe pain in the stomach. They said I had infections and …I don’t know what! There is something that they said and I don’t remember it* |
|  |  |  | *So they when I brought the results here, they told me they are treating this part of the stomach {touches the upper abdomen}, There is a part that is damaged inside. Did they say it was the heart? [trying to remember what the doctor told him}* |
|  |  |  | *Ah, they told me that they were going to test me, they even told me the tests they were going to conduct on me. They told me that they were going to do a pregnancy test, they were also going to test me for HIV, they tested fever, they tested pressure, yes they tested all of those and they told me. Everything they were going to test on me, they told me* |
|  |  |  | *Ah, they told me that they were going to test me, they even told me the tests they were going to conduct on me. They told me that they were going to do a pregnancy test, they were also going to test me for HIV, they tested fever, they tested pressure, yes they tested all of those and they told me. Everything they were going to test on me, they told me* |
|  |  |  | *And at the end of it all, they ended up explaining to me that what is bringing water in my stomach is an infection. The infection is the one that is causing fluids to fill up my stomach. That’s what is happening right now. And then the time comes, the bacteria get to the liver and then produce water and that’s why I’m vomiting blood because the bacteria is stuck on the liver causing the liver to produce fluids eh* |
|  |  |  | *When I brought the results, the health worker said that I should go and buy tablets because they found that I had fluids inside, so when I reached here, the health worker looked at the results…the one who has sent me to the scan and told me to go and buy drugs because the scan showed them that I had bacteria in my stomach which were producing fluids.* |
|  |  |  | *But when you explain to them something within their body, how the disease started how it progressed, the organs it has affected and those about to affect. The patient becomes concerned you find it becomes easy for them to but certain drugs* |
|  |  |  | *Yes, because we talk to them in their local language. We just tell them that they have an infection, what is causing the pan, general body weakness, loss of appetite.* |
|  |  |  | *usually tell them that because most times we find they have bacterial infections so I explain to them because one of the tests I do is CBC and it explains to the patient the cells whether they are elevated or less. So, I explain to the patient very well what the various cells, when they are high, what it means if they are low.* |
| 1. **Some HWs do not explain the cause of the illness even after getting the lab results** | Attendants given test results without any explanations  Wanted the HWs to tell attendants what the patients’ illness is  Not being able to understand the meaning of test results for their patients  HWs not explaining the illness in detail  HWs not informing patients ow what they suffer from or even their test results  Most of the respondents expressed that doctor do not inform them findings of their assessments.  Patients do not understand their illness even with explanations by HWs  Not getting enough health education | | *I want when I bring a patient they test her and they give me the test results. They tell me that my wife is suffering from this and that. But here what you see is just the cannula, and a letter asking you to go and buy drugs* |
|  |  |  | *They again sent us to the scan, they tested her and found that there was water, they also took blood saying that they were going to test it so that they know what is going on. Since then, they never told us the results* |
|  |  |  | *Eeh and they should tell me the results of the tests from the laboratory so that I also understand what the doctor has found in my body.* |
|  |  |  | *The health workers did not explain to me what the patient was suffering from. We are the ones who explained to them how we have been staying with the patient and his usual condition. But now he has changed whether the condition is good or bad. So we would tell them and they would be writing*  *I didn’t really understand what was going on, they said the patient had a liver and kidney disease because when I asked, because they told me that it is caused by hepatitis B and yet they said she didn’t have hepatitis B. So, I left everything at that, that she has a liver and kidney problem. I’m still waiting for them to tell me, what happened when they took blood for testing. They have never given me a report* |
|  |  |  | *But when my wife asked that second nurse, she also told her to go back and read the papers if she wanted to understand. So, I wondered up to now why they can’t explain what is written in our forms* |
|  |  |  | *They didn’t tell me anything. We came back to the ward, I gave the paper to the health workers there, everybody would just look at and go away without saying anything. So what we did was to put those results below my feet but on top of the bed and I slept. We kept thinking that they will come and pick them…they didn’t touch them until the other health worker who had worked on me came on duty* |
|  |  |  | *Ah I didn’t understand anything. I was wondering why I have water in my stomach and why it was causing me blood to vomit.* |
|  |  |  | *No, they didn’t tell me anything, even my father, the moment they paid money, they stopped talking to him a lot* |
|  |  |  | *I would just see them treating me and I thought that they were trying to stop the vomiting, I did not really understand, I would just see them coming and putting me on drips and injections. And there, they don’t explain, you just see drips coming* |
|  |  |  | *They tested many things including pressure, but I didn’t have pressure. However, they said that I was at risk of getting pressure at any time. Now I don’t know why I’m at risk for getting pressure! I didn’t ask but he told me to reduce salt and sugar so that I reduce the risk of getting real pressure. That if I reduce those, I will reduce the risk* |
|  |  |  | *What is missing is that when you come here, they didn’t tell me what is causing the pressure because they said I’m at risk. Those ones they are not teaching* |
|  |  |  | *When they tell me not to worry, it’s not enough. Every human being has problems; I can’t stop worrying. At least if they can tell me what to eat and what not to eat, when to take the pills for pressure and the kind of work that I shouldn’t be doing…that would help me to reduce…at least they tell you may be to dig for one hour instead on a whole day. The doctor here had said that he will teach me, but he didn’t. Maybe they will teach me when they give me the discharge form* |
| 1. **Caregivers do not cooperate with HWs, refusing to listen and do what is expected of them** | Attendants not willing to practice what health workers are instructing them to so  Attendants abandoning their patients  HWs playing the role of caregivers  attendants shouting on the ward  Attendants refuse to go out when requested  Sometimes patients are rude and with high expectations that HWs cannot meet  Attendants insisting on using specific drugs even when HWs advise them to change  Presence of multiple caregivers on ward, attendants sitting on patients’ beds  Attendants misunderstand HWs when sent out of the ward | | *There are areas especially now the caretakers themselves whereby some of them however much you explain to them, they do not have the spirit of doing something the way you explain to them to do. Some of them the way you want. Like the hygiene, some of them do not have that time however much you explain to them* |
|  |  |  | *There are other who end up abandoning the patients and they do their own things. For example, there was a woman who was abandoned here, she was peeing everywhere. The attendant abandoned her yet she was the real daughter and so she left her here alone. In such a situation when we are also few especially during evening hours, you find out you are torn apart and the workload is too much. At times you are here on emergency yet the inpatient is also in a bad condition and they need attention. In the end we do not give proper care to those ones who want it.* |
|  |  |  | *Some of us at times we feel bossy so they normally over shout in the ward. At times when we request them to go out, they start shouting. These patients also comment on us from all the wards. They do not say anything but they just describe some of us the way we act because we have different behaviors* |
|  |  |  | *We normally meet patients who are not cooperative, very rude with high expectation, but we cool them down and explain to them what is on the ground* |
|  |  |  | *I can say is at times you can get patients who are un-comparative. Or they are too bright to understand the care. You want to do this but someone wants to direct here. Someone knows when I get sick, I use this specific kind of medicine and it is what he wants but he has been over using it and it has become sick and sick meaning that it needs to be alternated. That person is only attached only to that medicine and getting addicted to its use. It is no longer providing medical results that we desire but the patients are insisting that he wants that medicine.* |
|  |  |  | *There experience is not different from what we have been saying like we can get so many caregivers coming to manage one patient. However much you explain to the caregiver to stop visiting the patient, they will not understand. You will find that instead of leaving the person alone who is septic because they need some fresh air, they will sit on the patient’s bed. When you try to send them away, they will say the nurse is rude. Sometimes they will fail to understand us.* |
|  |  |  | *The hospital by then it was not involved but the nurse on duty was the one to act so we did the necessary part that the patient needed. That patient was on the bed so we had to transfer her to another bed so as she feels comfortable until the attendant came in the next morning. There are times when they over shout in the ward because they do not cooperate with us.* |
| 1. **Patients running away without completing treatment/Discharge against medical advice** | Patient run away   - When sent to the lab and find that the lines are very long - when not getting treatment - expenses on food - When not recovering - Equipment are down   Patient relapse and are re-admitted  Others ask for discharge when they can’t meet the above  Not completing their dozes | | *If they can’t get the treatment, they want. If you send them to the lab and the lines are very long, then they don’t come back* |
|  |  |  | *We call it discharge against medical advice. The patient sees that he or she is not getting treatment, they are here, they are buying food, they have no care, the machines are sometimes down, they don’t know what they are suffering from. They decide to discharge themselves. Whether they die home or recover, nobody knows. Others go home, and then relapse, like they get sick again and come back as new admission. If they are lucky, they find the drugs. if they are not lucky they get IV fluids and ask for discharge…it’s a big challenge.* |
|  |  |  | *This is common especially when it comes to treatment. Most times we have the medication especially if the medical stores have supplied the medicine. When they are not there, they don’t get. That is why we have so many patients running away from the hospital* |
|  |  |  | *Sometimes it is clinically but those investigations are ordered for and patients are told to go to outside clinic to those who can afford. For some who cannot afford, do not do the tests and sometimes causes people to run away from the health facility. This is because they will start to tell you that they send them to do this and that. They will tell you that this is a government hospital, the services are supposed to be free and indeed they are right. In the morning by the time you come, the patient is not on the bed. They have run away because they see no reason to stay if they cannot be investigated or given drugs* |
|  |  |  | *We explain the symptoms in relation to the infection. Majorities accept and be admitted, treated up to end. If they were not accepting, they would be running away. There are few who run away without completing their dozes.* |
| 1. **Patient/caregiver relationships/communication** | Attendants shared items with neighbors such as food and drinks  Helping other attendants and patients helped attendant not to be alone  Relating well, consulting and advising one another on how things should be done  Attendants trusted one other  Leant of the need for a good language between attendants and patients, importance of making friends to be helped  Importance of having friend, cooperation  Talked about the importance of living in harmony, relating well,  Attendants easily related to attendants whose patients had similar conditions. Offering help to turn and change the patients  Got friends  Helping the neighbor with what they don’t have  Attendants related well with other attendants or patients  Discussions about treatments and tests  Sharing food and other items | | *I expect there to be good relationship…because there are some patients whom you find and have nothing and you offer them something. When I bring something to eat, I share it with other people. The patient near me did not have anything to eat so I told my wife to give her soda to drink. There is no any challenge I have faced with my fellow patients or care givers. I even gave her my basin to use for washing.* |
|  |  |  | *Even though the people who were our neighbors in the ward were not speaking our language, they were speaking Swahili but we were helping each other very well even when I would be outside they could call me and tell me that the patient needs something and this I was not expecting to find it there given that I was alone in the hospital. So in case I was moving out I wouldn’t get worried that she is a lone but I was sure that they will take care of her.* |
|  |  |  | *Okay, what made me happy about the other patients, we have been relating well, we were consulting each other very well, and somehow you even advise the person how this and that should be done as a person who is taking care of his or her colleagues. The person also advises you on how you should do things. He or she shows you that go here, you will get a doctor or a nurse.* |
|  |  |  | *By being good to each other, we were trusting each other. You get to know one another when you have come to the hospital, you should have good language, you befriend your neighbor, so that you can be helping each other. If you are neighbor is not around, you should help him or her and if you are also not around, the neighbor will be helping you* |
|  |  |  | *They are my friends in many ways; my patient cannot sit and is there is a bad way in which she is positioned, my friends would come and they want to help me so that we help her to make her sit at least in one position* |
|  |  |  | *I know that when you are in the hospital and another person comes and he or she is sick, it would be good for the attendant to cooperate hmm and there, the most important thing is let us say cooperating* |
|  |  |  | *The other thing I saw is that sometimes, you can come to the hospital when you are unable or poor let’s say; the patient on the other bed would help you and give you what you don’t have and of course we were communicating well as if we were siblings* |
|  |  |  | *Otherwise, in the ward, we didn’t have any problem with anyone. Even the bible says that birds of the same feather fly together. It is the same here, there is a way we are relating well with our colleagues. You find that your situation is similar to that of another person. Like the lady who was calling me; we are like friends; you find that we are sitting there. If I want someone to help me change the patient, I call her and she helps me together with the nurse and we carry together. And if they also want me to help them, I also go and help them. There is a way in which we help one another. It depends on how you work together and how you have appreciated one another. We have got friends.* |
|  |  |  | *What I saw is that we the patients and attendants, we were staying very well. We would greet each other and even talked, ask how are you, you even ask what your neighbor is suffering from. We have been relating very well* |
|  |  |  | *We were talking about things like the illnesses that we had, we would converse about how we started getting sick, how we arrived here like that, how we are now…what the health workers have told us, how we paid money for drugs…* |
|  |  |  | *I and the other people on the ward, we were looking at people and saying that this hospital is good if you have something, it needs you to have some money. If you don’t have money at all and you are very poor, you can or your patient will die.* |
|  |  |  | *Even in helping, there are people who have their appetite and the way they are eating. But you find that a person like me, I do not have any appetite to eat food and I just want to drink soda and I don’t want anything else. So I would share some of my food with my neighbors* |
|  |  |  | *I was with people, the attendants and we were there chatting and talking about health workers when you don’t want them to know what you are talking about. You look at someone, you say that he or she came well and showed willingness to care for you, then eventually they ask you to pay money.* |
| 1. **Family support** | Being helped by siblings to manage dropping the samples outside hospital  Friends, attendants, and family members played an important role in the recovery of the patient | | *Yes, all these processes were difficult. What helped me is because I had my siblings, I would leave the patient here with them and take the blood samples. I would the oldest child of my mum and I was the one moving in and out to ensure that all the tests are completed on time* |
|  |  |  | *I feel very happy. I think they were patient and waited for me. I also had good manners; I wasn’t this patient who disturbs attendants.* |
|  |  |  | *Because there are patients who came with their children as attendants. That same child takes care of the patient until they recover. Another one may come with the husband and that’s the only attendant. I had five family members attending to me. And all the five people were sleeping on the ward* |
|  |  |  | *The other thing that I was happy about; ever since I got sick… my husband doesn’t even know anything about home. He has been here with my daughter who also doesn’t even know what is at her home…they have been here supporting me* |
| 1. **TECHNICAL PROCESSES IN CARE PROVISION** |  | |  |
| 1. **Delay in reporting and identifying /recognition of sepsis** | HWs delaying identifying sepsis early  Mainly diagnosing other diseases  Patients sent home but no improvement  Sepsis recognized after patient’s return  Spiritual beliefs such as prayers delayed recognition of sepsis and treatment | | *When they took her for check-up, they tested for typhoid. Results came, they treated us and we went back home. They prescribed four drugs, gave us two drugs and other drugs I bought them. We then went back home. When we went back home, after two days she got sick again. Like in two days she was swallowing the drugs but there was no any improvement. I decided to take her to a private health center but I saw that it will need much money. So, I decided to go back to XXX* |
|  |  |  | *They told us to take him quickly and prepare the bed and we come and attend to him. And in five minutes he had already worked on him and the only medicine that was not there was for malaria, he searched for it in vain then he asked me to get two thousand and I go and buy it but I told him I don’t have the money but because I had friends, I asked my friend and she is the one who gave me that drug for free I didn’t buy it.* |
|  |  |  | *When we went back home, we spent there one month, and we came back and they admitted us, we stayed here last year and we left and now this year. So on 31st January this year, she again got sick, there, I would only hear her saying that the chest is paining her so much and her heart was beating very fast, then we came back here*  *So since January, I have come to this hospital four times. They discharge us, we reach home and spend there three to four days and we come back. This time we came because she got a stroke* |
|  |  |  | *At the beginning…she first got hernia. That is before this admission. So with the hernia, they operated her and after operating hernia, she came back home. After going back home, she did what? The stomach started swelling and I said; now, what is wrong with this woman? Why is the stomach swelling? Let me bring her to the hospital. they treated her and gave her injections they removed fluids and she improved.* |
|  |  |  | *There is a time when she was going to pray, there is a father (priest) where she has been going for prayers for the three days after I had brought her to xxx hospital to remove the fluids. Then they went for prayers and spent those days there. The priest prayed for her but upon coming back, she came when the stomach was what…it came back when it was swollen and since it was swollen, I had to come back this side* |
|  |  |  | *Yes, I was in xxx but they took me to a private hospital they told them that it seems the old woman is suffering from malaria and typhoid. They were all seeming to be not sure, they were guessing.* |
| 1. **Patients are irritated with long waiting times in the laboratory and at the pharmacy** | Long waiting times in the lab  Patient paid money for lab tests and didn’t get the results until discharge  Patient collapse in the waiting line in the lab  Example of a male patient who collapsed in the line and brought back on ward. | | *But we went there and spent almost three hours. I asked my husband why we can’t stop the tests and we just ask to be discharged. He told me to be strong at least we buy drugs. I slept in the chair until they called us then we went and they tested us. And after taking your urine and blood samples, the rest are easy and results come back very fast and they tell you what is happening* |
|  |  |  | *Because they would send you there to the lab for blood tests but the time you would spend there, you would see a very long line and then you come back and start again…you find that they would just leave. And if someone sees that the line is very long and they leave, it means they will not come back tomorrow.* |
|  |  |  | *There is a time a male patient collapsed there, and they brought him back to the ward. It seems he was not feeling well, he stayed there, and they brought him back to the ward. I didn’t see him myself but I heard people saying that he had gone to the lab to be tested and then they brought him back* |
|  |  |  | *All the money I had, had been used to buy drugs and to pay for the scan. They kept telling me we are testing this; we are testing that and they were not bringing the results and yet I had paid for all the tests they told me they were going to do. So I begged him and I offered fifteen thousand so that he follows up my things and I get the results. He said no, he started confusing me, that loss, that they don’t have time and things like that. I insisted that I will give him at least that money and he gets my results.* |
|  |  |  | *When I reached here, but to go to the lab and all the waiting time took me about two hours, almost three hours because when I reached the hospital, the service was not that quick, wherever I went I delayed a bit* |
|  |  |  | *There is a time a male patient collapsed there, and they brought him back to the ward. It seems he was not feeling well, he stayed there, and they brought him back to the ward. I didn’t see him myself, but I heard people saying that he had gone to the lab to be tested and then they brought him back* |
|  |  |  | *like delaying them in the line. For example, you see a patient you sent to the lab, they stay there the whole day and they come back and tell you “musawo” [health workers], I have not been worked on. They lines are always long; they don’t prioritize such very sick patients on ward. So they complain about that* |
|  |  |  | *You know people should not wait before for days to get just a unit of blood, we need give it on time, time and treat them and also they feel contented not the five minutes that we give them* |
|  |  |  | *So you find some patients wanted to be discharged that day, and at discharge, they have to get drugs from pharmacy, they lines are long. So they can’t go home. When they come back from pharmacy, they find the bed has been given to another patient. That annoys them.* |
| 1. **Patients irritated by repeat taking of samples** | Lab reports being misplaced or lost  Feeling that attendants are not responsible for the custody of their patients’ files  Feeling that sepsis patients already have little blood  Irritated by the cost of repeated tests  Attendants have to look for money to meet costs for additional repeat tests  Irritated by HWs wanting tests to be done in specific labs | | *I got angry and asked him; “why are you saying that I should go and test more blood?”. He said that they lost the previous lab report. I wondered why they lost such papers and yet they had a file for each patient. I thought that every time we bring a copy, you put it in the file. I told them; “If you keep telling me to go out and repeat tests, it shows that I’m equally responsible for the custody of my patients files, which is wrong. Health workers are entirely responsible for keeping my patient’s documents and forms”.* |
|  |  |  | *Yes, they wanted to take off another blood to see whether the blood is now enough after they had given her the first pack, or they add her another second pack but we said we don’t have the money, the blood that she has received will be enough. But I didn’t like that given that the patient already had little blood* |
|  |  |  | *I said that because I had paid fifty thousand for the previous test, now they wanted me to pay another fifty thousand to repeat the test and I refused. But in the night I realized that I was too angry and yet health workers were also helping me. So I went and told one of the doctors that I will look for money the following days and if I get it, I will go and repeat the tests. And every time you talk of lab tests, they mean you going to specific labs that they know. I think they earn some money when you go there.* |
| 1. **No wheelchair to transport patients from OPD to ward. No direction to the wards** | Sepsis patients carried by attendants  Fear to lose the patient as they take them to the ward. HW should direct or accompany the attendant and patient  Attendant asked to take patient to the ward  Good care means availability of wheelchairs to push the patient. Need to have enough wheelchairs  HW tells patient to take patients to the waiting areas  Patients and attendants not directed-get lost  Attendants get lost while they carry patients to the ward for admissions  HWs mention that patients are transferred to ward with the use of wheel chairs and trolleys  Only one-wheel chair available  HW thinks that the patients know the ward very well, no need for direction  Patients accompanied to the ward  Patients admitted on ward yet they should be in HDU  Poor coordination regarding where the patient should be admitted especially when with diabetic wounds  Patients accompanied by relatives to the ward | | *If you have a very sick patient, you have no wheel chair, you have to carry her here, eeh, and if you don’t know where you are going, she can die in your hands, so the health workers at the reception should direct you or come with you to the ward.* |
|  |  |  | *So the health workers tell you to take the patient to the ward, take her to the women’s side because her stomach is swollen. She came when she couldn’t sit, we would just carry her even in the vehicle. So three days ago that’s how they admitted her.* |
|  |  |  | *Eeh good quality care is to say that if the drugs are there, and the other service like if your patients is very sick, they need wheel chairs. But there are no wheelchairs for pushing your patient. All the wheelchairs are broken; they are not there in the hospital. You find that there is one wheelchair and all of you are in need of that one wheelchair and yet you are about six patients. And when you take your patients, you will find it difficult, you carry the patient on the mat, another one also brings his patient on the mat. If the wheelchairs could be enough.* |
|  |  |  | *The patient could not come to OPD from the vehicle, we should take her directly to the table so that the nurse takes her to the consultation room to see a doctor. So we reached the table and the nurse told us that since the patient cannot sit or stand, we should go straight to the waiting area there (ward)* |
|  |  |  | *Another thing is that when we come here, there are some places we don’t know yet it is where someone has sent you. From the reception, many patients are sent on ward and they get lost, you can end up going to the accident ward, you reach there and they chase you a way. There is no body to direct you to which ward you should go. What helped me is that I was here many time and I knew this ward.* |
|  |  |  | *At the OPD they asked me the way I felt, how the pain was and how the sickness started and what happened. After that, they gave me a number and I sloped this side. My husband carried me here.* |
|  |  |  | *We have only one and sometimes we rush to OPD to get one in case we have many patients. I end up using a lot of energy when rushing to get a wheel chair.* |
|  |  |  | *The care is all given but at times things get out of hand. There are some patients who are diabetic. You see those patients the first thing they see is medical ward and what we do is to identify and we send to HDU. There are some patients who are sent there direct but for diabetes the send to us so when we see we cannot manage; we send them to HDU* |
|  |  |  | *The challenge comes when you find a patient has diabetic wound and that wound needs the attention of a doctor from the other side. Once a patient is admitted, there is that gap. For them they will send the patients here yet the patient cannot receive proper care from here because there are no sterilized materials here. In this situation I realized that there is a gap* |
|  |  |  | *They are usually accompanied by a staff from the OPD who brings the patient directly to the ward and when they reach on the ward here, we have our admission room.* |
| 1. **Long queues, high patient load and shortage of HCWs, leads to high workload for HCWs and delayed care for patients** | Long waiting times especially on Mondays  Fear that the patients can die in a queue  Delay to be registered, delay waiting to see a clinician, wait for your name to be called delay to get an OPD number  Delay to be admitted, first asked to do a scan  Fear that patients collapse while waiting in the line at OPD. Attendant wants sepsis patients to be worked on urgently  Spending hours in the laboratory waiting to get test results  Delays at OPD waiting to get numbers  Numbers given but late  Many patients in the queue  Long queues and waiting times at triage and OPD because nurses at triage are few  Feeling that the patients can collapse in lines  Recommended that the nurses at triage be increased | | *Because they can keep you standing in line and someone can easily collapse. And on Mondays, if you are not very sick don’t go there on Mondays because you can easily die. So, it’s better to go there on Tuesday. Because you know that on Saturday they will not be there too.* |
|  |  |  | *They tell you to first go to that table for registration and after that, they tell you to sit and leave your book on the table in OPD, you have to wait and they call your name on the book. Then they send you to the doctor and yet the number of patients waiting is very big in OPD. It requires more health workers in that area.* |
|  |  |  | *By the time we reached up there in the hospital to register her, I saw the patient was continuing to be worse. We took her with a boda and I was very scared but the nurse saw us and hurried to help us* |
|  |  |  | *When we came to XXX, we stayed in the OPD for some time. After waiting for some time, the people in OPD they sent us to the scan* |
|  |  |  | *The first thing is to start with is that of OPD, there are so many people there and you have to go there very early. And if you have a very sick patient in OPD, they work on them quickly. But if the patient is able to talk and walk, it is the patient who knows what is paining him. You as a person cannot know the pain that patient is going through, such patients spend lots of time in OPD. In that instance, the patient spends more time. There are nurses who receive patients on the table. They tell you to first go to that table for registration and after that, they tell you to sit and leave your book on the table in OPD, you have to wait and they call your name on the book. Then they send you to the doctor and yet the number of patients waiting is very big in OPD.* |
|  |  |  | *There are patients who collapse in OPD and you find that a person who has pressure and sometimes has diabetes and is in a very bad condition and may be the pressure is high. So they should work on such patients very urgently. But such patients can die in OPD before they see a doctor.* |
|  |  |  | *We spent many hours there…[Silence]. I mean that we spent about an hour up there because we went at three and came back at four in the evening. We waited so much for the form that was requesting for blood, so the doctors stayed there chatting and asking themselves. We had to wait!* |
|  |  |  | *We came at twenty minutes past eight but the health workers had already started working. But may be to say that sometimes we delayed there, the line was very long, so we got the number late because people were many. Now, after getting that number…we got that number at around eleven but by around twelve, we were still waiting for the health worker to work on her.* |
|  |  |  | *Eeh that time, she had not yet been admitted, we stayed the whole day. I remember it was a Saturday they told me to come back on Monday because I had not completed the process in OPD. So I went back on Monday and completed everything in OPD. We completed the pressure things, they sent us to room 10 upstairs where the pressure patients are cared for. There are doctors inside but they are like doctor XXXX is the only one seeing the patients there. I heard people saying that if it is Dr, XXXX then we will receive good care. So you would see that the patients were very many and the doctors are only two. If dr. XXXX is not there, then one doctor will be seeing all these patients. Yet you see that the number of patients is very big. They should increase the number of health workers to reduce the waiting time, in order to save the patient.* |
|  |  |  | *There is where they give numbers from the reception. You go to get a number when you are very weak, you wait and you get tired. Remember sometimes health workers come late, it seems they first go for meetings up there. They finish working on you everywhere but you find those giving numbers are not there* |
|  |  |  | *At times patients can even collapse while waiting for the numbers. It is disturbing and therefore they should be giving those numbers in the wards or they give it to you there and then. Remember, if you don’t have it, there is nothing you can do, you can’t be admitted without it. I was therefore requesting that they quicken that process for us* |
|  |  |  | *They should put enough nurses at the triage so that they can give patients numbers on time, they should increase health workers. Like I told you, they should be increased. On the triage, they should put enough nurses to give numbers to patients* |
| 1. **Long waiting times at triage leading to delayed care for patients** | OPD not active 24 hours  OPD emergency not very active. Patients seen by clinicians on ward  Patients who arrive after 5pm go straight on ward  Vital signs are taken on arrival and patients see a clinician in OPD  Referred patients do not wait at OPD, they go straight on ward  OPD delays to decide whether patients belong to accident or emergency causing a delay for the medical emergency patients  Patients not understanding why some patients are worked on before the others | | *OPD emergency room is there but I don’t see it operating the way it is meant to. In most cases when they realize they just come straight to this side.* |
|  |  |  | *All patients start with OPD except patients that are in road accident and maybe patients who come in at night. The patients who come at night, they find OPD closed because they do not work at night. OPD opens up to 8 am. All patients that come after that time go direct to wards.* |
|  |  |  | *Well we receive patients when they are sick through OPD and some come here on their own mostly at night that is when they reach here directly so it is upon us the health team to assess the patient and find out depending on the presenting signs and symptoms or the complaint from the patient.* |
|  |  |  | *Sometimes it is difficult and you find them seated there waiting or sleeping there for over 30 minutes without being attended to. At the time of discharge, we do not take a lot of time. The most time when they need attention is when we are in the investigation.* |
|  |  |  | *Any patient that comes thinks he is the priority of care yet we have a system of prioritizing emergencies. You find a patient looking at you with at you when moving a patient on a wheelchair or who is on a crouch and badly because she has sat for one hour. Someone wants to squeeze in before the emergency. In most times you may not have time to sit done and explain that you have an emergency? At the end of the day someone may have a bad heart with you thinking that person has paid yet this a public hospital.* |
| 1. **Unavailability of equipment for triaging and examinations results in no or limited diagnosis** | Most tests are done outside the hospital  The tests are paid for and expensive  x-ray machines, ultrasound, CBC machines are broken down  Critically ill patients travel on Boda bodas o seek services outside the hospital  Laboratory tests are paid for | | *On that day, when I came, they didn’t do any tests. Yesterday, they sent me for the scan. The scan of here has a problem. It is down and not working. So, I went for the scan which I paid for, I got my results, they put me in the scan, and they found that I have an internal damage.* |
|  |  |  | *I already told you about the scan, how hard it is to get transport to the scan when you are very sick in my condition. I would see some patients would come and they don’t have anyone to care for them. How do those ones go to the scan outside? At least if you are admitted here, the scan should be working. Otherwise, a patient can die on a boda boda when they are going for the scan hmm* |
|  |  |  | *And the testing is not for free, I paid money twice. They tested for typhoid and ulcers, I paid Money for those ones. Then the scan, I paid money for the boda-boda to get me to the scan and back, I paid twenty-five thousand shillings for the scan, that was an extra pain. But xx is a government hospital before we came, they told us to go with some money because everything here is for buying.* |
| 1. **There is perceived unwillingness among HCWs to carry out the recommended diagnosis processes** | Somme nurses on night duty refuse to wake up when called upon by patients  Acts of mistreatment by HWs | | *But there are these ones working on night shifts, patients wake them up and they refuse to wake up. That should be improved, health workers on night duty should do their work* |
|  |  |  | *They are ever in the ward but there are those who don’t work also, I also know them, they even abuse in the afternoon and waking them at night that the drip is finished, she almost kills you. Even today she is on duty, most importantly, she did not mistreat me, but I saw her shouting and refusing to help people who are in drips* |
| 1. **Understanding of Sepsis** | Patient Understanding of sepsis  They understand the germs and how they lead to sepsis  They don’t seem to understand sepsis after explanations  Patients don’t understand if they are illiterate  HW not able to explain in the local language  Patients understand what has been explained and discuss the plan for their care  HWs: Not sure if they can explain the technicalities of sepsis  HW think explaining would stress the HW and the patient  Health workers understand sepsis when they see a wound  Sepsis is used as an escape goat  Most doctors know sepsis as a post operation or surgical would | | *I don’t know how they know because in most cases we do not tell them that they have sepsis. Even if we tell them sepsis, they will not understand. They will understand germs or fever, or malaria. We tell them they have “a lot of germs” in the body and these germs can lead to complications if not treated well* |
|  |  |  | *In some cases, they have the concern and sometime they seem not to have understood. There are conditions which are hard to understand like if you get a CBC, they tell you that you have an infection. The first question the patient will ask is which infection?* |
|  |  |  | *That if someone has some degree of understanding but when they are low or a bit illiterate, they will not understand by what you meant by infection and you will not be in position to explain in the local language what the infection means because there are so many infections* |
|  |  |  | *Well results we explain to patients’ even those which are hard for example malaria when we get the results, we tell the patient that we have tested blood and you have malaria. There the patient will understand and then you discuss the care that this is the possible treatment we want to give you then you go on to check from the in-patient pharmacy if it is there.* |
|  |  |  | *Sometimes you are not sure whether you will explain to the patient and they explain the technicalities of sepsis. What happens is that you don’t go in detail. Once you have confirmed sepsis, you just treat the patient to avoid stressing yourself and stressing the patient.* |
|  |  |  | *Basically to ensure that the terminology is not so much for example for bacteria, you can say germs. They can understand that here are germs in the blood and the body is trying to respond or to fight and sometimes the fight response maybe so much for the body that other organs are also affected. Some may understand depending on their level if education* |
|  |  |  | *What I understand is that sepsis can be medical or surgical whereby surgical sepsis are patients who come with wounds, infected with microorganisms thus creating pus. They say when a wound is septic, it creates pus. When it comes to medical perceptive, sepsis does not have wound with pus. Someone can come in septic shock because where there is sepsis, the patients have high fevers* |
|  |  |  | *Yes, you know sepsis is broad. There is visual sepsis and tested sepsis. There is what we term septicemia when it is within the blood system. Then there is this one you see like this part is infected like diabetic foot there, sepsis is going on. There are also those who come some infections after operation and you have to give them medication properly. But nevertheless all the diseases we have here are causing sepsis* |
|  |  |  | *Okay, like when the patient over stays more than days, because we do investigation like patients with meningitis if they are investigated and approved with positive results, they are started on drugs. The rest we treat clinically based on signs and symptoms. We conclude that this patient has sepsis even if the tests are done hahah [laughs] we use our own judgement.* |
|  |  |  | *In general, when you talk or go into details in sepsis, a health worker will think of a wound that is oozing pass. Now when you come to medical ward, it is hard to find that. However, in the in depth of sepsis, it is a successful infection. When the body is infected successfully, that is sepsis. Any condition can bring successful infection including malaria. The lay man understanding of sepsis is as if you are talking about a wound* |
|  |  |  | *In surgical ward, they get to know if there is a wound and it is not healing, producing a lot of pass continuously. That is when they can know but otherwise we don’t tell them.* |
|  |  |  | *it depends; there are clinicians when they do not understand what is going on in a patient they say it is sepsis. Someone comes with back pain and maybe a clinician is not well trained like some people try to use sepsis as an escape goat when they cannot get the answer they say infection or sepsis. And others are so specific, actually those in surgical discipline for them they are sepsis specific related to the site of operation. Now on medical ward, sepsis is broad. Ideally one has to confirm is it meningitis, is it pneumonia so depending on the clinician available on the ward. Sepsis is perceived differently and many people are given antibiotics in the out-patient they go home but the actual thing is not sepsis they just use it as an escape goat.* |
|  |  |  | *We may say sepsis to be localized or it may just be systemic just you know where you cannot force so the common blood test is CBC that can tell you that there is an infection in the blood but other localized forms of sepsis like meningitis that one you do a lap up rapture to confirm it. If it is pneumonia, you have to do an x-ray to confirm. So, if someone has focused urine tract infection, you do urine test what they call urinalysis* |
|  |  |  | *According to the doctors I have interacted with almost 90% know sepsis as post operation and surgical sepsis. Medical not so much. Because of that challenge, there are gaps in management. Anything from CBC is severe infection…that’s the starting point. Sepsis, sepsis but in reality, I also find it hard to explain it. So number one is knowledge gap on sepsis can and addressed.* |
| 1. **Health workers lack training on sepsis** | Training of Clinical officers different from doctors, C/Os cannot diagnose sepsis  Nurses lack training on sepsis  CMEs do not cover sepsis identification or management  Training on sepsis would improve clerkship and patient management  Mentoring of HWs  Need to train HWs on how to deal with sepsis patients  Sepsis perceived in a different way  On job training  Refresher training  More CMEs  Training  Sepsis is common in gynecology  HWs not knowledgeable about sepsis  Need recurring CMEs on sepsis  More training on triage to reduce delays in OPD  Training of HWs in patient monitoring, customer care, communication skills and empathy | | *What I mean to mention here is that the training for clinicians is not the same as for doctors. It is totally different. There is that level of education that is lacking so they cannot do diagnosis very well like how a doctor can do it.* |
|  |  |  | *Another thing, we the nurses lack training on sepsis, we haven’t received any training and even CMEs we also talk about sepsis a bit. Mainly TB, HIV, guidelines, Hypertension, DM like that. I have never attended one myself and even my nurses, at least for the last 2 years. Those are the main areas. But how to identify or manage sepsis, we treat as we know* |
|  |  |  | *The CMEs would ideally improve on the management including the clerkships themselves. This is because these CMEs training on sepsis, define what sepsis is about, how patients presents, how to manage. It covers all that, so that when they get these patients, they are able to know that this one has this. During their learning sessions, they can be mentored especially on specific areas where we see there is a little bit of weakness. Our concern is the knowledge gap and here at the hospital CMEs and mentorships are okay. If there are trainings, seminars outside this facility, they are also welcome.* |
|  |  |  | *Since you people have come in, you can help us train people who can help us in such cases. We need to be trained. The training will work best for us to know what to do and to deal with such patients* |
|  |  |  | *But in reality, we need to train our juniors staff; doctors and nurses on sepsis. They perceive sepsis in different ways.* |
|  |  |  | *We need on job training and also other trainings. You see we are aging and since our qualification, years have passed but to find refreshing trainings is hard. We have more of meetings and even trainings. CMEs are done in buildings and not on patients so there is when you need a practical thing. Pick on a condition, have time to it then area review the patient with such problem, there you will improve care. This is because someone has seen and can implement than hearing and you cannot figure out how the thing really presents* |
|  |  |  | *I think also training us on sepsis, like I told you we think sepsis is a wound with pus and that is what most health workers will tell you. Most sepsis patients are in gynae. So maybe we lack knowledge on sepsis and its management. That will help us health workers. And to also give is charts which we can follow to manage fluids for sepsis patients* |
|  |  |  | *I saw a case where one was given medicine to go home with yet the HWs really observe that it could be[sepsis]. So it means some people are not knowledgeable enough. They need more CMEs and trainings. Yes, I think that is what I can say about it.* |
|  |  |  | *The health workers understand sepsis as a wound and if you see a diagnosis as sepsis, just know that they were guessing. I think with us the project staff, we received training on sepsis and how to identify it. The training we have got before the study helped us to get a better understanding of sepsis. But the health workers are actually not trained, so we understand it better and sometimes try to explain to them. Sometimes, a patient comes, they say septicemia just like that but we sometimes try to explain to them when they seek for information* |
|  |  |  | *So I think when it comes to health care workers we need to think CMEs continues medication like right now there is someone who has a CMEs in about a month time and I am working with him to develop a presentation. So we need these recurrences, how does it affect the quality of care for sepsis, we should not give wrong medicine, we should not have wrong approaches in treating them, we should not have wrong diagnosis you get eh so we should empower clinicians through the CMEs so that they are able to identify, manage and treat appropriately, eh we should not have people with TB go home with fragile just because somebody thought it was a different illness* |
|  |  |  | *I think more training I would think…train them on triage because there is a problem at OPD, delays, also on ward, there are delays. Train on patient monitoring and talk, customer care and communication skills, empathy and things like that* |
|  |  |  | *What I would do next is maybe continuous education or you can keep getting new staff expect these recommendations keep coming up so having a continuous education of the staff* |
| 1. **Lack of guidelines and Job aids to manage sepsis** | No algorithms specific to sepsis  Need to provide guidelines | | *In addition to that probably on the policy level, I may say that on sepsis we do not have algorithms for managing sepsis patients in this hospital. These algorithms could give you step by step way of how the patients should be managed until when they are discharged so that we have a uniform management style for all patients* |
|  |  |  | *Provide the guidelines. If you asked how am managing sepsis, there is no clear guidance, or anything written down. At the back of our mind, there is nothing like sepsis. We know that there are specific diseases that can lead to sepsis. Sepsis is just an outcome of mismanaging an infection at its early stage. So staff should know how to manage as early as possible.* |
| 1. **Out of hours’ investigation are not efficient.** | Patients who arrive late after 5 PM delay because providers are changing shifts  Limited access to services after 5 Pm and on weekends  Patients are told that lab is closed after 5 Pm and on weekends  Specialist doctors leave early,  time lag between changing shifts,  Example of no admissions, wait until the following morning  Patient told to go back home and come back the following day | | *I went to the laboratory, we left and went to the lab because I had arrived here at five in the evening. So, when we went to the laboratory, we found that the lab people had left. I was told that they left because they stop working at five in the evening. There it was past five because we arrived at the gate at around four and we delayed at the reception, where we started from, we delayed a lot. That’s why we were late to reach the lab* |
|  |  |  | *I came here on a Saturday and we reached at about nine in the night, because I was feeling very sick, I could not wait till morning. I would have died, I was very sick, I was feeling pain in the bones, so then I worsened at night.* |
|  |  |  | *According to the time I reached here, most health workers had gone home, but there was that nurse on duty. So I didn’t get any treatment that day, there were no doctors, they just drew my blood and measured my pressure that night. I spent the night in that room till the following day. In short, I started getting treatment on Sunday, that’s when I started getting serious treatment after they had tested me and known the real illness that I had eeh* |
|  |  |  | *We delayed there, we sat and waited, I think we spent there like two hours. So what we did was to ask why, and they said that the lab people had left and after all it was a weekend. We wondered what to do, they told us that we had to wait until the following day on Saturday. We wondered about what we would do because I was in severe pain. We decided to go home but where we were going was very far and there is no way we would have reached there at night. And remember we are not allowed to move at night because of corona.* |
| 1. **Informal/hidden charges** | Some health workers ask attendants to pay some money to be attended to quickly  Need to investigate the issue of hidden charges  HWs tell attendants about how the services are free but again ask for money  Must pay some money to health workers if they want their patients to be helped quickly  Patients informally asked to pay for services that would have otherwise been free  Patients think that the HWs were selling drugs to them yet they are free  Patients think some HWs are selfish and corrupt  Patients suspect HWs to ask for money when told to seek services outside hospital | | *But the nurse said that we give her some money so that she attends to her and I don’t have money. After giving her 10,000shs she was very quick to help us and she wrote for us and she didn’t even ask about the book again. She then supported the patient herself and led us to the woman doctor who asked us what had happened and we explained to her and she helped us.* |
|  |  |  | *He told me that he wanted to check for two things. There was a machine which had got a problem but I don’t know them well. But he told me that if I want they check all the sickness in her, he will need…(makes a sign as if he was counting money)Now what I see is that at xxx hospital, we need there a very good spy so maybe that’s when they will stop those things of corruption which have failed the progress and this is what is making people say that Museveni is not working. They say he is not working yet the man is working and I’m his fun* |
|  |  |  | *I told him that I don’t have that money so what am I going to do? He asked me how much money I was having. I told him that me I did not come with money because I knew all the drugs are here and I can get them for free. Every day I hear some women there saying that anyone who asks for money should be reported to them. They were saying that no health worker should ask for money.* |
|  |  |  | *There was a woman from whom I asked for advice, because I had no money and I needed treatment. She told me that it is not possible because no one will buy for you medicine. I told her that I am going to give her twenty thousand since I saw her helping other people as I was seated there…. because I didn’t have the money, she continued working on other patients as I was sitting there. I told her that they had asked me to pay forty thousand but me I will pay her twenty thousand. She told me that we are going to be helped so I gave her the money* |
|  |  |  | *That they are going to check her from OPD. I asked him why he had asked for 15,000 yet I had 10,000. He told that he will plead with the person going to do the checkup. He told me that let him call the person as I sit here there waiting for him. He went did his work passed that side called him and after, he came back and told me that the man had refused the 10,000* |
|  |  |  | *So what you do, you just decide to pay something so that they also come and work on your patient. So that the doctor or nurse can work on her very fast. The nurse went and told the doctor that the medical forms have come, come and check the patient and we start removing the water. So, the doctor kept on saying she is going to come treat the patient but was not coming. That is when one of them told me; “why don’t you give her something so that he cares for you very fast?”. So if you want urgent help, what do you do? Yes, I paid 5,000 and they came and removed the water.* |
|  |  |  | *But I saw them telling him to pay the money and they start putting me on the drip. I think the money was theirs. Because we had to first pay it before anything could be done on me. There is no way those health workers could start treating me without first getting their share* |
|  |  |  | *And let’s say, when they are treating you, they are very welcoming. But these ones welcome you and after that they start telling you that we need this amount of money.* |
|  |  |  | *But what made me angry were simple and small things like those ones of money. You find that it is a small thing but the health worker you have run to help you is the one coming back to you asking you for money. That is the only thing that annoyed me* |
|  |  |  | *There is a drug that has been helping a patient and the health worker still wants to sell it to you and yet it was given by government for free. That’s is a big problem here. You find that something or a drug is available but the health worker wants you to first pay money. That is what I would like the hospital to improve.* |
|  |  |  | *I would say that may be the health workers don’t want to provide care, like their services are full of selfish people who are corrupt and want to be bribed before they work on you. Those are the things that I would be thinking about* |
|  |  |  | *Because I felt that may be because I’m poor, because I would see that those who gave in some little money {bribe] were being worked on. And after we also gave in some little money, that’s when they started working on me, that’s when they even thought of prescribing for me these drugs that they asked me to go out and buy* |
|  |  |  | *Patients can come and say, “Doctor I have come for this” then you tell them that is not done here. They think the services are there and maybe you want something from the pocket so as to offer the service. When we come on the ground and explain to them, some do understand. We also refer some to talk to administrators to talk to them* |
| 1. **Delayed patient monitoring. Delayed/ ignoring timely changing of materials i.e. cannulas and catheters** | Subsequent doses not given on time  Attendants have to remind patients  Being told that the prescribed drug is not there after it is late  HWs are informed and requested to remove or replace IV fluids but delay because they are busy  Skipped doses because the nurses are few and patients have to keep reminding them delayed treatment due to inadequate staff  Positive experience by one of the patients well monitored.  Patients are monitored till discharge  HWs asked attendants to remind them to monitor the patient when the time comes because they are not settled  Patients who need close monitoring are placed in closer beds  Fear that their condition may deteriorate any time  Patients with sepsis are reviewed twice daily depending on their condition  Observations not taken daily  Attitude of individual HWs  Nurses are Reluctant to follow the management plan  Doctors roles are different from those of Nurses  Patients miss getting treatment even when it is prescribed  Doctor prescribes an 8 hourly doze Nurse gives a 24 hourly doze  Example of a late prescription  Unable to take observations because the HW is alone  Untimely monitoring of patients can affect care and patients come become so sick because they have not got treatment on time  If observations are improved HWs can discharge more and more patients  Observations not taken regularly  HWs do not have equipment  HWs not able to tell if patient is deteriorating  Need equipment to monitor sepsis patients  Equipment only available in HDU and theater  Sometimes, HWs do not tell patients what kind of drugs are needed for their management  Monitoring it o whom it may concern  Staff have to take it upon themselves to learn monitoring even if they trained in nursing schools  Staff would talk to doctors about patient monitoring if given opportunity | | *I told the doctor that the drip is finished but I don’t see anyone caring for my wife since it stopped yet they were supposed to add one more drip. When doctors come, they bypass here. They worked on others but they did not work on mine so I said let me ask them. The doctor asked me what was the file name for my wife? They went to a room and got the file. They told me that the second drip is not there.* |
|  |  |  | *Since six in the morning, they only added her a drip, we have not received any other treatment. But they have been coming to check on her to how she is. They keep coming and reading his name, and we would say we are here, they continue to come and see how his condition is and then go and them come back and see how he is…* |
|  |  |  | *There was one doctor who was working on day duty I told that the drip is done and told me that I go and tell another doctor then they come and work on it and change the drip. But all the people I went to they said they were very busy, and there was one old woman who said that let me come, so they came and changed the drip but after a lot of time, so they could take very long to come and change the drip* |
|  |  |  | *I stayed with the bottle, there is a time when they put it on at about two in that afternoon and it got finished at around six in the evening and they removed it. Then they would come back to give you treatment the following day at 2pm. So they really take long to check their patients. They don’t come back to see what is happening* |
|  |  |  | *I was feeling bad and what else would I say? I would feel angry that I wouldn’t get the treatment that I required because the hospital cannot provide me with free drugs. Sometimes, we would delay to buy the drugs and get them late. Then my mother would go to the health worker after buying them, she would tell her that we have got the drugs. Instead of coming to treat me there and then, they would tell you; “the time for giving drugs is over, this is not the time to give drugs, we have finished”.* |
|  |  |  | *But I would see that when another patient comes, the nurse would go ahead and treat them. Sometimes, we would even try to ask the doctor why nurses were not giving us the treatment yet we had bought it. The doctors would also say; “we talked to the nurses but they were not responding and we don’t know why”. So I would really feel very angry about such things* |
|  |  |  | *Let’s say they put me on a blood drip at around two in the afternoon. Then I sleep, so once I sleep, there is no health worker who can come back and may be say let me go and check on this patient who was on drip. They can’t come back to say how is the patient? The drip gets finished, you stay with it the whole night, no one will come and say let me get the empty sack from the patient. No one comes to ask at night, you spend the whole night with an empty sack of blood till morning* |
|  |  |  | *The health workers would turn me, they kept changing my drips on time, they changed me from one side to another, immediately they would bring a pressure machine to take my pressure all the time. When they see that it is high, they would bring another machine, when these ones fail, they bring others, on the legs, the hands, what else should I say?* |
|  |  |  | *Technically like in my ward as I told you. When patients come, we have a specific part of the ward where we keep those patients. So they are aware. The moment they find them in that area because it is label for sepsis patients so the health workers know* |
|  |  |  | *First of all, as well, a hospital like this is a government facility and attitude varies from person to person. Me as a person I do it yes it is personal then other people some people just come write the file notes above with treatment. For example, the nurse has not yet taken the vitals, does not know if the patient is progressing, does not ask if the patient was worse the other day and today is better things of that kind* |
|  |  |  | *Usually the doctors have their role and the nurses have their role. So the doctors will do their role and finish now me I will see the patient. When I see that patient and put up a diagnosis and a management plan, it is the role of the nurse to help the patient go pick the drug and help the patient get those drugs. So if they have not done that part, the patient is not helped.* |
|  |  |  | *Because you are alone, you will not be able to take observations which is every important. Sometimes we do not give medication to them on time yet to give quality care; you have to give drugs at the right time. You may start like around mid-day and by the time you finish the 40th person, it is 4pm yet the drug was met for mid-day* |
| 1. **Delay to be reviewed by the clinicians and getting the bed** | Long waiting times waiting to be seen by a clinician  Delayed starting treatment. Patient spent a night without treatment  Patient spent more than 12 hours on ward before treatment could be given  Positive experience: Most attendants feel that their patients were attended too urgently when they were on ward  Example of a patient fainted in the waiting area while waiting to see clinician in the ward  Health workers carried collapsed patient to the bed.  Patients wait on ward before getting the bed  It means we are not doing well; it means patients stay in the line waiting for care…like the delays I told you; patients stay here for 30 minutes when the doctor is still doing ward rounds.  Delay because a decision to admit can be overturned on ward  Patient stays overnight  Procedures to admit patients such as clerking and prescribing drugs  Example of patients who arrive on ward after 5 Pm and are not recruited in the study yet they arrived early but delayed to be admitted  Patients come with prescriptions from OPD and can get treatment as the clinician on ward re-assess the patient  Sepsis patients have investigations that cannot be done immediately causing a delay  Patient can wait for 30 minutes before being seen by a doctor  A delay when patient is sent on ward and the doctor is attending to a patient on ward  Doctor compelled to halt award round to attend to emergencies on ward  Example of a delay; patient comes at 8am a decision to admit is made at 5pm | | *Yes, the challenges I faced was when I was taking her to the hospital when her condition was worse, when I was going for the test and to be given a bed. They were serving us in a line but the patient was very sick and I was seeing they were not reaching me soon. So they delayed to give me the letter and even when I reached in the ward, they also delayed to give me the bed and I spent there some long time.* |
|  |  |  | *He told me that you go and wait for me to come, when he came he asked me where is the letter that they gave you, then he said that this patient has not yet even been registered in the book, that’s why they have not yet given her the medicine. That’s when he took her in the room for doctors and started to write her details, and now we were as if we have just come in yet we had come like at 1pm and we were going to 5pm* |
|  |  |  | *I was looking for where they can put her, I would go to the doctors and he sends me to another one who also sends me to another, with my receipt and they were all telling me to go to another one yet my patient was very sick. They were there tossing us around. They were saying that you go there and when we go there we don’t find any bed there to give my patient. That night she slept on the floor without any medication.* |
|  |  |  | *I came at 9 in the morning, I spent the whole day without attending being attended to. I slept there without the health workers attending to me then at around 10 am the following day, that’s when they started to treat her and my heart was telling me that maybe I take my patient to a private hospital.* |
|  |  |  | *She told me that she cannot refuse to give my patient a drug unless it’s not there. In five minutes, they gave him four injections at once, they brought another drug and put him on cannula and by then we were going to 2:pm but they attended on him quickly but it was approaching 6:pm and I saw the patient was getting better and I said okay at least even this day has gone.* |
|  |  |  | *But you can come here like in that room and you find there many people, remember the doctor is only one in the room. There are these doctors…it happened to us sometime back when we came, we found that OPD was sending many patients to there ward. And when we reached here, we found out that there were many patients on the ward, and if you can stay there and wait, you see they are sending more, these were considered critically ill, those are the ones that were being seen urgently by the doctor who was sitting there in the room until my mother fainted*  *That is when the health workers themselves carried her from the floor and took her to the bed. So it is important that health workers are increased.* |
|  |  |  | *The doctor called us very fast and we brought the patient. The doctor asked how the patient was, her condition, and when the illness started. We explained to him as he was writing but when he finished writing, he immediately admitted us. Before we even finished laying the bed, the nurse had already arrived to start giving us treatment. Yes, the doctor said that what are these things they have brought for me and she said that this patient is dehydrated, we need to hurry up and go so that they give her a bed* |
|  |  |  | *I stayed here on the male ward. The health workers told me to sit there at the waiting bench, I gave them my letters which I had come with. They first reviewed my letter, and told me to wait, they didn’t do anything on me. They started asking me questions. Then I told them that they shouldn’t be asking me when everything had been written in the letter that I had given them. The good thing, I told them that I was feeling severe pain in the abdomen. One of the health workers told me to go and sit on one of the beds.* |
|  |  |  | *I sat on the bed…I spent almost 30 minutes waiting for them to decide. So after sitting on the bed, one of the health workers came and told me “hmm, how can we deal with your illness, your illness requires money”. So she said, “eeh what do we do?” I stayed there for long before they started treating me* |
|  |  |  | *I have seen people sleeping on the floor, I have seen them line up the whole day without lunch, just waiting to be seen by just a healthy work or waiting to go home with some medicine that is why they sacrifice their time they queue up and wait. Those who are admitted can even sleep on the floor but waiting for those two things.* |
|  |  |  | *I think there is a delay there because sometimes the patient may come maybe passed let me say 5pm and the decision at OPD is to admit the patient. They stay overnight but that decision is over turned so they will delay on the ward spend one night. You know the cost of being in the hospital only one night then the decision is over turned. It really causes delay to the patient* |
|  |  |  | *Now sometimes you get stuck to screen this or not because at this point a decision has not been made at the ward but has been made at OPD and the patient is on the bed already so at some point we also get stuck in this so I have to wait* |
|  |  |  | *What we get from the patients while they come on ward because we have some exclusion criteria where we do not admit patients who came after a certain period. We do not recruit those who have come past 5 pm but when we tell them that this is the reason why we are excluding you they will assure you that they came in the morning so that is how we can tell the delay. We ask them what time did you come by now we excluded you because we know you have delayed so we have got so many interventions so they assure you that they came way earlier.* |
|  |  |  | *The system its self we do not have enough clinicians on the ward so you find that some come and there is no one to see them even right away from the OPD people come to the hospital as early as 8 am. The decision to admit gets to be done late in the afternoon or in the evening at 5pm and most clinician have already gone by midday by 2pm and you find the first medicine the real medicine that they are to get is got the next day so that is the challenge. It is basically system deal that is and we do not have enough clinician to see people in time even the system to start treatment is usually delayed* |
| 1. **Emotionally traumatized with frequent deaths in the wards** | HWs care less when patient dies  Attendants uncomfortable when dead bodies are placed in the veranda of the ward where attendants rest from  Fear that they could lose their loved ones too  Not helped when lost her patient  Confusion after losing the patient  Traumatized by referrals to XXXX | | *Another thing is that when someone dies, health workers do not care. What I think they should do is to care for the dead body. Most times they just put dead bodies there either in a wheel bed just there, bodies stay there for a long time, then they tell us to sit in the shed because of the sun and you find the bodies are still there.* |
|  |  |  | *They placed the dead body outside there while other people are sleeping. No. they put it outside there yet people were saying that they isolate them. So they should find a place to isolate dead bodies so as people do not see it.* |
|  |  |  | *It disturbs because when they put it there, they spray it. You find that people can not fit here so the seat there at the entrance since there is sunshine outside.* |
|  |  |  | *I was thinking that my wife could leave me anytime she could die.* |
|  |  |  | *When I was at the hospital I saw people dying here and there and my heart skipped and I said oh my God, now see this one has gone yet he was next to my wife, and now my heart would pump faster. I said dear God help me saw that my patient gets well, not to die, but you could see the other person has died already and I could see my wife crying and even me I could start to think a lot in my heart since we were at the hospital I would be worried and ask myself that now what should I do for this woman? I don’t have money now how will this woman feed? So I would make sure I run very fast and buy whatever she would ask for. There was one day when I ran three journeys to the market.* |
|  |  |  | *So that actually made me confused and I didn’t understand it. Because if a person dies in a government hospital, they wrap and treat the body and then give it to you. So I had to pay for treating and wrapping my own body because I had never attended to a patient and he or she died in the hospital before. This was indeed my first time, and I didn’t know all these as a person.* |
|  |  |  | *For the time I spent here, I would see people dying in this hospital and the bodies would be taken outside the ward. But I didn’t know that when your patient dies, the same health workers on the ward will be the ones giving you phone numbers of the private people that they know to come and treat the dead* |
|  |  |  | *ut I also thank God for that. There were also patients, whom they would bring to the ward and as soon as they would get there, they would die even before they start treatment. And you see that your patient has also survived for another hour and yet she has been in that critical condition. So the health workers tried their best* |
|  |  |  | *Yes, there are patients who are taken to XXXX. You see that the health workers have failed and decide to send them to XXXX. That is scaring to me. I start thinking; what if my patient is also sent, will I manage the costs? Do I have the money? Hmm, that is all* |
| 1. **Some patients come without attendants/ vulnerable patients** | Should go next to patient. Caregiver relationship  Causing more germs  Need help and care from the HWs  Not sure if such patient would recover well  Hospital should provide necessities for the poor  Recommended that the hospital can have a fund to support the needy and helpless  Some caregivers of critically ill patients run away  Patients who need specialized care become difficult with no attendant  Nutrition department supports such patients with food | | *I’m talking about my neighbor who has been vomiting and she has no one to help her, I have been helping her to tell the health workers to come and help her so that she can be treated. The other neighbor has been helping me a lot. And I have also been helping her. Like when she wants drinking water I give her and she drinks. When my patient was getting worse and needed to turn, she was helping me to carry her and turn her. That’s is it. Some patients were being getting worse and were referred to xxxx… and I saw one being taken…I have forgotten the name of the patient that has been transferred. Eeh like I have my neighbor on the other side, she is not very sick but the nurses are the ones who are cleaning her, that is what I have seen, it is hard to clean someone who is sick and has no attendant.* |
|  |  |  | *And is the patient really going to recover well? You find the patient is vomiting but he is not going to wash. So that requires the doctors to teach about hygiene which is brought about being unclean and being poor because the person will come and they cannot afford, he or she does not have the requirements to go with to the hospital. That alone brings germs from his or her bed to other patients* |
|  |  |  | *Now concerning hygiene, there are patients who cannot help themselves. You may find that they have brought a patient who has only two bedsheets. And the patient is on those two bedsheets the whole day and the whole night, not even washing them* |
|  |  |  | *Still in our ward, there was an old woman who was abandoned by her children.* |
|  |  |  | *May be what I had forgotten, there are patients who come here and they don’t have people to take care of them. You find that someone was brought in by an attendant, but when the situation gets bad, the attendant runs away* |
|  |  |  | *I was suggesting; can’t this hospital have a fund to help such people who have been abandoned? Because the old woman was really going to die and the students were coming every day to lay her bed, to clean her and even change her. So she has improved. But if we had not collected money, she was going to die of hunger and thirst!* |
|  |  |  | *I don’t have the opportunity, but I ask government to continue looking after our hospital because there are people who are poor and cannot afford to take care of themselves. The government should bring bedsheets on the patient beds so that when the poor patient comes, he or she can be given a pair of bedsheets to change because he or she is poor and cannot afford.* |
|  |  |  | *Mainly those patients who need specialized care, or need to be seen by a physician and if he is not around, what do we do? Sometimes there are patients who come by themselves or brought by boda boda people, the reach here and they are admitted and have no attendant. Those are the ones we discuss.* |
|  |  |  | *There are patients who come without caretakers and they are either brought by police or someone walks in here without a caretaker yet the condition is badly off. So we go through our nutrition office with support from the social worker to produce them some food and milk while other will ask why they are giving specific people* |
| 1. **Gaps in providing Health education during admission** | Not taught about the patients’ illness  Attendant needs to be taught what to do when patient is discharged  Not taught anything  Attendants should be taught about the causes of the illness  Those who were taught:  Topics  HIV, nutrition and feeding  Health talks are given and assurance that patients are in safe hands  Health talk given individually in some cases according to individual issues  Patients taught on prevention, good diets and recovery  Discharge counselling emphasize timely taking of drugs and coming back for review  Health should encourage patients to go to nearest HC  Counselling services given on HIV  Education on the importance of staying in the hospital  Need to tell patients what they are suffering from. Causes and management | | *I really don’t know, but I did not receive any form of education since I came here in this hospital* |
|  |  |  | *The health workers should teach us what we should do at home so that the patient doesn’t get sick again. It can help us to avoid coming to the hospital every time, because even if you come to a government hospital, it is like you are in a private hospital, you keep paying, paying money for drugs. If they can atleast put drugs in hospitals, so that we the poor don’t have to buy drugs* |
|  |  |  | *About the old man, they only treated him they didn’t teach me anything and if they had taught me something I would say okay. Now what doctors can educate patients and care takers in the ward I really don’t see it because they have ever educated them about everything and even the people themselves see it* |
|  |  |  | *Then after, your patient will recover and if he or she has diarrhea, they should teach him or her about the kind of diarrhea that they have and what caused the diarrhea. They should also ask the patient when the diarrhea started, for how long the patient has been diarrhating or if it is vomiting, for how long has the patient been vomiting and why it this patient vomiting….and for how long?* |
|  |  |  | *And the water that was piled in the stomach they should have given her other things and they also tell that don’t eat this food and that. Yes, those are the ones I would like them to tell my patient maybe I would also buy and give them to my patient.* |
|  |  |  | *Yes, they taught me about HIV/AIDS. When you said you wanted to talk to me about my experience, I thought you were the same people who were telling us about HIV. They taught me and later told me to go for HIV testing. When they checked, they did not get any problem with me. HIV was not there. Another thing I know is the time for taking in medicine, how to take it.* |
|  |  |  | *So while we were here, they removed the water, they told us to reduce salt she should start eating cold food. Before we left this place, she started eating and they insisted that she should eat cold foods. It means food without salt, so we stopped giving her food with salt,* |
|  |  |  | *I also give them health talk, ensure the patients and care takers when they come the first time that they are in the right hands and they should not worry. I also do counseling and guidance depending on the issues they have.* |
|  |  |  | *We also do health talk. In mist times I do it on individual bases in the ward because everyone has issues to address and sometimes because of the other challenge of the language to address them to understand me. We do not give it generally in the ward* |
|  |  |  | *I had forgotten to tell you that when we reach on the ward, we health educate our patients very many things for example the malaria patients we educate them about prevention of malaria in their homes and for cases of diarrhea we tell them to boil water for drinking and sleeping under a mosquito net. We also teach our patients about the good diets to help them prevent diseases but also for recovery.* |
|  |  |  | *According to the issues I have identified and which need attention Yes, especially hygiene. Sometimes people who have hypertension, diabetes, I usually give them health talk depending on the condition they have like the diet, how to take drugs and the hygiene. I usual do it on discharged.* |
|  |  |  | *Before the patient is discharged, we actually give health talk on arrival. We tell them that when they are here, their patients need A, B, C. we explain to them there part and our part. At the course of the treatment we also observe if the care is going on well or there is a gap. When we find there a gap, we also address it until the patient is discharged.* |
|  |  |  | *If we discover that our patient is not improving, we can have some changes depending on the investigations. To provide quality care, we also need to do health education talks. Thus means we shall health educate the workers themselves, patients and caregivers on how to minimize sepsis. It is happening but not in all areas. There are health educations that are given to the public. I have also seen them in the antennal and OPD side. In the wards, it is usually one on one and this happens when there is need.* |
| 1. **Some patients appreciated the periods of waiting time** | Not waited for long  HWs were quick to work on the patients  Did not wait in the queue | | *So, we reached here at eight in the morning and there is a health worker who came and gave us a number, she looked at the patient’s condition and the fact that she was very sick, she picked us from there very fast and took us to a room and they examined her and after that they admitted her. When they admitted us, even here on the ward, they worked on us very fast and they gave us good care and we appreciated and were very happy about it* |
|  |  |  | *They are doing their work well and very fast because they don’t have any other way in which my mother would have improved, because we reached here, they worked on us very fast and they treated us. I haven’t seen anything wrong. The situation was very good. Considering the way, we left the village, we were given better care compared to what we expected. Yes, the health worker took us to the room and the nurse came and quickly examined. After quickly examining her, she sent us to another window where they were going to give us the book. When we reached that window, the nurse realized that the people inside were not being fast enough. The nurse went inside her- self and came to the window they placed a number and they sent us here* |
|  |  |  | *We didn’t spend a lot of time that day. When we arrived in the ward, they responded very first to start me on treatment. The health workers worked on me very urgently, except that we had to buy the giving sets for the drips. The following morning, they also came to treat me, we bought the drugs that they had asked us to buy and they treated me.* |
|  |  |  | *The health workers still. First when reported at the reception in OPD, they registered me very fast. And when I gave them my letter, they immediately sent me here. I heard the nurse saying that this is a referral case and the doctor said let her go straight to the ward. That one made me happy. At first, when we reached and found a line, I said … “Oh God, when will this like be finished”. I used to hear people talk about xx, that things of xx, you can die in the line. So when I reached here, that is not what I found. I went straight and when I reached the ward, they put me in that room in the middle, doctors came and worked on me immediately.* |
| **Description of good quality care** | 1. Being given proper medicines, shown care 2. Being cared for by HWs, attended to quickly, and given treatment 3. Being able to recover 4. Spending less on medical care 5. Having more than 2 staff to do the scan and prioritizing sepsis patients so that they get urgent help 6. Being examined and tested, identifying the illness before being treated. HW explaining how drugs should be taken. 7. Being asked to discuss if patient will manage the treatment given 8. Having testing equipment in the laboratory, having enough HWs available during the day and night 9. Providing free bed and beddings, curtains 10. Qualified Health workers 11. Enough drugs for difficult diseases 12. Providing equipment 13. Being quick to attend to very sick patients 14. HWs wearing their uniforms 15. Disciplined HWs who try to understand the patients’ problems 16. Being seen by qualified HWs 17. HWs respond immediately 18. Caring for the patient 19. Providing urgent attention 20. Patient improvement 21. Welcoming the patient on arrival 22. Giving the patient assurance of being in safe hands 23. Providing patient care 24. Good words and attitude towards the patient 25. To treat patients like human beings 26. Not changing the mood. Making the patient feel comfortable, giving them a chair, telling them that the doctor is coming 27. Having fewer deaths in the hospital 28. Timely review and monitoring of patients 29. Timely giving of treatment 30. Recovery and survival of patients 31. Fluid management in the first two hours 32. Providing care at the gate 33. Patients knowing here to go 34. HWs attending to the patient fast 35. Patients not waiting in the line 36. Availability of wheelchairs 37. Knowing which ward to go to 38. Someone to direct patients on ward 39. Patients are given drugs, treated and discharged 40. Giving drugs to patients as prescribed 41. Patient survival or recovery 42. HW building rapport with patients 43. Technical, Process and quality care | | *Good quality care is when you come here and they give you medicine, care for you and proper medication.* |
|  |  |  | *The things that show the good quality medical services is when they reach you at the hospital, they should be caring about you. When you take the patient there and you reach the hospital they should be rushing to quickly receive you and put the patient on drugs quickly.* |
|  |  |  | *The good quality care… now like with my child, if I have gone there when I’m sick or when I have taken there my sick person, they should get the drugs and give me. They treat my patient well and they recover without spending a lot of money. If they give me drugs, the money for buying the food that one I can spend it because it’s my duty to feed my patient. But if they say that I have to buy all the medicines, even the drip you buy it; even the malaria testing kit I also paid for it because I bought it at 4,000shs* |
|  |  |  | *She will assess you and tell you that you will need injections and today we will give you three injections, and she will tell you that at midday we will add you another injection, then at 6 pm we will add you another one. And they do that on time as the doctor has instructed them. The doctor should also ask you whether you will manage to swallow those tablets or you will have problems. And then he advises you and tells you that may be use milk or juice or soda. Now isn’t that a good quality care to enable one want to come and receive it from here! That is the good and most important care because you will go there with a lot of hope. But if you go there and they all look at you, will that be the good care?* |
|  |  |  | *A hospital that is providing good quality care, is that hospital which has everything and if it a government hospital, it should provide them for free such as beds, bedsheets, curtains, it should have trained health workers who are teaching interns, it should have enough drugs for difficult disease, it should have equipment to test and good hygiene like a home. All equipment should me modern, not these ones where they tell you the machine is there but it is lacking certain things.* |
|  |  |  | *But the good care now would be that they work on diseases like ours. In the scan room, at least there should be about two people so that they can help the patients that are critically ill and they do what? The help them from here so that they can get urgent help that they need.* |
|  |  |  | *I saw, just health workers coming in and going out, dressed in uniforms, carrying trays and also students wearing green and blue uniforms* |
|  |  |  | *A hospital that has machines that test everything and every disease; blood, x-ray, scan and drugs, that is when I would see that the care is of good quality. Also when the doctors are around all the time and also the nurses are there day and night, that is when I would see a hospital as a quality hospital. If there are machines like those in the lab, that test everything, that would be quality care because the drugs are also there* |
|  |  |  | *What comes into my head is that when you go there they attend and treat you very well, that’s what comes in my heart. That’s what I think is good care. But most importantly I want that if I take there my patient, they should attend to her very quickly they take her to the bed and they inject her, there I will be happy in my heart.* |
|  |  |  | *Then another thing is that the doctors should be putting on their medical court. Yes, so they should be putting on uniforms because one day they told to go and talk to doctor is there but I looked for the doctor but I failed to allocate him and I by passed him. So if you are a doctor you are supposed to put on your uniform because me now I bypassed him and went to another one until they told me that I told you to go to that one, I didn’t know him because he was putting on casual clothes. So that one is also very important and it should be there because some doctors don’t mind because people can’t reorganize them* |
|  |  |  | *The qualified doctors assisted by students, not leaving students alone to see complicated cases like ours. Students should only treat malaria.* |
|  |  |  | *What they call care is health workers, they are the ones who bring quality. You can have well educated health workers but they cannot put the hospital to say it is of good quality. Quality care depends on the behavior of the health workers because if I tell the health worker that I have this pain here like this, I need treatment.* |
|  |  |  | *That’s why I’m saying that I have appreciated this hospital. If it is treating, they are treating, they are disciplined, when you call someone, he or she comes and reviews you, asks you questions and he understands your problem, if he can help you he advises you or he goes to fellow health workers and asks* |
|  |  |  | *Good care, and the patients who are coming to the hospital are being treated very well and the health workers are discharging them. I also came here plus the other people that I found here and the left me here, it means they received quality treatment. The moment you come here dying, screaming, unable to talk, can’t eat and helpless, then they work on you and you wake up and be discharged, it means there is quality care* |
|  |  |  | *Quality care to me is the care you give to the patient especially when the patient needs your agent attention and the patient improves from the time the patient comes until the patient is discharged. Improves from the care and appreciates it. That is how far I can explain* |
|  |  |  | *The first thing is the way the patient first arrives and the way you the nurse welcomes the patient. You first assure the patient that she is in safe hands even if she is very sick. You welcome him and assure him that he will get the best services. The first message you give as the health worker to the patient is the word that he will be fine. Secondly, the treatment you are going to give the patient are your words attitude towards the patient which will enable him to be okay.* |
|  |  |  | *Yes, there is a drug given the way the patient has been reviewed or checked every time. The time of giving the treatment so I think in most cases that is all.* |
| ***Description of poor-quality care*** | 1. *Lack of drugs having some drugs for particular illnesses and lacking others* 2. *When very sick patients have to wait for services for long hours* 3. *Attendants not being in position to maintain the toilets clean* 4. *Being worried of getting more infections from poor hygiene* 5. *When HWs refuse to wake up when called upon to help a sick patient* 6. *death of patients presenting with infections* 7. *When people wait for care and no staff to attend to them* 8. *Failure to carry out tests due to lack or reagents* 9. *Poor conduct of HWs, failing to provide care on time,* 10. *Not educating patients* 11. *Abusing patients which causes psychological problems and mental problems* 12. *HW unethical behavior* 13. *No giving treatment, being inconsistent with observations* | | *Yes, it is poor because there are no drugs there. You can’t find them there. Well, some drugs are there but some are not in so that is what puts it to be of poor quality because the drugs are not there. So even if you go there now, the drugs are not all there but if you go to the private, the drugs are there. So this is what makes the hospital to be of poor low quality. Yet you as a patient you go there hoping that it will be there.* |
|  |  |  | *Already I have told you how we have been sitting for hours waiting for the scan and yet the patient is very sick. Instead of saying we are starting with those that are very sick, they follow the order in which patients came. That is poor care. If the attendants are making the toilet dirty, and they are not clean and every time you fear to use it, I think it is a bad image for the hospital, they are very many. If a doctor says that you should give this patient an injection at let’s say at midnight. Then the nurse puts a drip and goes away, midnight comes and you wake her and she refuses to wake up, isn’t that bad care? It is. Except that this is a government hospital, you cannot complain a lot.* |
|  |  |  | *The poor-quality health worker will tell you no, I feel tired. That patient will take a report to another person and say, “ah, that hospital doesn’t treat people well, health workers do not care, people die there” like that. But such health workers always want to show that they are educated.* |
|  |  |  | *You can go to a hospital …okay there are government hospitals where they tell you that the drugs are not there* |
|  |  |  | *Yes, it means, they will just look at you, they will shout at you. Even if your attendants goes to tell them something, they will just ignore him or her. But this hospital cares, and the quality is good but not very good* |
|  |  |  | *Sometimes, someone thinks that they have abused her or him as the nurses talked to her, that makes them think that the quality of nurses is not good and once nurses are rude, unfriendly and cannot treat the patients as human beings, it means patients will fear to go there and that’s poor quality* |
|  |  |  | *You may find that the hospital doesn’t function very well and yet they have everything. So it depends on the behavior of health workers. You may find that the health worker is proud, when you try to talk to them, he or she tells you to live her alone, “I’m tired”.* |
|  |  |  | *Yes, when a patient dies, it means you are not giving quality care. Why should a patient die with an infection? When she dies of an infection, you are poorly performing. Because infections are the easiest to treat with antibiotic. Severe infections are not complications and not chronic diseases, so we must manage them even if the patient presented late.* |
|  |  |  | *People are waiting for care but there is no one. Even there is no one to start assessing them and they are seen after two days. People may come on a Saturday then real care is done on a Monday, but these are two days in hospital admission, travelling to come and see them a lot of effort is being put in by the patient to come and seek for care and it will take those people 48 hours to get. I think it is poor quality.* |
|  |  |  | *Poor quality care would also include failing to test patients because you have no reagents. It means the hospital has failed. If the patient is tested let’s say and has sepsis and you can’t provide free drugs in a government hospital, that is poor care* |
|  |  |  | *There are also issues to do with professional conduct of health workers; keeping confidentiality, communicating with patients, providing care on time, not just care. If you don’t do these then your care is the poorest. Do you know even failing to explain to the patient or not educating the patient is a poor quality care? So it’s a big concept, talking about it will consume more of my time. Remember I gave you only 15 minutes and now you are remaining with 5 [laughs].* |
|  |  |  | *Yes, things like not giving them treatment, abusing them brings psychological problem, mental issues. May be I used a wrong term; being unfriendly; rude, refusing to explain to the patient, shouting at patients, chasing them from the ward by force and other unethical behavior.* |
|  |  |  | *Not giving medicines, being inconsistent with the observations. If they say every 2 hours and you do every three hours, it means that’s poor quality work.* |
|  |  |  | *Okay but they may be right because what they say depends on how they were treated. May be they are not treated like me. Someone may come and the hospital doesn’t have anything and everything you have to buy, testing you go out, drugs you buy and in other places, doctors ask for money to treat you.* |
| ***The priorities of patients*** | 1. *Being tested before treated* 2. *Being welcomed by HWs* 3. *Being asked questions about how the patient is feeling (taking history)* 4. *Being told the cause of the illness* 5. *Being given medicines* 6. *Security* 7. *Access to water* 8. *Getting care for my patient* 9. *Being assessed and explain the results, getting immediate treatment* 10. *Recovery of the patient* 11. *Being discharged* 12. *Being taught how to maintain hygiene* 13. *Cleaners able to maintain the place clean* 14. *Being registered quickly, getting a bed quickly, starting treatment quickly* 15. *Not waiting in a line* 16. *Having enough and qualified doctors* 17. *Accessing water* 18. *Getting the drugs enough for all patients* 19. *Equipment for testing patients* 20. *Fear that patient can die on the way to private labs* 21. *Testing very sick patients on the ward* 22. *Being put on treatment* 23. *Stop the patient from death* 24. *Doctor to see very sick patients’ first* 25. *Get a bed, being examined knowing my illness* 26. *Being able to recover* 27. *Being discharged* 28. *Free treatment services* 29. *Not paying for any service* 30. *To receive free and rapid tests at the hospital using modern equipment and for free without being told to do the tests outside of the hospital. HWs to explain the test results and on time* 31. *Receiving support and comfort from friends and relatives* 32. *Being treated equally* 33. *Patients have protection needs* 34. *Patients need their issues addressed and their health improved* 35. *Communication, direction, patients diagnosis* 36. *patient always ask doctors* 37. *Attendants need a health talk to know what they should know* 38. *Good customer care* 39. *Being taught on HIV prevention* 40. *Have their health improved* 41. *Expect free drugs* 42. *Being seen by qualified doctors* 43. *Getting and knowing the test results* 44. *Being cared for by nurses and doctors* 45. *Free services* 46. *Being talked to in a polite way* 47. *Seeing the patient improve* 48. *Being welcomed* 49. *Free services, seeing a clinician* | | *I want that if I am sick they test and see which diseases is disturbing me and they treat me very well and if I want the drug, they give me the drug without asking me for any money like 100,000shs or 80,000shs.*  *When I come to the hospital I expect to receive a ‘welcome my patient’ and then the health worker can about what am suffering from. After that, I will explain to him what happened to me and I assume there will be machines to test what I’m suffering from. I may have fever and yet there is a cause for that fever. So if I come and I tell you I have a fever, because you have the machines to test the fever, you are the one who will tell me that you have a fever but may be it has resulted into may be pressure or the highest heartbeat. Like my mother was hit by a stroke and that was caused by high blood pressure and the heart*  *What I would think is first is the medicine to treat my patient and to care for the patient. The second thing is defense, thirdly hygiene then last water…Taking care of my patient should be the last one*  *Now like water, I need to find it here and I use it. There is no water and the toilets are dirty. I want the toilet to be clean with water. Remember the toilets are for flashing and there are people who do not know how to use such toilets. What I have also seen is that the cleaners do not know their work. They clean at 9;00 and they don’t do it again.*  *I mean to see that you are being cared for, you get relieved. Do you see those private hospitals? They really care for you, someone comes and cares for you even if you have paid this little money of ten thousand, they really care! As soon as you come in they show that they care and that care compels you to go back*  *What I should find in this hospital is that they should take my pressure, they should explain what they have got whether it is low or high, and whether it is stabilizing. If they find that it is very high, they should get him immediate treatment to see that the pressure goes down*  *I have been seeing patient coming here very sick, they get treatment and they recover in a day or two and they are discharged and go home. It is encouraging because you get encouraged and become hopeful that your patient will also recover like the ones that have treated and sent home. Eeh you keep trusting God.*  *The truth is, whenever I come here, I expect my patient to be treated and to recover completely. At least if they could treat the patient and she totally recovers without coming back to the hospital. Then they can discharge us at once and we go back home*  *I want to see that my patient has recovered and is well. That’s all that brought me here What I am happy about it that the patients are being given treatment, they are giving them drugs and they are recovering.*  *Eeh yes, I expect my patients to improve and be healthy, in that way, I expect to be discharged and go back home in few days*  *Hmm, you see people leaving they come and tell you that we have been discharged, you feel happy for them. Because you don’t want anyone to die. So that shows that the health workers are treating well*  *What is supposed to be done for the patients is when the health worker educates the patients on how to use flash toilets. The truth is that there are some people who do not know how to use them. At times water is around but still people just defecate anywhere. Even the cleaner should also clean in case they cannot manage they should quit. At times a patient also comes and she does not know how to use it. The health worker should teach me how to flash and how to keep the toilet clean.*  *Yes, we get the drugs and they also care about us. But the issue of caring about patients is very important. Yes, like if I have come with my patient, you quickly register her and give her the bed and the drugs quickly. Yes, that’s what it means to care about me. To give me the bed quickly and then and the drugs, but now when you reach there, they put you in a line and yet others who have not been in a line they come and they bypass you and go and receive the drugs.*  *The first is the drugs, and the second is that the doctors should be there and enough. Then the other one is that all the doctors should be knowing what to do. Then the third one is that there should always be water in the hospital.*  *The most important thing that shouldn’t miss are the drugs but me I have not really been getting them from there. But those that get drugs from there have their special time they go there but they tell you that sometimes the most expensive drugs are never there, and yet for us we think those drugs are there. So that is what I was thinking they should put in the hospital so that people can come and get them from here instead of them saying that those drugs are in Kampala. So they should also stock those drugs so that if I get such a dangerous disease I can also run there quickly and get them.* |
|  |  |  | *The truth is, the machines that do the tests are very necessary because those things of saying that let’s first go out there and look for the labs and then come back, the patients can die while you are still on the way. Those machines are very necessary. At least even if I buy drugs, as long as the patient is tested, I would rather buy the drugs.*  *The most important thing in XXX…if the hospital has equipment for testing all diseases especially for patients like my mother, and other patients who were very sick, then it becomes easy to treat them. If we have enough drugs in XXX… But if those expensive drugs and tests for our patients could be done by the hospital, then it gives us hope that the hospital has what it takes to treat those difficult diseases. But without drugs in the hospital, the truth is the life of that patient will always be in danger. But sometimes, you can go to a hospital and you find everything there and still die. So what is important is health workers to have all those things and provide them for*  *The issue of having no testing equipment to test patients. At least they can say we will only test patients who are on ward. Because they cannot walk, those who come here come when they cannot walk at least the machines should be there to test them for free and given the drugs for free. Those two issues are very difficult for both the patients and the attendants. Someone looks and says; “I have to go to XXX, I have to be with some money. And if I don’t start with money, I won’t receive any care because I have to pay for tests and drugs”.*  *I expect to find that the health workers can put the patient on treatment very fast so that she doesn’t die. When I come here with her, I want her to be put in drip very fast so that they also give her blood and other injections. Not to wait here when the doctor is seeing other patients who are not very sick like mine. Hmm that is what I expect to find in this hospital and the health workers too*  *First of all, I expect that the patient will be admitted and put in a bed, they should then examine her and take her to the lab and know what she is suffering from. After testing her, they will know if he or she has malaria or cough or flu or has diarrhea or she is vomiting and then that’s when they say…they begin to give him or her drugs or pills to swallow or they give her an injection like*  *You just see that the doctor has treated your patient very well, you see that your patient has recovered, it means you patient has been healed and has left the hospital and gone back home to do her usual work*  *To find that there good medical treatment without asking me for money so that even me when I reach there they can give me a malaria drug, ah they can give me the deworming drug and even other things that I need, they give me. It’s because of my sick legs my child all my children hate going to xxx, now they take me to the private hospital but I ask them to take me to xxx hospital. But they say let’s take you to xxx they check you and they just write for you the drugs because they will not give you any drugs. I don’t know where they put that medicine?*  *If am a patient, I expect everyday someone to come to check on me if I am a patient then let someone tell me what happened to me, what was the cause,*  *Yes of course being reviewed every day. They want their patients to be reviewed every day. Even if they are reviewed only once, they will tell you, “The doctor came in to see my patient and this is what he said.”*  *Patients expect a lot from us especially in terms of treatment, communication, directions since most times they do not know where to go. This is because it is there first time. When they are sent to where to test from, you are supposed to direct them. At times there are things they need to know from us as the staff. When they come, the way you talk to them will be the way you impress them. At times patients also what to know what their patients are suffering from which means you have to be confident and tell them the right thing. They usually do not go to the doctors but they ask the nurses. Even when the doctor is doing the ward round, they will not ask but come later to ask you.*  *Caregivers need health talk since there are few who know what they are supposed to do but still there are others who still to be told. There are patients who are very sick and they need protection of the caregiver.*  *If am a patient, I expect to be taught how to prevent the disease and how to protect my family, what I should do, how I should take my medicine and am sure that’s what they expect from use health workers.*  *After getting those, I want the staff to be friendly and polite to me, keep monitoring me because the equipment and drugs are there, check my vitals as recommended by the doctor and even respect me the way I respect them It’s not us, it is the situation. So that’s a big challenge. So if am a patient, I want to leave here when I feel I have regained my energy and even walking. Not getting a wheel chair to be taken home. Do you see what am saying?* |
| ***Things that are working well*** | 1. *Patients recover and return home* 2. *Partially getting free drugs and being asked to buy some* 3. *Patient treated and cared for on time* 4. *Receiving very good care* 5. *Being cured* 6. *Being discharged* 7. *HW working hard to save the patients’ life* 8. *HWs communicating with patients and telling them what they should do* 9. *Being motivated to do their best for the patient* 10. *Hospital environment being clean* 11. *HWs treating the patient with one heart* 12. *Appropriate care* 13. *Happy that very sick patients sleep near the nurses and are well cared for* 14. *HWs treat illnesses that they know after conducting examinations and tests* 15. *HWs prioritize emergencies* 16. *HWs advised patients on alternative drugs to buy* 17. *HWs come back to discuss with the patient challenges in taking treatment* 18. *Nursing care, drug administration and communication with attendants about requirements for patient management* 19. *Nurses take samples from patients and ask attendants to go out and carry out the tests* 20. *At OPD, all patients are triaged* 21. *Presence of clinicians to see patients before admission on ward* 22. *Most patients that come with sepsis recover and are discharged* 23. *Presence of a good laboratory to do most of the investigations* 24. *Well-equipped laboratory and good lab results* | | *The thing that went well was to see that they treated her and she returned home well because when she was going there I didn’t hope that she was going to come back I thought we were going to spend there a lot of time but when we went, we spent there very few days and they discharged us and we came back, so those are the few things that I never expected to find there.*  *What made me happy is when I took a paper where they gave me two drugs and they told me to look for the other from the pharmacy. Atleast they gave me those two!*  *Okay, they really cared for the patient, they treated her on the real time when they are supposed to do it. They take the responsibility at that time. If you are outside, they look at your file and at the appropriate time, they call you. And if the drugs are not there, or if they are there, they can do what? They can treat you. That’s all that made me happy*  *The other thing that I’m happy about is that the care is really good. If the health workers have drugs, they treat people here and they get cured as long as you have followed the health workers’ instructions. You can do that and the patient will cure*  *The patients would be admitted while am seeing, they would be discharged when I was seeing. They find you in the hospital and leave you there. I never lost hope. I knew the health workers were working so hard to save her life.*  *So when the health workers are available in the hospital, he or she will tell you “oh you are sick, go and get a Panadol, we will be getting for you other drugs later”. Don’t you see that such a thing motivates you? The health worker can say; “if you have money, first get a fragil and we work on you”. That alone motivates you. But when you come to the hospital and you find it just there without health workers, aah you end up losing morale, you may not even spend a night here because even if they admit you without health workers, they will have done nothing*  *Those are the only things; they treat the person very well, the hospital is very clean, what else would you say, they have improved everything*  *The other thing that made me happy is that they treat patient with one heart. We came, they were happy for us and they gave us treatment. The way a person gets happy, you compare the way the person came here, you see how you were taken care of, that’s when you see that the person is happy. Even me the caretaker of that patient, I feel happy!*  *For me, I got whatever I expected from xxx hospital. I have found them here. I have found good treatment. I already talked about it that good care is when they patients has received treatment and has not died in the hospital; the person has recovered*  *Good care that I received…they took care of the patient, they never left me alone, and they gave us drugs on time, the patient got all the drugs he needed on time. That has really made me happy, because I didn’t expect that I would get that from here. So when I got it, I felt happy.*  *But when you are very sick and about to die, the nurses put you nearby. Even me, I was sleeping near the nurses so that they see what is happening to you.*  *And yet the health workers know how to give treatment. Most of the people they treat finally recover. In xx, they have never treated a patient and he or she doesn’t recover. But you hear about the other health facilities, that when you go there, you cannot recover.*  *What made me happy in this hospital is that the health workers are available in the hospital. Health workers are there and they are working, they are not like health workers in other health centers. When you call the health worker of here, they come and listen to what the patient has to say.*  *Most times, they respond to very ill persons, when you come very ill, they will rush to you very fast and leave others. They are patients who cannot talk nor walk especially those with too much fever*  *The first time I came here, on Thursday, because the doctors tried their best. You would see this one is measuring the temperature, taking my pressure and putting equipment in my chest and fingers, they were working together on only one patient*  *Like I told you, you cannot get appropriate treatment when doctors don’t know the illness they are treating. So if a big hospital like this does not have the tests, then what are they treating? It means they will not touch you, you can even die there*  *The health workers examine you and they take your blood samples, they test and see what you are suffering from. They won’t treat what they don’t know. They are not guessing. They want to know; “what is this person suffering from”*  *Because I know that when I come here, they will test me and first know what I’m suffering from before I start treatment*  *They come and start from the other end of the ward. When they reach you, they start asking how you are, they really try to ask you questions and you tell them how you feel. You start to tell them how you feel and if you still have stomach pain you tell them, or if there is anything paining you, you tell them. They prescribe for you drugs there and then and your attendant goes and buys the drugs, when you bring it, they come and inject you*  *I will start with the nursing care, though we are unable to do investigations but there is nurse patient relationship to explain to the relatives the care they expect to get. This is because when we get a septic patient in most cases the relatives are informed and explained to provide gloves to the people handling the patient. We also do drug administration and investigations though we cannot provide all but at least they are ordered for. Most time the nurse takes the sample and tells the caretakers to take out to the clinic and test that blood. To me those are the most important things. For septic patient we do isolation or burial nursing. Immunization is also important in some diseases to be immunized against*  *You asked about the point of care. I think what is working well is that the triage is being done in OPD, I don’t work there, but am sure they triage all patients and there are clinicians to see these patients in OPD. Every patient is first seen in OPD by a clinical officer or doctor*  *Another thing am proud of in this ward is that most of our patients are managed well and recover, for example this week, we haven’t lost any patient. Sometimes we take two weeks discharging patients, which is a good point for us.*  *In this facility what we are doing well is that we have a good laboratory which does most of the investigation; culture and sensitivity, LFTs, CBC, RDTs, HIV, TB and other tests. We actually have a full lab in OPD, gynecology and in fact they are three labs, there we are tick. The laboratory is well equipped, and we are getting good results.* |
| ***Things that did not go well/Participants were not happy about*** | 1. *Lack of water to use* 2. *Not being able to locate the right places to go to* 3. *Being asked to pay money* 4. *Emergency drugs not within the HWs reach* 5. *Do not have enough supplies* 6. *HW feels that the patients are getting care in peace mill due to lack of supplies* 7. *Nurses are not enough, difficult to give attention to each patient* 8. *Weak support supervision from management* 9. *No one to ask for accountability for staff absence* 10. *Patients take long hours waiting to get treatment when drugs are available* 11. *Untimely administration of drugs to patients/delay in administering doses as prescribed* 12. *Delay causes patients to stay in the hospital for a longer period* | | *Yes, those ones I have told you but also you find the water is also scarce in the hospital and I never expected to find such a crisis in town but more especially in the hospital where people are many and even the sick people who want to use water mostly.*  *They need to correct that one because there are some people who know how to read and some who don’t know how to read. There are those who know how to read that this room is for me or not but there are also others who don’t know how to read. There should be someone to direct you where to go, to show you that this Is for accident victims or so that you know where to go and where not to go. That one is a very big challenge, they send you somewhere and you find you have gone somewhere else.*  *I felt very happy and I said that even if my things are in the house let me ran at least I sell my property I come and take care of my patient to recover. And that’s what I did when I would run short of money, I would sell my goat and get money I come and pay for the bills and buy the drugs for my patient.*  *And then another thing that I would like to be changed first is thing of asking money from people. May be you have taken the patient thinking it’s a government hospital but the people are busy selling drugs and asking for money from people. Now me where do I get the money? If I have the money, then why I am I not in private?*  *What didn’t make me happy is that they are discharging people very late. I come from nearby, but there are people coming from very far. There are people going to XXXX, XXXX and you ask yourself, when will they reach home! They would have told them like yesterday that we are going to discharge you tomorrow so that they prepare themselves.*  *HWs would come to ask why they are not working on you or why they jumped you, sometimes they don’t respond to you, they make themselves busy…that one also didn’t make me feel good as a human being*  *There is where they give numbers from the reception. You go to get a number when you are very weak, you wait and you get tired. Remember sometimes health workers come late, it seems they first go for meetings up there. They finish working on you everywhere but you find those giving numbers are not there.*  *You have to first see the doctor, they assess you and then they give you a number. We all get numbers but at times there is when there are many patients compared to doctors’ available giving numbers.*  *At times patients can even collapse while waiting for the numbers. It is disturbing and therefore they should be giving those numbers in the wards or they give it to you there and then. Remember, if you don’t have it, there is nothing you can do, you can’t be admitted without it. I was therefore requesting that they quicken that process for us*  *There is where they give numbers from the reception. You go to get a number when you are very weak, you wait and you get tired. Remember sometimes health workers come late, it seems they first go for meetings up there. They finish working on you everywhere but you find those giving numbers are not there*  *I think it is because of the few doctors working there and the truth is that there are only two student nurses; they are measuring the heart, the pulse, asking you questions but the good thing, they work on you when you are very sick.*  *You find people sitting there waiting for the numbers while others are sleeping because they are weak. Those giving numbers are slower than the time you spend when the doctor is seeing you in OPD*  *Sometimes, even getting drugs, someone goes and waits in the line there for almost three hours just waiting for drugs, then after waiting, they tell you that the drugs aren’t there. That was not good*  *70 % because the health workers helped me a lot. Nurses, doctors. And I know if I had been seen by more senior doctors, I would be feeling much better than now. The most important thing is that I have recovered and we are going home.*  *If it was not that we do not have enough supplies many times, if we had enough human resource, it is all easy to implement all that. We always endeavor to give them when supplies are around. The truth I should say these patients do not receive the best care because even if you prescribe 2liters in one hour, you will find the patient has got 500ml. That becomes a challenge because we have so many numbers of patients. We find it difficult to give*  *attention to a single person so challenging especially for the nurse when she is only one.*  *I think supervision also in health care systems in the public settings I think have weak supervision much as we are saying health workers are not enough, even the few who are there are not around. No one asks them no one punishes them, no one asks them to answer their absence. So, I think it is a big gap that should be worked on even the few are not there, someone comes and works two days in two months. So, we have people who have not come since May, we are now finishing July. They have come to sit in meeting and they have now gone away and no one asks them and that affects the type of quality care also* |
| ***Recommendations for improving care for sepsis patients*** | 1. *Improve triage* 2. *Drugs should be available all the time not to ask attendants to buy their own* 3. *There should be security on the ward* 4. *Beds should be enough* 5. *Make scan equipment available* 6. *Reduce waiting time during testing* 7. *Enough HWs* 8. *Severely ill patients should be tested from the ward* 9. *Provide bedsheets* 10. *Educate patients in hygiene* 11. *Provide necessities for the poor and vulnerable* 12. *Attendants to choose where to test their patients when hospital equipment is done* 13. *HWs should teach attendants what to do for the patients at home* 14. *Increase drugs especially for major diseased* 15. *Increase beds* 16. *Increase the number of staff at triage* 17. *Samples to very sick patients be drawn from the bed (prioritized)* 18. *Patients should be given some other time to come back for review* 19. *Provide enough oxygen, cannulas giving sets should all be available for sepsis patients* 20. *Laboratory services should be made available in the hospital* 21. *Stock adequate drugs that meet the demand of the hospital* 22. *Advanced testing equipment for diagnosing sepsis* 23. *Need to change the attitude of HWs to treat patients as humans and the way they are* 24. *Sensitization of Health workers they have towards patients* 25. *Improve Health Workers’ Knowledge on sepsis* 26. *Train health workers to understand sepsis* 27. *Health education is minimal and should be* 28. *Provide essential supplies; equipment, medicines and make them available for patients* 29. *Sensitize HWs at lower facilities and clinics on early referral so that sepsis patients can reach the hospital early* 30. *Continue with ongoing ward meetings with administrators* 31. *Patients should also comply with drugs* 32. *Continuous CMEs to increase knowledge on sepsis to improve clerkship, triage and identification of emergency care* 33. *Sepsis patients need to be prioritized* 34. *Improve on allocation of nurses to handle very sick patients* 35. *Allocate a nurse to care for very sick patients* 36. *Improve the roaster to have at least two qualified nurses everyday* 37. *Should follow algorithm for managing sepsis* 38. *Every ward should have algorithms* 39. *Sepsis is hard to manage* 40. *Clinicians use their brains to manage sepsis* 41. *Ministry has not prioritized sepsis* 42. *Emergency unit should be fully stocked with equipment, staff* 43. *Well-equipped laboratory* 44. *Conduct CMEs and increase HWs knowledge* 45. *Improve supplies and make them readily available* 46. *Improve patient observations as prescribed by the doctor* 47. *More drugs and make them available for free* 48. *Equip the laboratory with reagents* 49. *Lab has equipment but not reagents to use* 50. *Adopt a system strengthening approach; supervision, being on ground; request enough medicine, and ensuring availability of equipment and drugs* 51. *Improve patient monitoring* 52. *Patients should be reviewed daily* 53. *Patients have to wait for transport to be able to go home and stay a day after discharge* 54. *Discharge should be discussed early* 55. *Patients discharged before time because of the many numbers and to create space for emergencies* 56. *Example of patients discharged before time* 57. *Patients having may questions whenever discharged before time and during COVID lockdown* 58. *Patients asked to go and recover from home to reduce Covid infections and to avoid crowding* | | *Another thing that I see can help is that when you bring the patient in a car, and then you take them in the prescription room, they will prescribe for you there and then. But when you go, there should be a system of attending to sick people quickly at least you can take them to bed that receipt can even find you on the bed.*  *Another one is that the drugs should be there all the time and the truth is all the drugs have never been there all the time. The times I have gone to the hospital there has always been at least a drug that is not there and you have to just buy it, so the drugs should also be there and they should also be caring. They should also put in a security guard so that they prevent people who liter the compound. Even the beds should be there so that people should stop sleeping down on the floor.*  *Drugs, pills, injections and testing the patient to see the disease that he or she is suffering from. Then they quickly bring help immediately they give her pills and injections and he or she recovers very fast*  *What is in good quality care; drugs must be there, the scan should be there and the other services that we have been complaining about. You find that there is one person working in the scan and yet you are about 50 patients, the first patients have handed in their forms. But because they took their forms first, even if you took there a very sick patient, they will first work on those ones and you sit with your patient until they reach you. That’s the service.*    *They would be doing a lot of good things like if they see I have brought my patient, they would be receiving her quickly and they start attending to her quickly, they would be getting drugs for her from the store, without having my money to go in excess. But when I brought her they first looked at her as if she was not sick.* |
|  |  |  | *The one to start with should be that of drugs, the second one is that they should help us in the scan room, there should be doctors at least two or three or even four. We should also be helped in the toilet; women should have their own toilets that are separate from the men. It is not safe to share a toilet with men in the hospital. If a woman goes there at night, a man who is from outside the gate can hide there and rape her. The care here, okay you find that the service here like some patients are for carrying. At least they should be tested from the ward or worked on first if they are sent for the scan. And because of lack of money and transport for taking the patient outside the hospital, they should help us and bring the testing machines to the hospital. But now, you put in transport to take the patient to another testing place and yet the hospital premises are very big…at least if there was a testing place here in the ward…at least those services would come here so that those patients can be worked on from here in the ward*  *Yes, they should put in the bedsheets, but they should also educate the patients and the care takers about the issue of hygiene. So that we stop defecating anywhere we find because that one also causes us ore diseases that we don’t know. Now I told you that not all of us are capable, even sometimes I don’t have a bedsheet to put on the patient’s bed so if I get a disease how will I manage*  *I was asking that they put for us the bedsheets and he blanket so that the patient can come and have something to cover themselves because they might have come from far and he got an accident and yet he is poor and he will not afford to buy*  *If you say that I want to go to xxx, they say no, the machines that they want are at Hi-Tech, that’s what they tell us. So if the machines are at Hi-Tech, if God can help and those machines come back inside XXX and we know that we have the machines that test people as a government hospital*  *The health workers should teach us what we should do at home so that the patient doesn’t get sick again. It can help us to avoid coming to the hospital every time, because even if you come to a government hospital, it is like you are in a private hospital, you keep paying, paying money for drugs. If they can at least put drugs in hospitals, so that we the poor don’t have to buy drugs*  *What I can talk about it that of increasing the drugs! the person is in a government hospital; I get confused when we buy drugs from private pharmacies. In this hospital, when you look at the patients here, you will find that there are those who can afford to buy drugs and there are those who cannot afford. On my side, I could afford but there were very many who could not afford. That thing of buying drugs from outside-what is the essence of bringing my patient where there are no drugs?*  *They should now put in drugs especially for pressure, stroke, diabetes, infections and even those other things they keep saying. Most of the patients in this ward, I got a chance to talk to a number of women. But you find some have kidney problems, liver, heart, pressure like that. So drugs for such problems, government should provide them for free because they are expensive. Then after that they put equipment for testing these diseases like x-ray and scan but they should also be for free*  *The most important one is the hygiene; it is very necessary because there are people who work in the toilet. They have been coming to the ward to teach people to observe good hygiene practices. Even us the attendants, we have been mindful of keeping the ward safe. You make sure your patient is clean and the bed is clean so that when the doctor comes, he doesn’t get surprised by the patient’s condition. So hygiene is very important, if people have come from different places, they need to be clean*  *They should keep treating and attending to people who come when they are sick instead of just keep looking at them. Now the patient has come but you are just looking at them, will your eyes heal them? It needs if the patient has come and you also were brought to the job and you earn a salary but you earn it from those sick people. Now to just look at them what does it help instead of just earning free government’s salary it needs if the person has come you treat her. You treat her well so that God can also see that this person has treated her well and you will get salary from the government with one heart. Because they pay you, and the patients’ care taker will also pray for you, that thank you doctor and if you are a nurse he will thank you for what you have done*  *They should add some beds because sometimes you can see that the patients are very many. 6It should be those things of testing you to see which disease that you are suffering from. Didn’t I tell you that they really cared about that child, they could come every night and they check to see and they change the drug. If I had not taken the child to the hospital, she/he was going to die for sure.*  *I would like to request… for diseases that are related to damage of the body intestines…, those are not easy illnesses. When your body organs get sick, it means you are always at risk, it means you have become weak. It is true I have been discharged, but I know, I may need some other time to come back and they check or test me a gain*  *Being quick on you, acting very fast to start you on treatment. You shouldn’t wait for long as I waited. They can be put you on drips even if you don’t have money, so that you can be regaining your energy and also feel good that they are treating you*  *So in this hospital, there should be enough oxygen for all patients plus cannulas and sets. Not to tell you to buy them*  *Now, you may find that the hospital has a person to operate those machines who always gets paid. I was requesting that they pay this money to the outside clinics so that the patient can go there and get that service for free. Hmm instead of paying someone and his work is to tell you to go to the outside clinic to be tested.*  *They should also stock the hospital…put enough drugs in the hospital so that us who come and are very sick, can get our drugs from here, after all this is still a government hospital. Not only that it is a referral, that is how we know it. When I went to the nearby health center, they sent me here, it means every other person has failed and I have to come here. When I come here and the government says no drugs, it means they are saying there is no money go and die.*  *The government has to buy the machines for testing the kidneys, even blood even the scan. Why are we paying and xx is a government hospital? Outside labs are very expensive, they ask for a lot of money and we are poor. You hear the doctor saying this test needs 30,000 to a poor person, that is too much money…*  *Yes, every Friday in case there is a CME on various topics. They are usually presented by clinicians, doctors, or the nursing staff. Then for the case of the supplies, IV fluids are essential. Nurses need to acknowledge that these are essential, instead of giving half doses to different patient, we should prioritize septic patients who are critically ill.*  *Ah ah I think CMEs because its low cost, it’s immediate and it has a better impact to start with. Then these other things of supervision, working with the human resource, working with procurement to have things, you see that takes time, some of them are beyond control but CMEs which focus on technical understanding, attitude of people ah yah like a holistic CMEs which is more of training, educating people about the ideal, like I think that can be the better thing to start with.* |
| ***Patients’ discharge experience*** | 1. *Not prepared early for discharge* 2. *Feeling that the patient was discharged before getting better* 3. *Patient retested at discharge* 4. *Attendant told and treated about the care for the patients according to the condition-before discharge* 5. *Caregiver was prepared before discharge and based on the patients’ condition* 6. *Asked to return to the hospital two weeks after discharge* 7. *Complaints of pain at discharge/little improvement* 8. *Following health workers’ instructions after discharge* 9. *Patient told to return to hospital if not feeling well* 10. *Appreciation that the patient was able to survive and go back home* | | *It was around 12p.m when I came they asked if I’m the caretaker of the patient. I said yes. They gave me a file and told me to go up there in OPD and get a number. As I was leaving, one nurse called me back. She asked me that am I the person taking care of the patient? I said yes. How do you call her? When were you born? I explained everything to her even my age. She then told me to put the file there and pick my papers and she told me that they have discharged us* |
|  |  |  | *He didn’t improve in any way apart from the drug that got finished and we had to look for money and we bought it from the private hospital. Then after we had bought it after two days, he took it for two days and the third day he refused those drugs. Even me I said we leave him because he can’t take the drugs yet he was not feeding and not drinking anything.*  *When they discharged us, he got high temperatures the whole body and the doctor himself checked him and said he doesn’t have malaria but when the fever went he started to sweat the whole body and every cloth was wet we were wiping him the whole night till morning then we called doctor and told him that this patient is having high fever we should check him.*    *He said that we have really tried to treat this woman. He actually talked to me on Friday and my mother died on Saturday. He said, “we have treated your mother and her doze is finished. We expect to discharge you anytime”. But I would really see that the health workers are planning to discharge someone and yet I wasn’t seeing any improvement. I was seeing that her condition was worsening but they were insisting that they were discharging her because her doze had been finished.*  *So the doctor took me to their room and told me the truth. He said, “be a strong woman, your doze is over. There are two issues; we will discharge you anytime but when we do, don’t expect your mother to be home and wake up or hold a stick to walk because she has a stroke. We now know that she has a stoke and a clot on the brain. So we are discharging you”. The doctor showed me and taught me how to care for her. They taught me about the hygiene, that I have to wake up and clean her teeth, wash her face, washing her and smearing Vaseline on her body to see that she doesn’t get wounds. The doctor also advised me to get a doctor who will be coming home to check on the patient and review her.*  *They came and checked her and said that we should go home and return after two weeks, we bring back the patient and they check her pressure and see where it has reached. After discharging us, they gave us a letter, we went and got drugs from the pharmacy. We got drugs that he will be taking from home.*  *Yes, I’m following the health worker’s instructions, because I also don’t have the ability but if the health workers had decided that I go home, it means he saw it fit for me to be discharged. He is the one who has been monitoring my progress*  *They gave me a discharge letter, they told me I first go home and I should not waste my money, but if I feel sick again, I should come back here*  *And when they tell you your sickness, you buy drugs and they start treating you until you feel better. So xx should keep it up on that. Eeh it feels good to be going home and you haven’t died*  *Right now, they came and reviewed me. The doctor saw me and he said that according to my condition, I have improved. I can continue to take drugs from home. They wrote some drugs for me to go and buy. I’m now waiting for the discharge form, once they write it for me, I will be leaving later today after talking to you*  *When they told me that I will be discharged today, I accepted because I also wanted to be home and back to my family. I also wanted to recover. So am sure when I take those drugs, I will get better* |
| ***In this section we summarize the pathway of a patient with sepsis and problems along the pathway that might lead to loss of patient from care. This analysis helps to identify obstacles at each step of the pathway, how to address it and expected outcomes.*** | | | |
| ***Pathway of care for sepsis patients*** | | ***Problem along the pathway that may lead to loss of patient from appropriate care*** | |
| ***Step 1:*** *Sepsis patient in the community* | | ***Problem 1:*** *Patient goes to the local HC or private clinic, treated and discharged or referred to XXX RRH. The patient stays home because they feel better or due to lack of transport*  *Some patients buy drugs for self-medication* | |
| ***Step 2:*** *Sepsis patient goes to HRRH* | | ***Problem 1:*** *If self-referred, the patient waits for a long time at triage to get admission number and meet a clinician. When the patient is sent on ward, he/she waits to see a clinician before getting the bed. There is one consultation room/resuscitation room for both male and female wards*  ***Problem 2****: Patient is asked to go for lab tests, ultrasound, x-ray services in private facilities outside the hospital.*  ***Problem 3:*** *Few referrals are made, and the patient is sent back to community* | |
| ***Step 3:*** *Patient is referred to xxx RRH* | | ***Problem 1:*** *There is no ambulance to take patients patient from lower HC to the referral site or home after discharge. Transport costs are very high specially during the Covid period. Transport was restricted. Most patients come from outside the districts* | |
| ***Step 4:*** *Patient arrives at HRRH* | | ***The patient is seen very first provided they have a referral letter***  ***Problem 1:*** *Patient is sent to the ward before admission, waits for longer hours. Triage nurses are few. If after 5pm, they find the lab is closed. Asked to come the following day*  *If not referred, the patient stays in the line waiting for registration and allocation of numbers*  ***Problem 2****: The patient is sent to the ward without a wheelchair to carry them. Caregivers ask sympathizer to help carry the patient to the ward* | |
| ***Step 5:*** *Patient arrives on the ward* | | ***Problem 1:*** *Nurses in the ward delay to receive the patient. The patient waits at the ward as clinician attends to other emergencies. There is only one consultation room and a clinician to review the patient before giving the bed*  ***Problem 2:*** *The ward if full and the patient sleeps on the floor and along the corridor which makes provision of care difficult. The patient is started on IV fluids while on the floor. After discharging some patients, the patient is offered a bed. Expected to provide own beddings*  ***Problem 3:*** *Patient is sent for lab tests outside the hospital due to break down of lab equipment. Caregiver suffers transporting the patient outside. Patient delays outside waiting for lab results. Returns to the hospital. The HW receives results and doesn’t fully explain to the patient due to lack of time.*  ***Problem 5:*** *There is no medication in the hospital for the prescribed drugs i.e. …….and the patient is told to buy. The patient is unable to buy. Delays to buy drugs and to receive timely treatment or attendant runs away*  ***Problem 6:*** *Patients don’t feel comfort because of poor poorly maintained toilets; other users throw pads, food, papers and other waste in the toilet and unable to flash. Patient fears infections*  ***Problem 7:*** *The patient is not monitored according to doctor’s prescription because they don’t buy all drugs, the health workers are few* | |
