# Supplementary material for: Facilitating high quality acute care in resource-constrained environments: Perspectives of patients recovering from sepsis, their caregivers and healthcare workers in Uganda and Malawi
Source: PLOS Glob Public Health. 2022 Aug 15;2(8):e0000272. doi: 10.1371/journal.pgph.0000272 (PMC10021962; doi:10.1371/journal.pgph.0000272)
Supplement: Attachment — Submitted filename: Response to Reviewers.docx [file pgph.0000272.s004.docx]

The Editor-in-Chief,

PLOS Global Public Health

30^th^ May 2022

**PGPH-D-22-00087:** **Facilitating high quality acute care in resource constrained environments: perspectives of patients recovering from sepsis, their caregivers and healthcare workers in Uganda and Malawi.**

Thank you and the reviewers for reviewing our submission and for providing detailed feedback. Please find below a point-by-point response together with track change and clean versions of our documentation. Concerns that were raised by the reviewers are shown in *italics*.

**Editor comments**

1. *It is a strength that data comes from two countries, it would be interesting to know how these countries happened to be selected for this study.*

**Response:** Thank you for this observation. This qualitative study was one among several research outputs by the African Research Collaboration on Sepsis (ARCS), whose designated Centres of Sepsis Research Excellence included Malawi and Uganda. Among other objectives, ARCS aims to build capacity in sepsis research within a network of ten countries across sub-Saharan Africa. The ARCS Cohort Study aimed to determine clinical outcomes for adult patients suspected with sepsis in Malawi, Uganda, and Gabon. We have provided this additional information about the selection of the study sites in the Study Design section (**page 4, line 78**).

2. *It’s really important that the reporting of this study adheres to current guidelines, e.g. COREQ* [*https://www.equator-network.org/reporting-guidelines/coreq/*](https://www.equator-network.org/reporting-guidelines/coreq/) *or SRQR* [*https://www.equator-network.org/reporting-guidelines/srqr/*](https://www.equator-network.org/reporting-guidelines/srqr/) *or both. Please go through the checklists and the associated guidelines in detail and ensure that you have considered carefully whether your reporting aligns with each point. In particular, consider points on relationship with patients, reflexivity, general methodological orientation, non-participation, setting of data collection, interview guide, field notes, data saturation/thematic sufficiency, participant checking.*

**Response:** Thank you for your comment. We appreciate the need to adhere to current guidelines for reporting qualitative studies. The information in our report largely adheres to both COREQ and SRQR guidelines, although some of the sections and information do not follow the exact flow of the guidelines. We have provided additional information and here is our response to specific sections raised in this question:

- *Relationship with patients:* This information is provided in the Data Collection section (**page 4, line 91**). Researchers established a relationship with the participants prior to data collection to introduce the study and build rapport before consenting.
- *Reflexivity*: We have now introduced a statement/ section on reflexivity in the report (see **page 7, line 137**).
- *General methodological orientation*: We have considered the methodological orientation of the study and would prefer to refer the design of the study as a qualitative descriptive study as described in the Study Design section.
- *Non-participation*: In Malawi, none of the participants declined to participate nor withdrew from the study. In Uganda, two patients declined participation. Although they had been discharged from the hospital, they expressed being too weak to participate in an interview. We have written this additional information in Data Collection section, **page 5, line 101.**
- *Setting of data collection*: Settings where data was collected is already provided for in the manuscript under Data Collection section, **page 5, line 98.**
- *Interview guide*: We have written additional information on how questions were delivered to the participants during the qualitative interview process. A semi-structured interview guide was used by the interviewers to guide the interview (see Study Design section, **page 4, line 93**).
- *Field notes*: There were no detailed field notes taken during the interview processes; instead, we utilised one-to-one in-depth discussions with the participants. Data analysis was based only on the transcribed data from these interviews. Any points written during the interviews were meant only to guide probing and follow-up questions.
- *Data saturation/ thematic sufficiency*: Data saturation determined data collection during the study. We acknowledge this information was omitted and we have since provided it in the manuscript (see Data collection section, **page 5, line 97**).
- *Participant checking*: The study incorporated participant checking as a way of validating the findings. While a session was conducted with healthcare workers in Malawi, COVID-19 prevented gatherings in Uganda. In both countries, follow-up interviews were also an opportunity for participant checking. We have included this information in the last sentence of the Data management and analysis section, **page 7, line 135.**

3. *It would be useful if you could explore further how your findings on professionalism and staff communication/behavior (and its association with overwork/potential burnout) relates to the existing literature in acute care – these are important findings that could be developed more.*

**Response:** This is an important observation. We have explored further on how our findings on professionalism and staff communication/behavior (and its association with overwork/potential burnout) relates to the existing literature in acute care. We have summarized findings from some studies (including scoping reviews) in acute care that have reported related findings (see Discussion section, **page 24, line 476**).

4. *It is particularly important to note that the data collection period included the start of the COVID pandemic - and to comment on whether your interviews and findings were influenced by this. In fact, COVID is not mentioned in your study - which is somewhat surprising*

**Response:** Thank you for this observation. In Malawi, data collection was concluded prior to the start of the COVID-19 pandemic. There was a slight delay in data collection in Uganda which coincided with the start of COVID-19 pandemic. We have since provided information on how COVID-19 affected the study in Uganda and access to care by the sepsis patients (**see page 5, line 104**). For the data collection process, some participants were uncomfortable to be interviewed for fear of COVID-19 transmission; the study team addressed these concerns through assurance and adherence to COVID-19 prevention measures. As far as experience of care was concerned, participants expressed delayed access to healthcare due to interruptions in the public transport systems which were introduced in partial lockdown.

5. *Purposive sampling is appropriate. I would like to know which of the research staff involved in the study were responsible for selecting the participants, as part of reflexivity.*

**Response:** Please refer to our Reflexivity statement mentioned in comment number 2 above. We have placed the statement on **page 7, line 137**, which describes how and who conducted the sampling of participants.

6. *It would also be interesting to have some idea about the denominator - of how many healthcare professionals and/or patients were available for recruitment over the period of time of the study.*

**Response:** The qualitative nature of the study meant that we sought breadth of lived experience through recruitment of patients/ and health workers representing the main cadres of staff and until thematic saturation was reached. We, however, did not collect information on total health workers staff during the study and therefore cannot provide an accurate denominator.

7. *Your inclusion criteria was adults who were discharged from the hospital after recover from sepsis. What of patients who failed to recover from sepsis - can you please comment on this?*

**Response:** This is correct. Participants included adults who were discharged from the hospital after recovering from sepsis. In addition, among caregivers, we included those whose patients failed to recover, and their insights and experiences have been included in the manuscript; these responses did not vary with the rest of participants.

8. *Who translated the transcripts? Did you use any software for the organization and analysis of your data?*

**Response:** Translation of the transcripts was conducted by local translators, working with the host organizations in the respective countries (Malawi Liverpool Wellcome Trust Clinical Research Programme in Malawi and Walimu in Uganda). They conducted both the translation and transcription of the audio recordings. Two authors (FL and OK) did the coding and analysis using Nvivo 12 (QSR international). We have now provided this information in the manuscript (Data management and analysis section, **page 6, line 128**).

9. *Thanks for noting the existing theoretical frameworks that influenced your deductive analysis of the data, can you also comment how you would characterize your analysis in terms of general qualitative paradigms/methodological orientation - e.g. some form of content analysis, grounded theory, qualitative case study etc. I think it would also be useful if you commented on your epistemological stance, e.g. was your position post-positivist, social constructivist etc?*

**Response:** Thank you for your comment. Please see our response to comment number 2 above. We refer the design of the study as a qualitative descriptive study as described in the Study Design section, describing experiences of patients, caregivers, and healthcare workers of sepsis care, shaped by their context, including social, cultural, or economic factors at individual or wider levels.

We would like to define our epistemological stance as having different facets including post-positivist (researchers’ ideas, values and experience influencing study findings and conclusion), as well as social constructivist (we valued independency of knowledge based on participants’ interactions with the society). However, we would like to define our stance as Naturalistic, where we studied how people experience sepsis care in the context of hospital stay. We acknowledge inherent values we had as researchers that might have influenced the study, but we reflect on these in the reflexivity statement (**page 7, line 137**) as a way of ensuring trustworthiness

10. *Does the “WASH” acronym apply to “space, strained water, sanitation and hygiene”? the letters don’t seem to quite fit.*

**Response:** The acronym WASH stands for “Water, Sanitation and Hygiene”. This has been presented in full in the abstract (**page 2, line 37**). We have restructured the sentence to show this distinctively.

11. *The data contains rich descriptions of the challenges to effective care for patients with sepsis. These findings are important and often resonate with what I’ve seen in other institutions in East and Southern Africa. Some of the descriptions of challenging behaviour and communication by healthcare workers are the most troubling and difficult data in the paper. Please expand on whether similar findings are found elsewhere in the literature, not necessarily for care of patients with sepsis, but in other acute care contexts - I think it’s important to situate these findings within a broader context.*

**Response:** Please see our response to comment number 3 above. We have summarized findings from some studies (including scoping reviews) in acute care that have come up with related findings. See in the Discussion section (**page 24, line 476**).

**Reviewer 1 comments**

1. *Methods: One referral hospital from each country was selected as the study site. Why were these two referral hospitals selected specifically? Are they representative of other hospitals that provide critical care in the country? The authors could elaborate on the differences between government versus other facilities to provide more context and rationale.*

**Response:** Thank you for this comment. Please refer to our response to editor’s comment number 1. This qualitative study was nested into an already existing clinical study in these two hospitals. The two are referral government hospitals in the two countries and are as such positioned to provide free specialized care that can be accessed by most local citizens whose socioeconomic status prevents them from accessing private facilities.

2. *Methods: Were there any participant exclusion criteria? Were any caregivers of patients who did not recover from sepsis included? These caregivers may have unique insights to provide to the study.*

**Response:** We excluded participants who were unable or unwilling to take part or give informed consent; did not speak a language understood by one of the field research team (not anticipated, as the team speak both English and the predominant local languages); were judged likely to be negatively affected by participation (e.g., due to distress) and patients that were too sick to participate in an in-depth interview.

Regarding caregivers whose patients did not survive, please refer to our response to comment 7 above from the editor. We included some caregivers whose patients died either in the hospital or at home (after discharge). We noted that they generally shared similar experiences with the rest of caregivers.

3. *Results: The authors can consider providing more demographic information about the patients and caregivers, e.g., age and length of hospital stay, to provide further context for the data.*

**Response:** Thank you for this suggestion. These data will be fully available following the publication of a complete cohort description (currently in preparation), which will reference this article once published

4. *Discussion: Given that there were some differences between the priorities identified by patients/caregivers and healthcare workers, the authors can consider addressing how to reconcile or explore this further.*

**Response:** This is an important observation. Although there were some differences in the priorities identified by patients/caregivers and healthcare workers, these differences are within the dimension of “Experience of Care”. Despite having this dimension as a priority across the groups, it presents the different viewpoints that patients/caregivers and HCWs have in areas of communication, respect, and emotional support. Delineation of the different viewpoints provides an opportunity for further research to explore how to reconcile these viewpoints, promote patient-provider interaction and, ultimately, to improve patients’ outcomes. We have provided additional information on **page 21, line 441**.

5. *Discussion: The authors can consider a discussion of limitations of the current study.*

**Response:** Thank you for this comment. This is already provided for in the manuscript. The penultimate paragraph in the Discussion section presents strengths and limitations of the study. For specific limitations, **please see page 25, line 500**.

6. *Minor comments. Please provide description of the acronym “OPD” on page 15 line 297. Please provide a higher resolution image of Figure 1*

**Response:** Thank you, this is well noted. We have provided the description of the acronym “OPD” (out-patient department). We anticipate that during typesetting, the journal editors will help us in ensuring that figure 1 has a higher resolution (see **page 17, line 348**).

**Reviewer 2 comments**

1. *The abstract while without headings, should nonetheless follow the IMRD format. Bits of the introduction are after the methods. It may be a tense issue, but it does not flow clearly.*

**Response:** Thank you for this observation. As rightly noted, this is a tense issue. We have changed this in the Abstract to portray that these were findings in our study than what other literature have found.

2. *The background reads well.*

**Response:** Thank you for the compliment. The idea was to situate the study into the global and local literature and interventions and highlight where gaps exist.

3. *In the methods section, the study settings need a little more detail to prove similarity. In one hospital (in Malawi, they seem to have some access to blood gases and cultures, which the Ugandan hospital does not have, does this imply a better equipped Malawian hospital and does this then not risk bias?)*

**Response:** Please refer to our response to the first comment of reviewer number 1. Both are government referral facilities, offering free emergency medical services and specialised care (see **page 4 line 83**). By care standards in sub-Saharan Africa, these facilities are where local citizens with low socioeconomic status would access specialised care at no cost. Though minor differences might exist (e.g., day-to-day operations, inventory and functioning of equipment), the two hospital settings were similar in terms of design, expected services and resources.

4. *In qualitative work, thematic saturation usually determines the final sample size. Did the cut off cover all three domains, or just one? This needs clarity.*

**Response:** Thank you. During the research process, we focused on two of the domains of saturation in qualitative studies, specifically, data saturation and thematic saturation. Authors FL and OK reflected on how to address emergence of repeated data during the data collection process. During data analysis, the rest of the authors continuously reflected on how the identified codes and themes were exemplified in the data. These approaches contributed to the determination of sample size cut off and the extent of our data collection and data analysis.

5. *What software was used to transcribe the recordings? What skillsets and experience did the transcribers have. What quality assurance was conducted to ensure agreement in thematic saturation?*

**Response:** Thank you for this inquiry. We used both manual approach and “Express Scribe” software to transcribe the interview recordings. Transcription of the recordings were conducted by the translation and transcription services of the respective host institutions (MLW in Malawi and Walimu in Uganda). Both institutions have skilled transcribers with more than 10 years of experience. Apart from member checking (see **page 7, line 135**), emerging themes were continuously reappraised and refined during discussions among the authors.

6. *Wouldn't having just 2 sites (hospitals) be a study limitation? While I agree with the findings being a LMIC practitioner? I am concerned there may be some generalisability issues. This can be addressed as a possible study limitation*

**Response:** We appreciate your concern and considered your observation. We would prefer not to consider this as a limitation. Qualitative studies are aimed at understanding in-depth issues than generalizing findings to wider sites. However, we acknowledge that approaches to care delivery, services and availability of resources are similar in referral facilities in these setups and can lead to similar experiences of care.

7. *The absence of treatment algorithms for sepsis can also be discussed within the context of the WHO-IMAI guidelines which were rolled out to SSA ministries of health. Is this not an implementation issue?*

**Response:** Thank you. There are many sources of potential guidelines for sepsis, from international organisations such as the WHO (IMAI) and the Surviving Sepsis Campaign. Hospitals also frequently produce their own, distilled versions containing local adaptions. We agree that the presentation of guidance to Ministries of Health is a key event in dissemination. However, this approach frequently fails unless there is a local stakeholder who takes ownership of the implementation, as the reviewer points out. We describe some of the ways in which the staff may be disenfranchised from this process and believe that a combination of improving the care for individual patients together with guideline roll-out is the optimal situation. We hope to take this on in the future.

8. *Did the study occur at similar pace in the different countries? The COVID pandemic was well and truly underway, how did the lockdowns and surges affect the study.*

**Response:** Thank you for this important observation. Please see our response to question number 4 from the journal editor.

Yours Faithfully

Felix Limbani on behalf of all the authors
